# DNA methylation levels in candidate genes associated with chronological age in mammals are not conserved in a long-lived seabird

**DOI:** 10.1371/journal.pone.0189181

**Published:** 2017-12-07

**Authors:** Ricardo De Paoli-Iseppi, Andrea M. Polanowski, Clive McMahon, Bruce E. Deagle, Joanne L. Dickinson, Mark A. Hindell, Simon N. Jarman

**Affiliations:** 1 Institute for Marine and Antarctic Studies, University of Tasmania, Hobart, Tasmania, Australia; 2 Australian Antarctic Division, Hobart, Tasmania, Australia; 3 Sydney Institute of Marine Science, Sydney, New South Wales, Australia; 4 Cancer, Genetics and Immunology Group, Menzies Institute for Medical Research Tasmania, Hobart, Tasmania, Australia; 5 Trace and Environmental DNA (TrEnD) laboratory, Department of Environment and Agriculture, Curtin University, Perth, WA, Australia; 6 CSIRO Indian Ocean Marine Research Centre, The University of Western Australia, Perth, WA, Australia; Austrian Federal Research Centre for Forests BFW, AUSTRIA

## Abstract

Most seabirds do not have any outward identifiers of their chronological age, so estimation of seabird population age structure generally requires expensive, long-term banding studies. We investigated the potential to use a molecular age biomarker to estimate age in short-tailed shearwaters (*Ardenna tenuirostris*). We quantified DNA methylation in several *A*. *tenuirostris* genes that have shown age-related methylation changes in mammals. In birds ranging from chicks to 21 years of age, bisulphite treated blood and feather DNA was sequenced and methylation levels analysed in 67 CpG sites in 13 target gene regions. From blood samples, five of the top relationships with age were identified in *KCNC3* loci (CpG66: *R*^2^ = 0.325, *p* = 0.019). In feather samples *ELOVL2* (CpG42: *R*^2^ = 0.285, *p* = 0.00048) and *EDARADD* (CpG46: *R*^2^ = 0.168, *p* = 0.0067) were also weakly correlated with age. However, the majority of markers had no clear association with age (of 131 comparisons only 12 had a p-value < 0.05) and statistical analysis using a penalised lasso approach did not produce an accurate ageing model. Our data indicate that some age-related signatures identified in orthologous mammalian genes are not conserved in the long-lived short tailed shearwater. Alternative molecular approaches will be required to identify a reliable biomarker of chronological age in these seabirds.

## Introduction

Ageing is generally considered to involve the complex interaction of accumulating organ, cellular and DNA damage which lead to functional decline and increased risk of disease and death [1]. Whilst ageing and senescence have now been observed in many wild animals, the rate of reproductive and functional decline varies [2, 3]. The effect of increasing chronological age is associated with key ecological characteristics including reproductive maturity [4–6], reproductive frequency [7], and mortality [8]. Given that many species lack measurable external changes with age, estimating chronological age is often difficult. Developing a non-lethal method for the accurate estimation of chronological age is an important first step for furthering our understanding of ageing in wild animals.

Within animal populations, age structure is a significant determinant of population growth rate and can reflect past population growth rates, for example, an over-representation of young individuals in fast-growing populations [9, 10]. Age has been associated with maturation, litter size, offspring body size and survival [11, 12]. Age structure can also be important for the management of wild populations including population viability studies for endangered species [13]. With the recent rapid advance of miniature sensors, free-living animal research is increasingly turning to the remote recording of activity and associated environmental conditions, which in combination with age information, could lead to a much broader view of animal communities [14]. Questions about avian physiological performance, foraging location and migration also require accurate age data to understand age-specific behaviours [14–16].

Molecular biomarkers have shown promise for age estimation in long-lived animals [17]. Chronological age is not always a reliable indicator of overall bodily function, or ‘biological age’, because individuals experience different exposure to damaging elements throughout life and their ability to repair damage varies [18, 19]. Several of the molecular changes involved in ageing can be quantified and these are an area of intense study in humans [18]. Recent evidence suggests that specific human behaviours associated with disease such as smoking [20] and alcohol consumption [21, 22] can be identified by quantifying epigenetic modifications. Whilst biological age has potential as a biomarker, we did not use damage-induced markers due to their innate difference from chronological age. Telomere restriction fragment (TRF) lengths have previously been shown to correlate with age in some mammals [23, 24]. TRF analysis has provided a good opportunity to study individual ageing over the lifespan of a single animal; however, it does not constitute a robust cross-sectional population biomarker [25, 26]. This is due to variation in telomere length at young ages and external effects influencing individual telomere attrition differently [27]. Sex and variable age-specific patterns of telomere loss have been reported in the thick-billed murre (*Uria lomvia*) [28] and wandering albatross (*Diomedea exulans*) [29]. Several molecular methods of ageing were reviewed by Jarman et al. [17].

Several recent studies demonstrate that specific epigenetic modifications can be used to determine individual chronological age [30, 31]. DNA methylation (DNAm) is the most studied epigenetic mark and involves the addition of a methyl group to the 5’ cytosine (5mC), of cytosine–guanine dinucleotides, hereafter referred to as CpGs [32]. The presence or absence of DNAm in important gene regions such as promoters can lead to changes in gene expression. Generally, higher levels of DNAm (hypermethylation) of promoter CpG islands results in stable gene suppression [33]. Methylation changes at specific CpGs have previously been linked to chronological age in humans [31, 34, 35], mice [30], humpback whales [24], dogs [36] and developmental stage of the bat wing [37]. Identification of DNAm changes in tissues that can be sampled non-lethally such as skin [24, 34] and blood [38, 39] provides the opportunity to develop an epigenetic model to estimate age in live seabirds.

The short-tailed shearwater (*Ardenna tenuirostris*) [40, 41], is the most abundant seabird species in Australian waters and are one of the few native Australian birds still harvested [42]. While there are many useful methods for age estimation in wild animals some are only applicable to dead animals or have a large margin of error [17, 43]. Some birds exhibit external features that allow the estimation of age or developmental stage [44], however the majority of seabirds, including shearwaters, have no morphological marks for age determination. Age estimation methods for colonial seabirds therefore rely on extensive and often labour intensive long-term banding studies [45]. Shearwaters breed at approximately five years of age in Australia and each year migrate 15,000 km to coastal areas around the Aleutian Islands and Kamchatka Peninsula [46]. Adults and newly fledged birds typically return to the same areas to breed if they survive the long migration; therefore, individuals can be recaptured allowing for both longitudinal and cross-sectional sampling [45]. These features make the shearwater an ideal species to test the applicability of mammalian age-related methylation patterns in birds.

We investigated the methylation levels of CpGs in several genes using DNA isolated from shearwater blood and feather tips. By using a closely related seabird genome, we identified a number of CpG sites within a set of candidate genes known to have age-related changes in DNAm in mammals. The DNAm levels observed in the long-lived seabird *A*. *tenuirostris*, did not support the hypothesis that DNAm changes with age recorded in mammals were conserved between the two vertebrate classes for these genes.

## Materials and methods

### Study sites

Blood and feather samples were collected from short-tailed shearwaters on Fisher Island, Tasmania (40°13'00.8"S 148°14'21.1"E) and from Fort Direction, Tasmania (43°02'48.7"S 147°24'58.5"E) under Department of Primary Industries, Parks, Water and Environment (DPIPWE) permit: FA 15230 and University of Tasmania (UTAS) Animal Ethics Committee permit: A14277. Samples for this study were collected from adults during incubation in December 2014, 2015 and from chicks prior to fledging in March 2016. During the surveys, all burrows were inspected by hand to determine occupancy, then the weight of the occupant and leg band number, if present, were recorded. During handling, birds were restrained in a dark, air-permeable bag and returned physically to their burrow of origin.

### Sampling

All individuals were weighed using a calibrated 1000 g or 2500 g Pesola handspring scale; blood samples were only taken from animals with a weight greater than 500 g. Venepuncture of the vein in the webbing of the foot was done with a 25-gauge needle. A few drops of whole blood (approximately 0.1–0.2 mL) were collected on a Whatman FTA® Micro (WB120210) card, dried and stored in sealable plastic bags. Three to five breast feathers were plucked and preserved in salt saturated dimethyl sulfoxide (DESS) solution. 31 known age feather samples were collected from Fisher Island in 2014 and 30 matched feather and blood samples from known age birds were collected in 2015. A further 20 matched chick blood and feather samples were also collected in 2016 from separate burrows at Fort Direction. The sex of the bird associated with each sample was determined with CHD1 real-time PCR and melt curve analysis [47].

### DNA extraction

Blood samples immobilised on FTA cards were extracted using Epicentre QuickExtract™ (QE09050) or MasterPure™ (MCD85201) DNA Purification Kits. For MasterPure™ extractions, a 3 mm punch was cut from an FTA card using a sterile punch and placed into a lysis solution containing Proteinase K and DNA was isolated according the manufacture’s instructions. DNA was eluted in 20 μL of TE. This protocol was also used for the extraction of 1–3 feather quill tips. Approximately 1–2 cm of the quill end was dissected with a sterile scalpel and placed into digestion buffer. Feather samples were centrifuged for an additional 5 minutes at 10,000 g at room temperature following protein precipitation and supernatant isolation to ensure no debris were carried through. 1–2 μL of isolated DNA was quantified with high sensitivity or broad range reagent kits for the Qubit® 2.0 Fluorometer and standardised to 50 ng/μL with TE.

Isolated feather or blood DNA (50 ng—1 μg) was bisulphite converted for DNA methylation analysis using a Zymo EZ DNA Methylation-Lightning™ Kit (D5030) following the manufacturers’ instructions. A dilution experiment was prepared using feather DNA for this protocol. Isolated genomic DNA was serially diluted in TE buffer at the following dilutions; 1:1, 1:5, 1:10, 1:100 and 1:1000 and subsequently bisulphite converted and amplified using shearwater bisulphite specific primers.

### Primer design

Candidate genes were identified from previously published data supporting an association between DNAm and age in mammals for specific CpG loci. Data published using the Illumina HumanMethylation 450k or 27k Array allowed for the ready identification of the CpG loci under investigation using the Cluster CG number and associated sequence. For other loci, position numbers relative to the transcription start site were used to identify their location within the gene. Target CpG conservation was checked using the University of California Santa Cruz (UCSC) Genome Browser [48] for the annotated human assembly (hg38) or chicken (*Gallus gallus*) assembly (galGal4). An unannotated genome (Assembly: ASM69083v1), sequenced to a depth of 33x, is available for the northern fulmar (*Fulmarus glacialis*), a long-lived seabird also of the Procellariidae family. As the shearwater at the time of writing did not have a published reference genome, sequences from the northern fulmar were used to design primers to amplify shearwater gene regions. If a target CpG was conserved in the orthologous chicken or fulmar gene the Reference Sequence (RefSeq) [49] was used as input for the online BLASTn suite [50]. Highly conserved sequences were returned from BLAST using the inbuilt Gnomon gene prediction tool to facilitate identification of orthologous genes [51]. These sequences were then checked for CpG loci, including any previously published loci and used to design primers for shearwater genomic DNA amplification.

Primers designed from northern fulmar reference sequences shown in Table A in S1 File were created using the online client of Primer3Plus (vsn.2.4.0) [52] and were split into *gene set 1* and *2*. *Gene set 1* consisted of initial mammalian gene targets, whereas *gene set 2* consisted of additional amplicons for ELOVL2, TET2 and two genes new to this study, MYOD1 and TRIM59. CpG loci were specifically excluded from primer pairs at this stage to simplify downstream design of bisulphite-converted primers. Each primer pair designed using this method was optimised with a temperature gradient (56.1°C– 64.7°C), no template control (NTC) and sequenced on an Applied Biosystems Genetic Analyzer 3130. Sequences were visualised in Sequencher (vsn. 4.10.1) and BioEdit (vsn. 7.2.5) [53] to identify base pair differences between the two birds and a short reference sequence for the shearwater was created. Once genomic amplification was achieved the new shearwater sequence was used as a reference to create new primers in MethPrimer [54] and Zymo Bisulfite Primer Seeker specific to bisulphite converted DNA (Table B in S1 File). Bisulphite specific primers were temperature optimised (50.2°C– 58.7°C) and run with NTC and genomic DNA controls to ensure specificity. All primer optimisation was visualised using pre-stain electrophoresis on 1.5% agarose gels running at 100 V for 50 minutes with SYBR® Safe (Thermo Fisher Scientific) or GelGreen™ (Biotium) dyes. PCR cycling conditions for genomic, bisulphite converted DNA and library preparation are described in Tables C-E in S1 File.

### Library preparation

To prepare the avian samples for targeted bisulphite sequencing on the Illumina MiSeq Platform, genomic DNA was bisulphite converted as described above. Up to 50 ng and 500 ng of feather and blood genomic DNA respectively was converted and eluted in 10 uL of M-elution buffer. Post-PCR products were diluted 1:10 and in-house identifier sequences were added to the end of the MiSeq universal primers with a ten round PCR. Samples were then pooled into a single 1.5 mL Eppendorf tube and diluted to 2 nmol/L. The library was prepared for sequencing on a Nano, Micro or Standard flow cell (Illumina) following the manufacturer’s instructions with 20% PhiX control.

### Data analysis

Fastq files were unpacked and Phred (Q) scores were assessed using commands for USEARCH v8.1 [55]. A stringent maximum expected error of 0.7 was used to filter poor quality reads. Known CpG sites were identified within target amplicons using a second script that used a unique ‘core’ sequence approximately five base pairs long before the CpG site. This approach reduces the chance of losing informative reads due to read end differences or errors in large base pair repeat areas. The methylation level for each site was calculated as number of methylated cytosines divided by the total number of reads for the given CpG pair and recorded between 0 and 1, where 0 is unmethylated and 1 is methylated (S10 File). A read depth of >100 was required to include a score in the final analysis; scores with a read count below this were discarded.

Linear regression of age and DNAm levels for CpG loci in amplicons from both gene sets was executed in R to aid in visualising the data. The R package ‘glmnet’ was then used to fit penalised lasso regularisation paths to variables in a generalised linear model (S11 File) [56]. We used a matrix containing all calculated ratios (predictors) for every CpG site for each gene target. Glmnet was then used to fit the DNAm data to age using alpha = 1 (lasso). In order to select λ (lambda), the penalty value, the cross validation function of glmnet was used. A penalised statistical method was used as the number of CpG sites scored was greater than the number of individuals used in the study.

## Results

### DNA yield from shearwater blood and feather samples

A total of 53 feather (47 adult, 6 chick) and 32 blood (30 adult, 2 chick) samples from individuals of known age were used to investigate the DNA methylation status of target genes. CpG loci in *gene set 1* (see Table B in S1 File) were investigated using DNA from 36 feather samples including 6 chicks (40–50 days old) and 30 adults (mean age = 11.7 years, range: 5–21). Matched blood samples from 14 birds were analysed in the same gene set (adult mean age = 12.5 years). Targets in *gene set 2* were analysed using DNA from 42 adult (mean age = 11.3 years, range: 2–21) and 2 chick feather samples. Blood samples from 32 individuals (adult mean age = 11.7 years, range 5–21) were also analysed for this gene set, 27 of these were matched to feather samples (Table 1). The sex of 27/30 (90%) known-age adults was determined using a real time PCR assay from blood spots [47]. S1 Fig shows an example of a successful melt curve analysis following real time amplification.

**Table 1:** Feather and blood samples used.

	Feather		Blood		Matched	
*Gene set*	Chick	Adult	Chick	Adult	Chick	Adult
*1*	6	30	2	12	2	12
*2*	2	42	2	30	2	25

**Fig 1:**
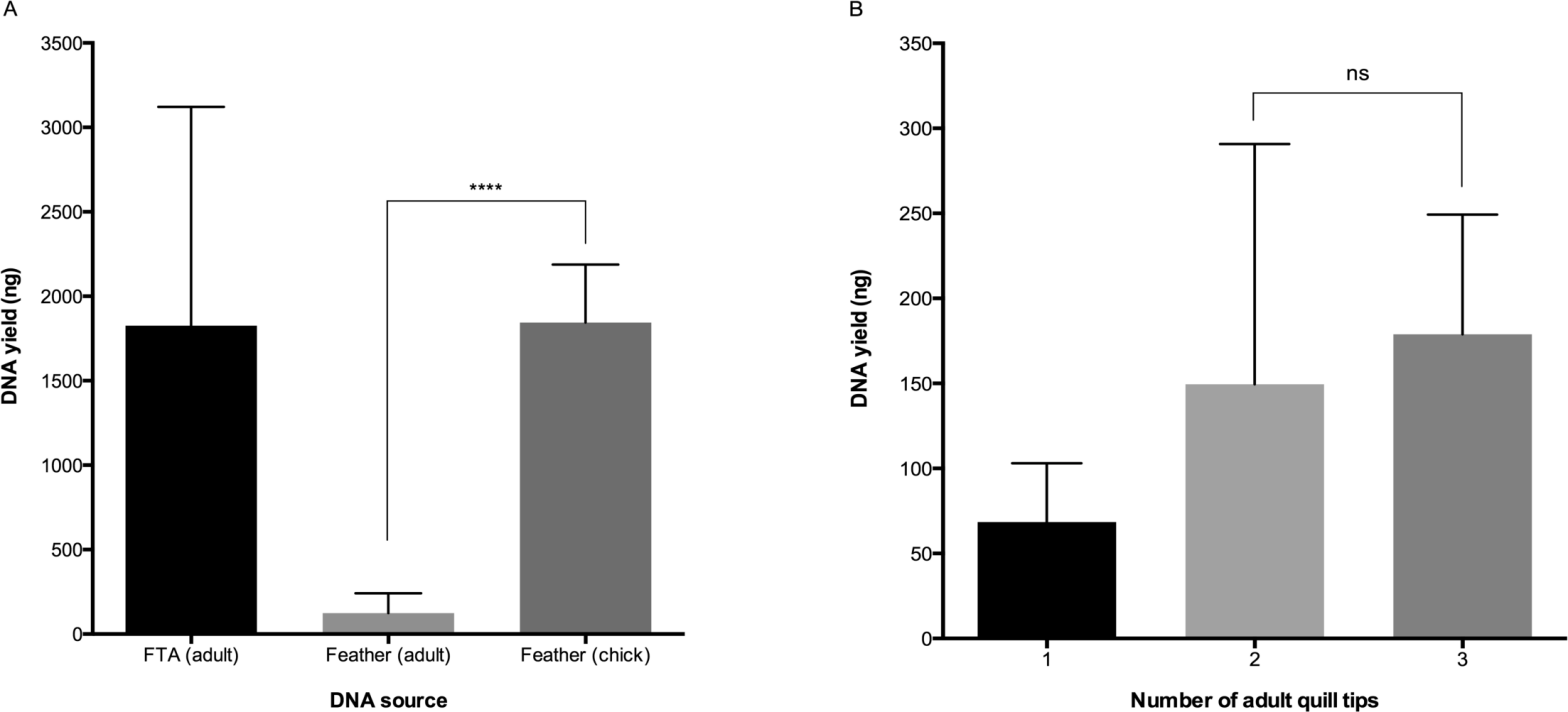
DNA yield varies with tissue source and age. **A**: Genomic DNA was isolated from blood stored on FTA cards (n = 132) and feather quill tips adult (n = 58) and chick (n = 6). DNA yield from adult feathers was significantly lower than chicks. **B**: The isolation of sufficient genomic material for experimental optimisation, bisulphite treatment and methylation analysis increases with the number of adult feather quill tips included in the extraction solution.

DNA yield was greater in feathers collected from chicks with an average total yield of 1844 ng (n = 6, ± 343) from one feather compared to an average of 124 ng (n = 47, ± 117) from adult feathers using the same extraction method (Fig 1A, Table F in S1 File, t-test: *p* < 0.0001). Total DNA yield was consistently high from shearwater blood samples stored on FTA cards with an average of 1826 ng (n = 30, ± 1295, Fig 1A, Table F in S1 File). DNA yield from adult feathers was dependent on the number of quill tips prepared for digestion. Extraction of a single quill tip yielded an average of 68 ng (n = 23, ± 34) of DNA whilst a two and three quill tip extraction yielded 149 ng (n = 25, ± 141) and 179 ng (n = 9, ± 70) of DNA respectively (Fig 1B, Table G in S1 File). Yield from one feather was compared to two (ANOVA: *p* = 0.0197) and three (ANOVA: *p* = 0.0194) quill tips. S2 Fig shows a quality comparison of DNA isolated from different avian tissues. Input of approximately 50 ng of DNA showed a high molecular weight band of genomic DNA when run on a 1% agarose gel indicating the successful isolation of high quality DNA.

### Amplification of shearwater DNA

For *gene set 1* a total of 13 genomic primers were designed from the fulmar sequence to target the first exon and promoter regions of each gene. Eight of these primers amplified the target whilst five failed to amplify template DNA. In *gene set 2*, seven primers were designed with two failing to amplify the correct product. In both gene sets positive products were sequenced and verified by BLASTn against the northern fulmar or chicken sequence. BLASTn metrics indicating the degree of conservation between fulmar/shearwater and human genomic regions are shown in Table K in S1 File.

Following amplification of target genomic DNA the products were sequenced on an ABI3130. This generated several short reference sequences from which we could design primers specific to bisulphite converted DNA. In *gene set 1*, nine primers were designed from the reference sequences and all but one of these amplified the correct converted product. In *gene set 2*, a further six primers were made and again one failed to amplify the correct product.

**Fig 2:**
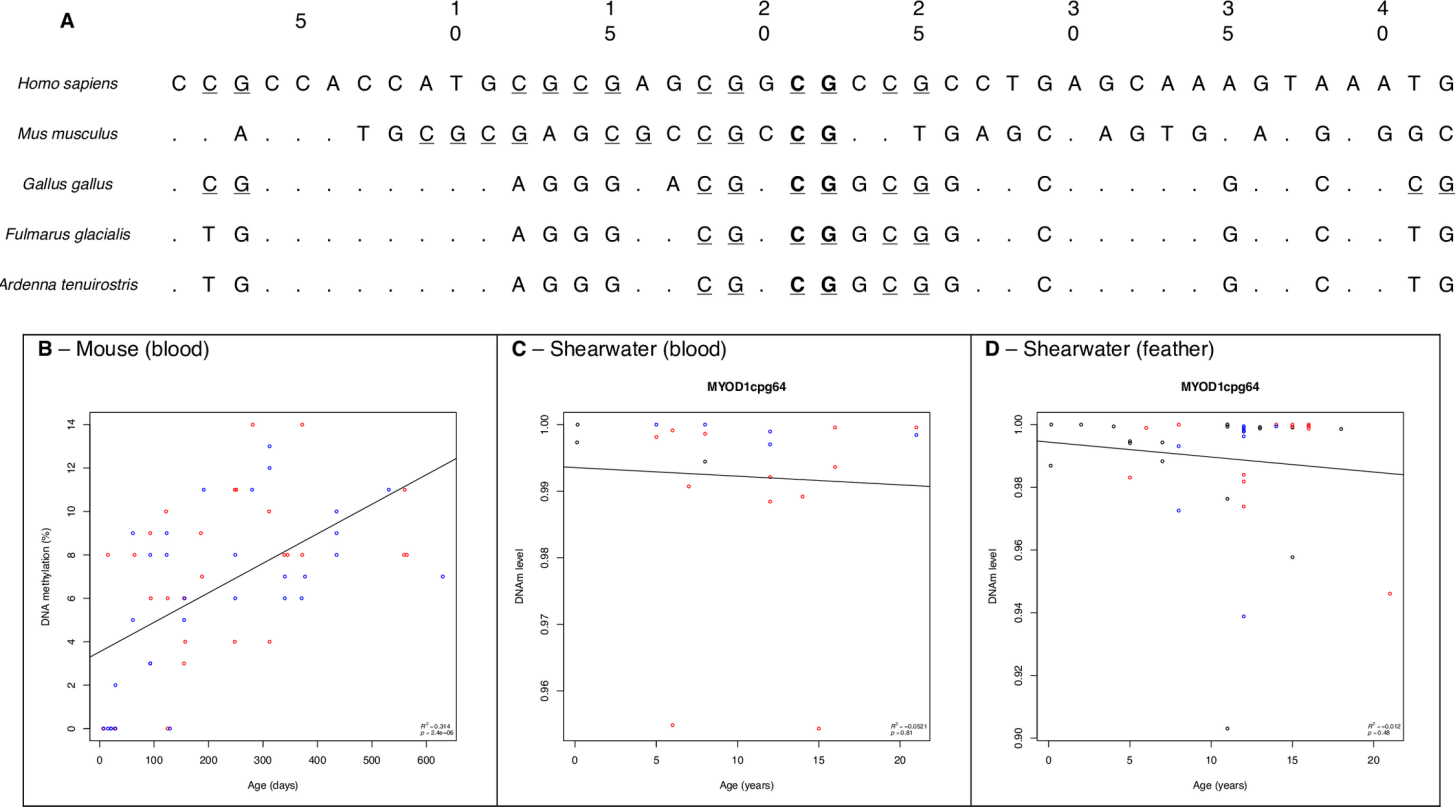
*MYOD1* sequence conservation in mammals and select birds. **A**: 20 base pairs are shown for both directions around an age related CpG loci (cg18555440, in bold) in the *MYOD1* gene in humans. Following the methods described in text, this sequence was compared to the mouse, chicken and northern fulmar genomes. Conserved bases are shown with a dot (.) and the base is given where there is a mismatch. Other CpG sites in the sequence are underlined and are shown for bird sequences. In this example, 4 CpG loci in humans were conserved in chicken, 2 were lost (pos. 12 and 14) and 1 was gained (pos. 41). However in fulmar and shearwater, 3 CpG loci were conserved whilst the remaining 3 in humans were lost (pos. 2, 12 and 14). Primers were designed from the fulmar sequence to amplify isolated shearwater DNA; for this short sequence there was 100% conservation between the two animals. A plot of the age relationship observed in mice at cg18555440 (Spiers et al. 2016) **Fig 2B**. The DNAm results for the same CpG site in shearwater are shown for **shearwater blood, Fig 2C** and **shearwater feather, Fig 2D**. Males and females are shown in red and blue respectively.

Overall there was relatively poor sequence conservation between mammalian and avian CpG sites. An example of sequence conservation between human, mouse, chicken, fulmar and shearwater sequences is shown for MYOD1 in Fig 2A. This figure shows the age associated cg18555440 and a further six CpG sites within the MYOD1 gene. Five of the sites are conserved within the chicken genome but the northern fulmar and shearwater sequence show further differences with only three sites of the original six present in shearwaters.

### Analysis of DNA methylation and age

As very little feather DNA was available for bisulphite treatment, which is known to be particularly harsh on DNA, we prepared a serial dilution of bisulphite-converted feather DNA to assess the potential amplification quality of diluted sample. Dilutions of a 10 ng/μL bisulphite converted DNA sample at 1:5, 1:10, 1:100 and 1:1000 all showed the correct 243 bp product for ASPA (S3 Fig). Based upon this result, subsequent bisulphite PCR used a 1:5 dilution of converted DNA (approximately 2 ng).

**Fig 3:**
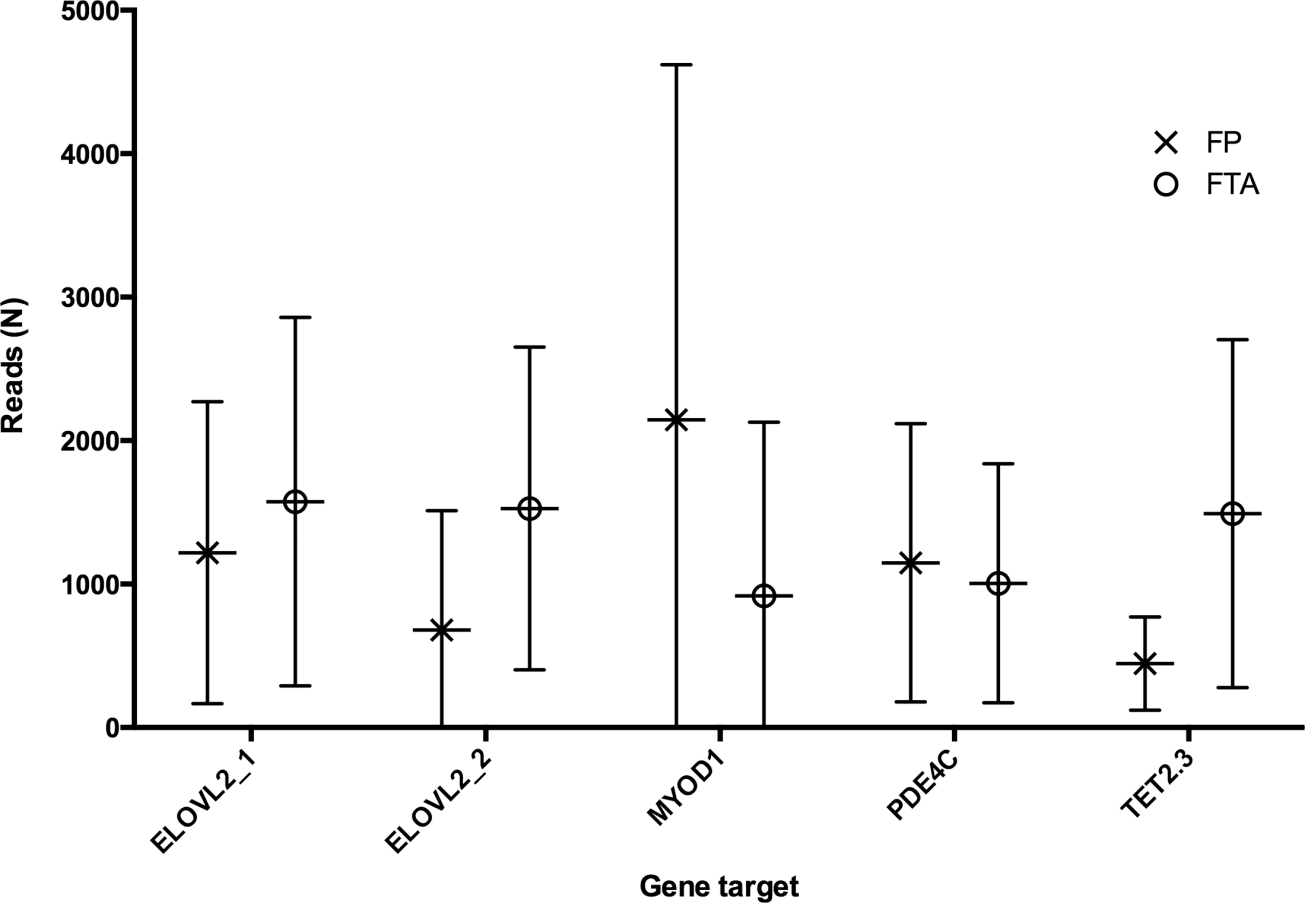
Total reads used to calculate methylation level in *gene set 2*. The average number of reads varied with each amplicon and tissue type (FP: feather, FTA: whole blood) being investigated. If the total number of reads for a single CpG loci was <100 the result was removed from further analysis.

For *gene set 1* blood and feather, run on Standard and Micro flow cells, the range of reads was approximately 10,000–100,000 and 500–10,000 reads respectively. For *gene set 2* all samples were run on the same micro flow cell and a standardised PCR input led to a read depth of 500–2000 reads (average reads per tissue shown in Fig 3). Two replicate feather samples were sequenced in *gene set 1*, a technical replicate (same individual different feather quill tip) for a chick and a run replicate (same DNA extraction) for a 12-year-old adult. The absolute difference in the reported DNAm level was calculated for each replicate as shown in Table H in S1 File. We report a mean difference of 5.84% and 5.04% in DNAm levels for the technical and run repeat respectively.

**Fig 4:**
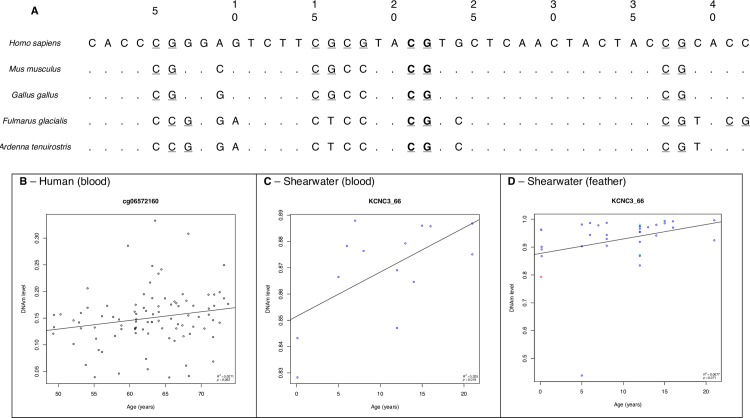
*KCNC3* sequence conservation in mammals and select bird species. **A**: 20 base pairs are shown for 3’ and 5’ directions around an age related CpG loci (27K: cg06572160, in bold) in the *KCNC3* gene in humans (*KCNC4/2* in *F*. *glacialis* and *G*. *gallus*). Following the methods described in text, this sequence was compared to the mouse, chicken and northern fulmar genomes. Conserved bases are shown with a dot (.) and the base is given where there is a mismatch. Other CpG sites in the sequence are underlined and are shown in the bird sequences. In this example, 4 CpG loci in humans are conserved in chicken, whilst 1 is lost (pos. 17). However, in fulmar and shearwater, 3 CpG loci were conserved whilst the remaining 2 in humans were lost (pos. 15 and 17). A plot of the age relationship observed in **human blood, Fig 4B** at cg06572160 (Rakyan et al. 2010). DNAm results for the same CpG site in shearwater is shown for **shearwater blood, Fig 4C** and **shearwater feather, Fig 4D**. A technical replicate is shown in red whilst a run replicate is shown in green for **D**.

The relationship (*R*^2^ = 0.314, *p* = 2.4e-06) between age and DNA methylation in an inbred mouse model for cg18555440 [57] is shown in Fig 2B. This result was directly compared with DNAm levels in blood and feather in our study (Fig 2C and 2D), indicating that an age relationship at this site is not conserved in *A*. *tenuirostris*. Fig 4A shows a similar comparison for the *KCNC3* gene and shows the loss of three CpG sites in fulmar and shearwater sequences. DNAm levels are shown for cg06572160 in humans (Fig 4B) as this site was previously associated with age and conserved in shearwater (Fig 4C and 4D, CpG66) [58, 59]. Sequences for ASPA were also compared in Panels A-C in S4 Fig.

**Fig 5:**
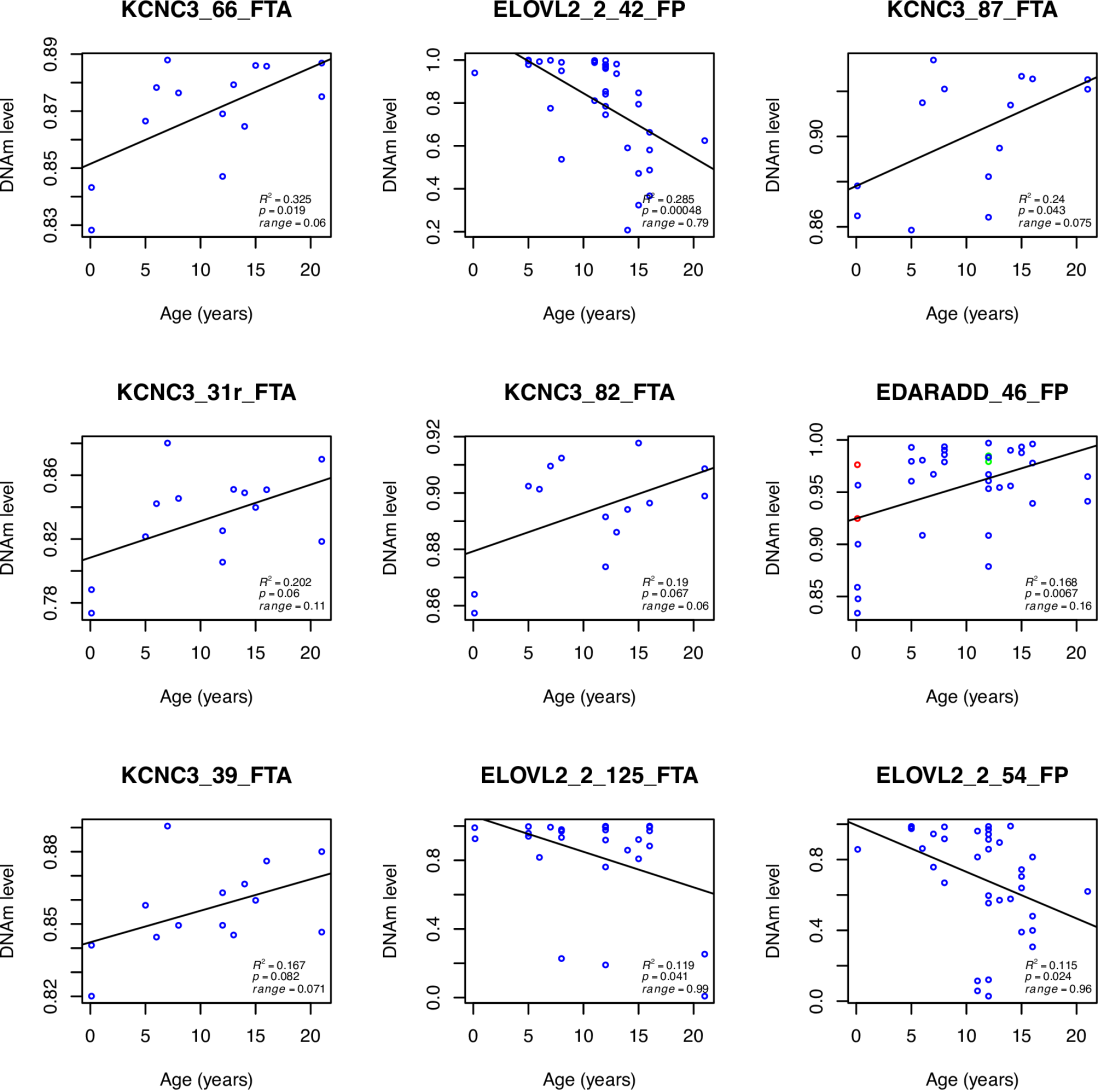
Methylation profiles by MiSeq analysis. Association of individual seabird proportion of cytosine methylation (*y*-axis) against age in years (*x*-axis) for the top nine CpG sites in blood (FTA) and feather (FP) samples. The best relationship with age based on *R*^2^ is shown from the top left to bottom right. A measure of the magnitude of DNAm change with age is also shown. A technical replicate is shown in red whilst a run replicate is shown in green in the *EDARADD* methylation plot.

The age distribution of samples used for the following results is shown in S5 Fig. The top nine CpG sites demonstrating the strongest relationship with age are presented in Fig 5. The highest coefficient of determination (*R*^2^) was 0.325 (*p* = 0.019) in CpG66 from *KCNC3* amplified from whole blood (DNAm range: 0.83–0.89). Five of the top 9 CpG loci were identified in *KCNC3* blood samples. CpG42 in *ELOVL2* (*gene set 2*) feather tissue was the next best site with *R*^2^ = 0.285 (*p* = 0.00048) but interestingly, we found a greater magnitude of DNAm change with age (range = 0.79). Three CpG loci from the same *ELOVL2* amplicon, in both feather and blood samples, appeared in the top results and all showed a conservation of the broad range of DNAm levels observed in CpG42. CpG46 in *EDARADD* feather tissue had an *R*^2^ = 0.168 (*p* = 0.0067) and a DNAm range of 0.85–1.0. Regression plots for *gene set 1* and *gene set 2* blood and feather CpG loci can be viewed in S2–S5 Files respectively along with the collated *R*^2^ values in Tables I-J in S1 File and raw data in S6–S9 Files respectively. A penalised lasso model was fitted to the collected data; however, due to the relatively poor correlations with age no CpG sites were selected at minimum or 1se values of **λ** (factor selection cut-off).

**Fig 6:**
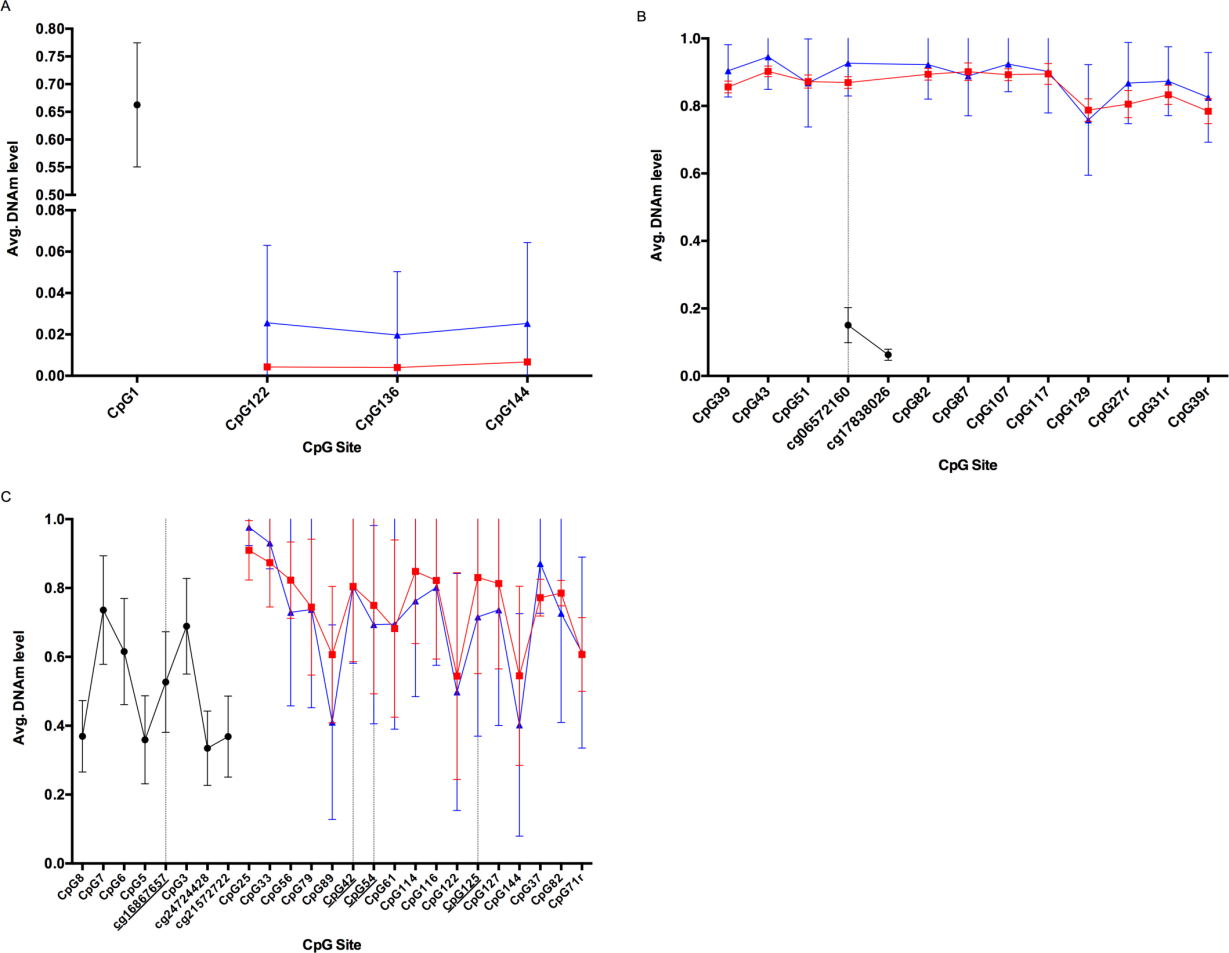
CpG site average methylation level comparisons. Human samples are shown in black, whilst shearwater blood and feather in red and blue respectively. **A**: Comparison of cg02228185 (CpG1) in *ASPA* analysed in Bekaert et al. (2015) and downstream CpG sites analysed in shearwater shows large differences in methylation levels. CpG1 was not conserved in the shearwater sequence (C>A in chicken, fulmar and shearwater). **B**: A direct comparison of cg06572160 in *KCNC3* analysed in Rakyan et al. (2010). Average DNAm levels in shearwater blood and feather samples were higher than in human blood. **C**: Average DNAm levels for a number of highly reported age related CpG sites are shown for *ELOVL2* in human blood (Bekaert et al. 2015). Whilst these markers could not be directly compared with the avian equivalent, CpG sites are shown downstream for both shearwater tissues. Underlined CpGs indicate those that are highly correlated with age in humans and appeared in the top nine results in our study.

Comparisons of average DNAm levels for highly reported loci were made between birds and humans where possible. Human cg02228185 in ASPA was compared to three nearby sites for the shearwater indicating large DNAm differences (Fig 6A). A direct CpG site DNAm comparison is shown between human and shearwater in Fig 6B, again showing a large difference in methylation level. A number of human promoter CpGs in *ELOVL2*, a frequently reported gene associated with age, were also compared to several downstream sites in shearwater (Fig 6C). A higher level of variation in DNAm was observed for these sites.

**Fig 7:**
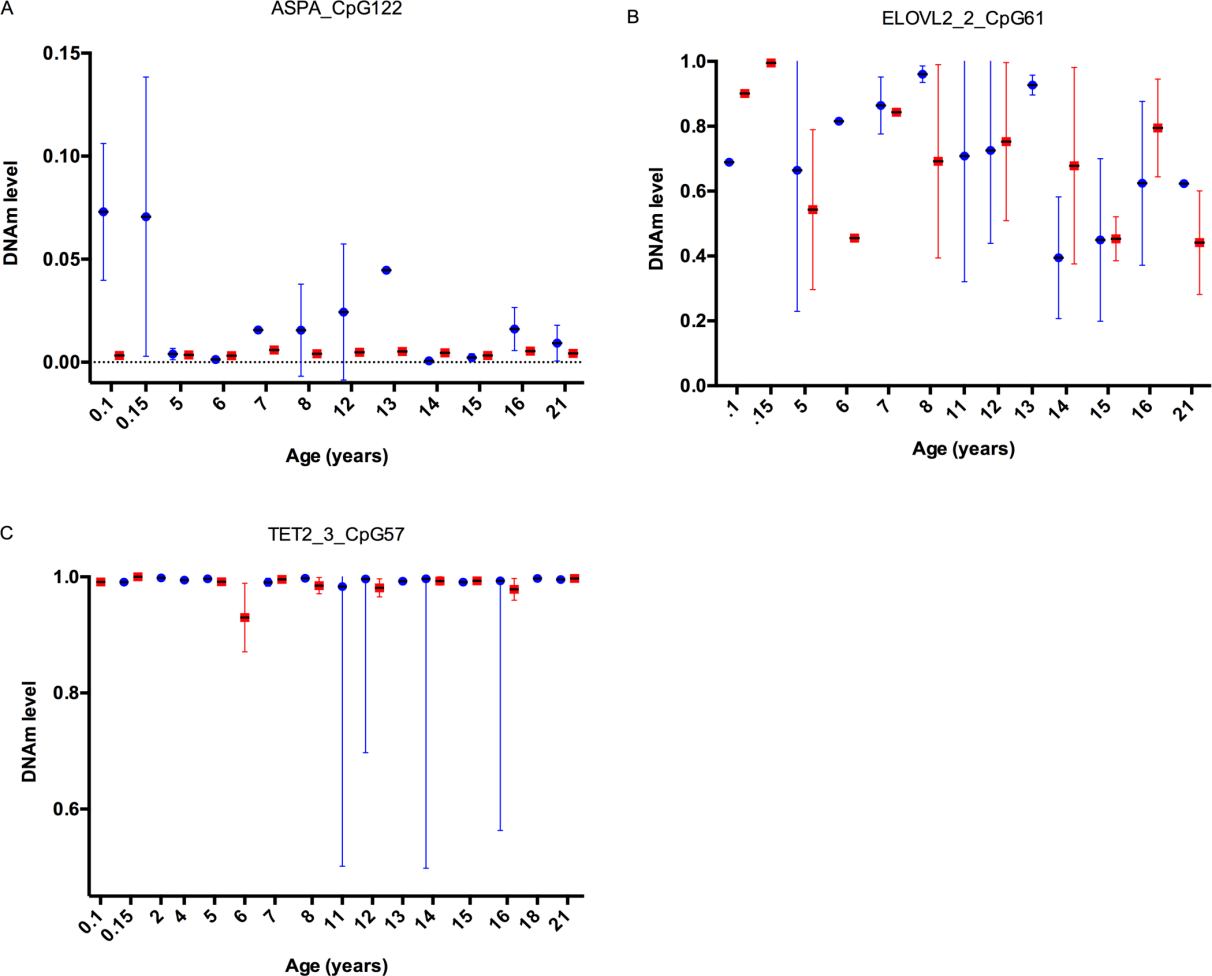
Comparison of DNA methylation levels in different avian tissues. We observed a large range of amplicon dependent DNA methylation levels. Here, the average DNAm levels for each age analysed in feather (blue) and whole blood (red) were plotted side by side to assess how conserved these signals are. Relatively low, stable (between tissue types) methylation levels are shown in **A)** for CpG 122 in *ASPA* from *gene set 1*, variable and less stable methylation levels are shown in **B)** for CpG 61 in the second *ELOVL2* amplicon from *gene set 2* and finally relatively high, stable methylation levels are shown in **C)** for CpG 57 in the third *TET2* amplicon in *gene set 2*.

DNAm levels were also compared between the two tissue types analysed, feather and blood. We observed large variances in DNAm levels between different genes as represented by randomly selected CpG loci within those genes (Fig 7A–7C). We observed relatively low and consistent levels of methylation in both feather and blood tissue for *ASPA* (CpG122 range: 0–0.15). In contrast, relatively high and stable levels of methylation were observed in *TET2* amplicons as represented by CpG57 (range: 0.5–1.0). CpGs in *ELOVL2* showed increased deviation in DNAm levels (range 0.2–1.0) and decreased parsimony between the tissues compared to loci in TET2 and ASPA.

## Discussion

This study is the first, to our knowledge, to target known mammalian age-related genes in a wild bird population. We successfully analysed methylation levels in 67 CpGs in 13 genes, but despite finding weak correlation with age in some sites, we were unable to create a model to estimate age using CpG methylation as done in other studies [24, 39, 60]. Age has been associated with DNA methylation levels in a number of different mammalian tissues including blood [31, 35, 38, 60, 61], skin [24, 34], saliva [62] and dentin [38]; we sought to investigate DNAm levels from two avian tissues: blood and feathers.

Blood samples provide a valuable source of DNA as avian red blood cells are nucleated and can yield up to 1.8 μg per 3 mm punch of an FTA card; greater than the DNA yield per equal volume of mammalian blood [63]. Current field techniques for blood collection generally require controlled low temperature storage in difficult climates or in locations with limited or no electricity. Our results further support the use of FTA storage cards that immobilise blood and pathogens on a paper-like matrix [64].

Feathers are a promising DNA source as they can provide DNA from the skin and recent studies have also shown that DNAm in mammalian skin samples are strongly correlated with age [24, 34, 35, 59]. While feathers are generally considered to be a more minimally invasive technique than taking blood samples they do present their own problems [65, 66]. First, our study shows the relatively low yield of high quality DNA can be limiting for repeated applications such as bisulphite sequencing. Second, condition of the feather and length of the sequence has been reported to affect PCR success [67], with one study reporting 60% and 50% amplification success with plucked and moulted feather respectively [68]. Of the failed PCRs in this study, sequence length did not appear to play a role. Third, low yield and quality, can lead to unnecessary laboratory repetition and ultimately re-sampling of bird populations that otherwise may not be required [66]. In our study, DNA yield from feathers was an issue as there would generally not be enough material for more than two sequencing runs. Given that our results indicate some differences between blood and feather, we would suggest that multiple tissues continue to be tested until a biomarker of age is discovered. But the feasibility of feathers as a tissue source for ageing needs to be assessed for each study. Further, the blood sampling method described allows the long-term storage of samples and numerous re-extraction opportunities from a single blood spot allowing access for future techniques or validation studies [64].

Amplifying age-related regions in birds based on previously defined mammalian targets can be challenging due to poor sequence conservation. Indeed, the examples shown in Figs 2 and 3 were the only cases of exact CpG site conservation in our bird sequences. Our study was helped by the availability of a closely related bird genome sequence. These examples highlight the difficulties faced when targeting specific mammalian biomarkers of age in non-mammals, but also shows that these targets can provide a solid foundation from which to begin investigations. Previously, primers used to amplify conserved sequences in the humpback whale (*Megaptera novaeangliae*) were designed using a closely related dolphin species (*Tursiops truncatus*) [24]. In this study, samples were then analysed with the PyroMark system, which compared to the MiSeq can have a higher read length but lower throughput [69]. Using the Illumina MiSeq Micro and Nano flow cells we were able to sequence to an average depth of 2100 reads per sample. This technique allowed us to calculate DNAm in a similar way to other studies [60].

In shearwater blood five of the top nine age-related loci came from CpG sites in the *KCNC3* locus. This gene has previously been identified to be age related (cg06572160, *r* = 0.70) in human dermis, epidermis and blood [59]. The protein encoded by *KCNC3* is an integral membrane protein that mediates potassium ion permeability and has an established role in adult-onset neurodegeneration in humans [70]. Hypermethylation of this gene may support its role in the gradual decline of cellular function with age [59]. It is interesting to note that whilst we observed changing DNAm, the overall level for this site is much higher in *A*. *tenuirostris* than in humans, possibly indicating regulation differences. In shearwaters the DNAm range (0.78–0.94) is quite small, so methylation levels need to be measured with large sequencing depth to detect differences. Additionally, samples from chicks are included and influence the slope whilst adults do not show a strong relationship (e.g. CpGs 87 and 82), and therefore offers limited predictive capacity. Some CpG sites (e.g. 31r and 39) showed a stronger hypermethylation trend in adults without inclusion of chick data.

The greatest difference in methylation levels with age (range: 0.2–1) were found in CpG sites in the fatty acid elongase 2 gene (*ELOVL2*) and were the second most reported in the top nine loci. Hypermethylation of sites in this gene during aging has been reported in human blood (cg16867657, *r* = 0.91) [61] and is being considered for forensic application [71]. *ELOVL2* and *5* have been characterised in chicken liver and reported to be different from other species due to their ability to elongate docosapentaenoic acid, however no methylation studies have been carried out [72]. Our results contrast with previous mammalian studies as the strongest observed change was observed in DNA isolated from quill tips (dermis) rather than blood and shows hypomethylation with age. Therefore, whether methylation is an indication of age only or is functionally correlated with ageing requires further study. Another top gene identified was EDAR associated death domain (*EDARADD*). CpG sites from *EDARADD* have been previously identified to correlate with age in human blood (cg09809672) [73] and saliva samples (*r* = -0.81) [62]. However, in our study we observed DNAm saturation for older individuals and relatively large variation in chick samples indicating that it is unlikely to be of use for shearwater ageing.

Shearwaters have a maximum lifespan of 40 years. Our samples covered approximately half of this range (chicks– 21 years). Previous models report age estimates with an error of approximately 3–6% the lifespan of the animal [24, 35]. If a strong age relationship were present in the sites investigated it would be expected to estimate age in birds that fall within this range. Nonetheless, our study could be strengthened by the addition of older individuals. Unfortunately the chance of capturing older individuals diminishes with each season due to mortality [45]. Despite the shorter lifespan of the shearwater compared to humans and whales, ageing-related DNAm changes are theoretically detectable. Several genes have been identified in mice which have DNAm changes on specific cytosines from a number of different tissues from mice ranging from 2.9 to 35.2 months [30]. This evidence would suggest that the DNA methylation changes observed in ageing are conserved between short and long-lived animals [17].

In this study, we focused on DNAm levels at specific sites as this approach has been used for all molecular age biomarkers developed so far [24, 39, 59]. However, an alternative approach is to investigate genome-wide DNA methylation, which in two studies of chickens (*Gallus gallus*) has indicated a relationship with age [74, 75]. Whole-genome scale methylation observations from 7-day-old chickens indicated that chicken tissues generally displayed methylation patterns similar to that in other animals. Compared to the pattern in mammals, DNAm was enriched in gene bodies and promoter methylation was negatively correlated with gene expression, indicating a suppressive role [75]. Genome-wide studies have so far focused on investigating developmental or tissue specific methylation and have included few adult samples. An observation from one study showed differing overall methylation levels between tissues in three strains of 14-week-old chickens [76]. These studies further our understanding of bird epigenetics and indicate that genome-wide techniques are a suitable approach for the detection of DNA methylation and therefore may be suitable for discovery of age-related genes.

In conclusion, we present a widely applicable and high-throughput technique to quantify DNAm levels in candidate age-related genes in long-lived seabirds. While we were unable to identify a combination of CpGs that would constitute a suitably strong biomarker of age in the short-tailed shearwater, we have identified several CpGs that were weakly correlated with age that warrant follow up investigation with a larger number of samples or wider age range. Our results show that an approach focusing on candidate genes identified in mammals is not a straightforward way to identify genes with age-related DNAm changes in birds. Future studies of a greater number of CpG pairs should expand the list of age-related sites in seabirds. Alternative methylation analysis techniques such as digital restriction enzyme methylation analysis (DREAM) and reduced representation bisulphite sequencing (RRBS) could be used.

## Supporting information

S1 Fig qPCR sexing assay of *A*. *tenuirostris*. DNA blood stored on FTA cards was used to amplify a small amplicon in the CHD1 gene. Melt curve analysis reveals a double peak for females (n = 4, green) and a single peak for males (n = 7, red). A NTC (n = 1, blue) shows no contamination. A third primer dimer peak is also seen for female samples at approximately 72°C.
(TIF)

S2 Fig Isolated DNA from blood and feather of *A*. *tenuirostris*. A QC gel indicating the isolation of high quality genomic DNA from shearwater tissue samples. FTA: DNA isolated from blood stored on FTA cards, FPa: plucked breast feather from adult, FPc: plucked breast feather from chick. Approximately 50 ng of DNA was loaded into a 1% agarose gel and run at 80 V for 30 minutes.
(PNG)

S3 Fig Bisulphite converted PCR. A serial dilution of bisulphite converted DNA isolated from a plucked breast feather. In this example a 243 bp target region of the predicted ASPA gene in the shearwater was amplified using bisulphite specific primers. 100 ng of genomic DNA was bisulphite converted and eluted in 10 μL of elution buffer. At an approximate original concentration of 10 ng/μL this converted DNA was then serially diluted to 1:1000 and 1 μL was used in the reaction mix. A 2% Agarose gel running at 100 V for 50 minutes was used to visualise the resulting amplicons.
(PNG)

S4 Fig ASPA sequence conservation in mammals and select bird species. **A**: 20 base pairs are shown for 3’ and 5’ directions around an age-related CpG loci (27K: cg02228185, in bold) in the ASPA gene in humans, mouse and birds. Following the methods described in text, this sequence was compared to the chicken and northern fulmar genomes. The resulting matches were aligned with the human sequence, conserved bases are shown with a dot (.), missing bases with a dash (-), and the base is given where there is a mismatch. In this example, the age-related CpG site in humans was not conserved in any other species. Primers were designed from the fulmar sequence to amplify isolated shearwater DNA to examine other CpG sites in the gene. **B:** The correlation of ASPA DNA methylation at this site [38] compared to **C:** the average methylation levels observed in shearwater blood and feather.
(TIF)

S5 Fig Age distribution of *A*. *tenuirostris* used in this study. The known ages of all birds used in this study are shown split into those used for feather quill tip (FP) and whole blood (FTA) methylation analysis.
(TIFF)

S1 File Editable excel spreadsheet of all supporting tables mentioned in text. (XLSX)

S2 File Regression plots for *gene set 1* CpG loci in blood (*A*. *tenuirostris*). (PDF)

S3 File Regression plots for *gene set 1* CpG loci in feather (*A*. *tenuirostris*). (PDF)

S4 File Regression plots for *gene set 2* CpG loci in blood (*A*. *tenuirostris*). (PDF)

S5 File Regression plots for *gene set 2* CpG loci in feather (*A*. *tenuirostris*). (PDF)

S6 File Proportion data for *gene set 1* CpG loci in blood (*A*. *tenuirostris*). (XLSX)

S7 File Proportion data for *gene set 1* CpG loci in feather (*A*. *tenuirostris*). (XLSX)

S8 File Proportion data for *gene set 2* CpG loci in blood (*A*. *tenuirostris*). (XLSX)

S9 File Proportion data for *gene set 2* CpG loci in feather (*A*. *tenuirostris*). (XLSX)

S10 File Python script used for calculating methylation proportion of specific CpG sites. (PY)

S11 File R script that does linear regression for each CpG site and uses the package ‘glmnet’ for lasso predictor selection. (R)
