# Supplementary figures and images for: DNA methylation levels in candidate genes associated with chronological age in mammals are not conserved in a long-lived seabird
Source: PLoS One. 2017 Dec 7;12(12):e0189181. doi: 10.1371/journal.pone.0189181 (PMC5720723; doi:10.1371/journal.pone.0189181)

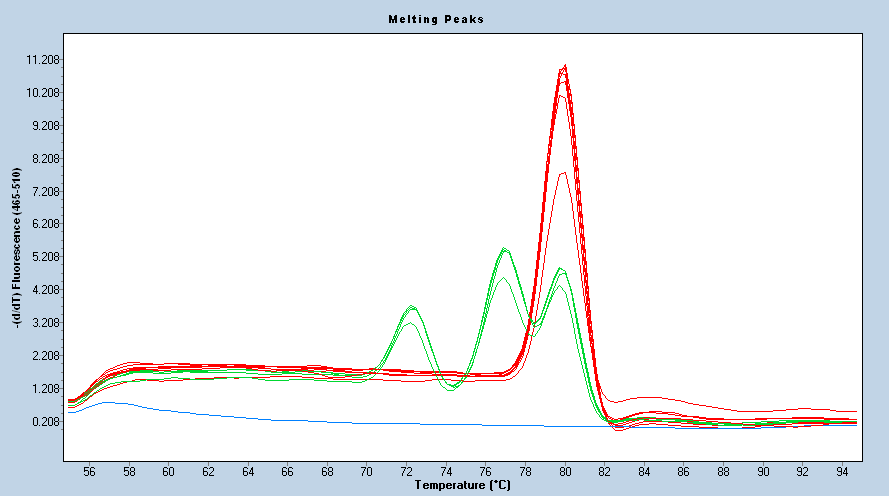

Supplement: S1 Fig — DNA blood stored on FTA cards was used to amplify a small amplicon in the CHD1 gene. Melt curve analysis reveals a double peak for females (n = 4, green) and a single peak for males (n = 7, red). A NTC (n = 1, blue) shows no contamination. A third primer dimer peak is also seen for female samples at approximately 72°C. (TIF) [file pone.0189181.s001.tif]

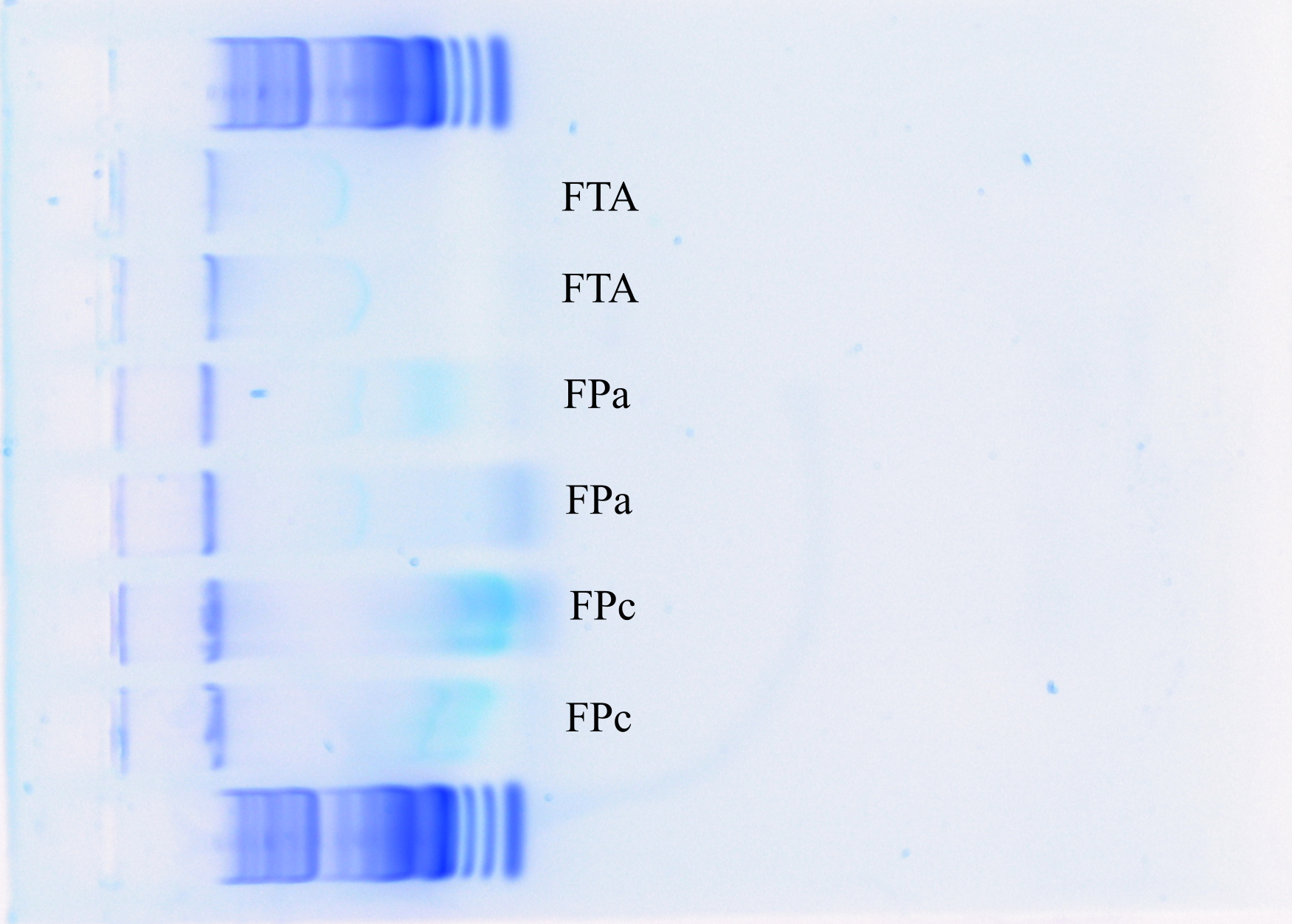

Supplement: S2 Fig — A QC gel indicating the isolation of high quality genomic DNA from shearwater tissue samples. FTA: DNA isolated from blood stored on FTA cards, FPa: plucked breast feather from adult, FPc: plucked breast feather from chick. Approximately 50 ng of DNA was loaded into a 1% agarose gel and run at 80 V for 30 minutes. (PNG) [file pone.0189181.s002.png]

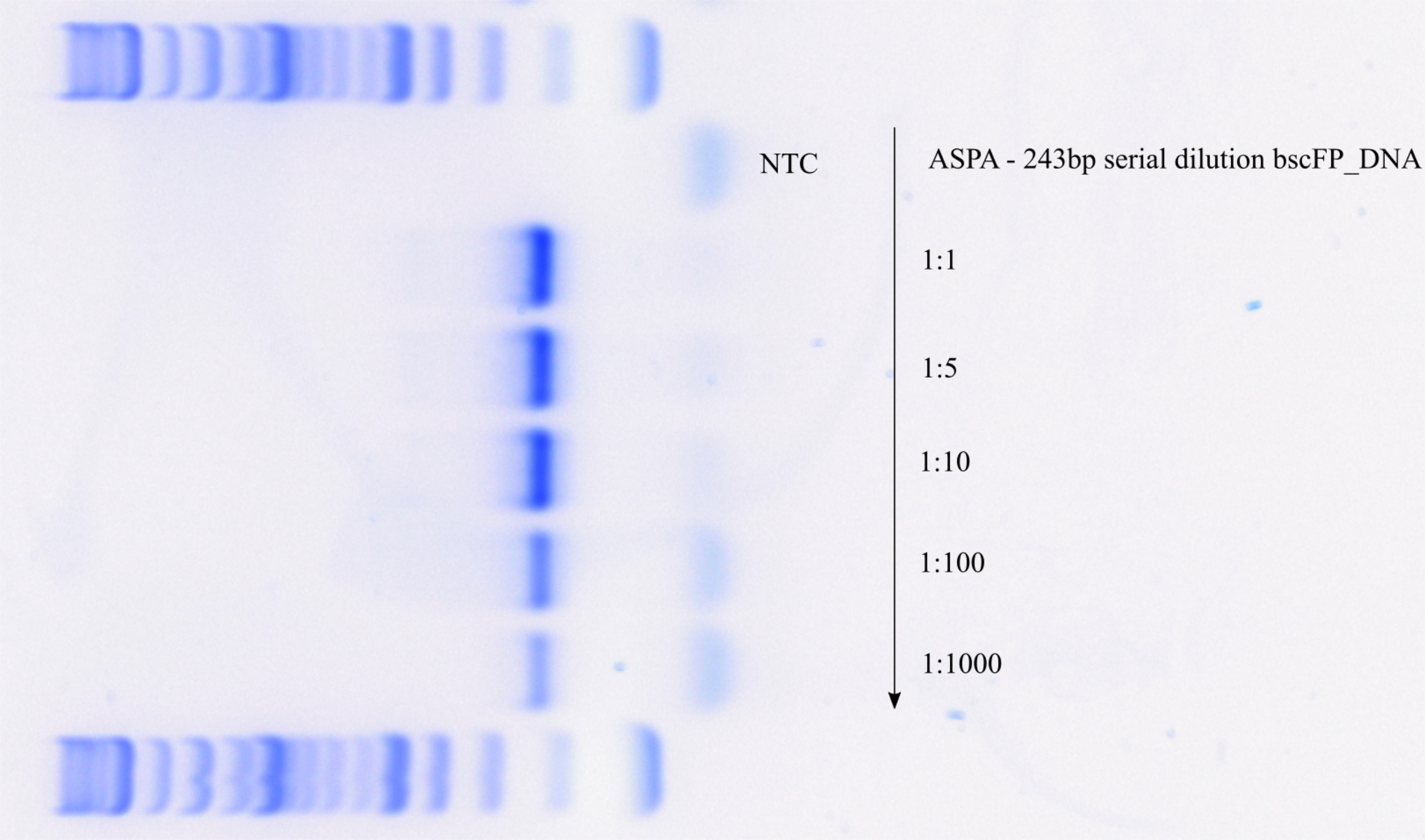

Supplement: S3 Fig — A serial dilution of bisulphite converted DNA isolated from a plucked breast feather. In this example a 243 bp target region of the predicted ASPA gene in the shearwater was amplified using bisulphite specific primers. 100 ng of genomic DNA was bisulphite converted and eluted in 10 μL of elution buffer. At an approximate original concentration of 10 ng/μL this converted DNA was then serially diluted to 1:1000 and 1 μL was used in the reaction mix. A 2% Agarose gel running at 100 V for 50 minutes was used to visualise the resulting amplicons. (PNG) [file pone.0189181.s003.png]

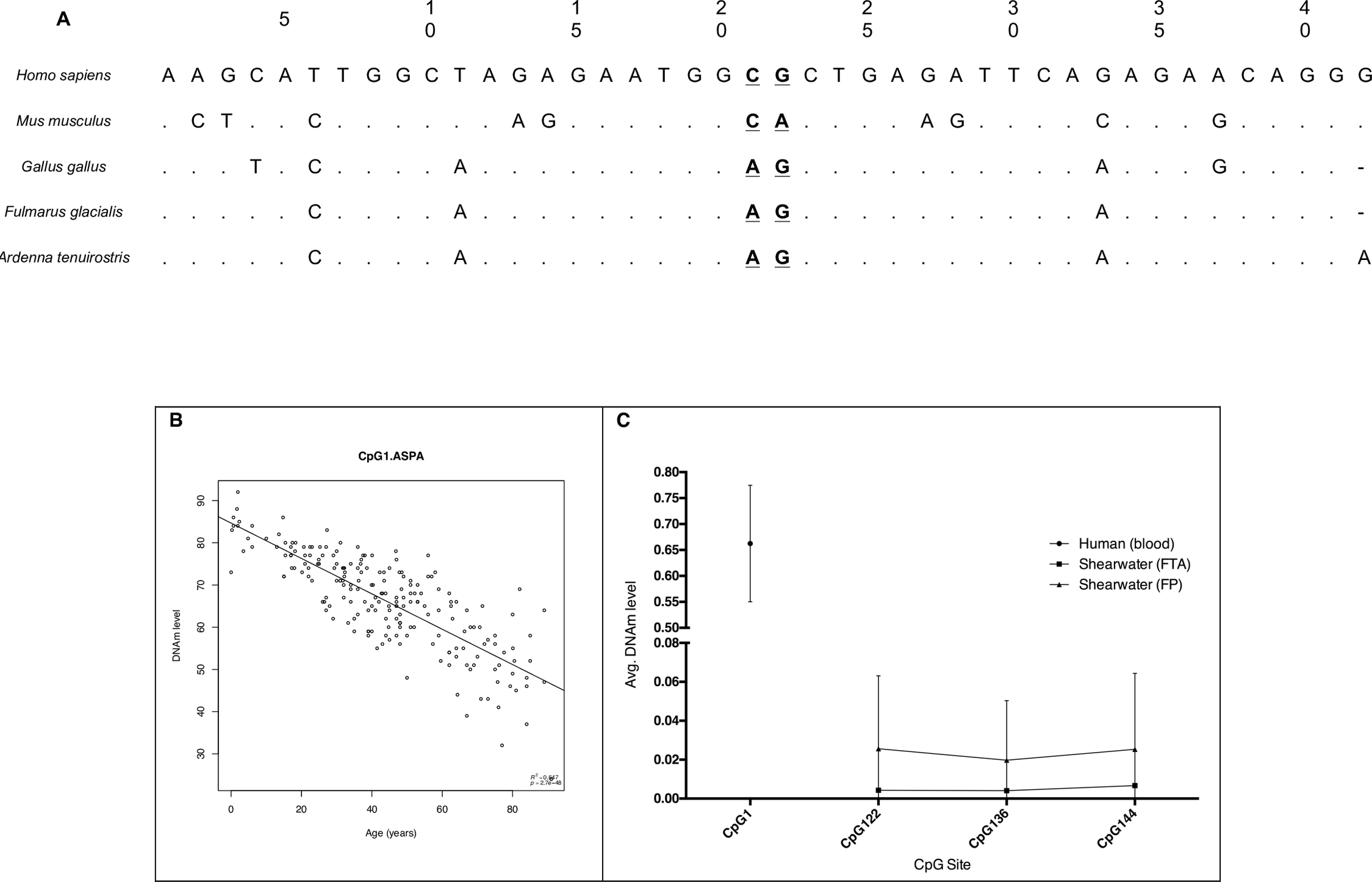

Supplement: S4 Fig — A: 20 base pairs are shown for 3’ and 5’ directions around an age-related CpG loci (27K: cg02228185, in bold) in the ASPA gene in humans, mouse and birds. Following the methods described in text, this sequence was compared to the chicken and northern fulmar genomes. The resulting matches were aligned with the human sequence, conserved bases are shown with a dot (.), missing bases with a dash (-), and the base is given where there is a mismatch. In this example, the age-related CpG site in humans was not conserved in any other species. Primers were designed from the fulmar sequence to amplify isolated shearwater DNA to examine other CpG sites in the gene. B: The correlation of ASPA DNA methylation at this site [38] compared to C: the average methylation levels observed in shearwater blood and feather. (TIF) [file pone.0189181.s004.tif]

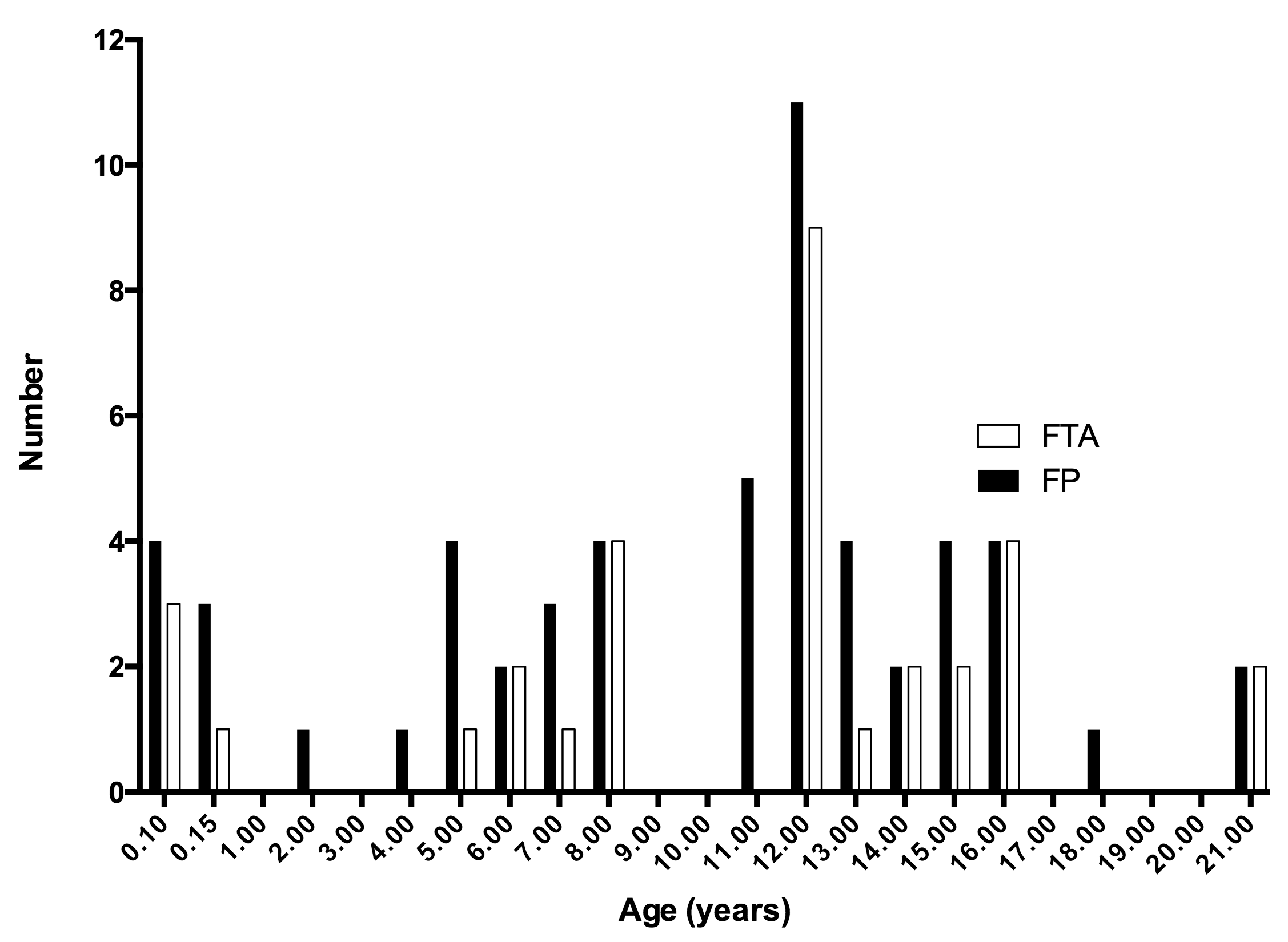

Supplement: S5 Fig — The known ages of all birds used in this study are shown split into those used for feather quill tip (FP) and whole blood (FTA) methylation analysis. (TIFF) [file pone.0189181.s005.tiff]
