# Supplementary material for: DNA methylation levels in candidate genes associated with chronological age in mammals are not conserved in a long-lived seabird
Source: PLoS One. 2017 Dec 7;12(12):e0189181. doi: 10.1371/journal.pone.0189181 (PMC5720723; doi:10.1371/journal.pone.0189181)
Supplement: S2 File — (PDF) [file pone.0189181.s007.pdf]

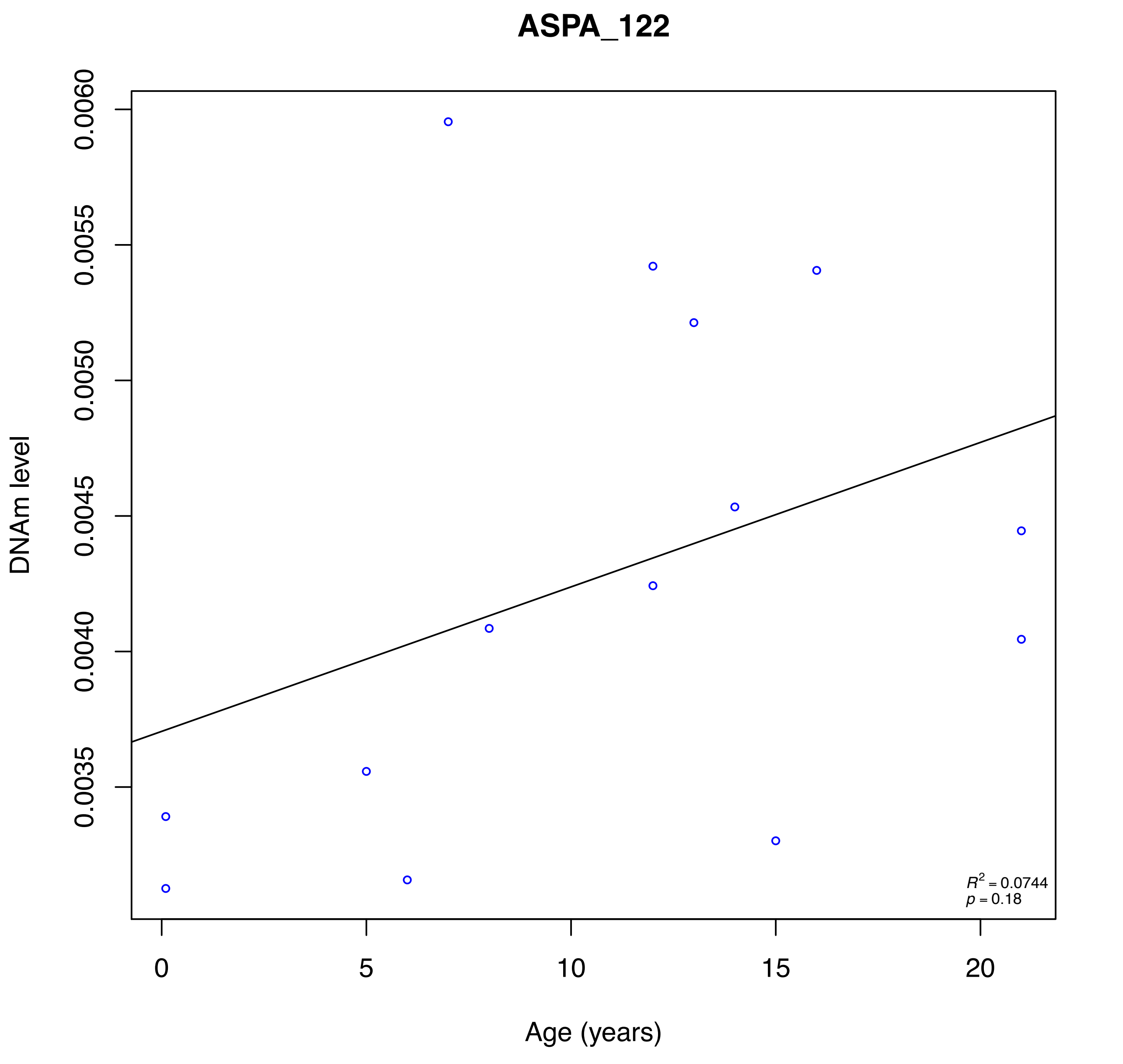
ASPA_122
DNAm level
0.0060
0.0055
0.0050
0.0045
0.0040
0.0035
Age (years)
0
5
10
15
20
R² = 0.0744
p = 0.18

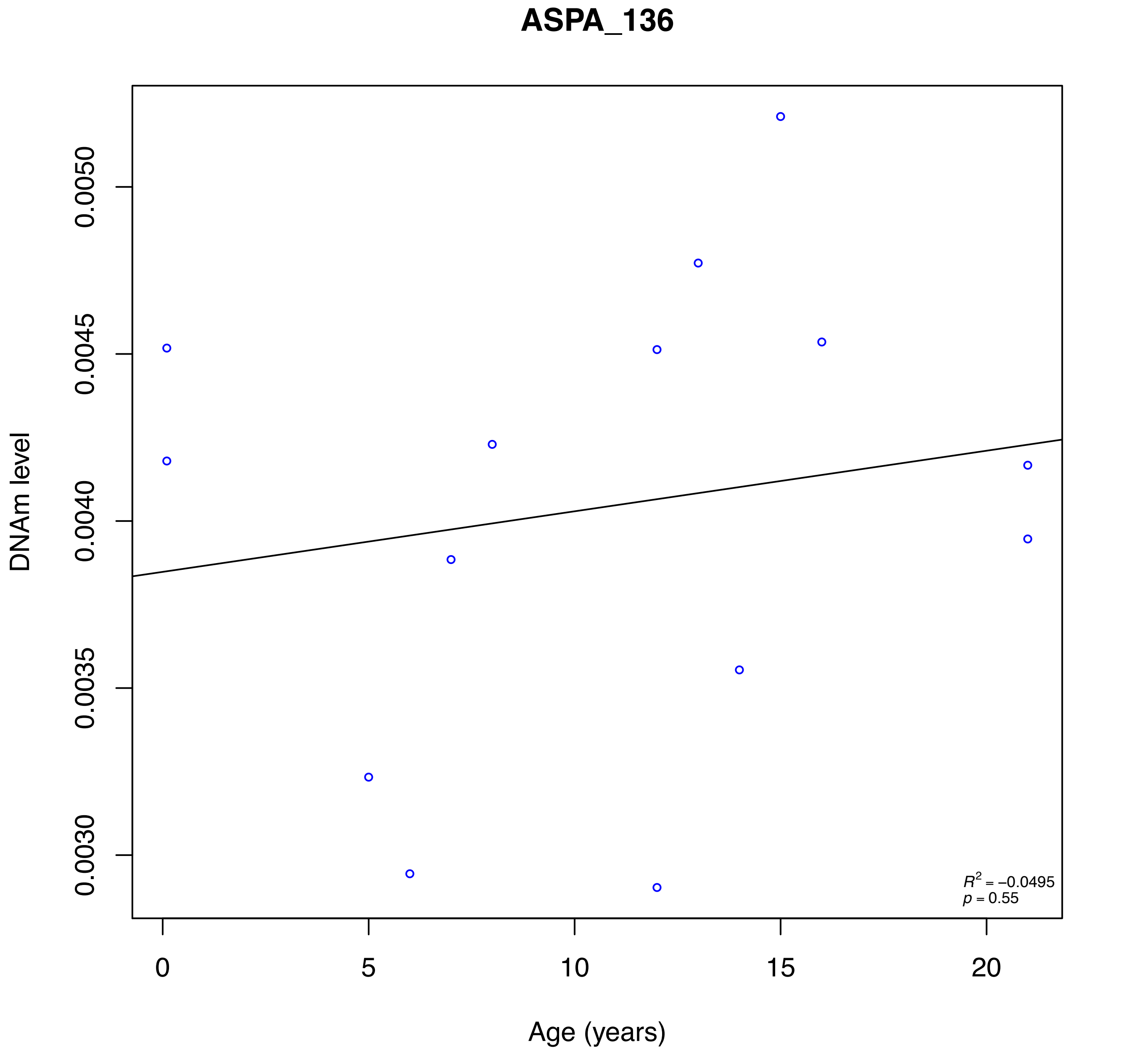

ASPA_136
DNAm level
0.0030
0.0035
0.0040
0.0045
0.0050
Age (years)
0
5
10
15
20
R^2 = −0.0495
p = 0.55

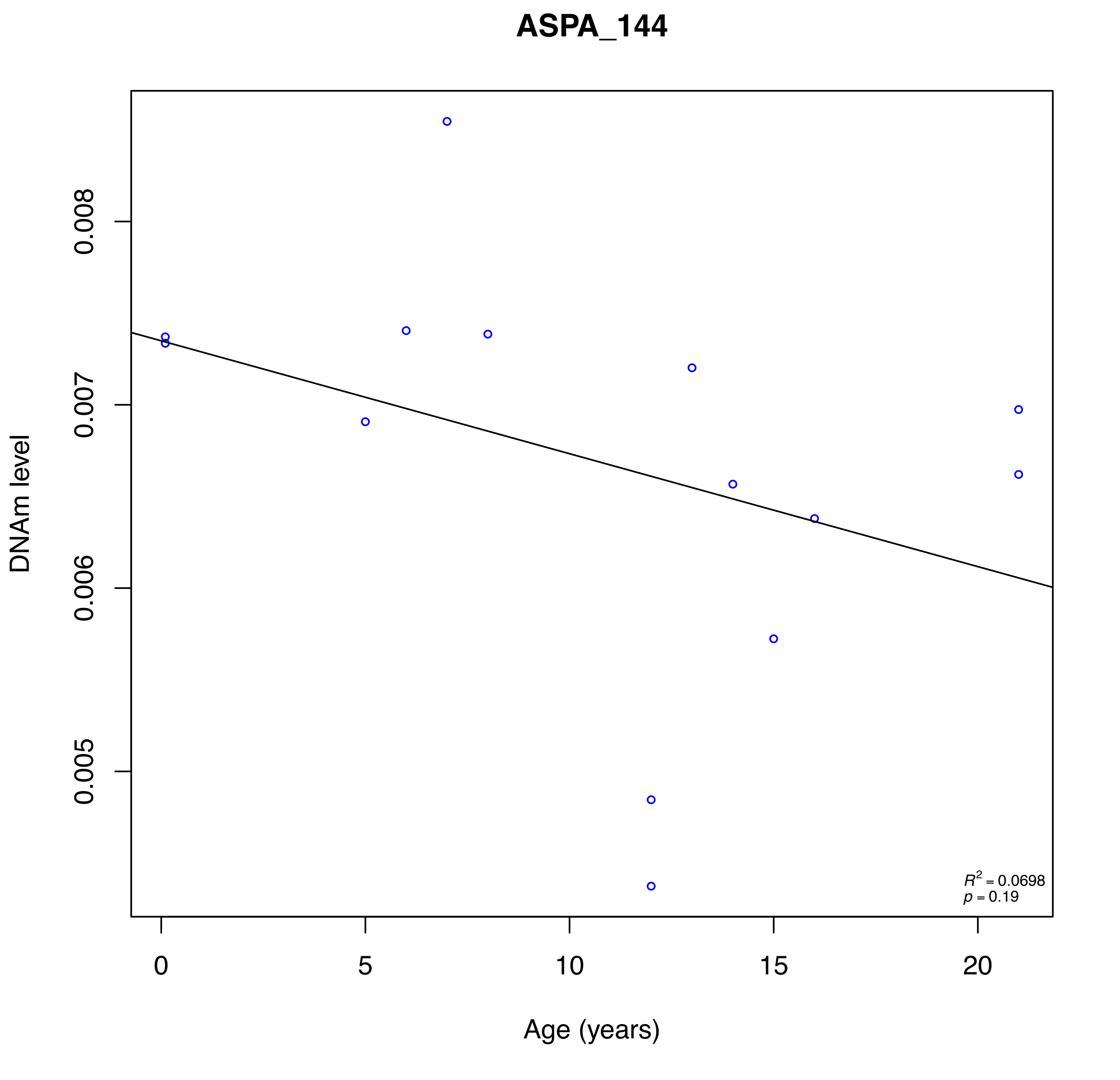
ASPA_144
DNAm level
0.005
0.006
0.007
0.008
0
5
10
15
20
Age (years)
$R^2 = 0.0698$
$p = 0.19$

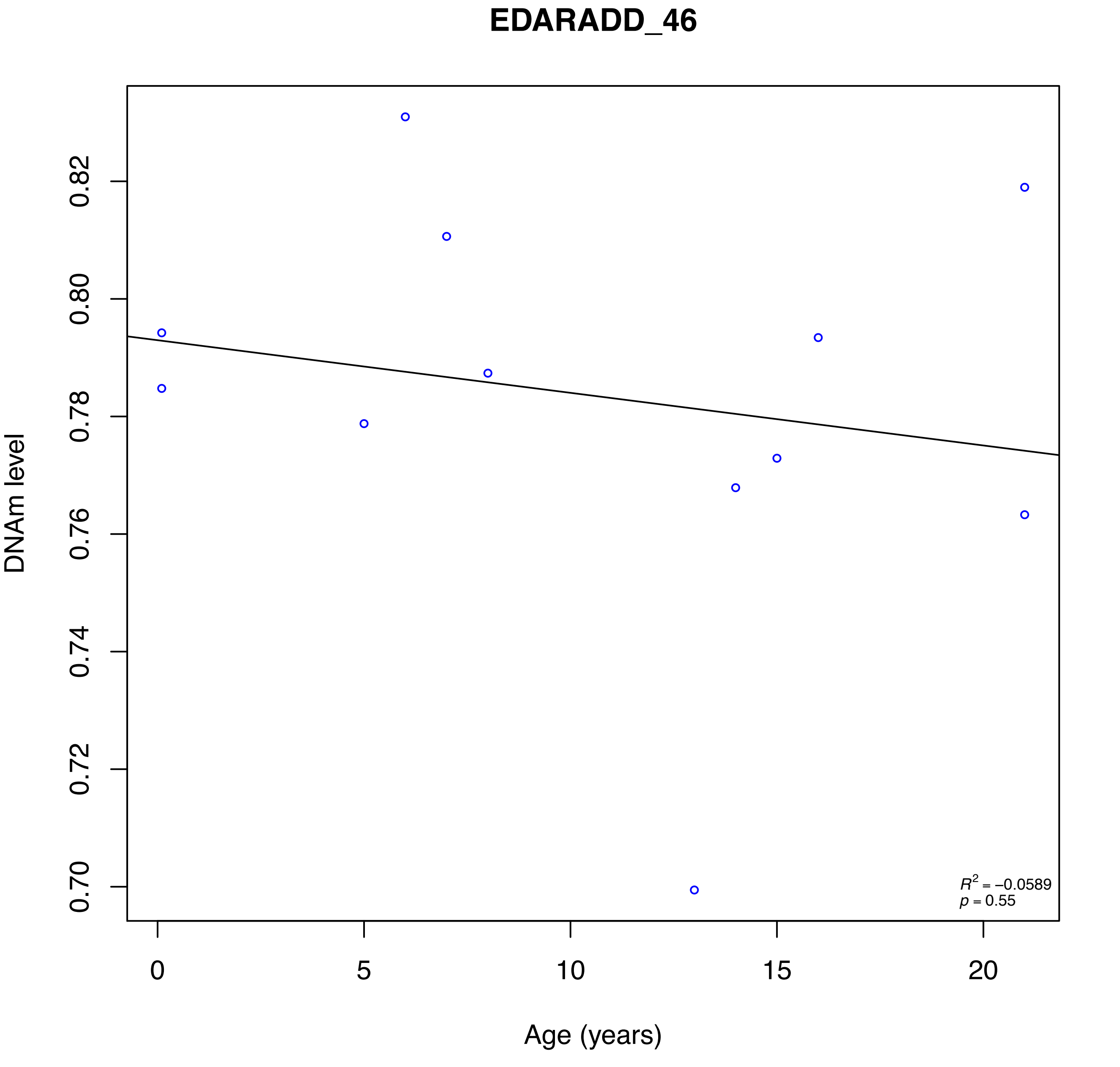

EDARADD_46
DNAm level
0.70
0.72
0.74
0.76
0.78
0.80
0.82
0
5
10
15
20
Age (years)
R^2 = −0.0589
p = 0.55

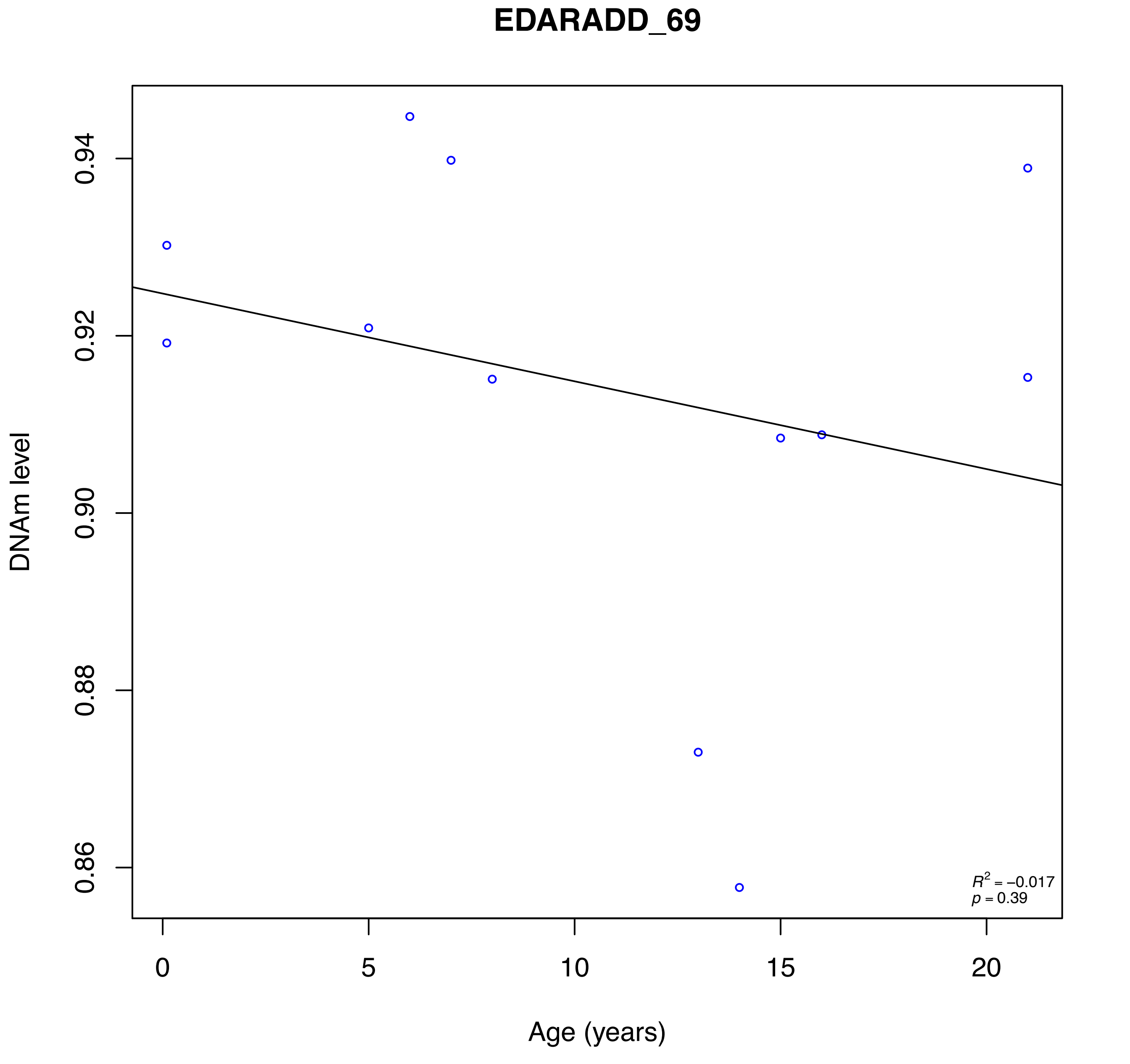

EDARADD_69
0.94
0.92
0.90
0.88
0.86
DNAm level
0
5
10
15
20
Age (years)
R^2 = −0.017
p = 0.39

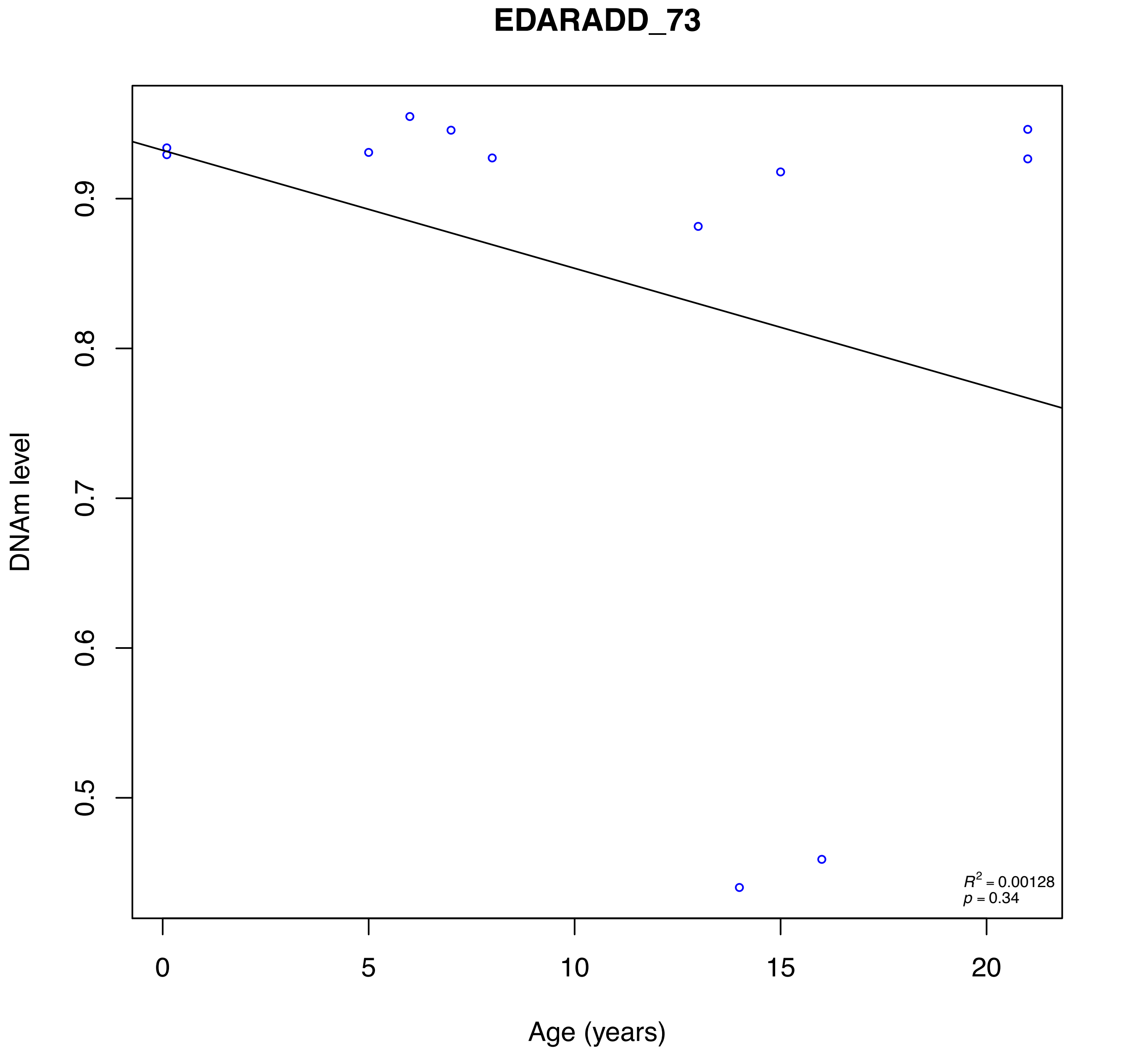
EDARADD_73
DNAm level
0.5
0.6
0.7
0.8
0.9
Age (years)
0
5
10
15
20
$R^2 = 0.00128$
$p = 0.34$

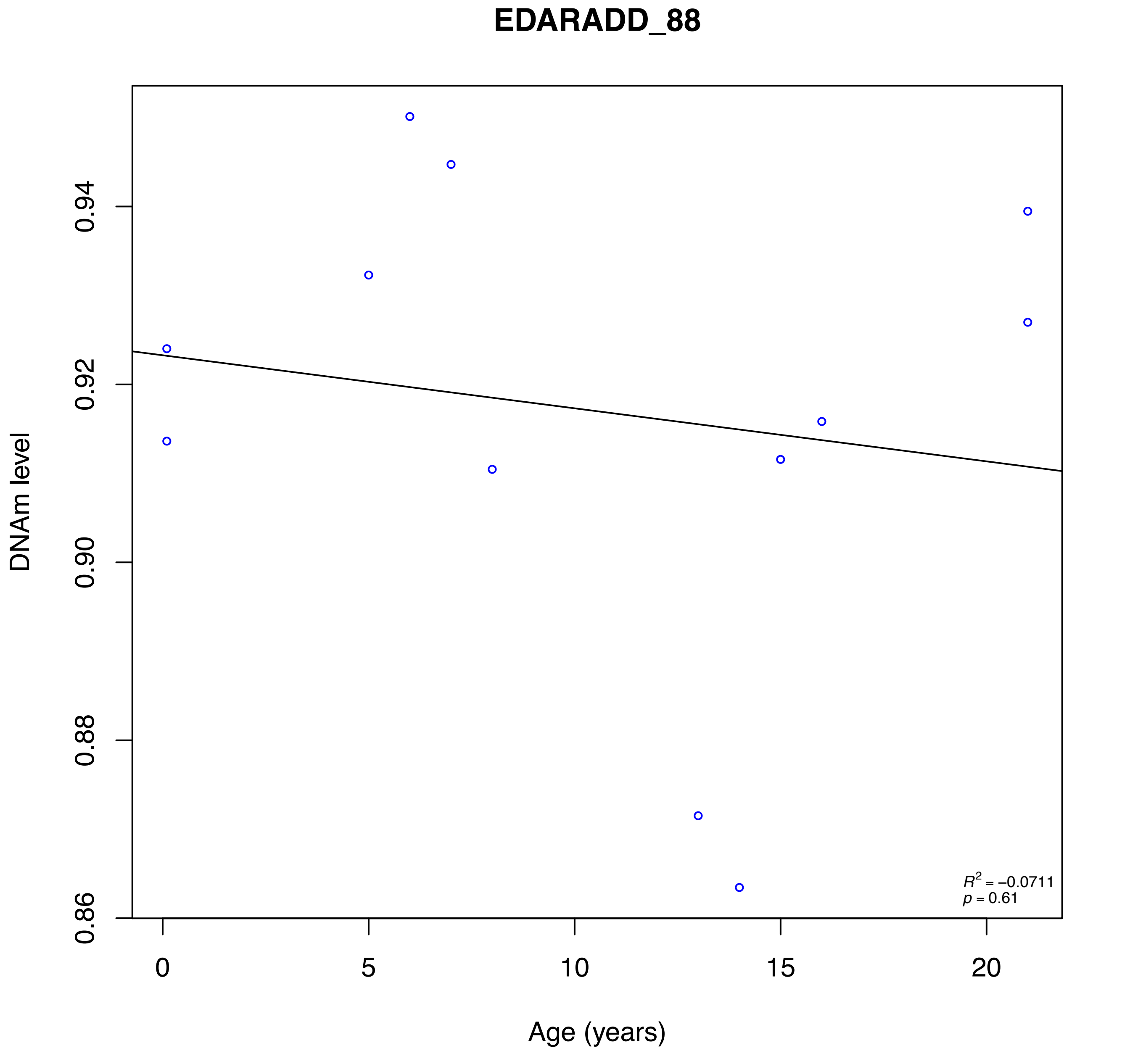
EDARADD_88
DNAm level
0.86
0.88
0.90
0.92
0.94
0
5
10
15
20
Age (years)
R² = −0.0711
p = 0.61

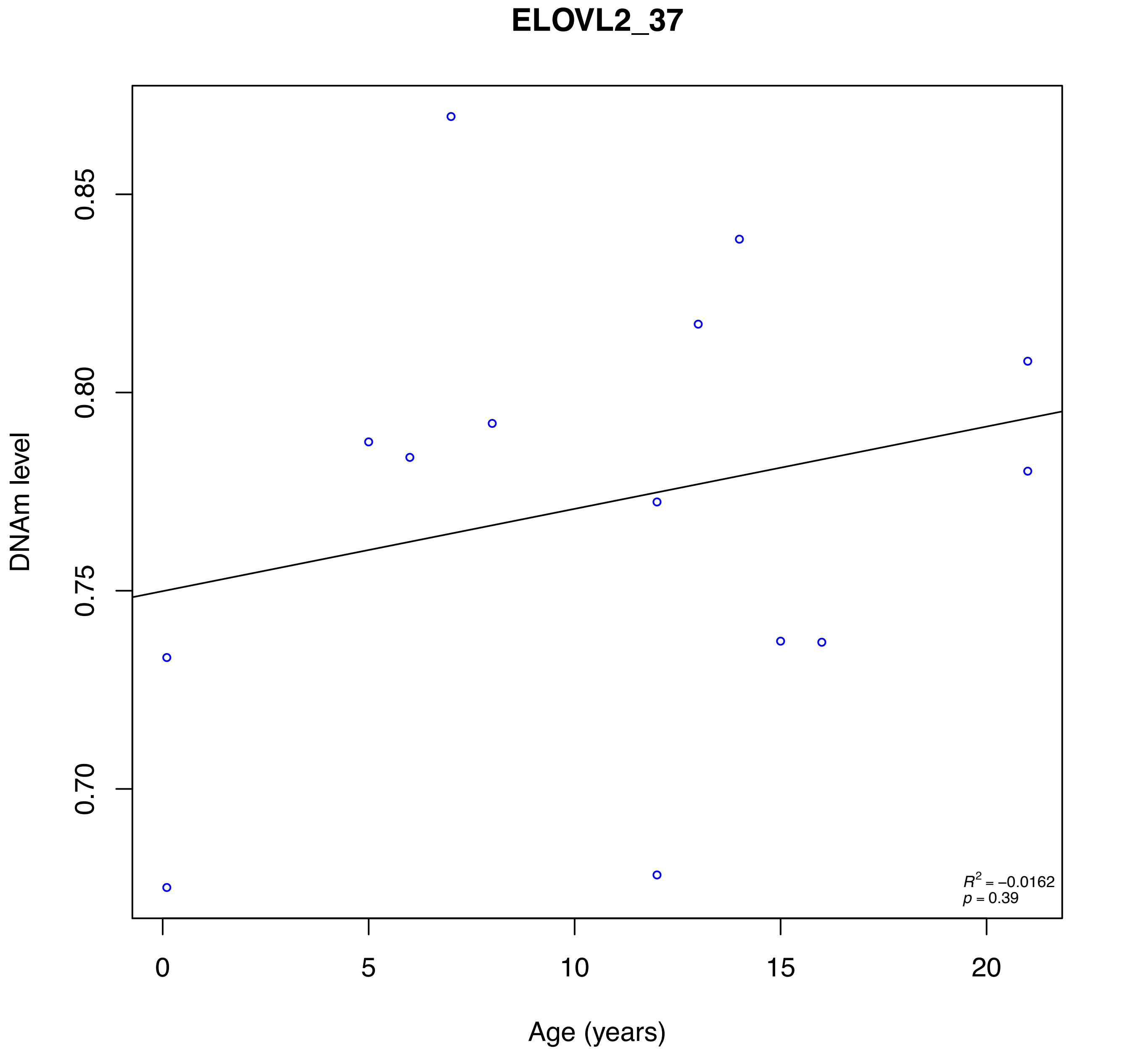

ELOVL2_37
DNAm level
0.70
0.75
0.80
0.85
0
5
10
15
20
Age (years)
R2 = −0.0162
p = 0.39

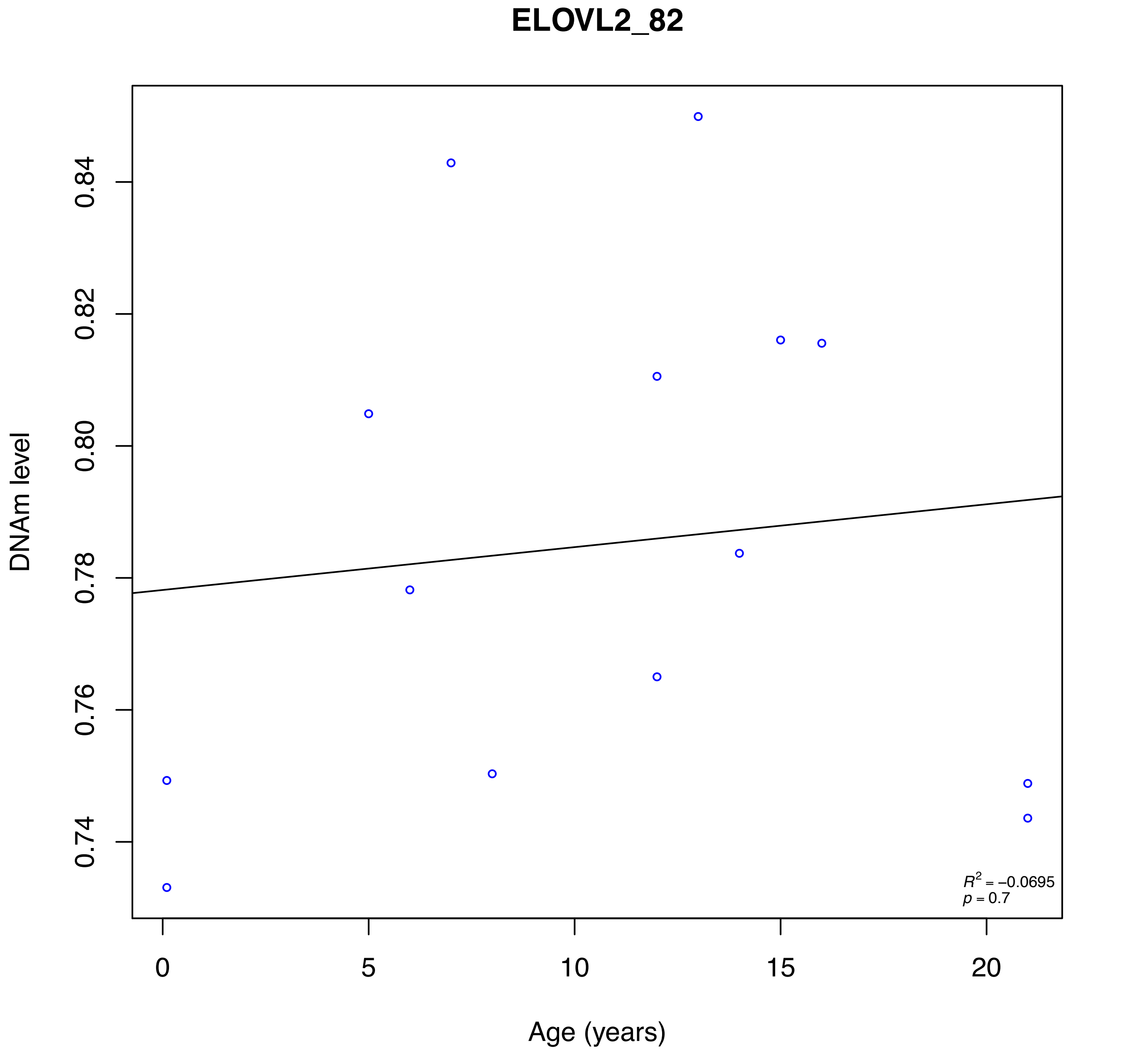
ELOVL2_82
DNAm level
0.74
0.76
0.78
0.80
0.82
0.84
0
5
10
15
20
Age (years)
R2 = −0.0695
p = 0.7

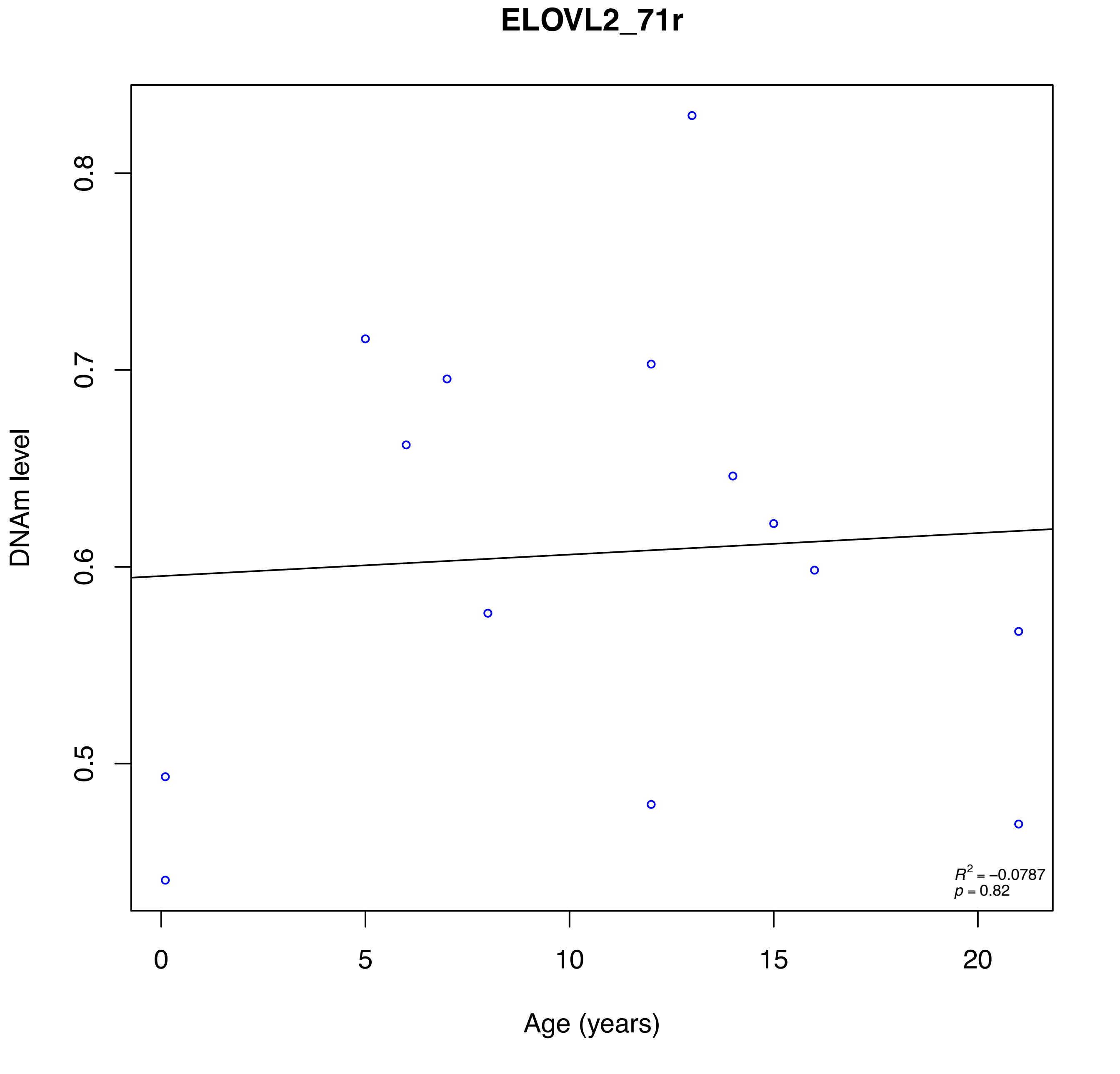
ELOVL2_71r
DNAm level
Age (years)
0.5
0.6
0.7
0.8
0
5
10
15
20
R^2 = −0.0787
p = 0.82

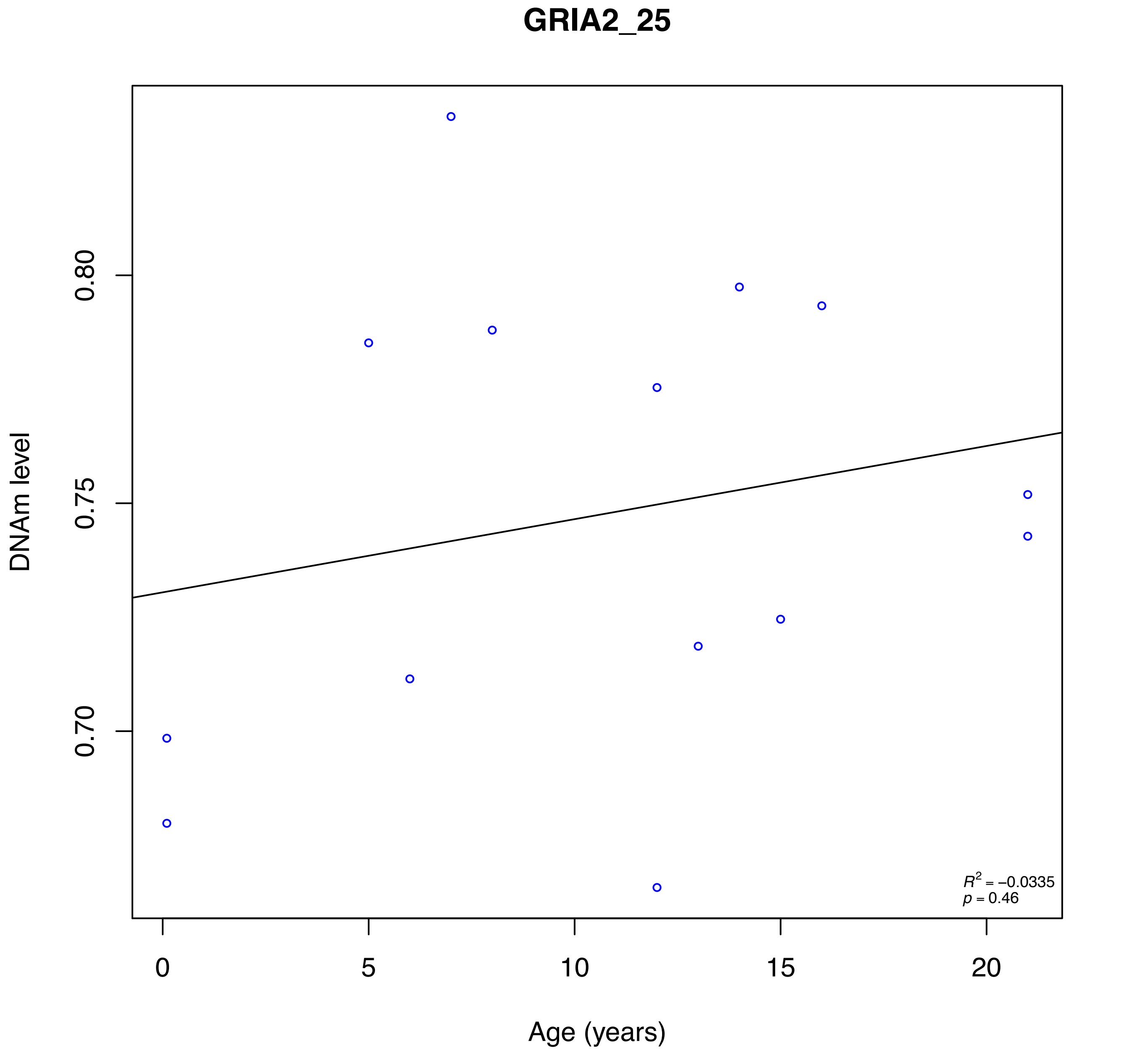

GRIA2_25
0.80
0.75
0.70
DNAm level
0
5
10
15
20
Age (years)
R^2 = −0.0335
p = 0.46

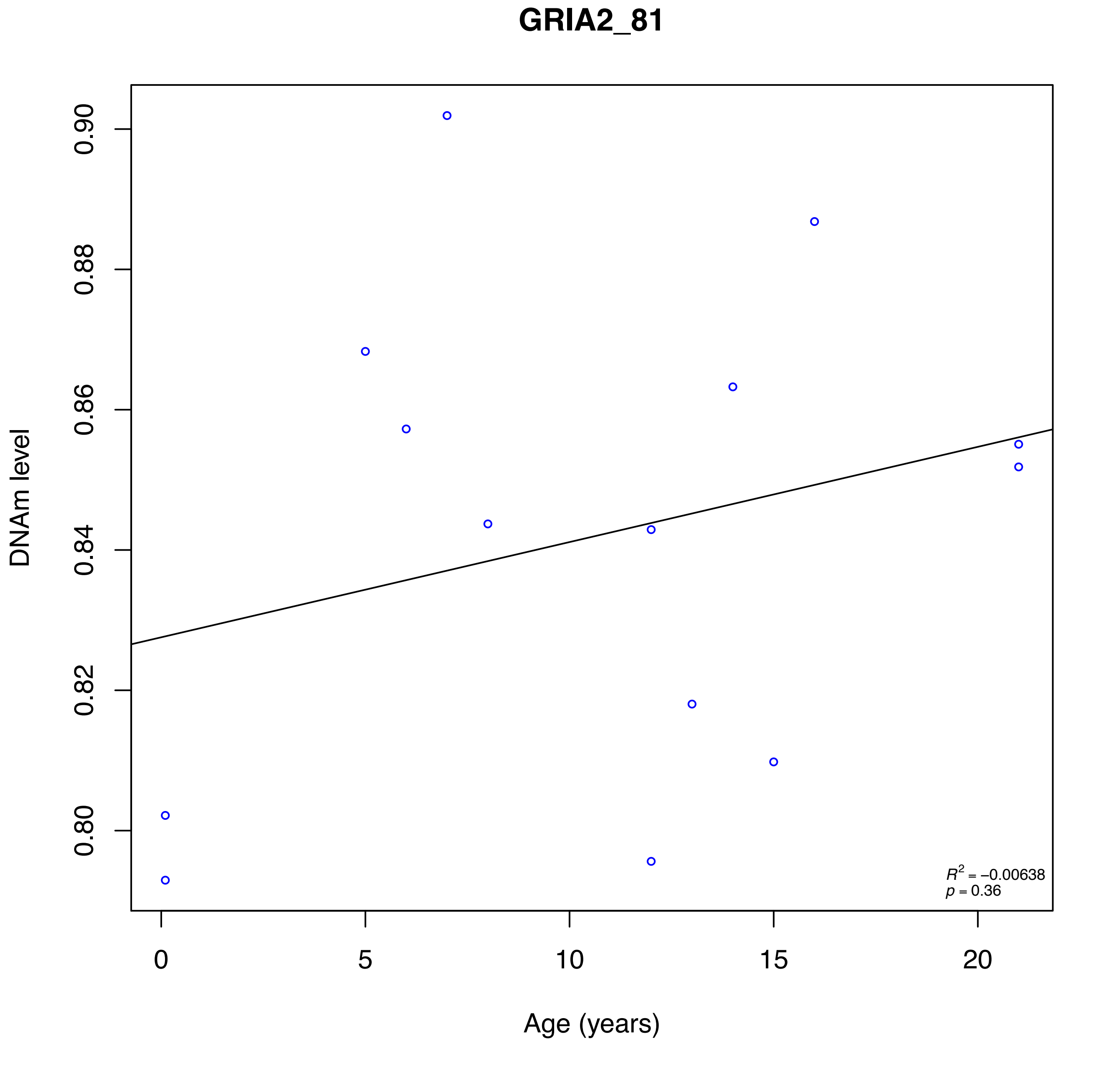
GRIA2_81
DNAm level
0.80
0.82
0.84
0.86
0.88
0.90
Age (years)
0
5
10
15
20
R2 = −0.00638
p = 0.36

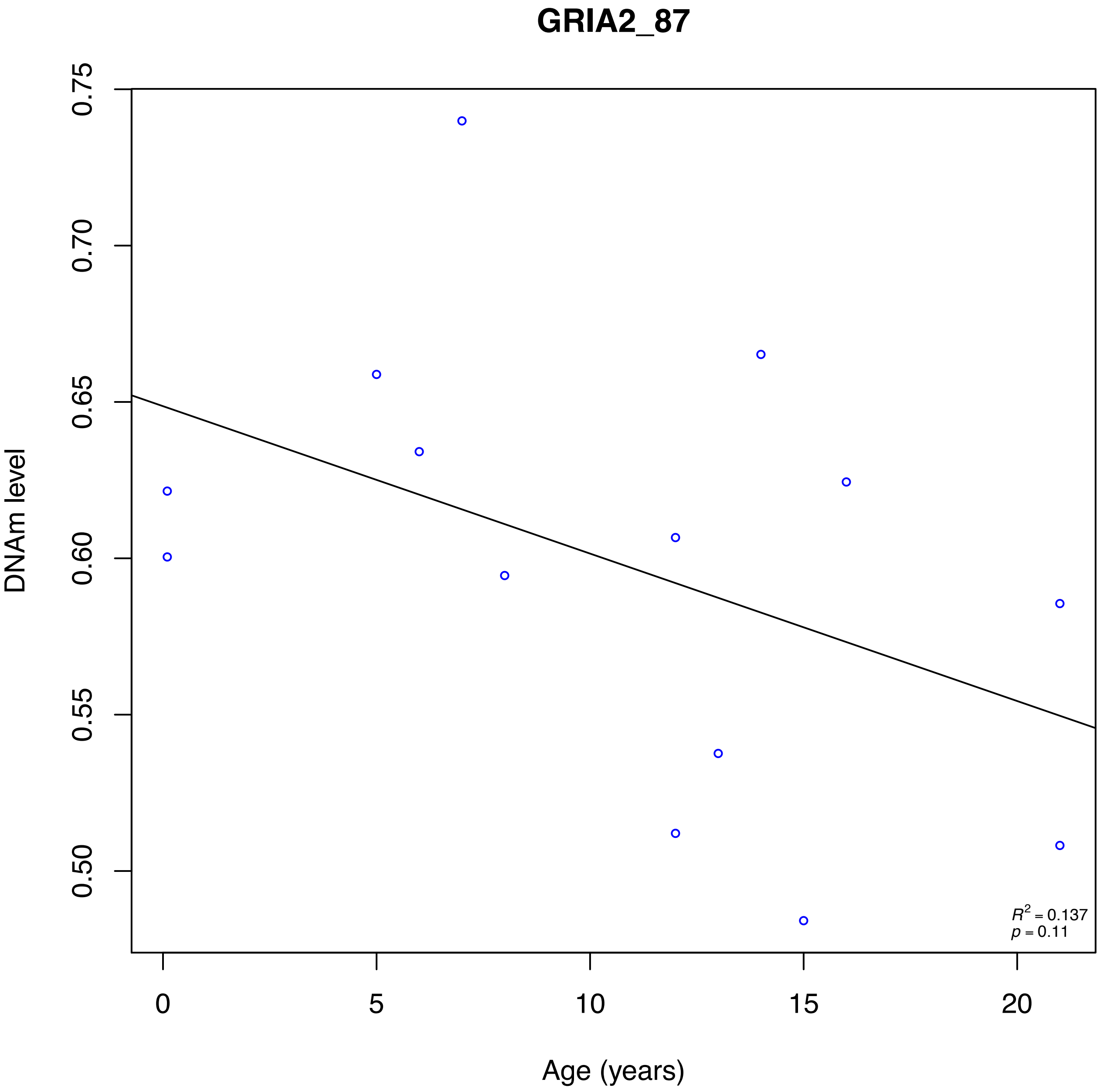
GRIA2_87
DNAm level
0.50
0.55
0.60
0.65
0.70
0.75
0
5
10
15
20
Age (years)
$R^2 = 0.137$
$p = 0.11$

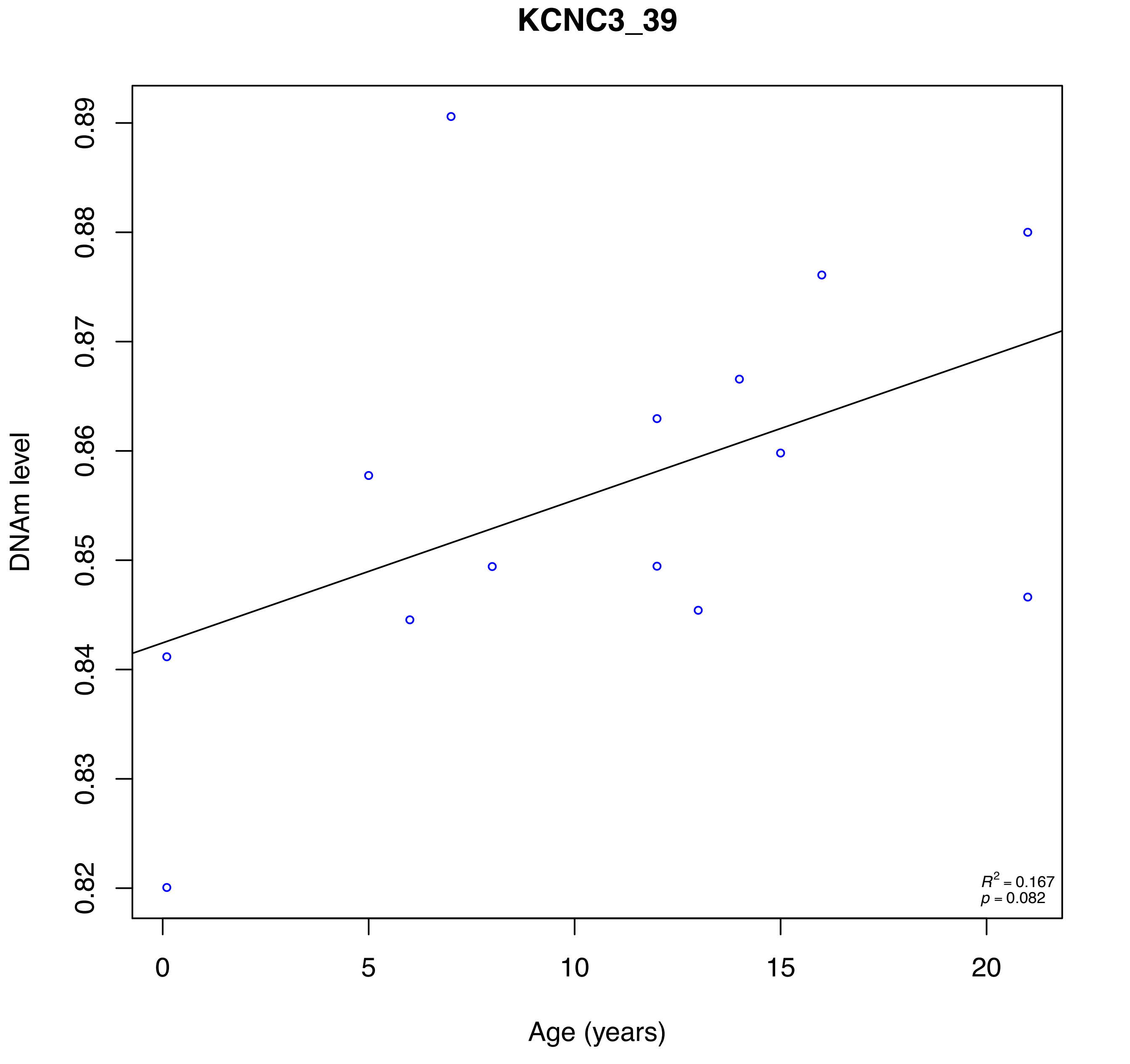
KCNC3_39
DNAm level
0.82
0.83
0.84
0.85
0.86
0.87
0.88
0.89
Age (years)
0
5
10
15
20
R^2 = 0.167
p = 0.082

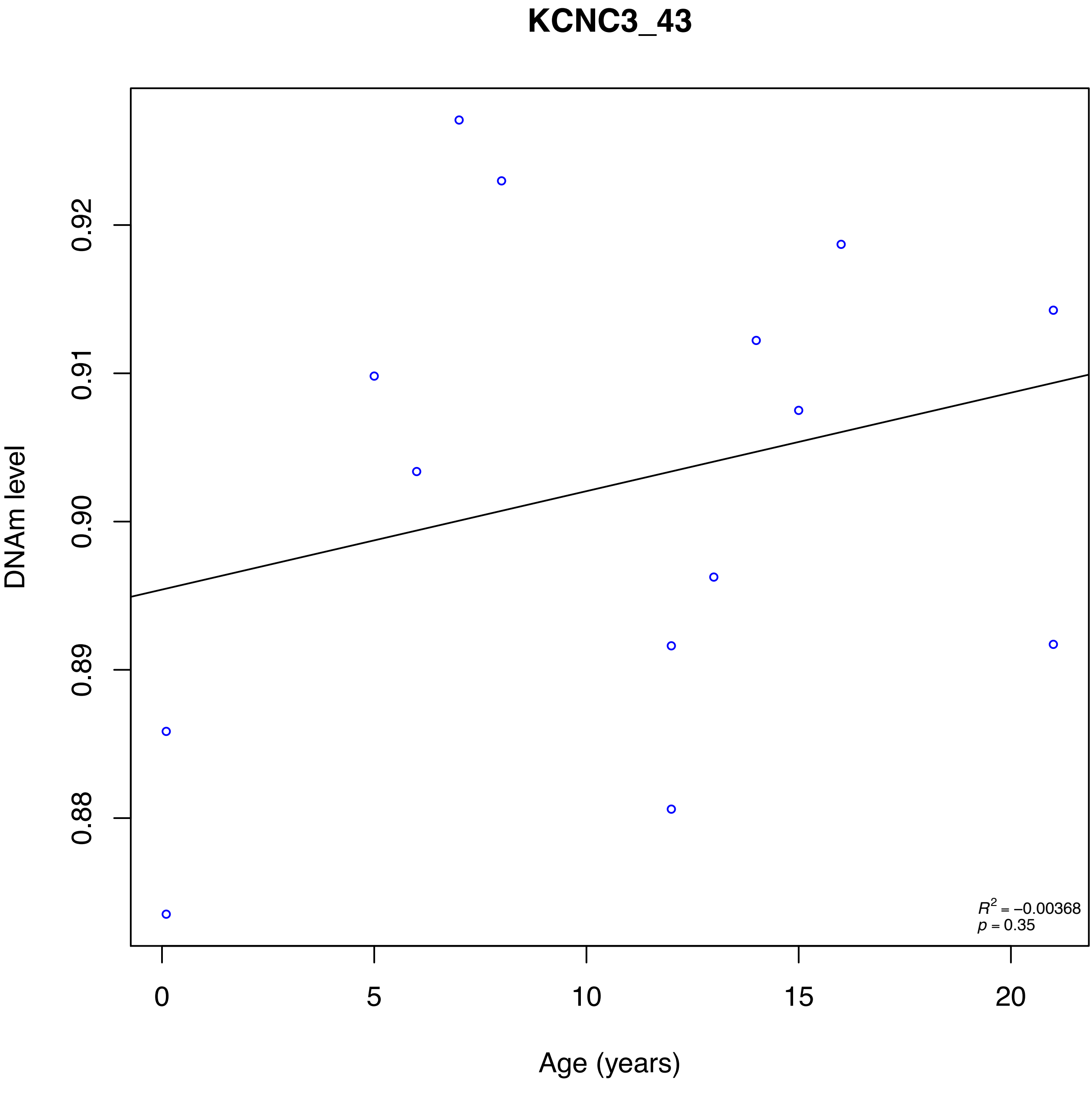
KCNC3_43
DNAm level
0.88
0.89
0.90
0.91
0.92
0
5
10
15
20
Age (years)
R^2 = −0.00368
p = 0.35

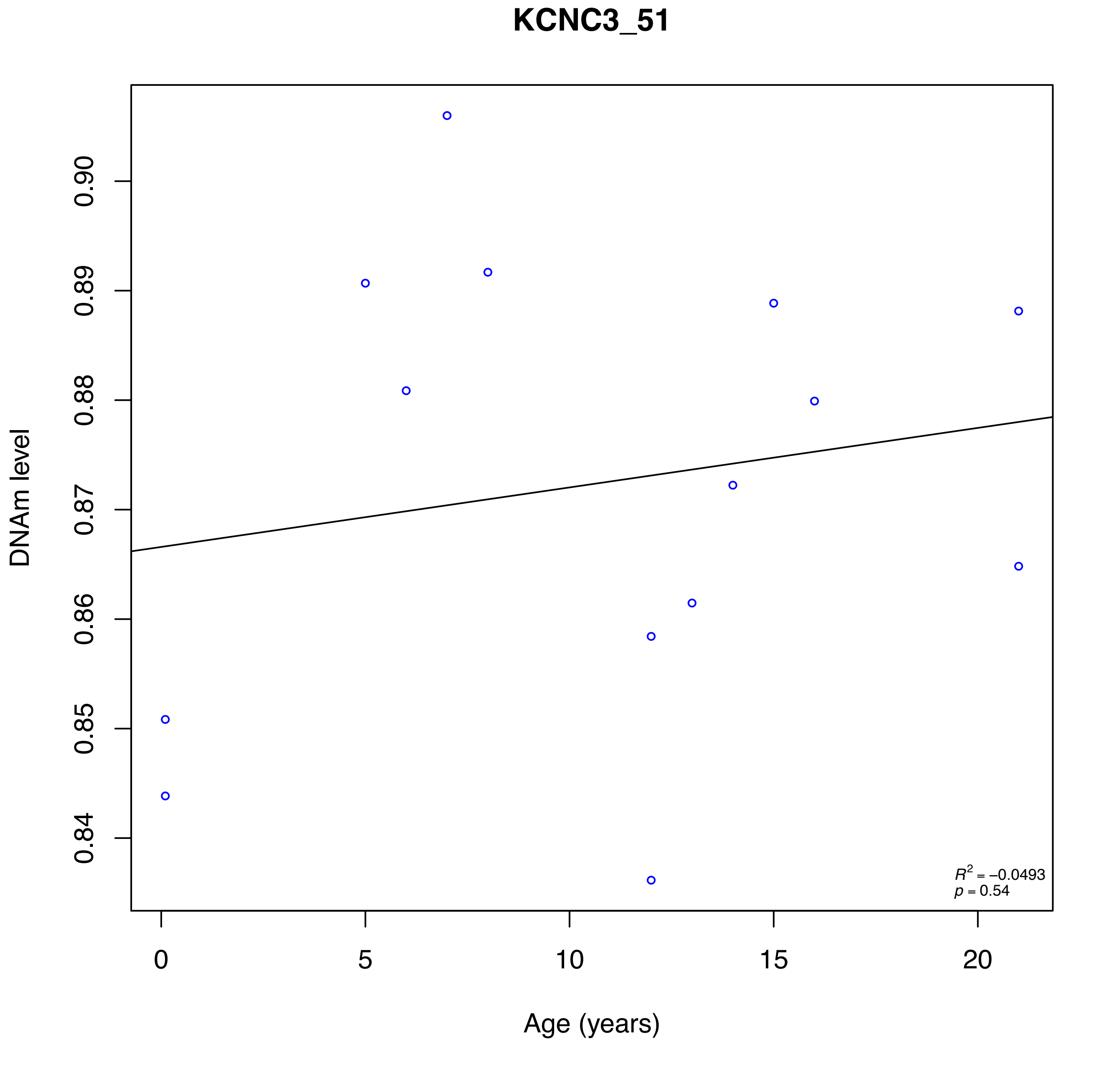
KCNC3_51
DNAm level
0.84
0.85
0.86
0.87
0.88
0.89
0.90
Age (years)
0
5
10
15
20
R^2 = −0.0493
p = 0.54

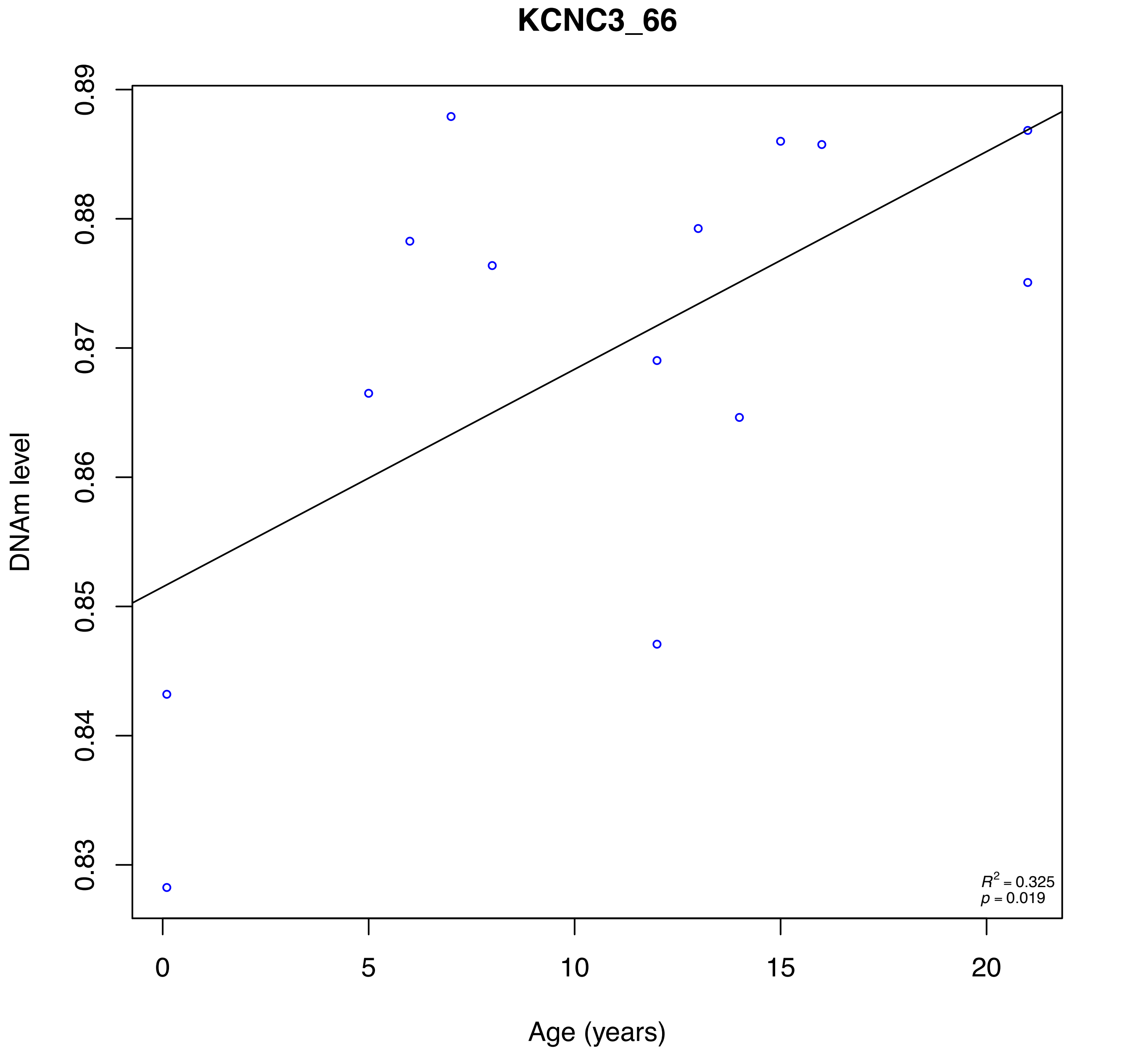
KCNC3_66
0.89
0.88
0.87
0.86
0.85
0.84
0.83
DNAm level
0
5
10
15
20
Age (years)
R^2 = 0.325
p = 0.019

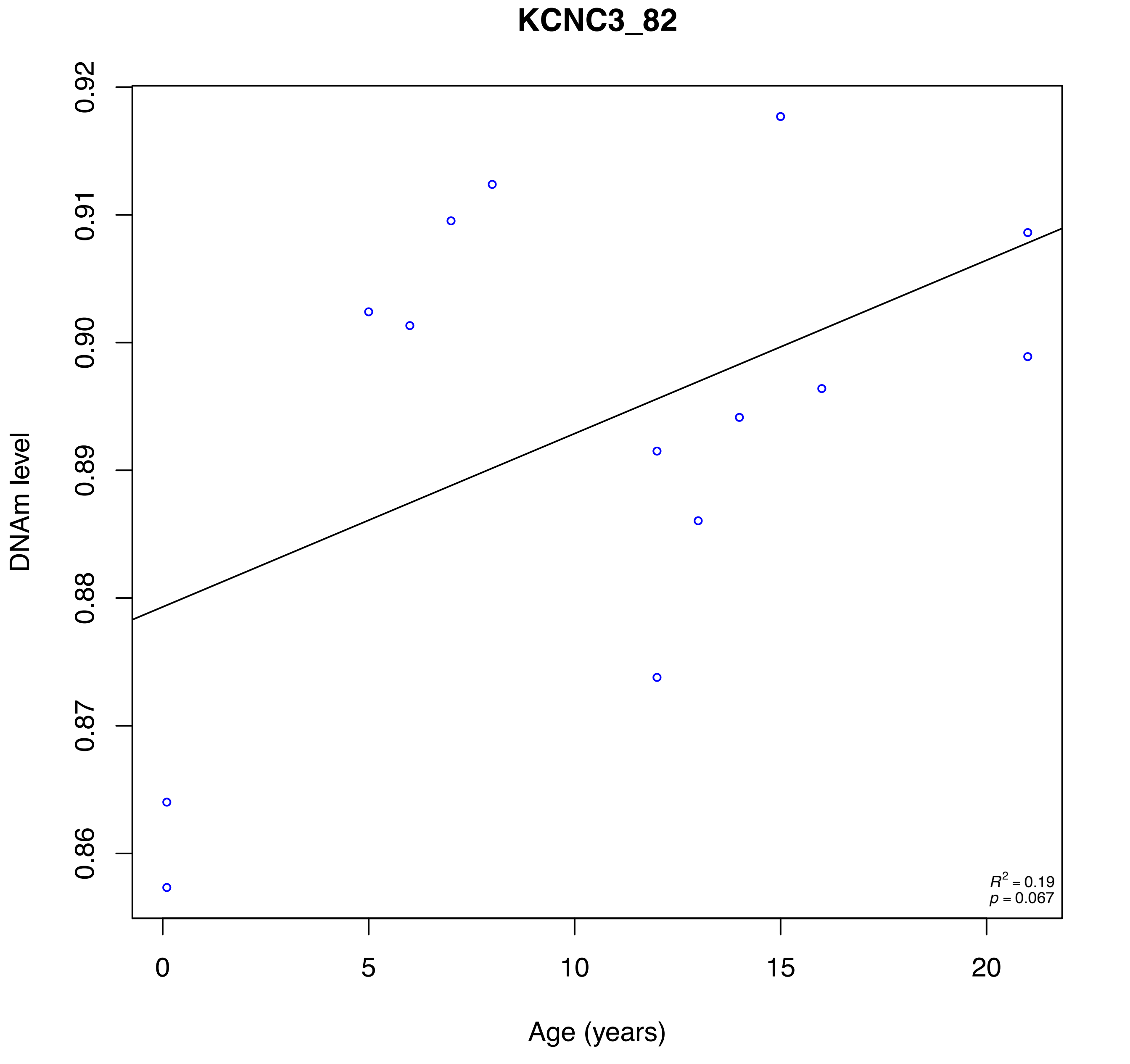
KCNC3_82
DNAm level
0.86
0.87
0.88
0.89
0.90
0.91
0.92
Age (years)
0
5
10
15
20
R^2 = 0.19
p = 0.067

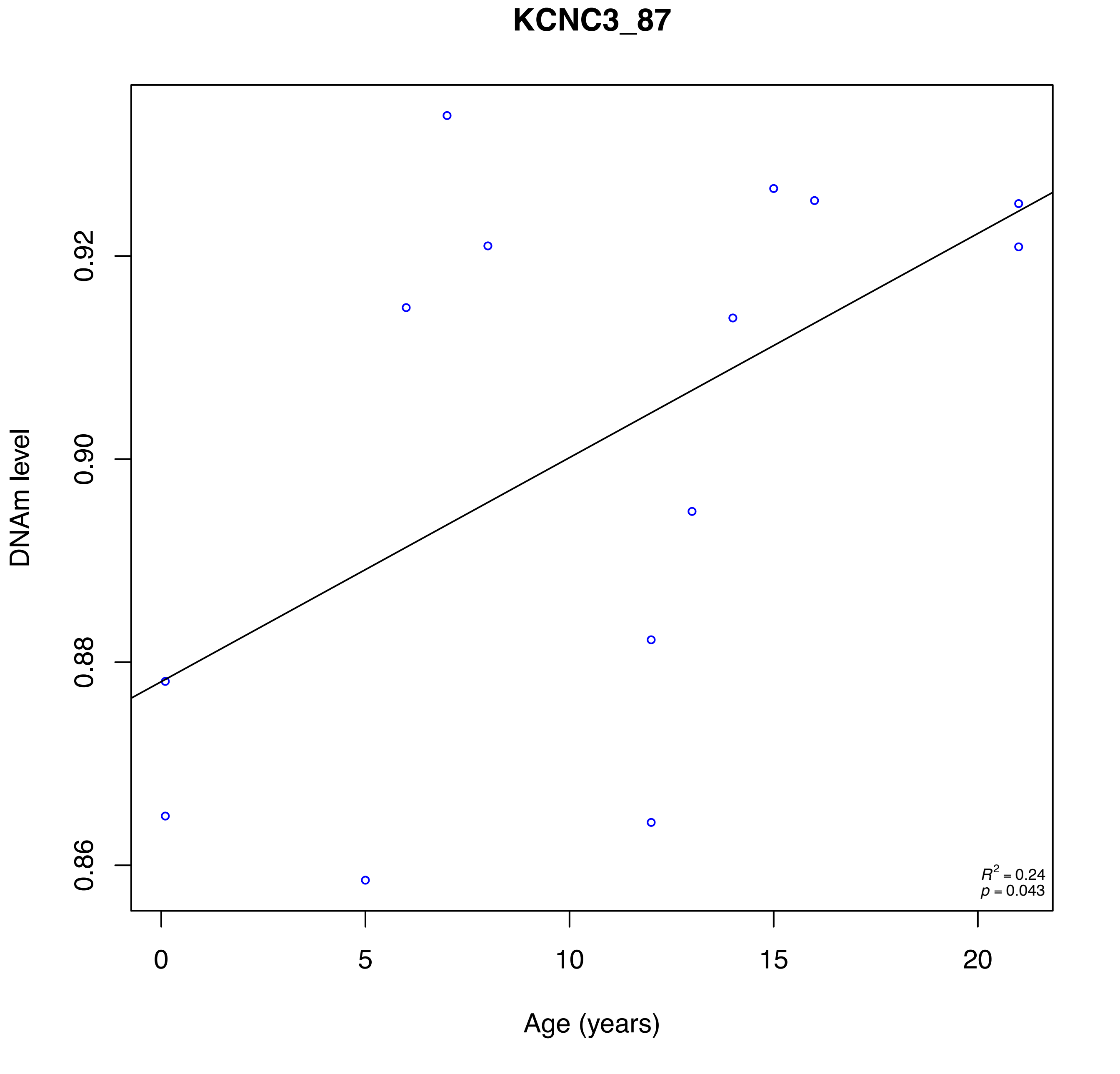
KCNC3_87
DNAm level
0.86
0.88
0.90
0.92
0
5
10
15
20
Age (years)
$R^2 = 0.24$
$p = 0.043$

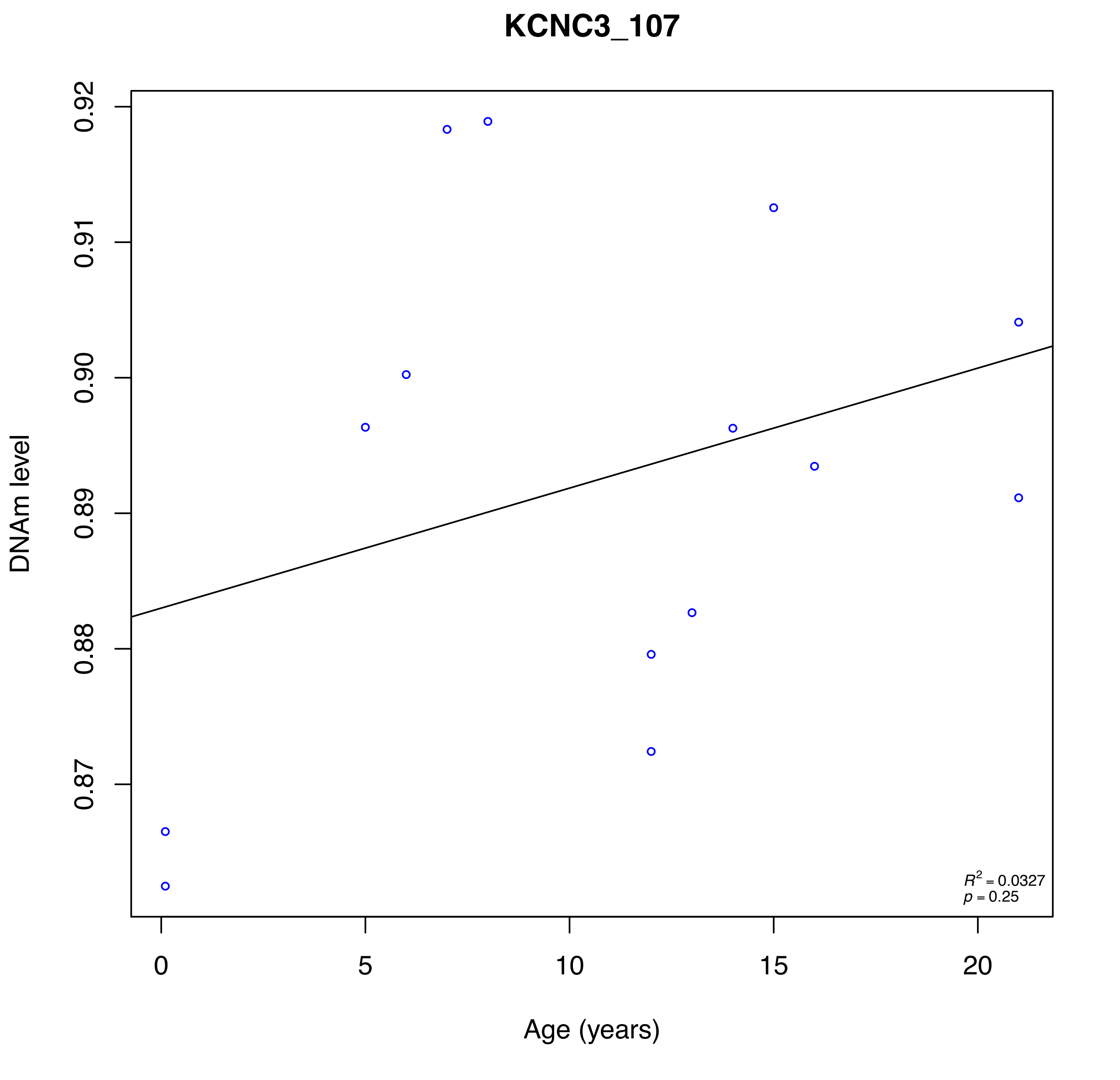
KCNC3_107
DNAm level
Age (years)
0.87
0.88
0.89
0.90
0.91
0.92
0
5
10
15
20
$R^2 = 0.0327$
$p = 0.25$

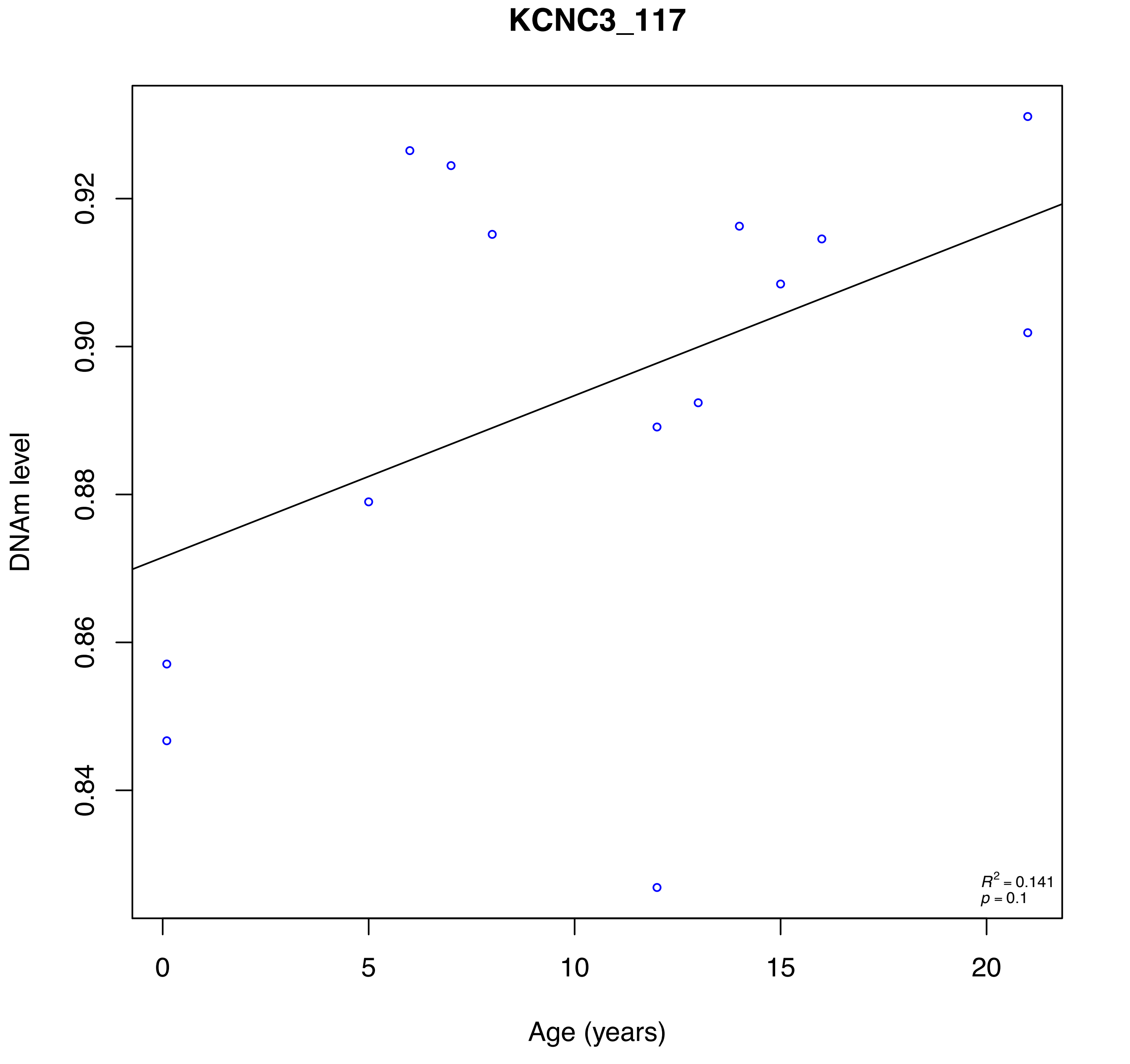
KCNC3_117
0.84
0.86
0.88
0.90
0.92
DNAm level
0
5
10
15
20
Age (years)
R^2 = 0.141
p = 0.1

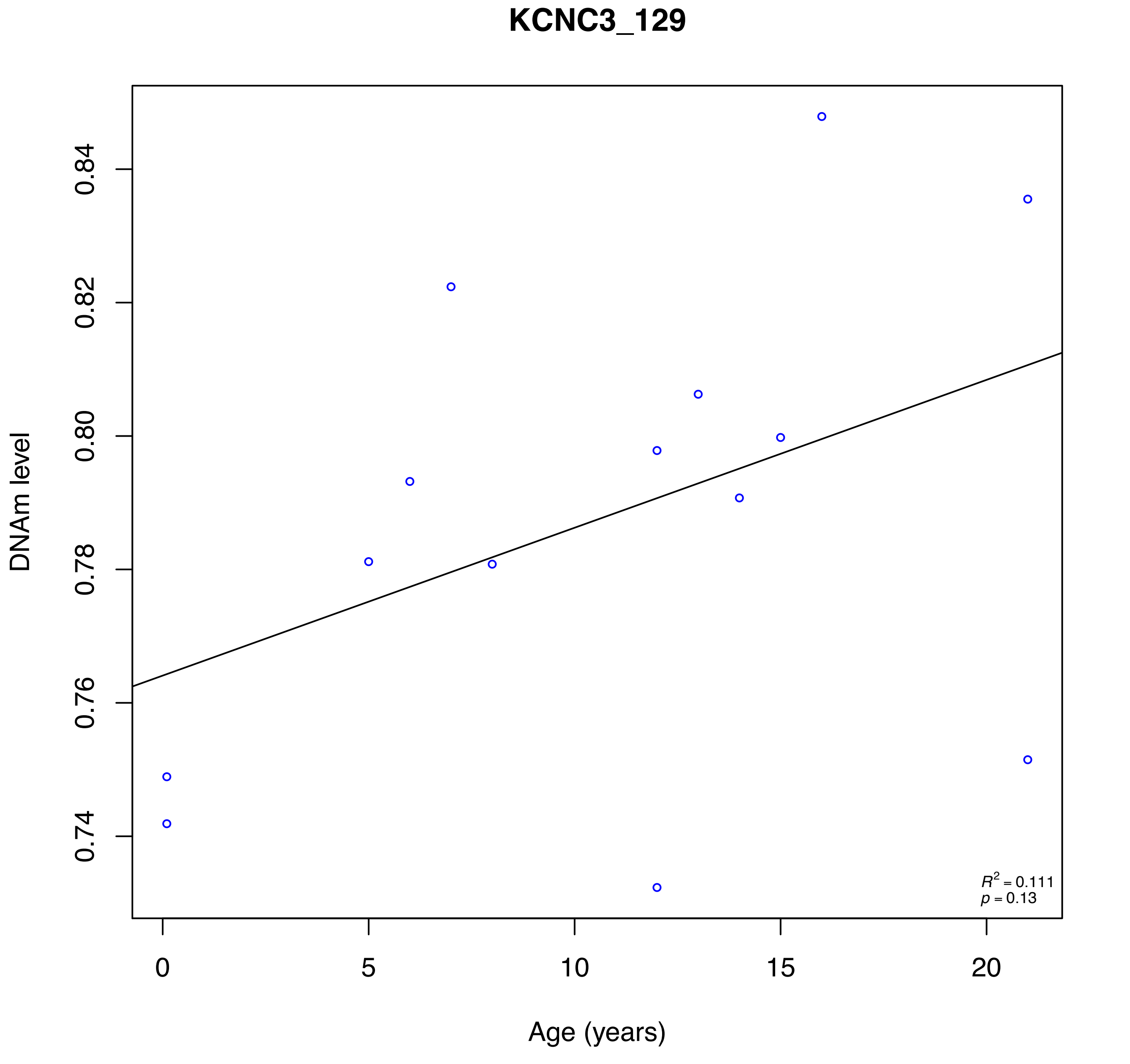

KCNC3_129
DNAm level
0.74
0.76
0.78
0.80
0.82
0.84
Age (years)
0
5
10
15
20
R2 = 0.111
p = 0.13

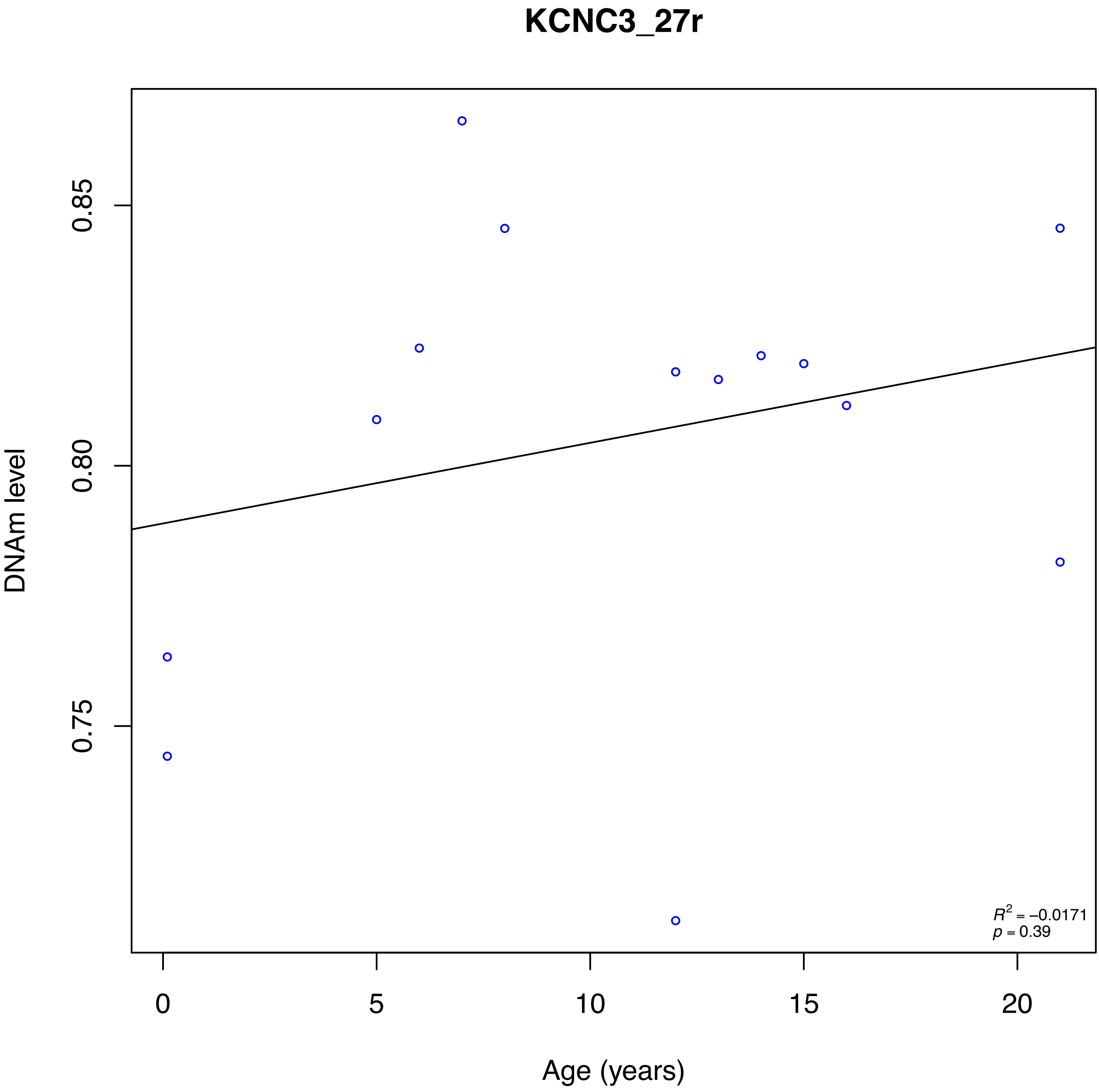
KCNC3_27r
DNAm level
0.75
0.80
0.85
Age (years)
0
5
10
15
20
R^2 = −0.0171
p = 0.39

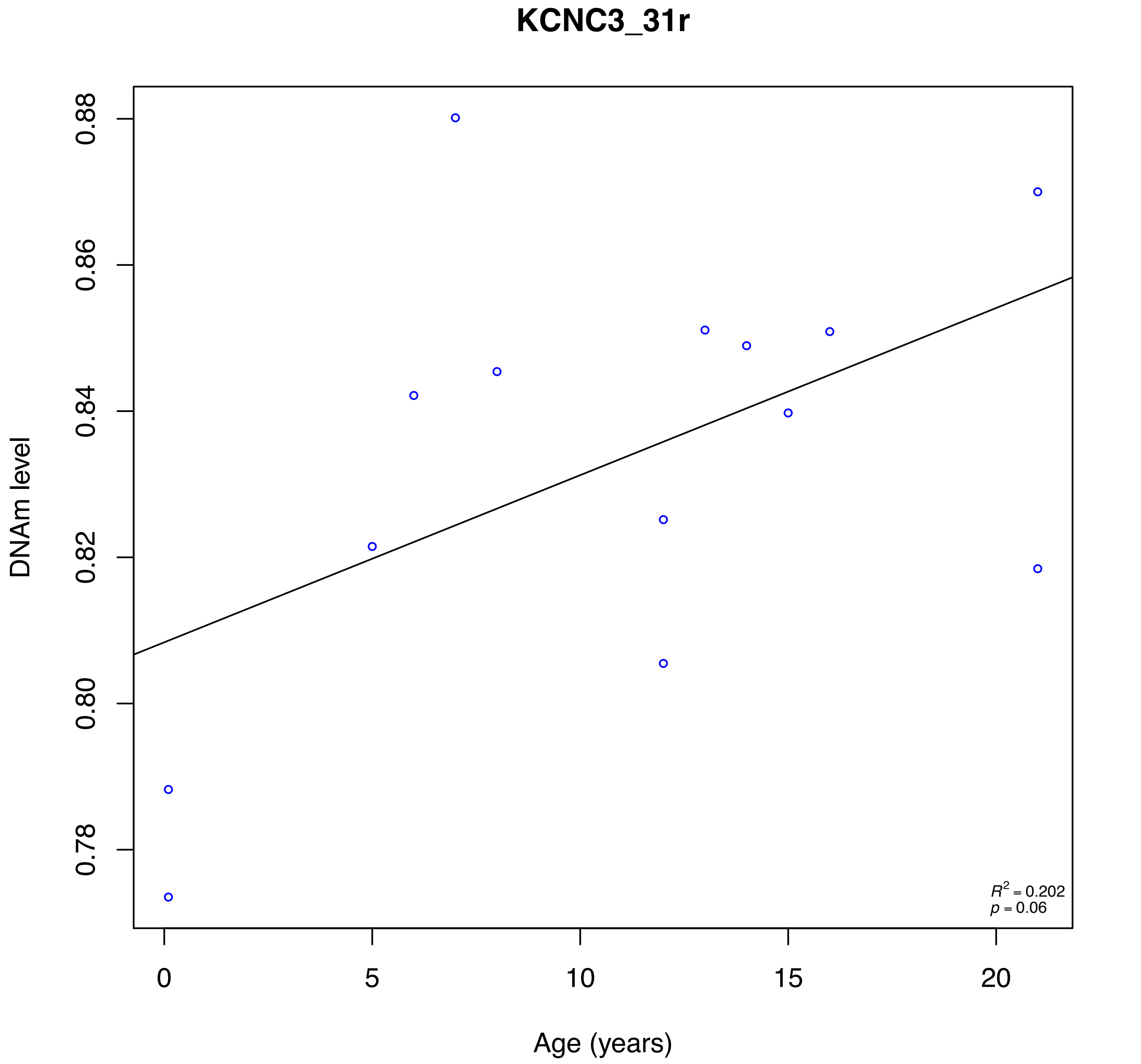
KCNC3_31r
DNAm level
0.78
0.80
0.82
0.84
0.86
0.88
Age (years)
0
5
10
15
20
R^2 = 0.202
p = 0.06

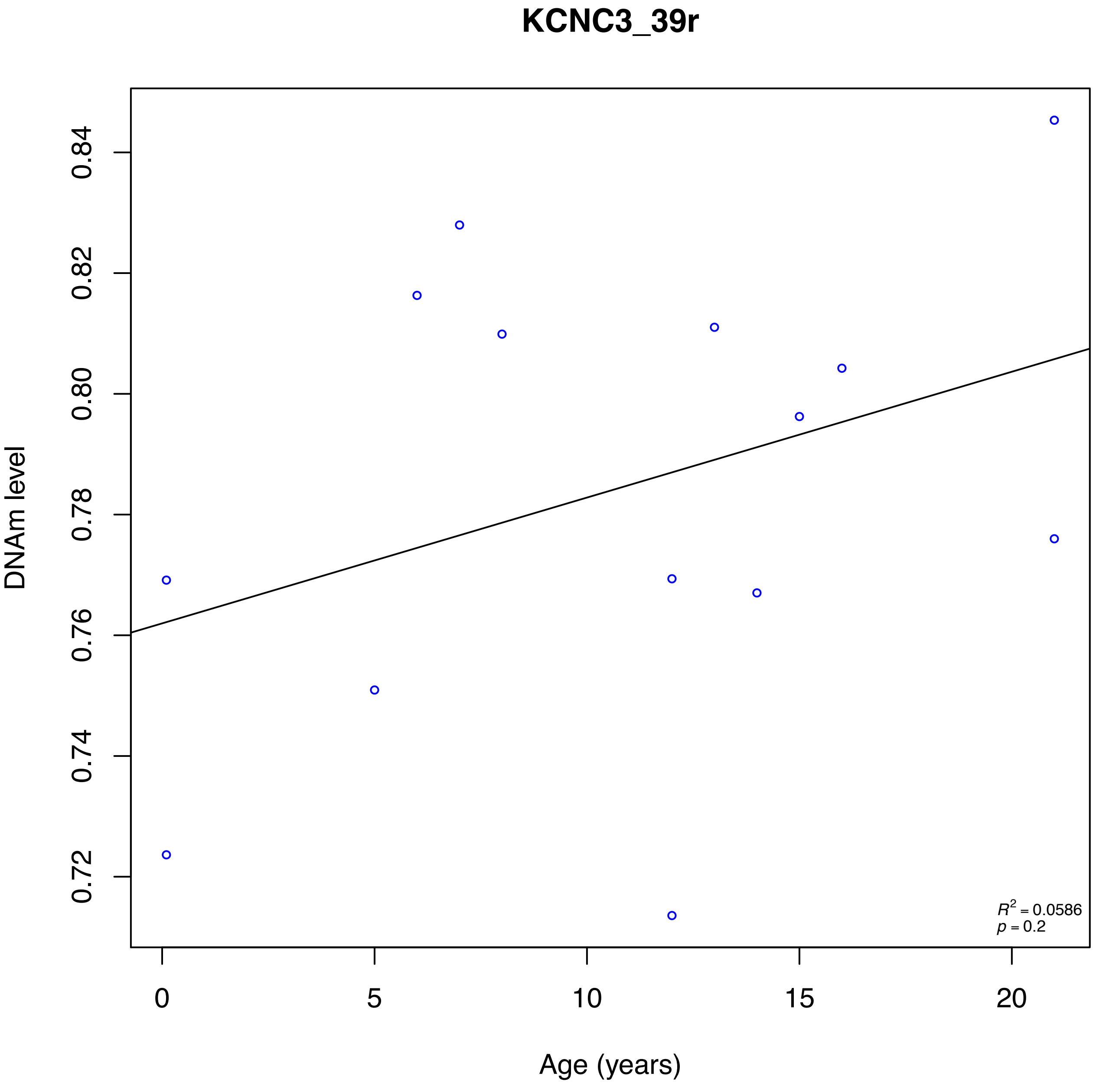

KCNC3_39r
DNAm level
Age (years)
0.72
0.74
0.76
0.78
0.80
0.82
0.84
0
5
10
15
20
$R^2 = 0.0586$
$p = 0.2$

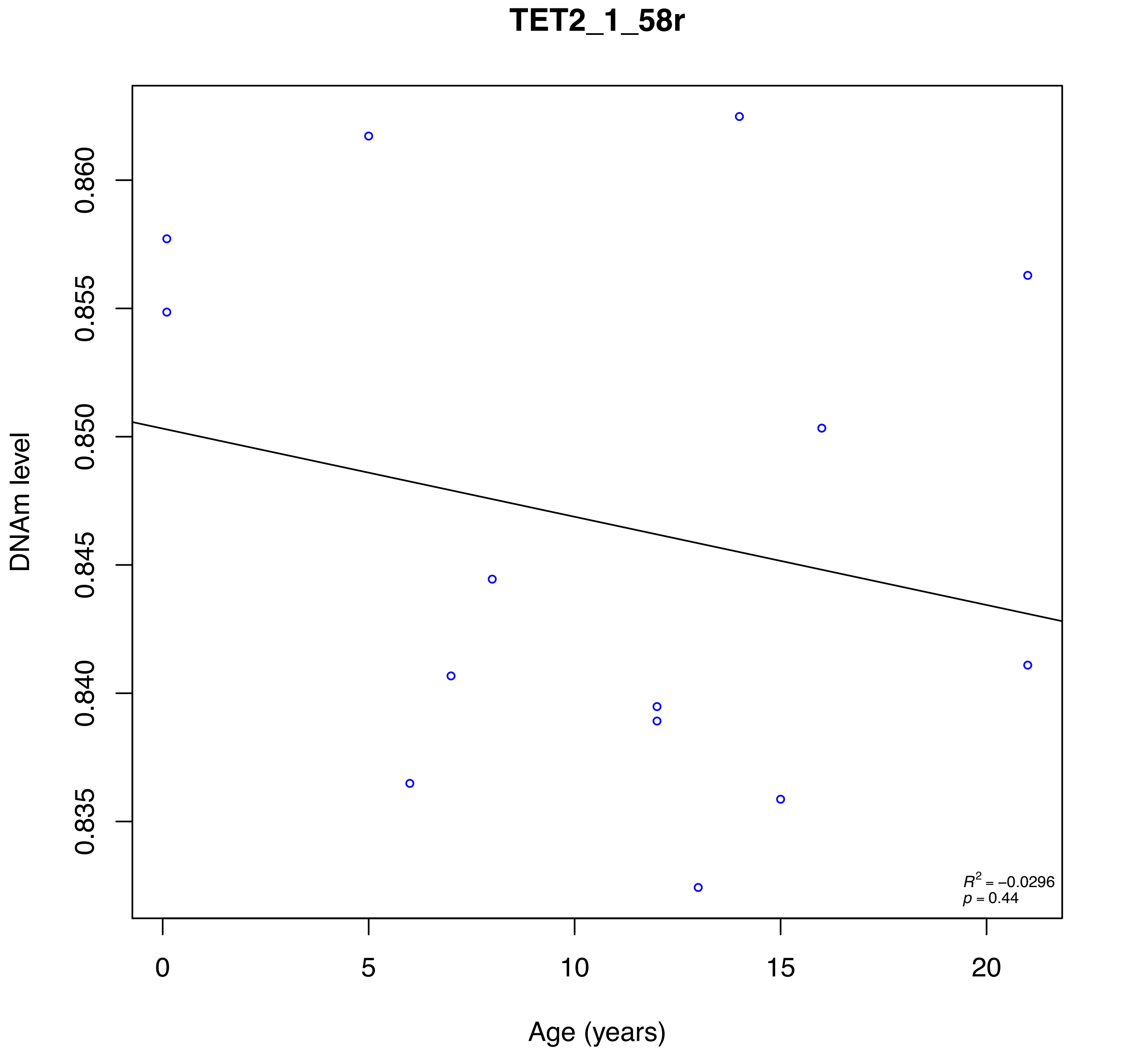
TET2_1_58r
0.860
0.855
0.850
0.845
0.840
0.835
DNAm level
0
5
10
15
20
Age (years)
R^2 = −0.0296
p = 0.44

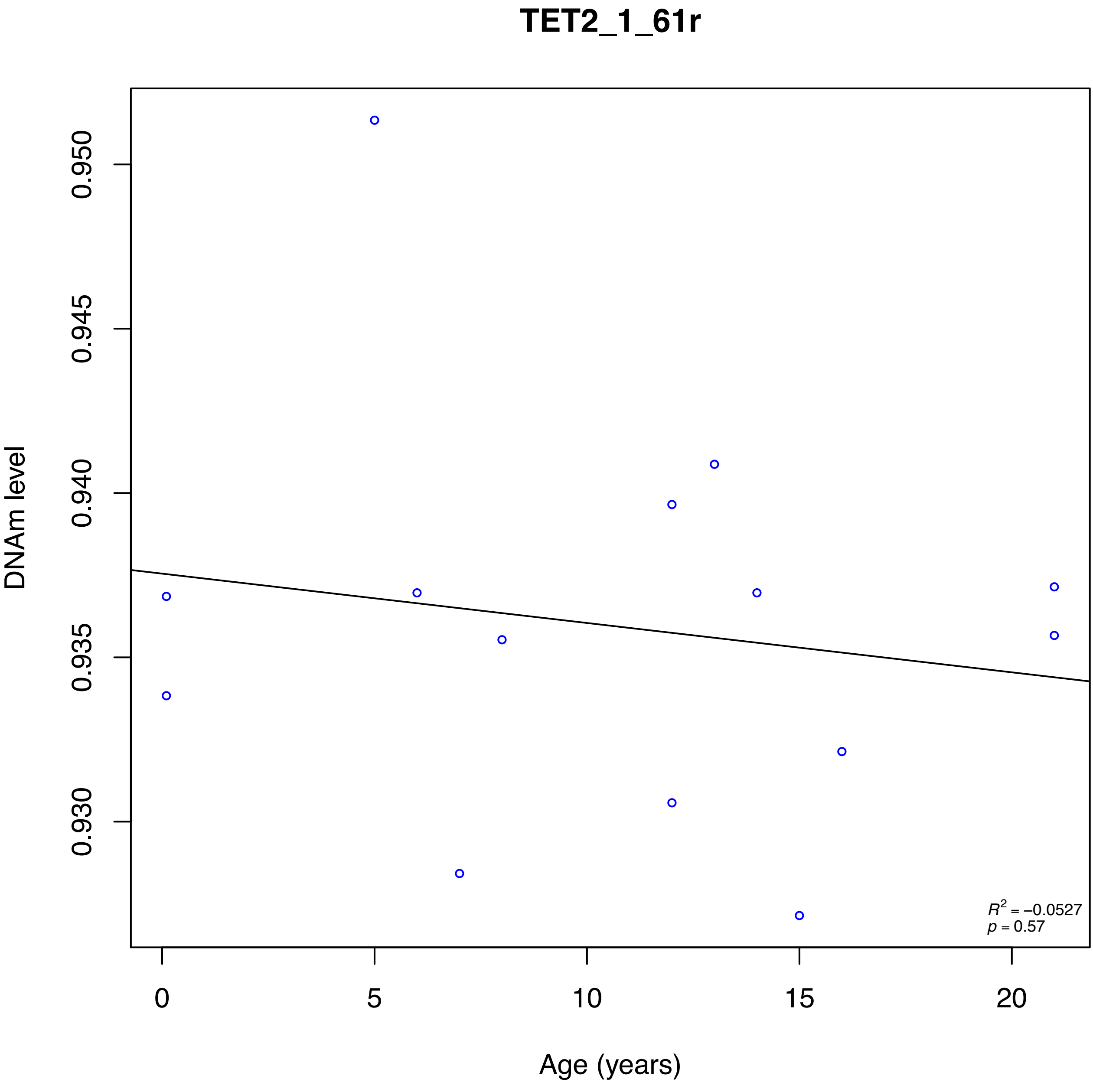
TET2_1_61r
DNAm level
0.930
0.935
0.940
0.945
0.950
Age (years)
0
5
10
15
20
R^2 = −0.0527
p = 0.57

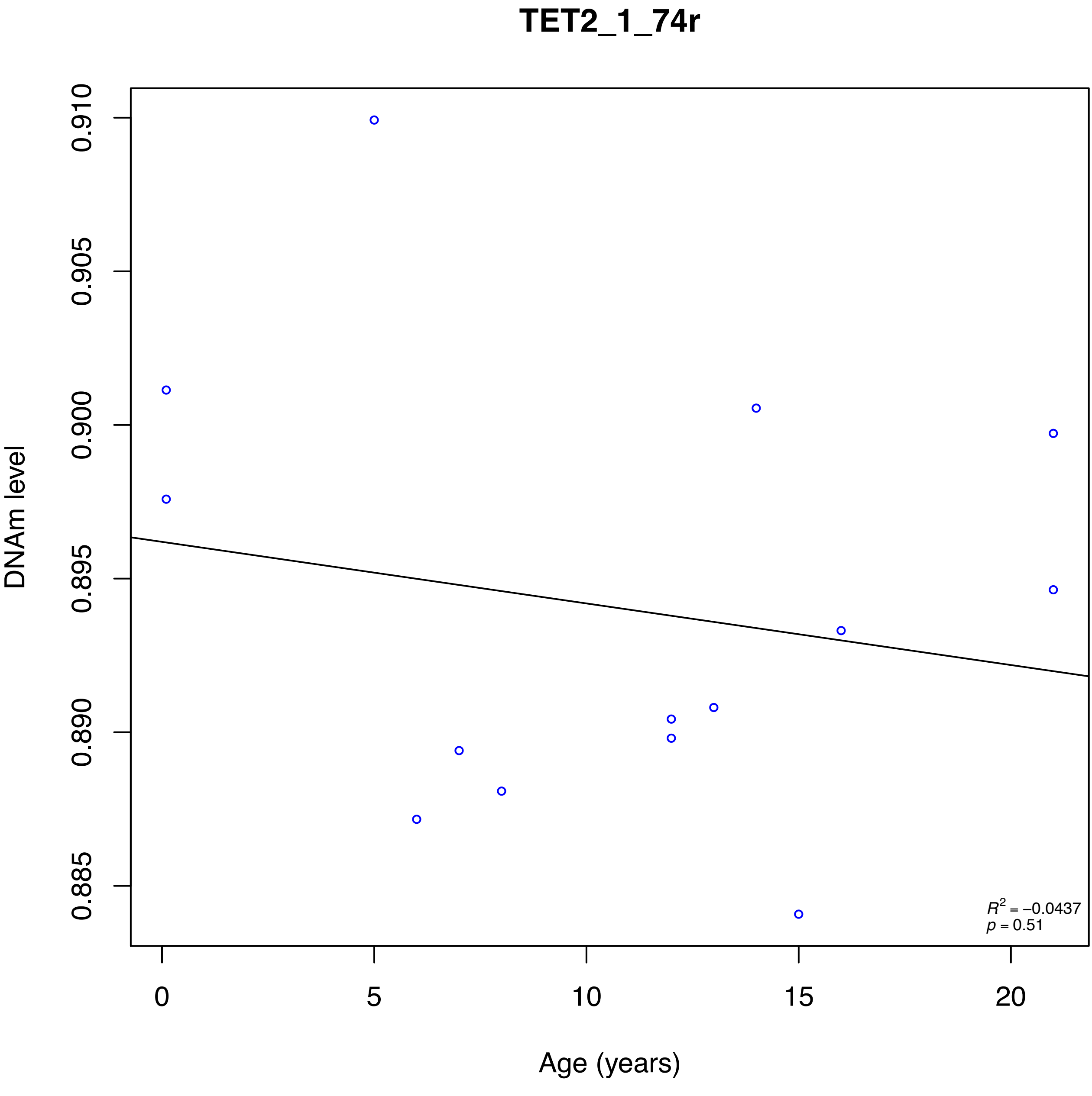

TET2_1_74r
DNAm level
0.910
0.905
0.900
0.895
0.890
0.885
0
5
10
15
20
Age (years)
R2 = −0.0437
p = 0.51

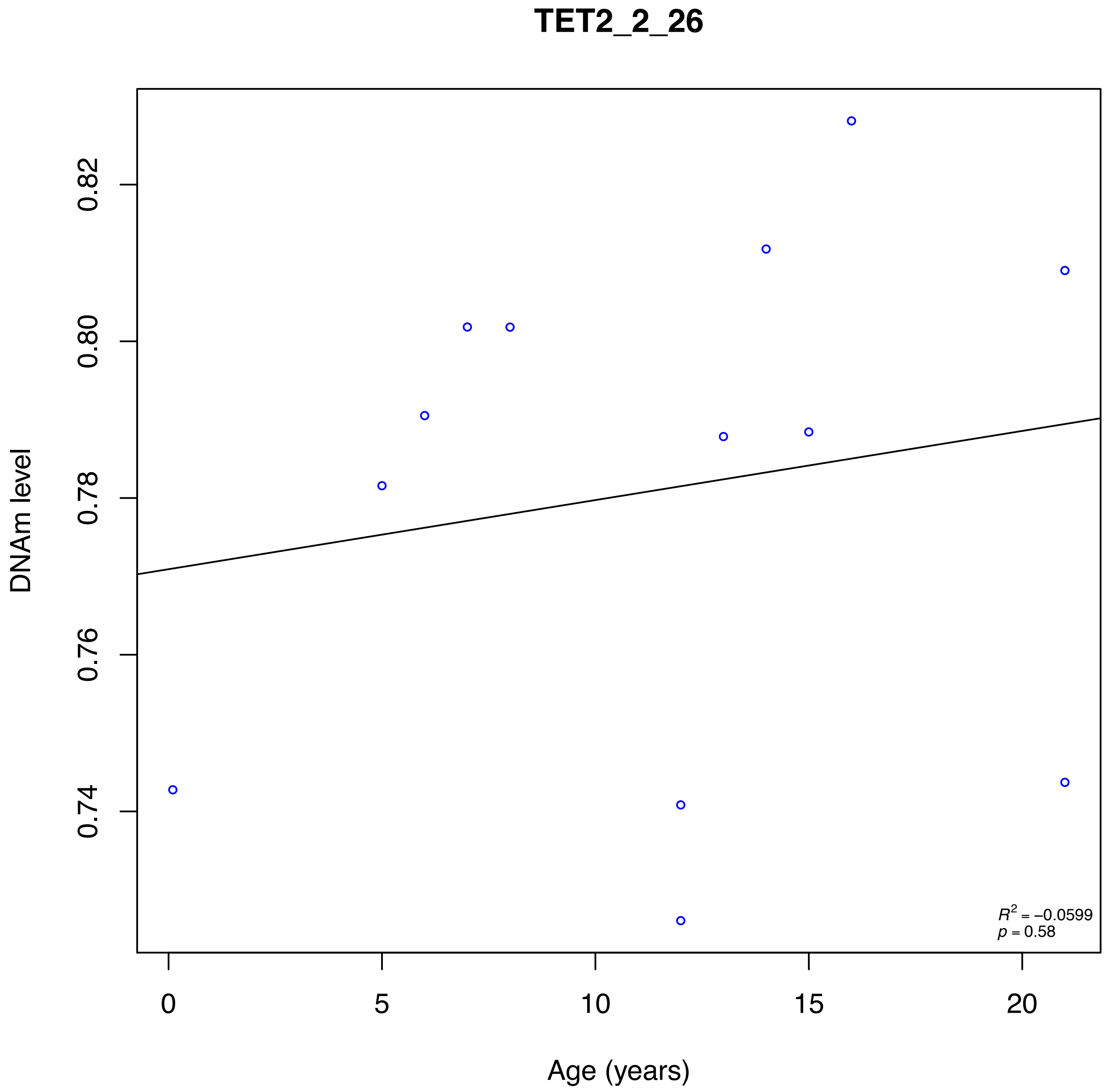

TET2_2_26
DNAm level
0.74
0.76
0.78
0.80
0.82
Age (years)
0
5
10
15
20
R2 = −0.0599
p = 0.58

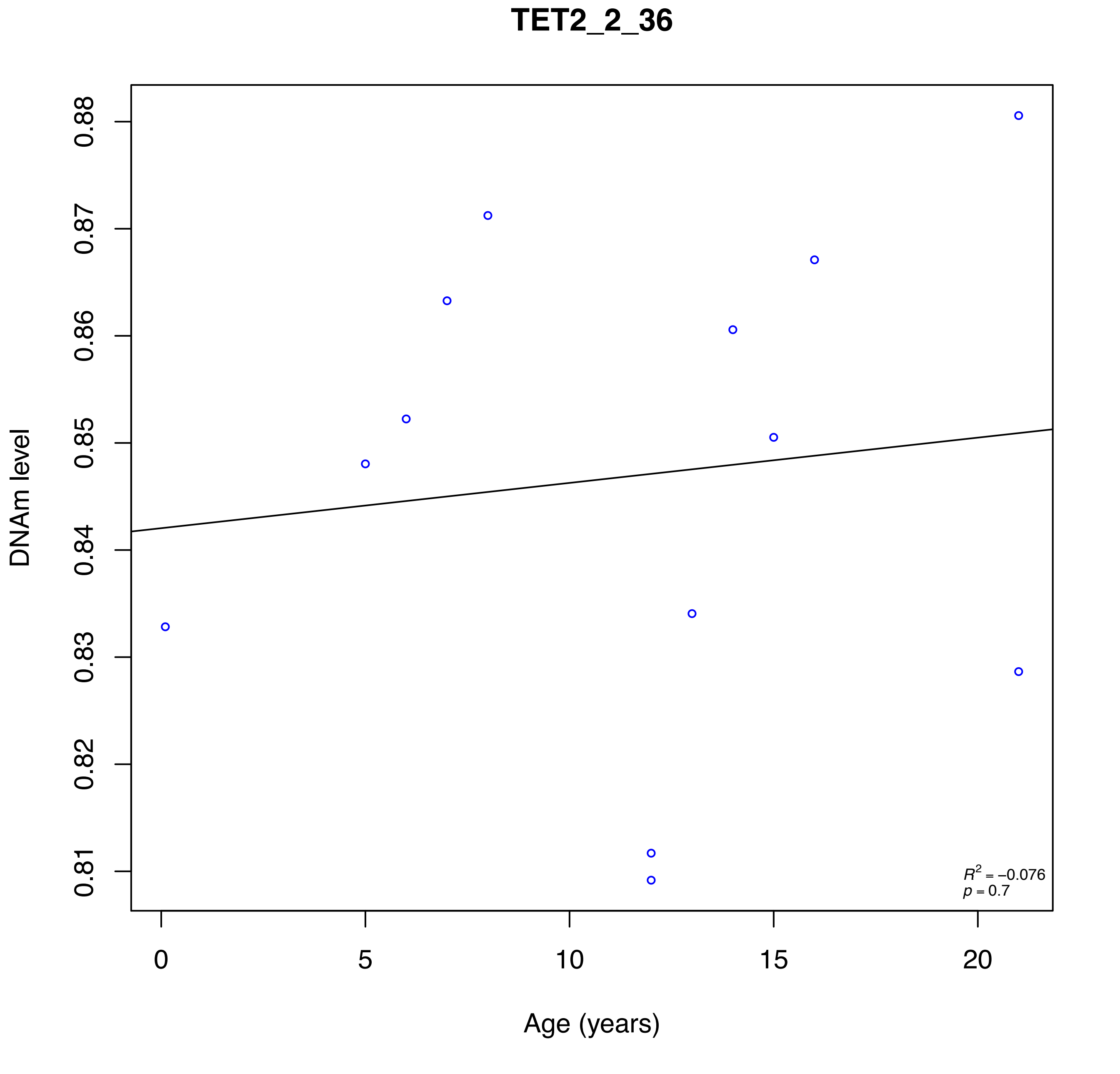
TET2_2_36
DNAm level
0.81
0.82
0.83
0.84
0.85
0.86
0.87
0.88
0
5
10
15
20
Age (years)
$R^2 = -0.076$
$p = 0.7$

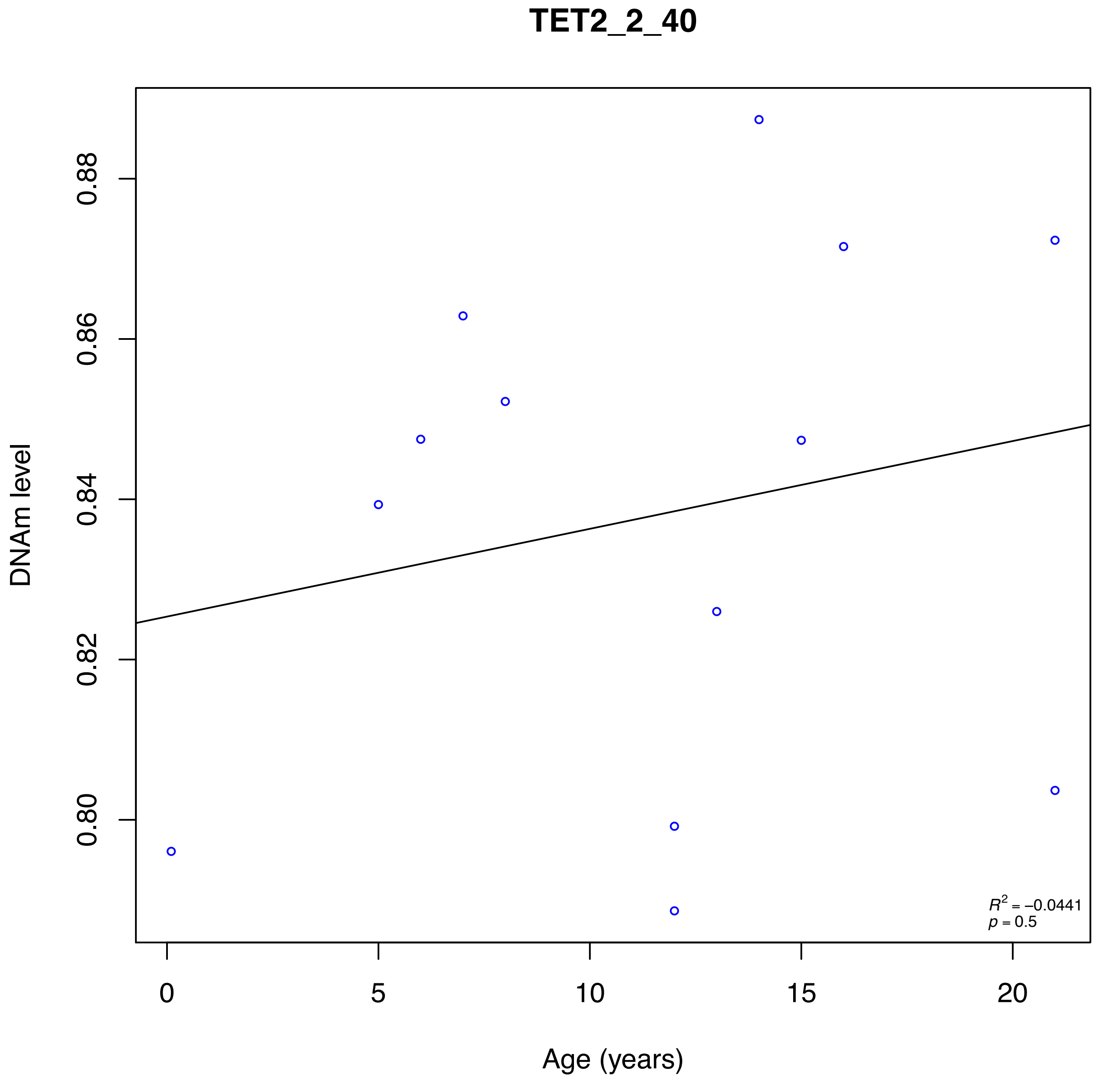

TET2_2_40
DNAm level
0.80
0.82
0.84
0.86
0.88
0
5
10
15
20
Age (years)
R^2 = −0.0441
p = 0.5

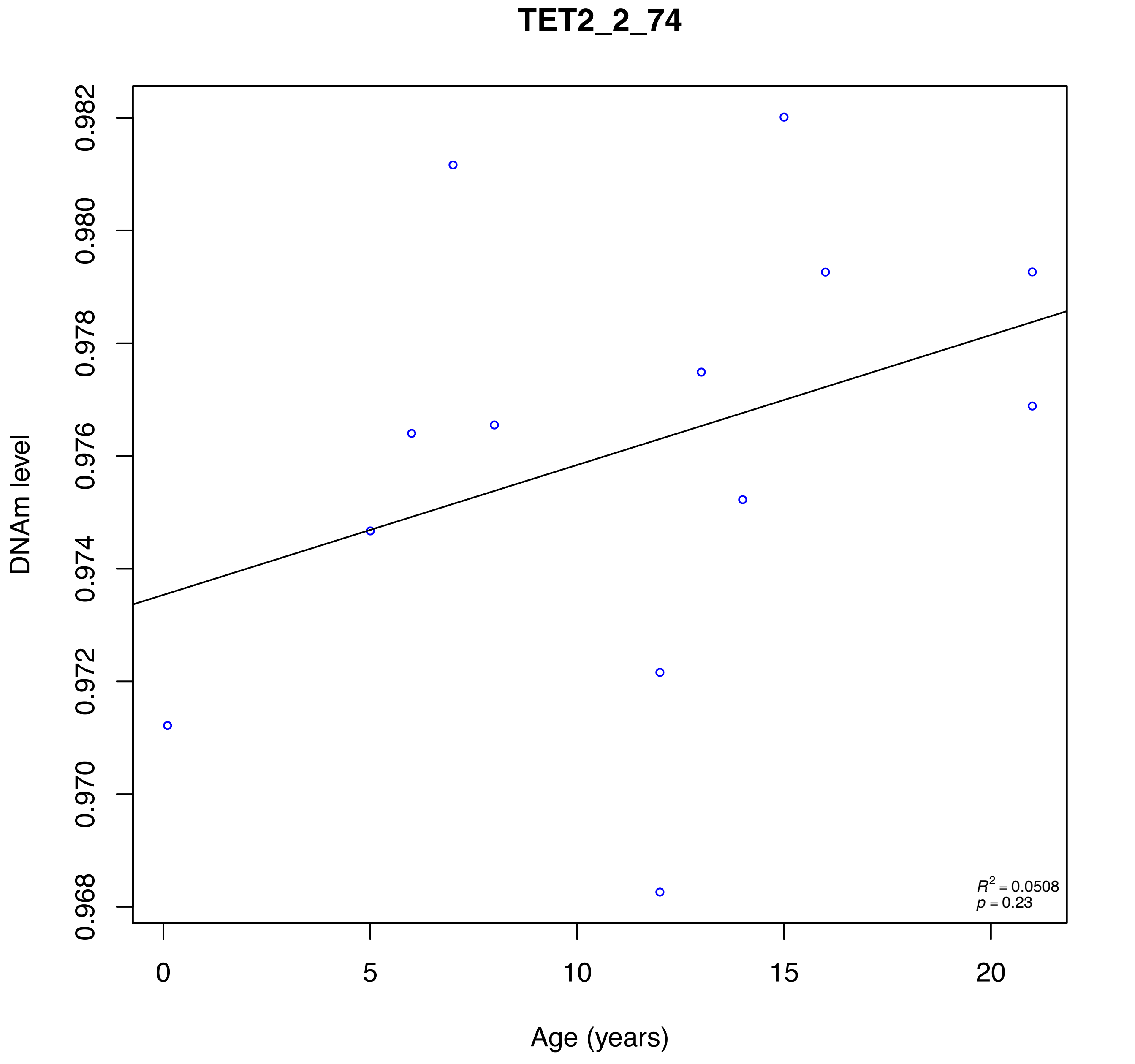

TET2_2_74
DNAm level
0.968
0.970
0.972
0.974
0.976
0.978
0.980
0.982
0
5
10
15
20
Age (years)
R^2 = 0.0508
p = 0.23

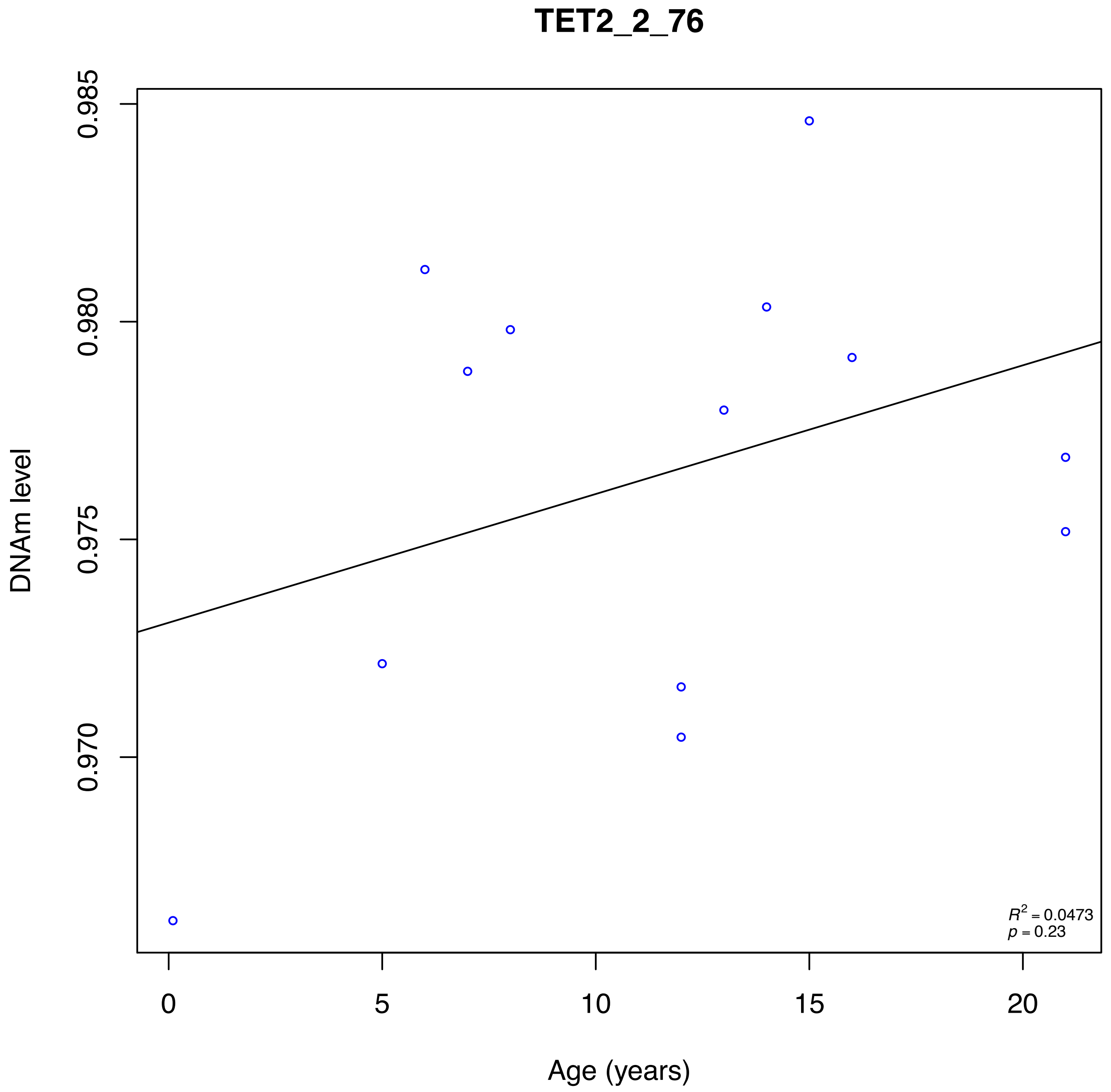
TET2_2_76
DNAm level
Age (years)
0.970
0.975
0.980
0.985
0
5
10
15
20
R^2 = 0.0473
p = 0.23

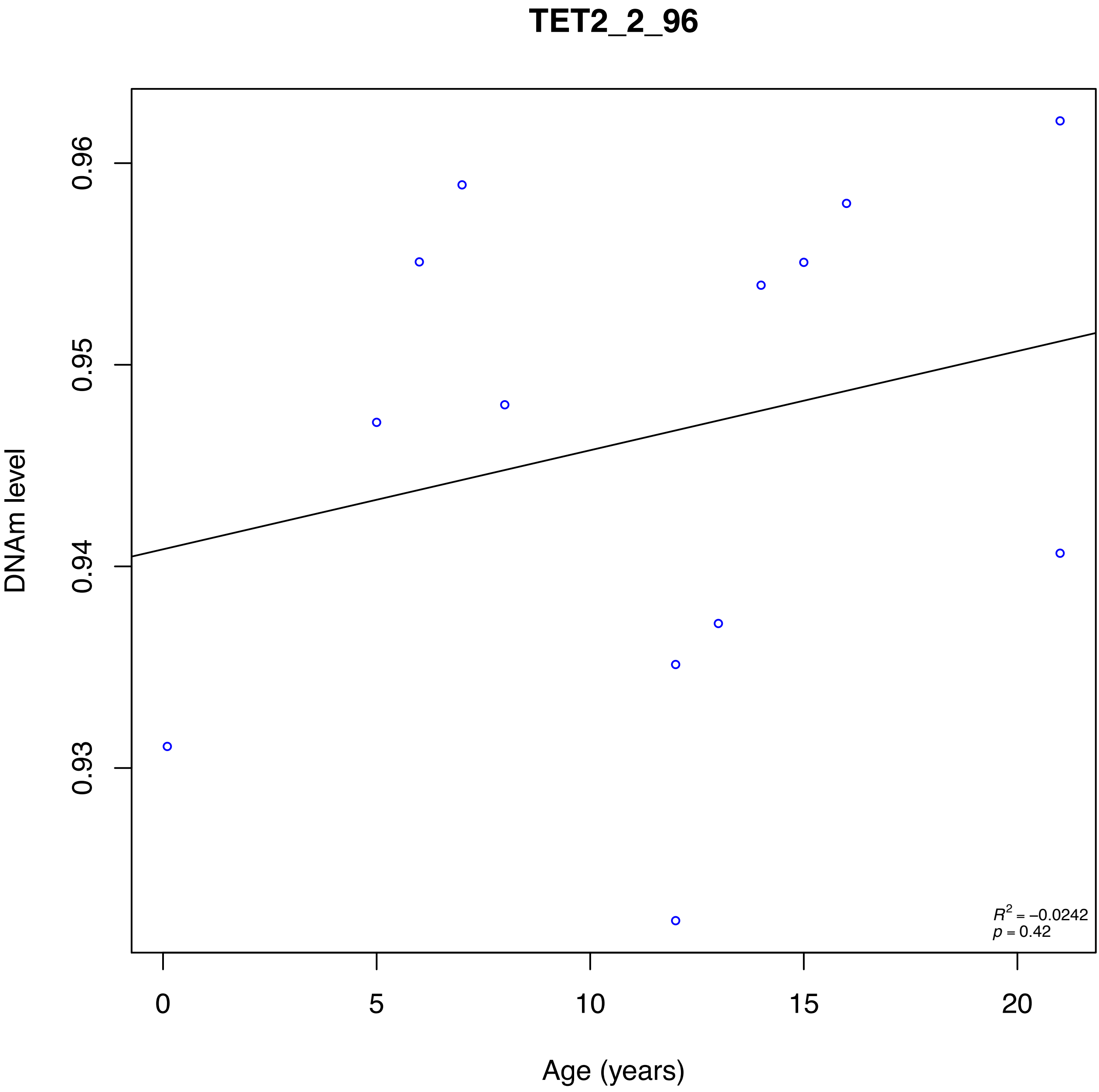
TET2_2_96
DNAm level
0.93
0.94
0.95
0.96
Age (years)
0
5
10
15
20
R^2 = −0.0242
p = 0.42
