# Supplementary material for: DNA methylation levels in candidate genes associated with chronological age in mammals are not conserved in a long-lived seabird
Source: PLoS One. 2017 Dec 7;12(12):e0189181. doi: 10.1371/journal.pone.0189181 (PMC5720723; doi:10.1371/journal.pone.0189181)
Supplement: S3 File — (PDF) [file pone.0189181.s008.pdf]

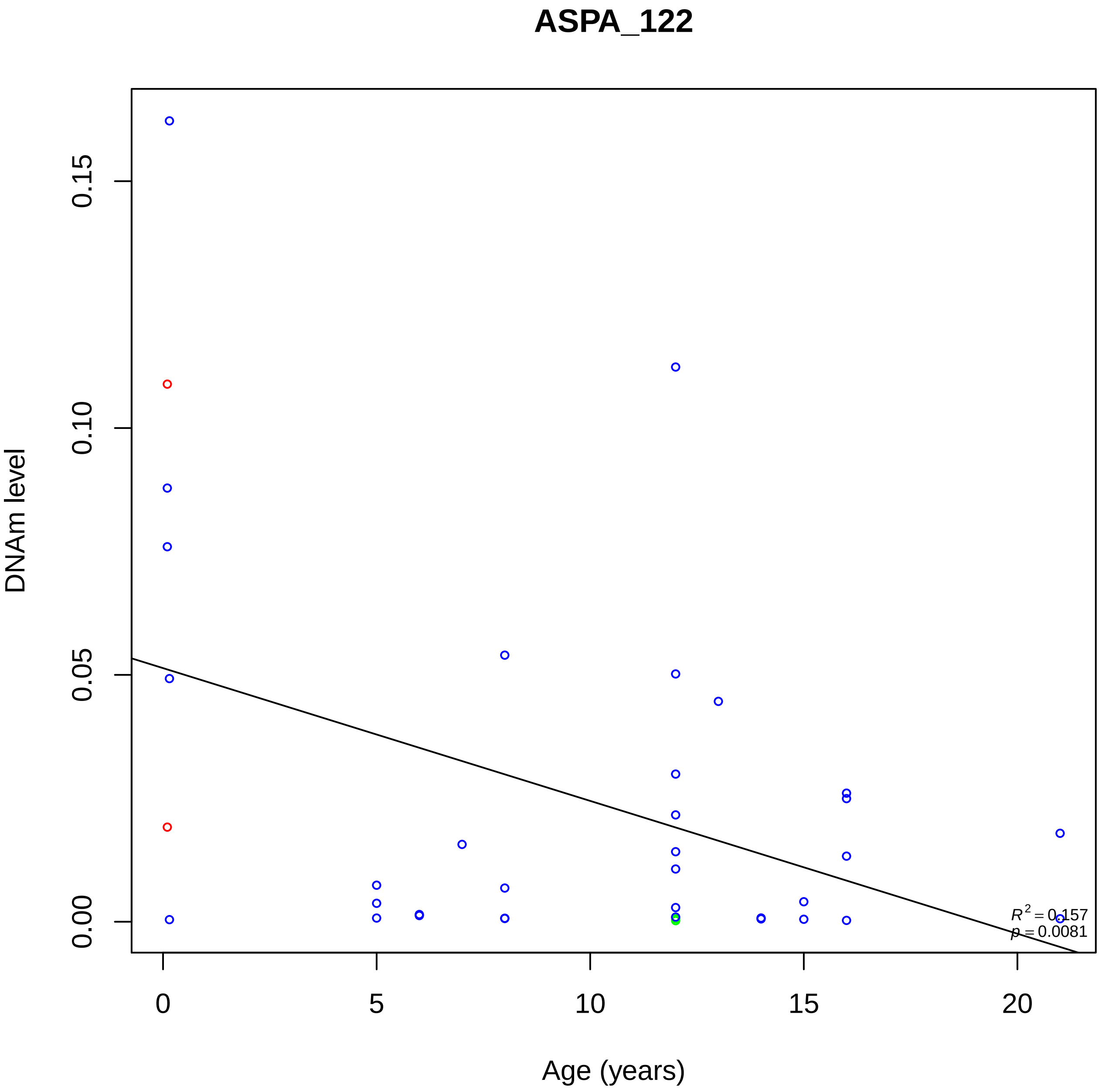
ASPA_122
DNAm level
0.00
0.05
0.10
0.15
0
5
10
15
20
Age (years)
$R^2 = 0.157$
$p = 0.0081$

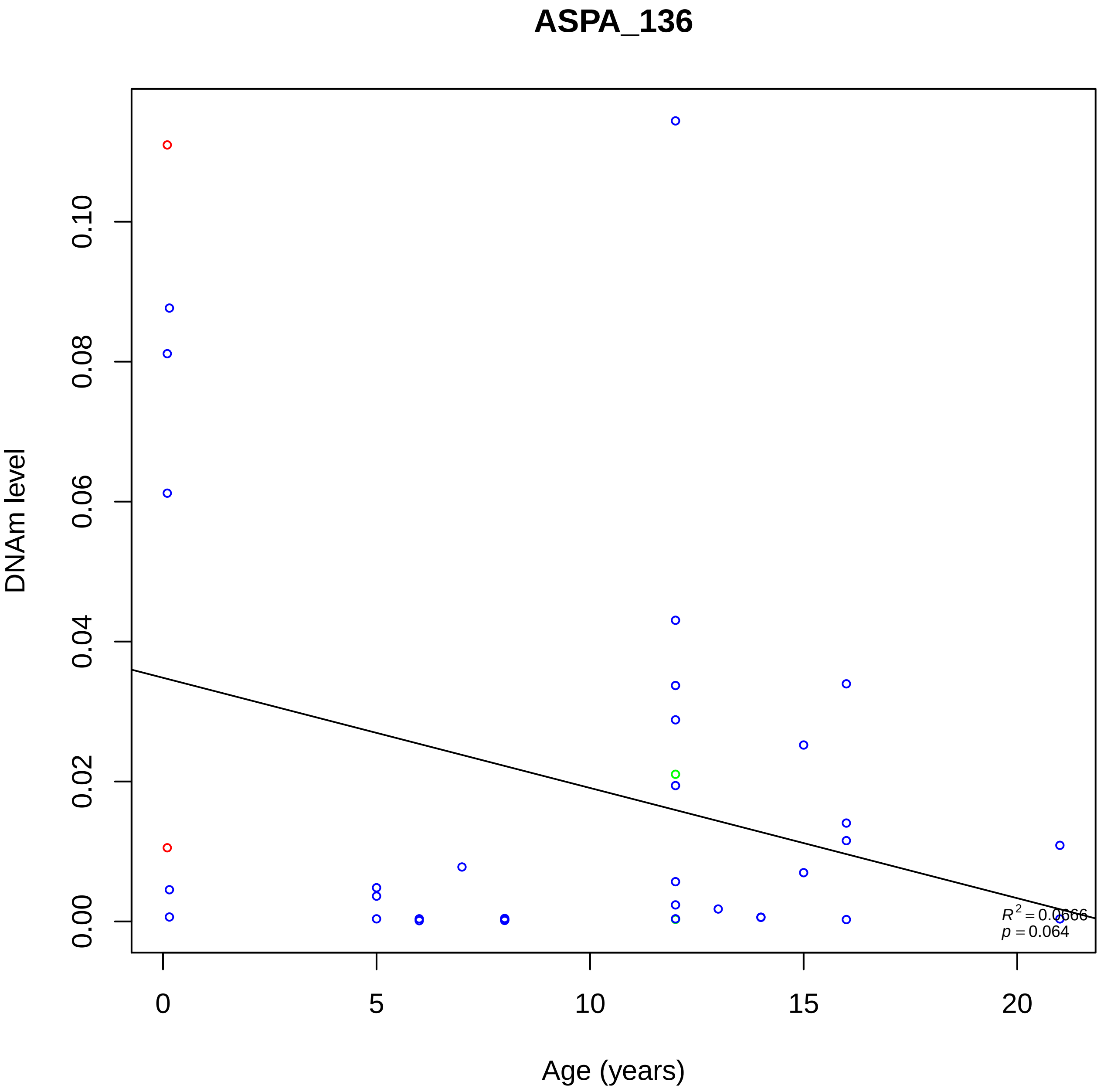
ASPA_136
DNAm level
0.00
0.02
0.04
0.06
0.08
0.10
Age (years)
0
5
10
15
20
$R^2 = 0.0666$
$p = 0.064$

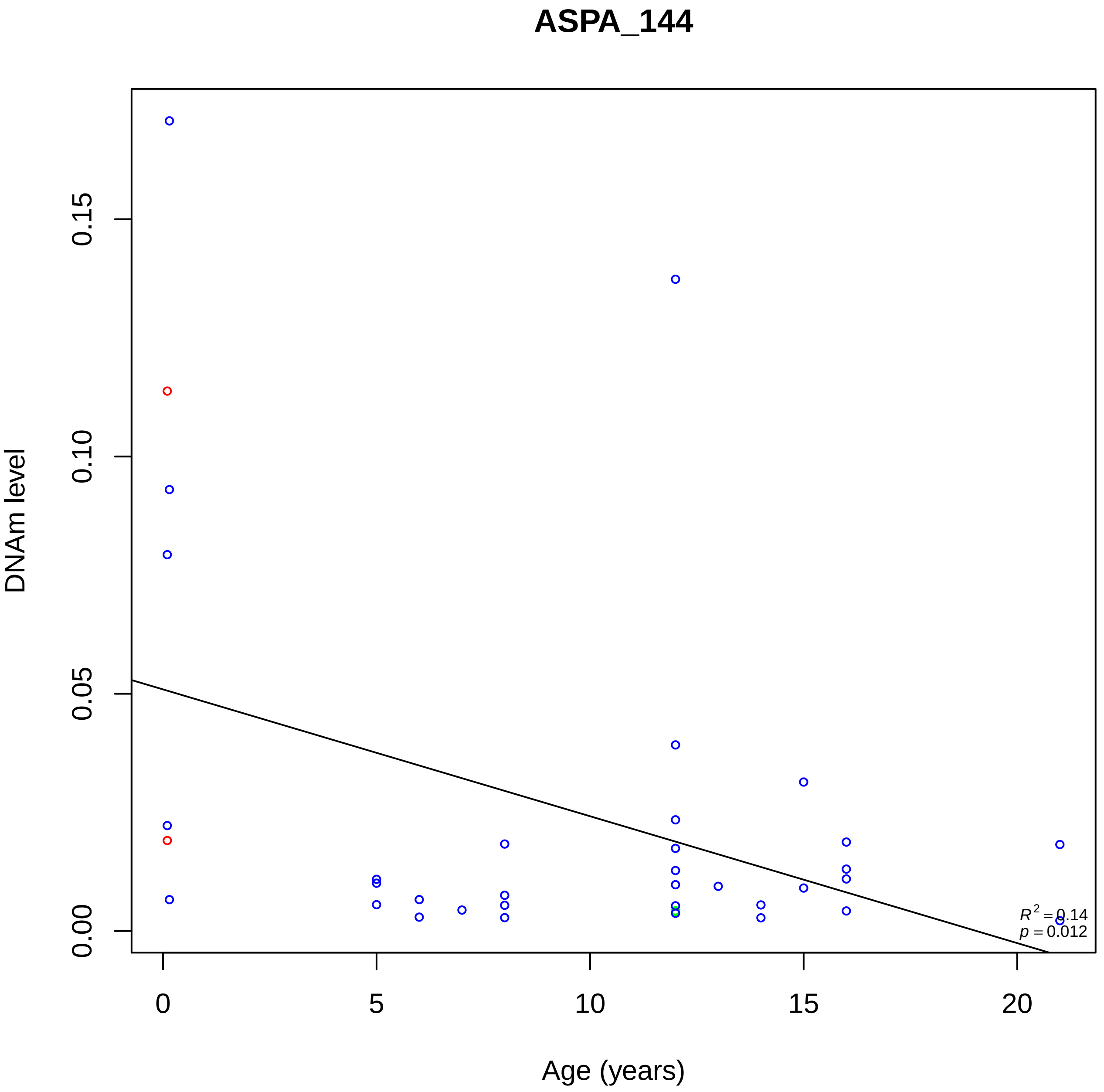
ASPA_144
DNAm level
0.00
0.05
0.10
0.15
Age (years)
0
5
10
15
20
$R^2 = 0.14$
$p = 0.012$

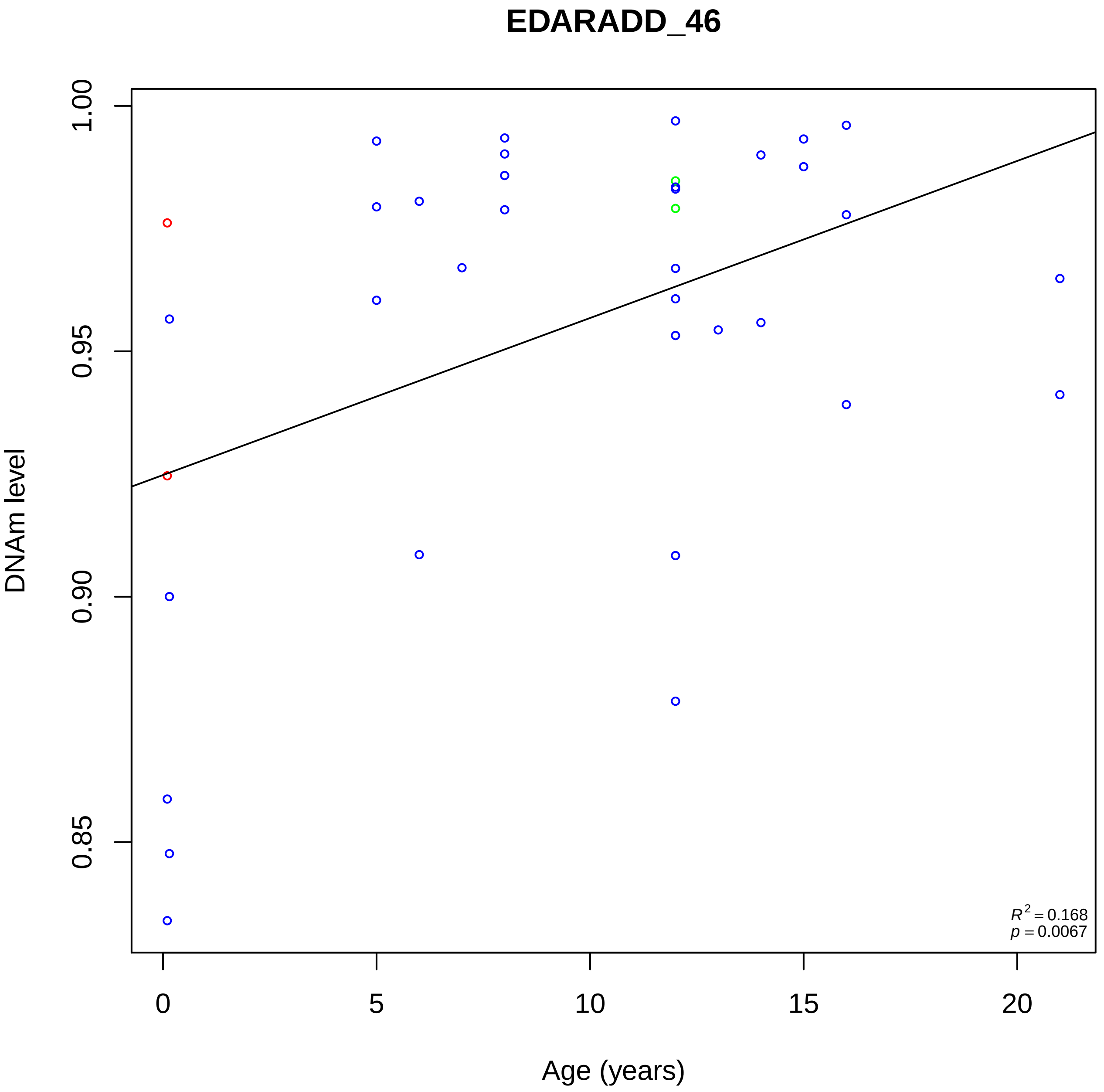

EDARADD_46
DNAm level
1.00
0.95
0.90
0.85
0
5
10
15
20
Age (years)
$R^2 = 0.168$
$p = 0.0067$

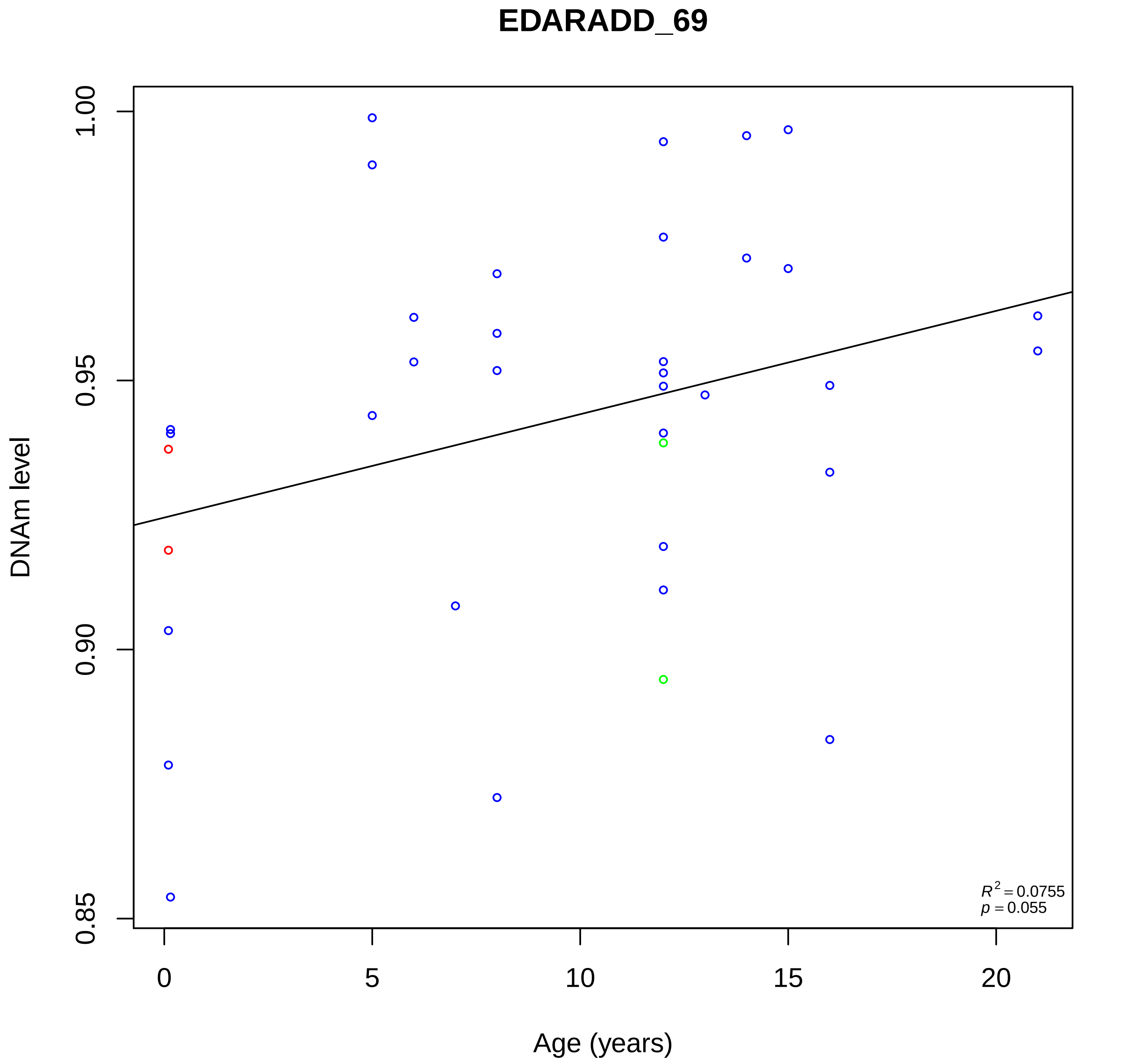

EDARADD_69
DNAm level
Age (years)
0.85
0.90
0.95
1.00
0
5
10
15
20
$R^2 = 0.0755$
$p = 0.055$

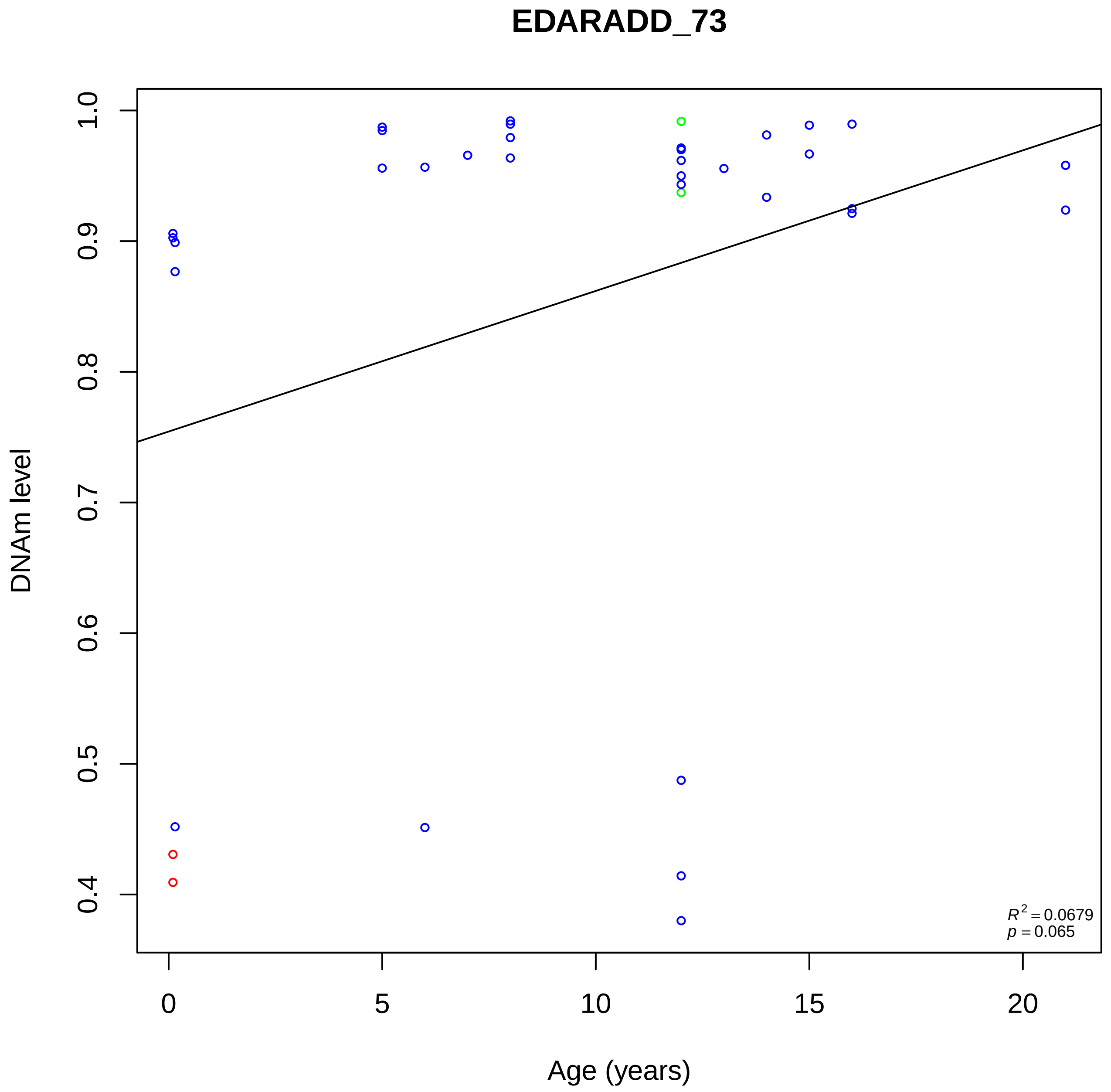

EDARADD_73
DNAm level
1.0
0.9
0.8
0.7
0.6
0.5
0.4
0
5
10
15
20
Age (years)
$R^2 = 0.0679$
$p = 0.065$

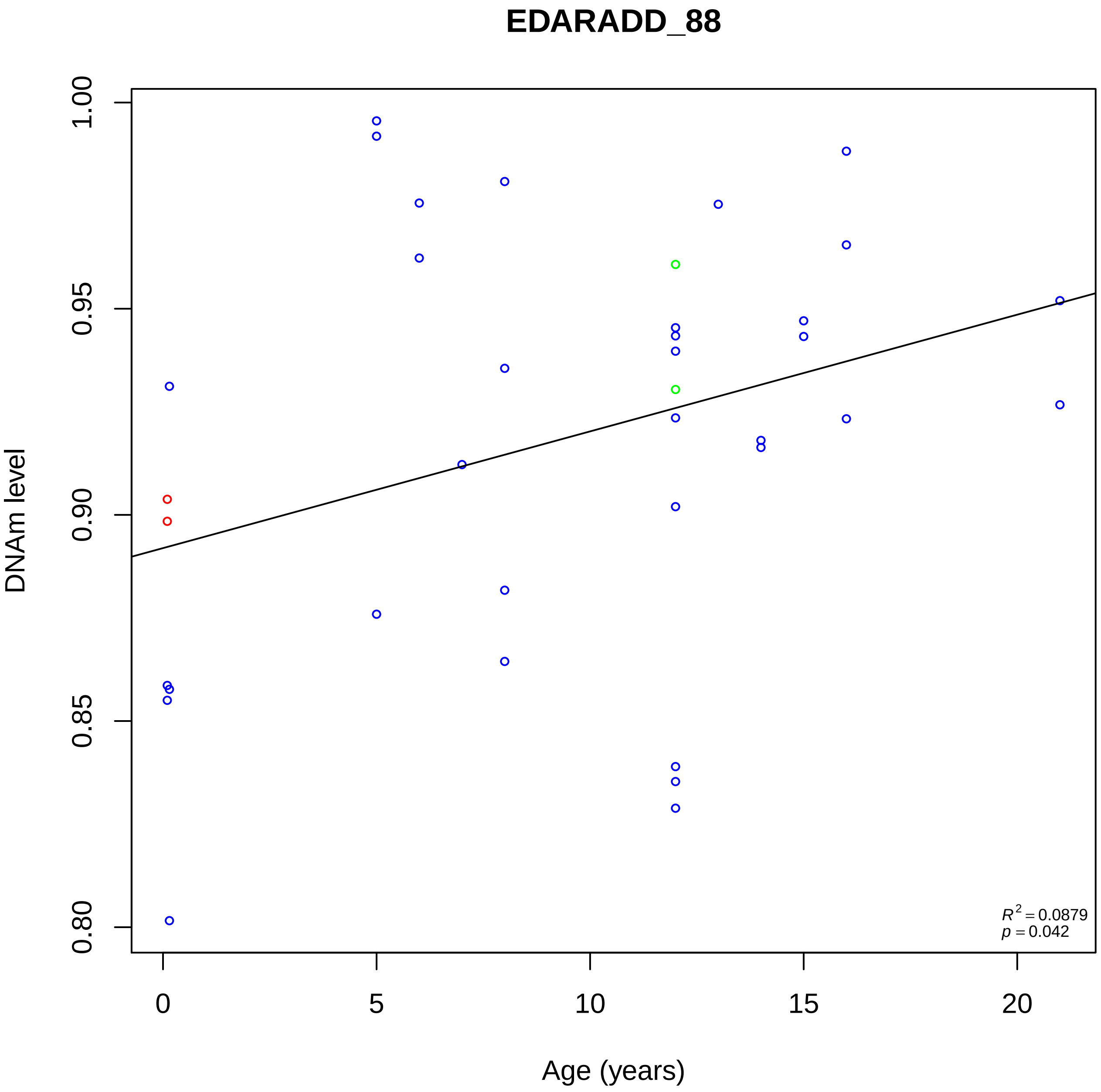

EDARADD_88
DNAm level
1.00
0.95
0.90
0.85
0.80
0
5
10
15
20
Age (years)
$R^2 = 0.0879$
$p = 0.042$

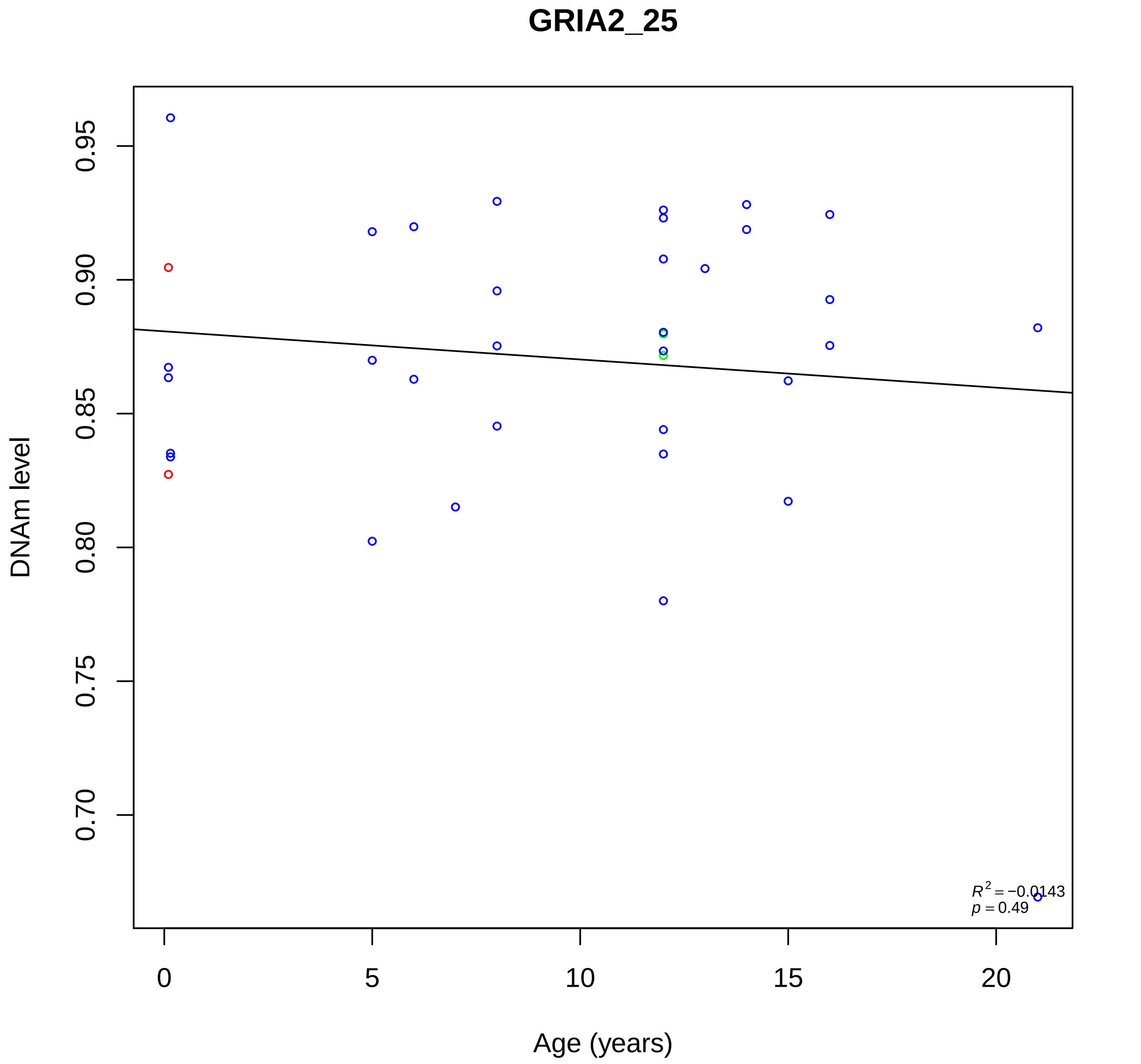

GRIA2_25
0.95
0.90
0.85
0.80
0.75
0.70
DNAm level
0
5
10
15
20
Age (years)
$R^2 = -0.0143$
$p = 0.49$

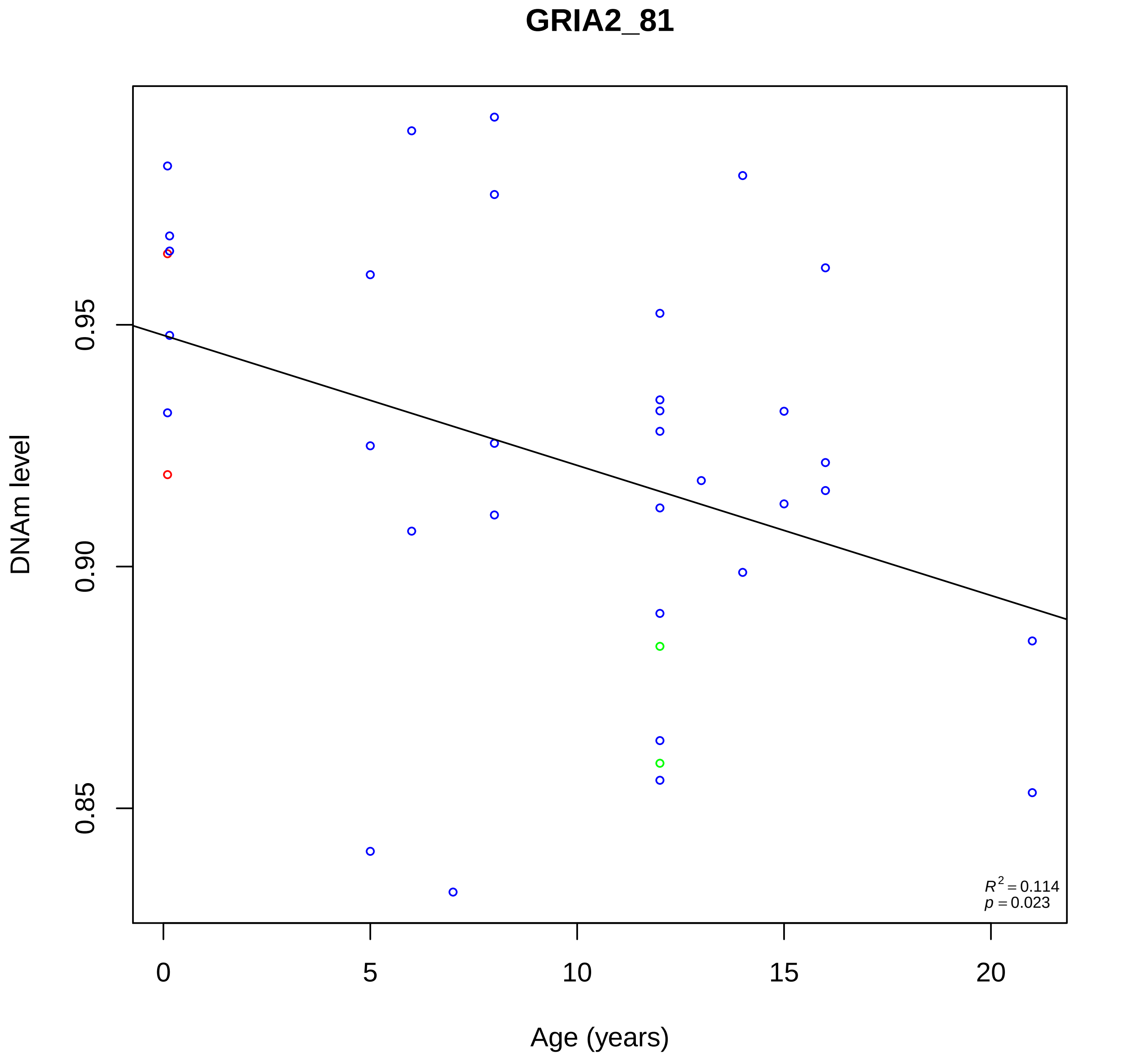
GRIA2_81
DNAm level
0.85
0.90
0.95
0
5
10
15
20
Age (years)
$R^2 = 0.114$
$p = 0.023$

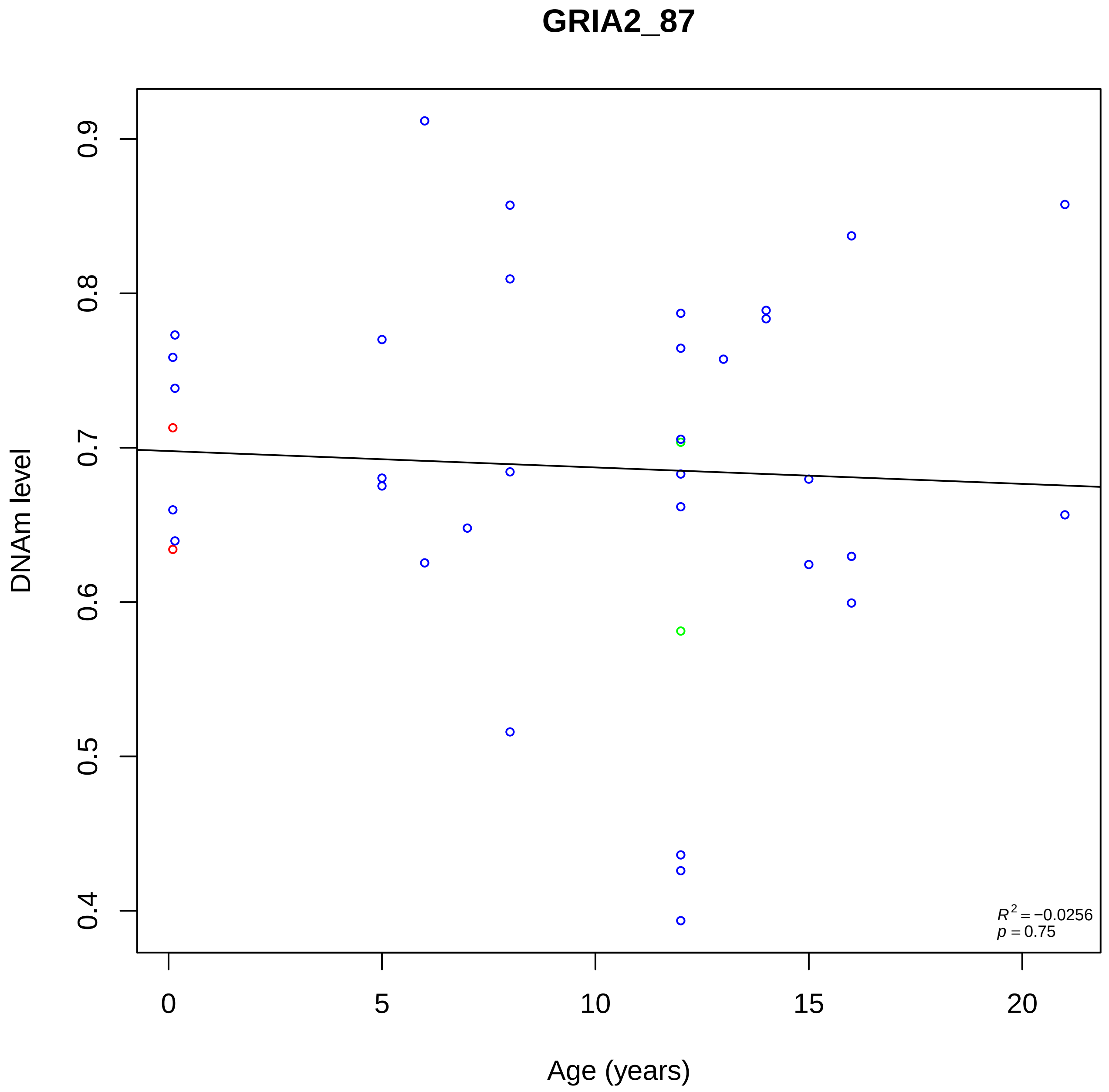

GRIA2_87
DNAm level
Age (years)
0.4
0.5
0.6
0.7
0.8
0.9
0
5
10
15
20
$R^2 = -0.0256$
$p = 0.75$

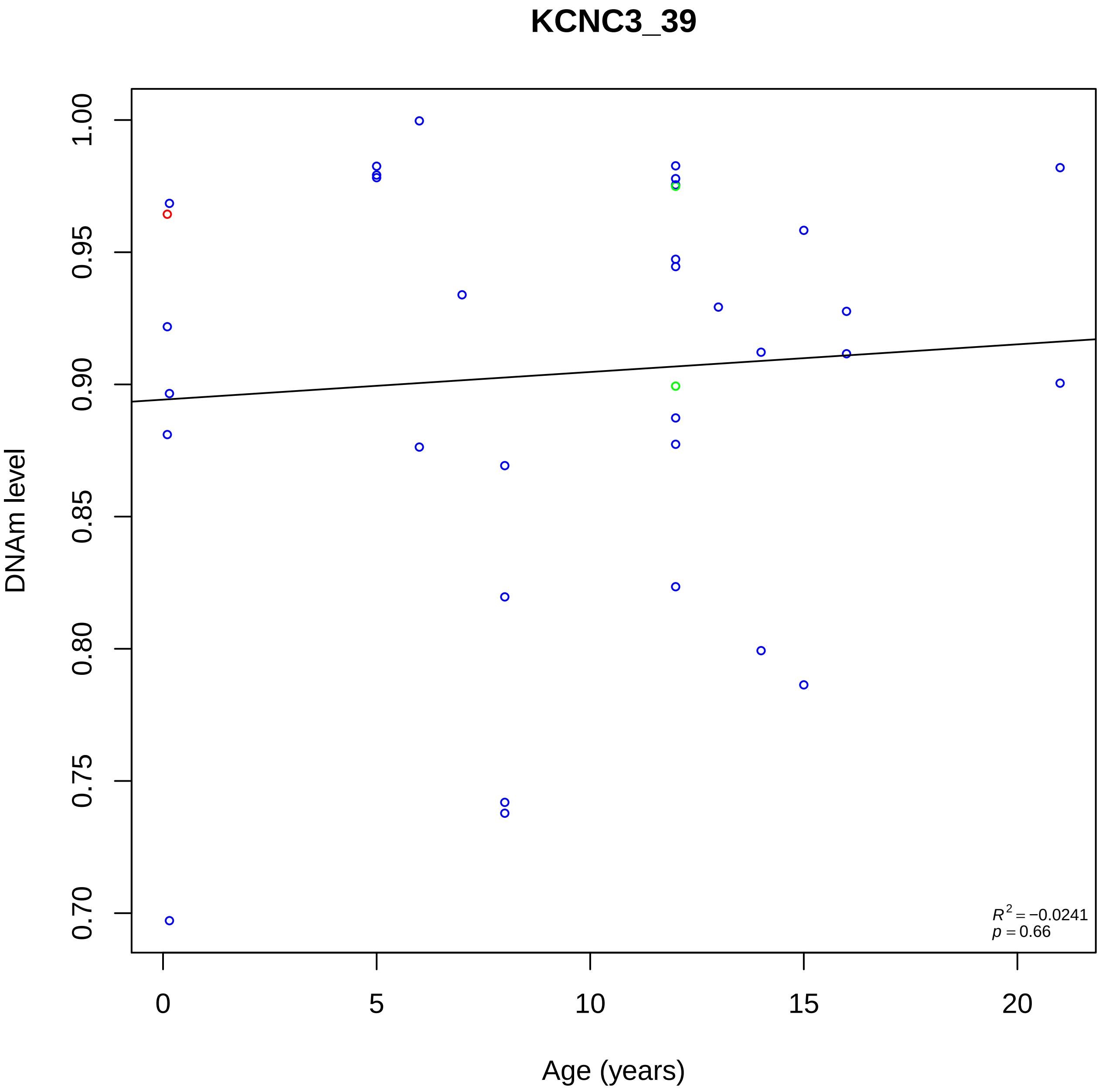

KCNC3_39
DNAm level
1.00
0.95
0.90
0.85
0.80
0.75
0.70
0
5
10
15
20
Age (years)
$R^2 = -0.0241$
$p = 0.66$

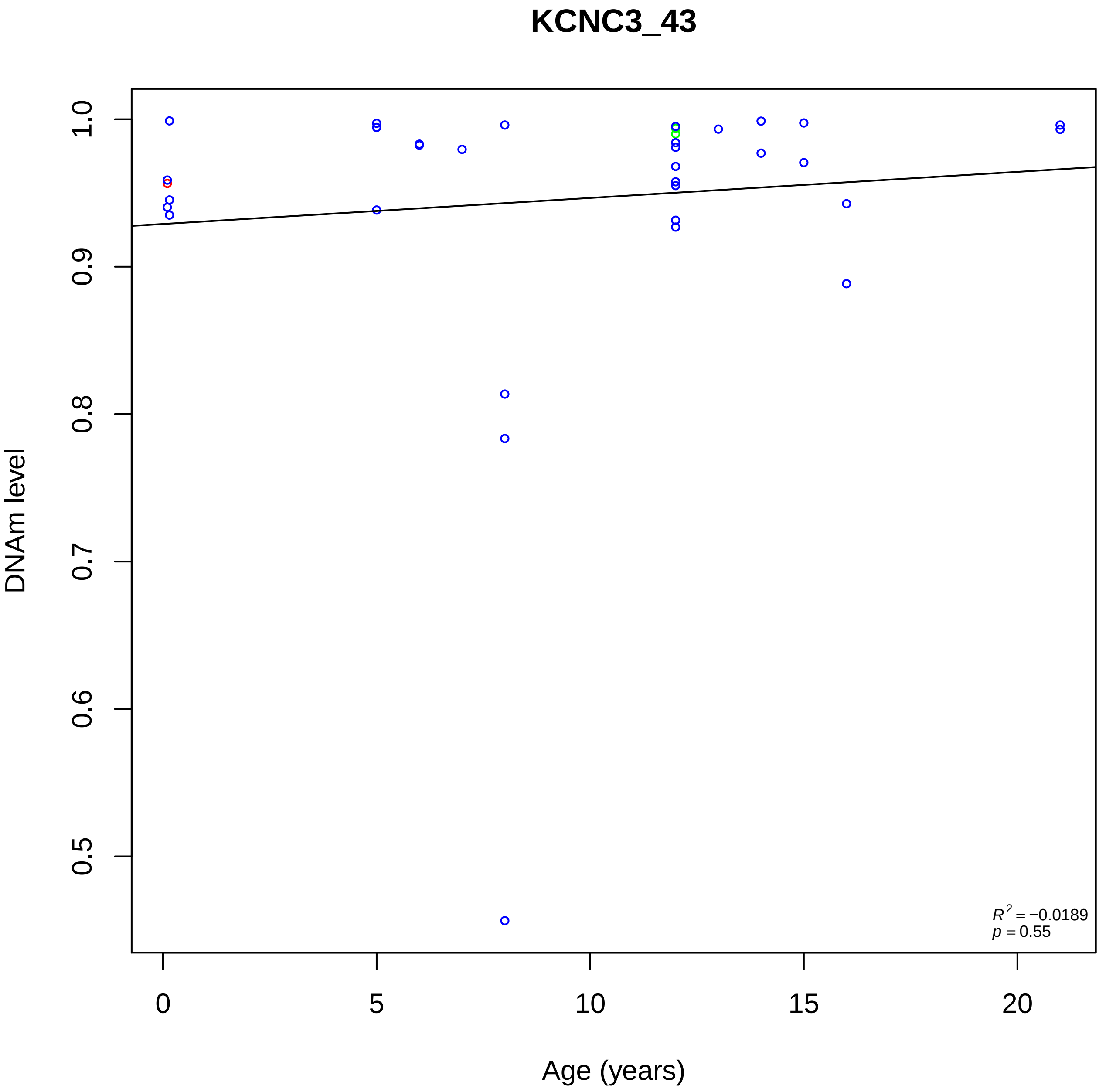
KCNC3_43
DNAm level
Age (years)
1.0
0.9
0.8
0.7
0.6
0.5
0
5
10
15
20
$R^2 = -0.0189$
$p = 0.55$

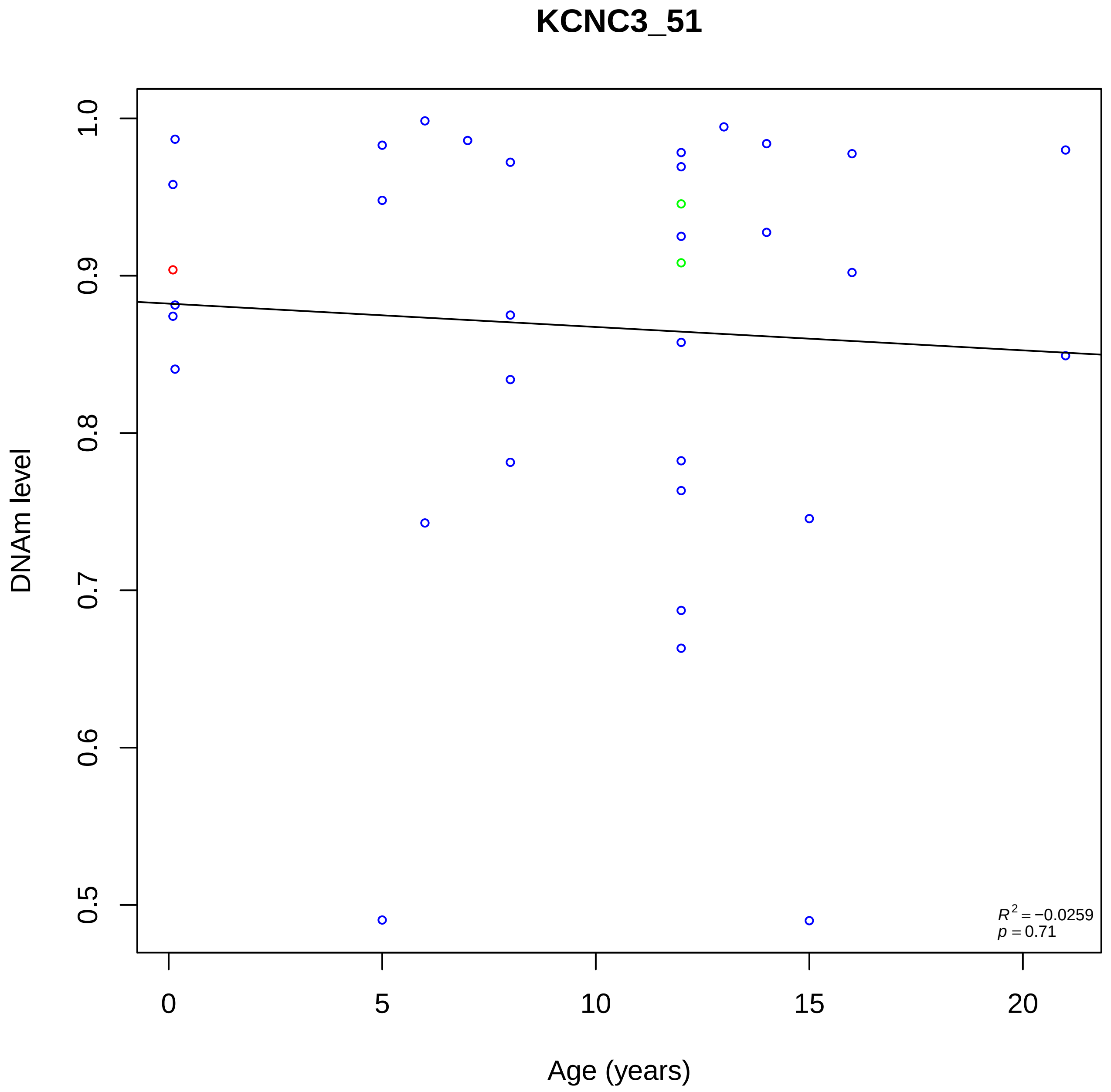

KCNC3_51
DNAm level
Age (years)
0
5
10
15
20
0.5
0.6
0.7
0.8
0.9
1.0
$R^2 = -0.0259$
$p = 0.71$

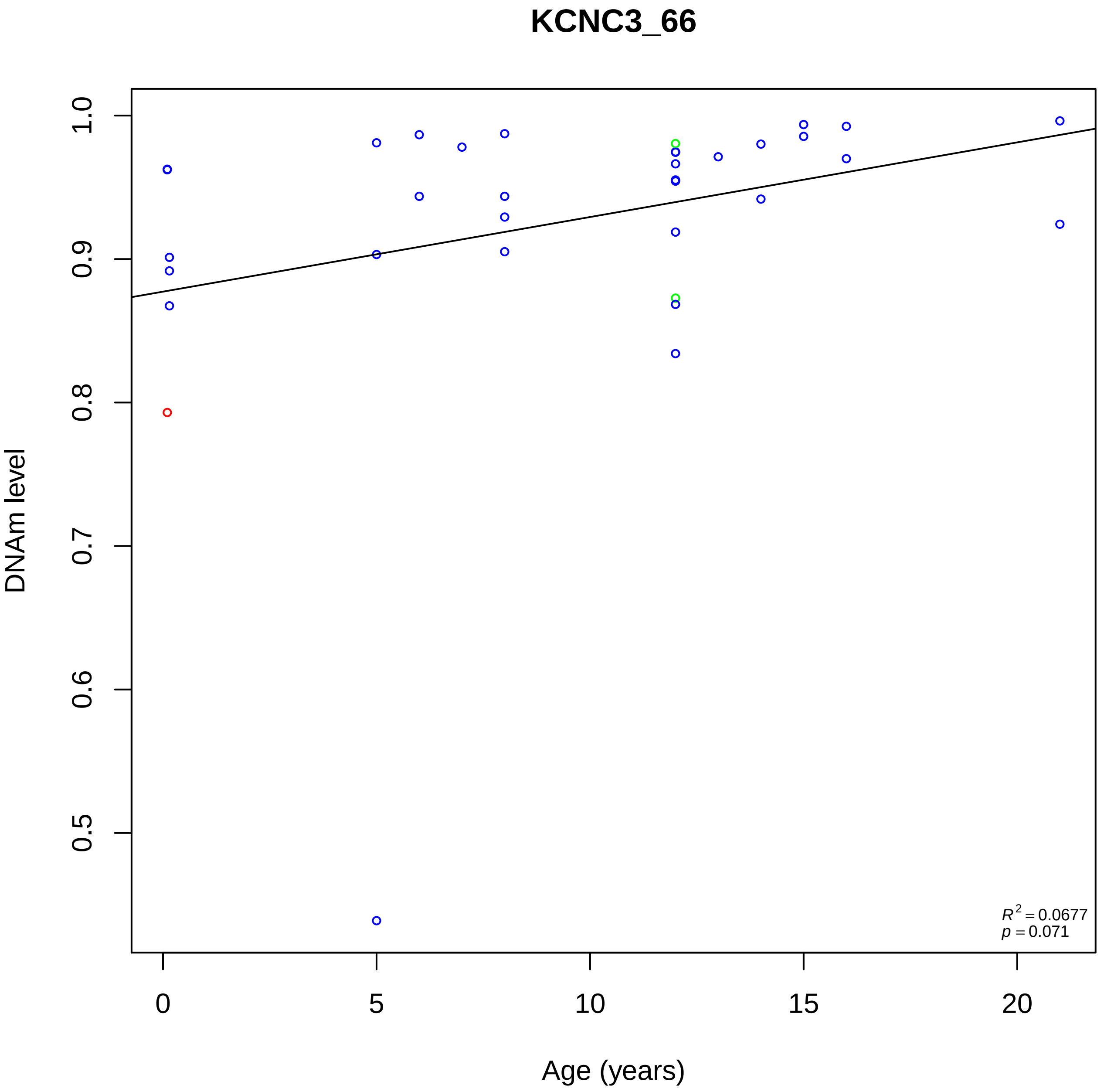
KCNC3_66
DNAm level
Age (years)
0
5
10
15
20
0.5
0.6
0.7
0.8
0.9
1.0
$R^2 = 0.0677$
$p = 0.071$

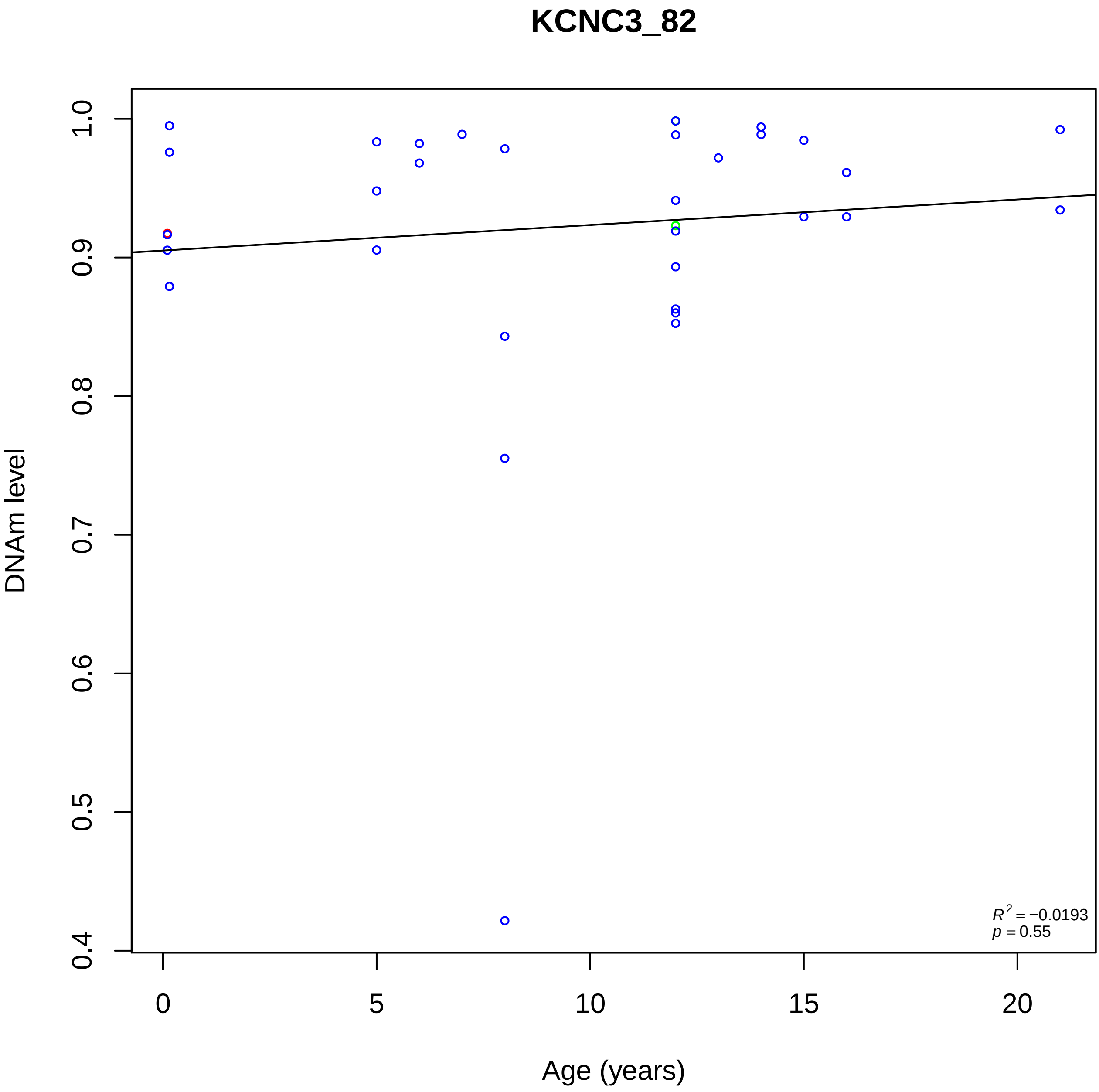

KCNC3_82
DNAm level
Age (years)
0.4
0.5
0.6
0.7
0.8
0.9
1.0
0
5
10
15
20
$R^2 = -0.0193$
$p = 0.55$

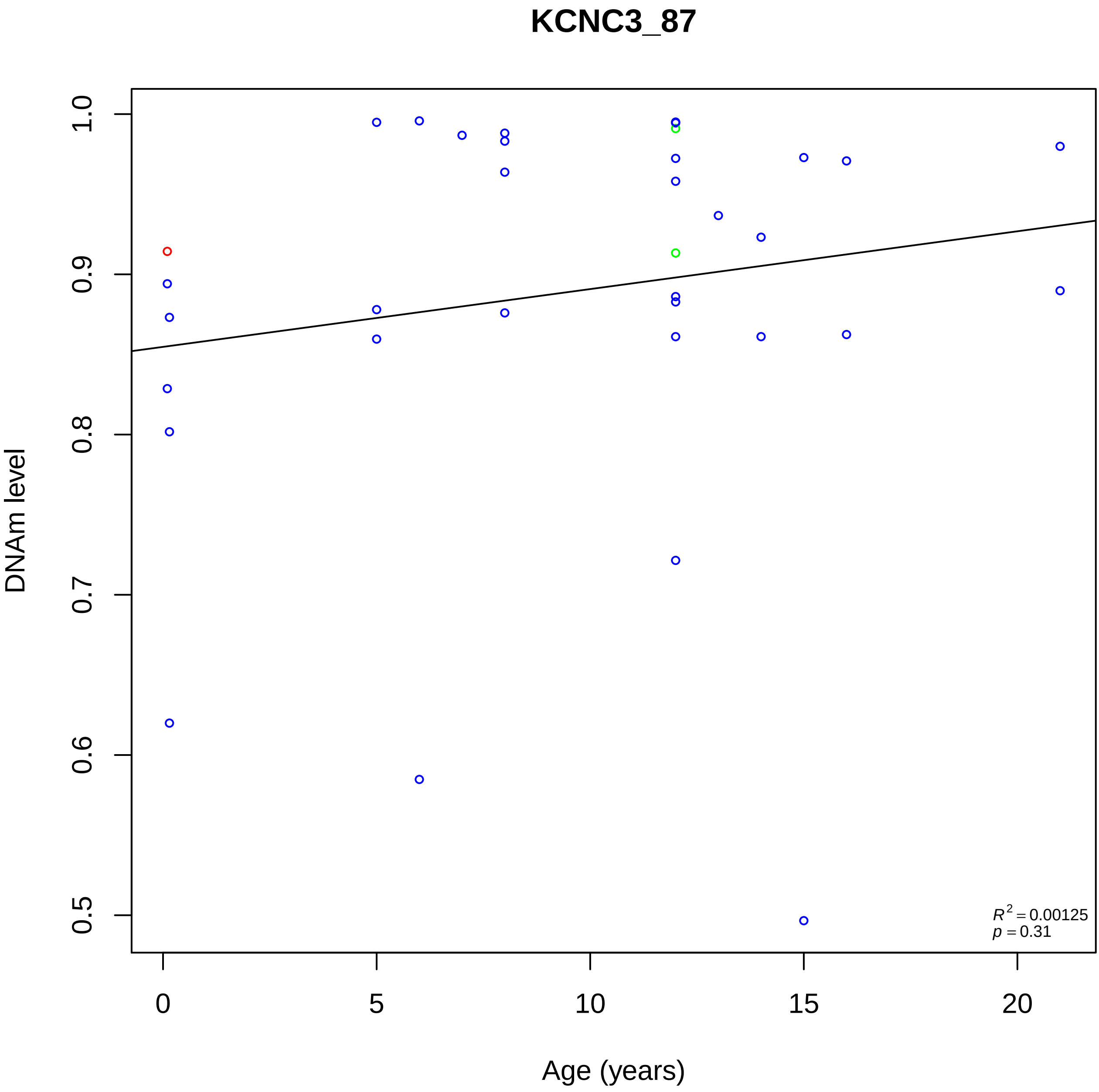
KCNC3_87
DNAm level
Age (years)
0.5
0.6
0.7
0.8
0.9
1.0
0
5
10
15
20
$R^2 = 0.00125$
$p = 0.31$

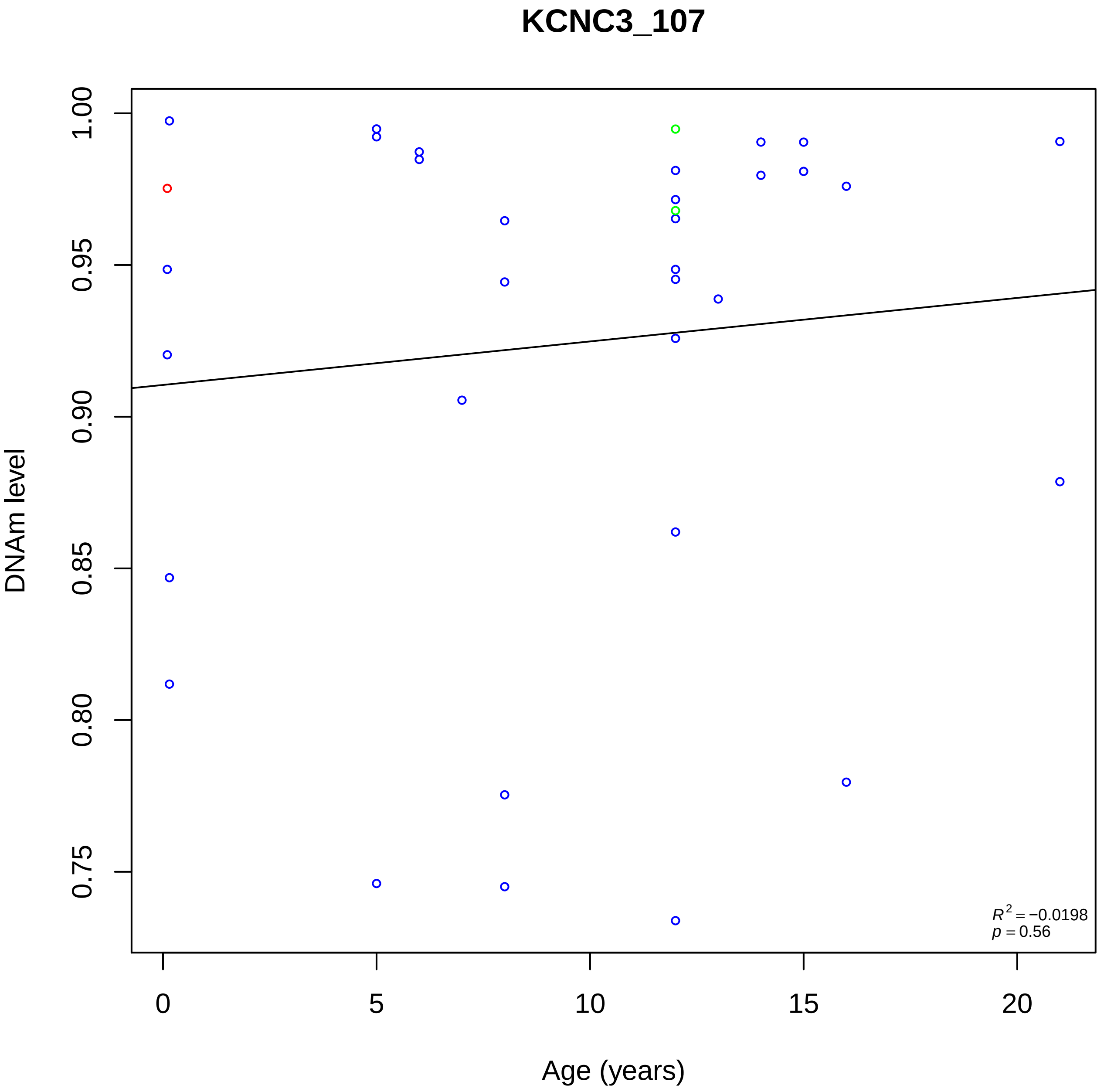

KCNC3_107
1.00
0.95
0.90
0.85
0.80
0.75
DNAm level
0
5
10
15
20
Age (years)
$R^2 = -0.0198$
$p = 0.56$

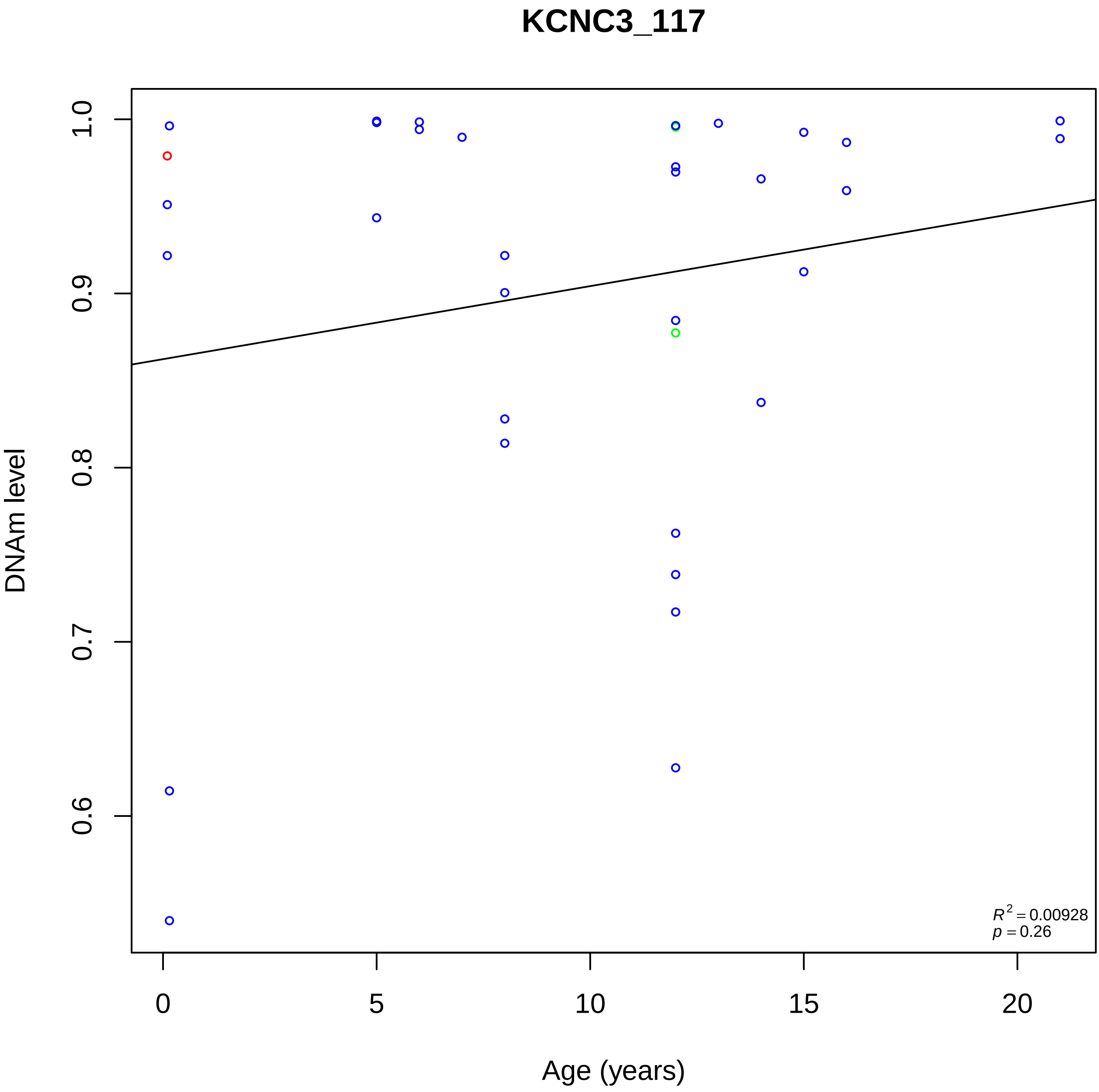

KCNC3_117
DNAm level
1.0
0.9
0.8
0.7
0.6
0
5
10
15
20
Age (years)
$R^2 = 0.00928$
$p = 0.26$

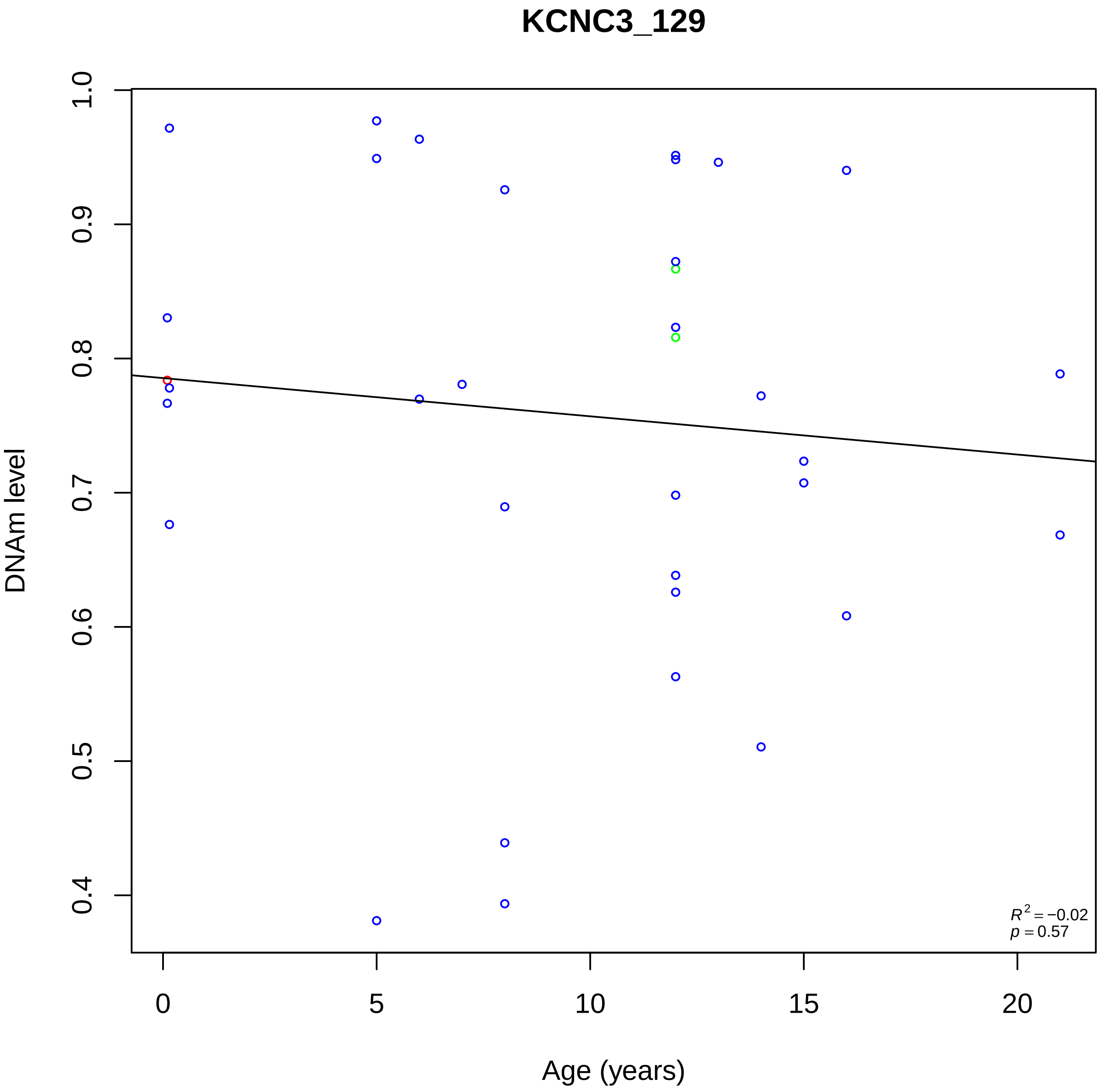
KCNC3_129
DNAm level
Age (years)
1.0
0.9
0.8
0.7
0.6
0.5
0.4
0
5
10
15
20
$R^2 = -0.02$
$p = 0.57$

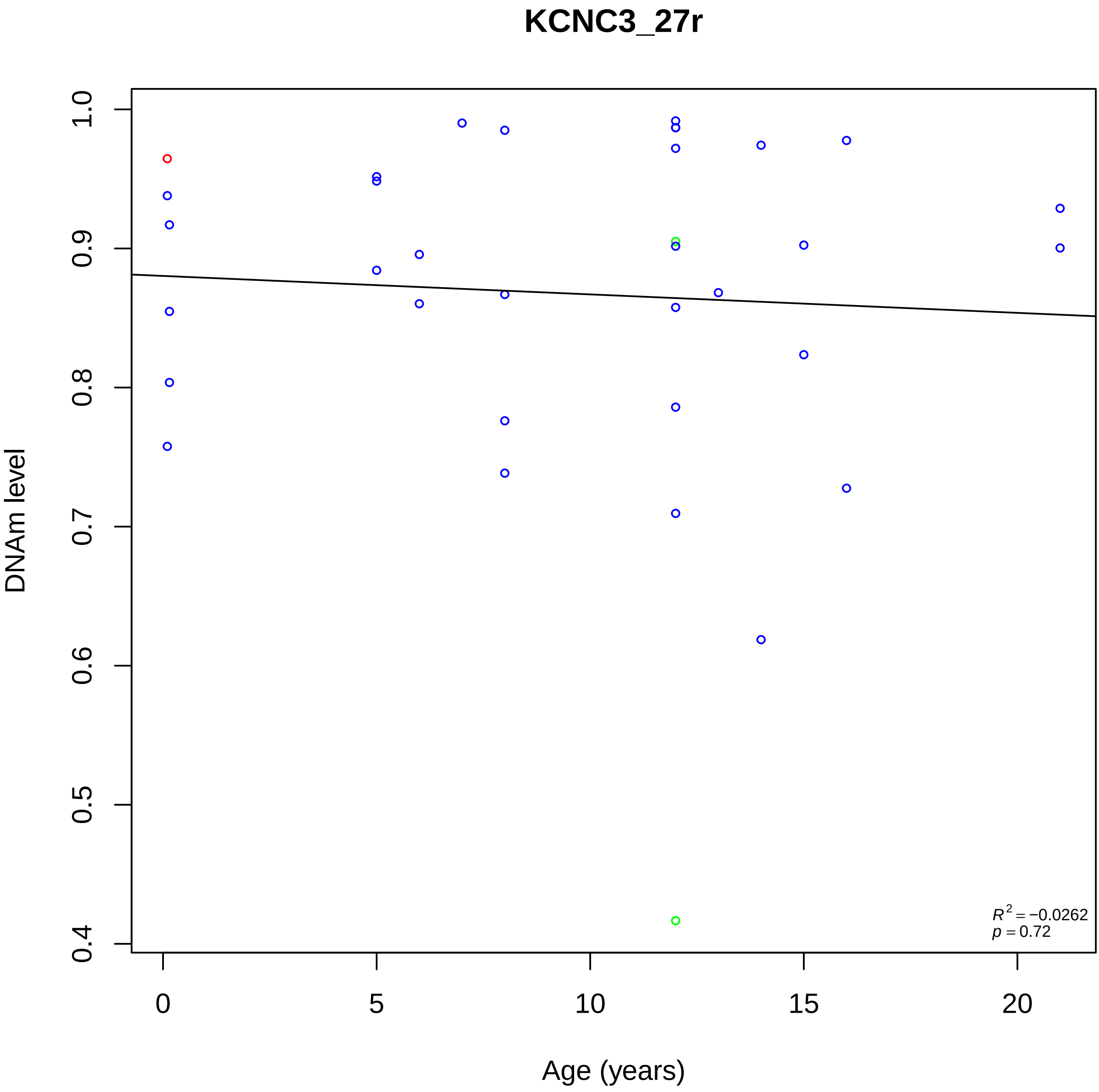
KCNC3_27r
DNAm level
Age (years)
0.4
0.5
0.6
0.7
0.8
0.9
1.0
0
5
10
15
20
$R^2 = -0.0262$
$p = 0.72$

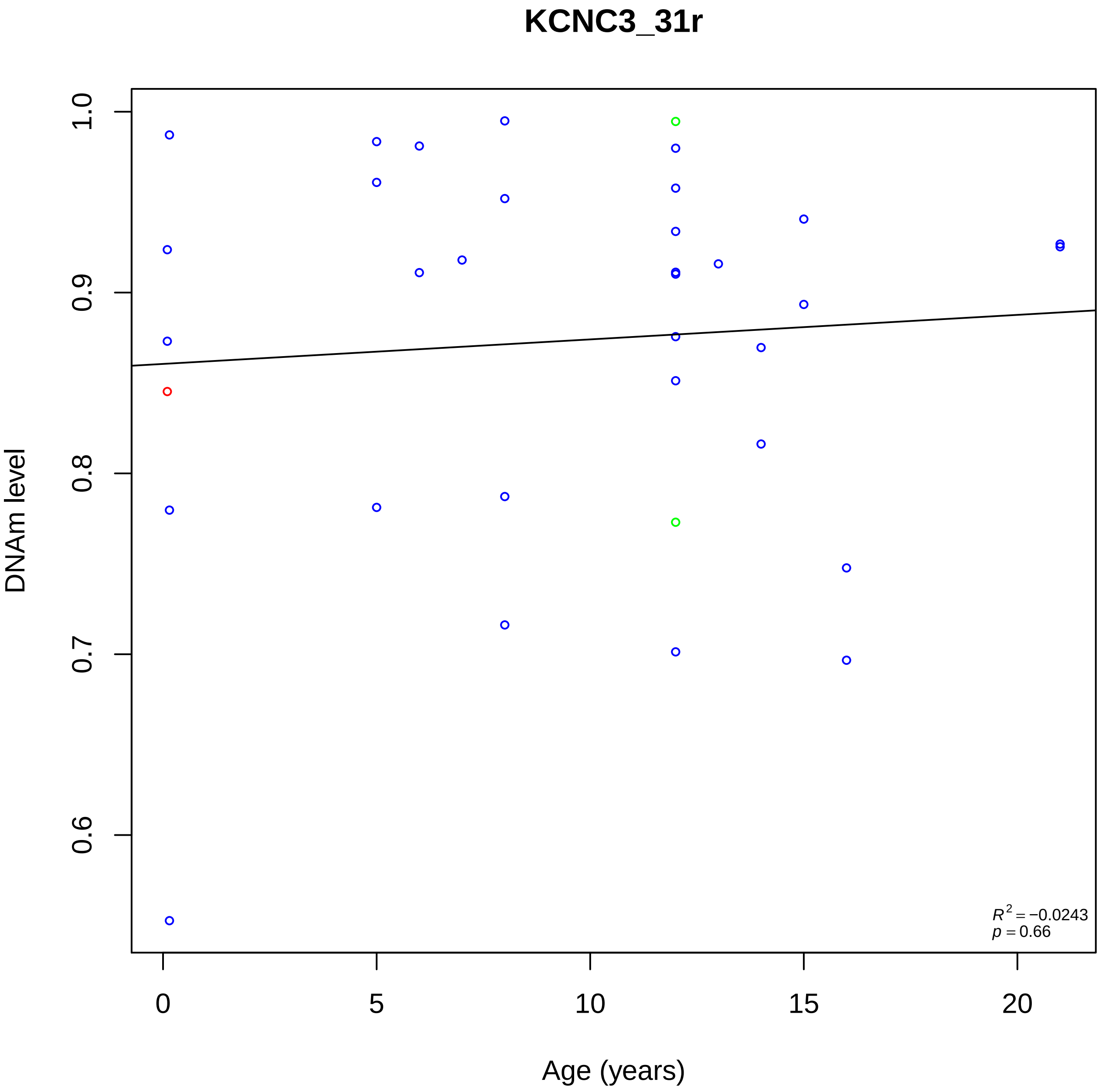

KCNC3_31r
DNAm level
0.6
0.7
0.8
0.9
1.0
Age (years)
0
5
10
15
20
$R^2 = -0.0243$
$p = 0.66$

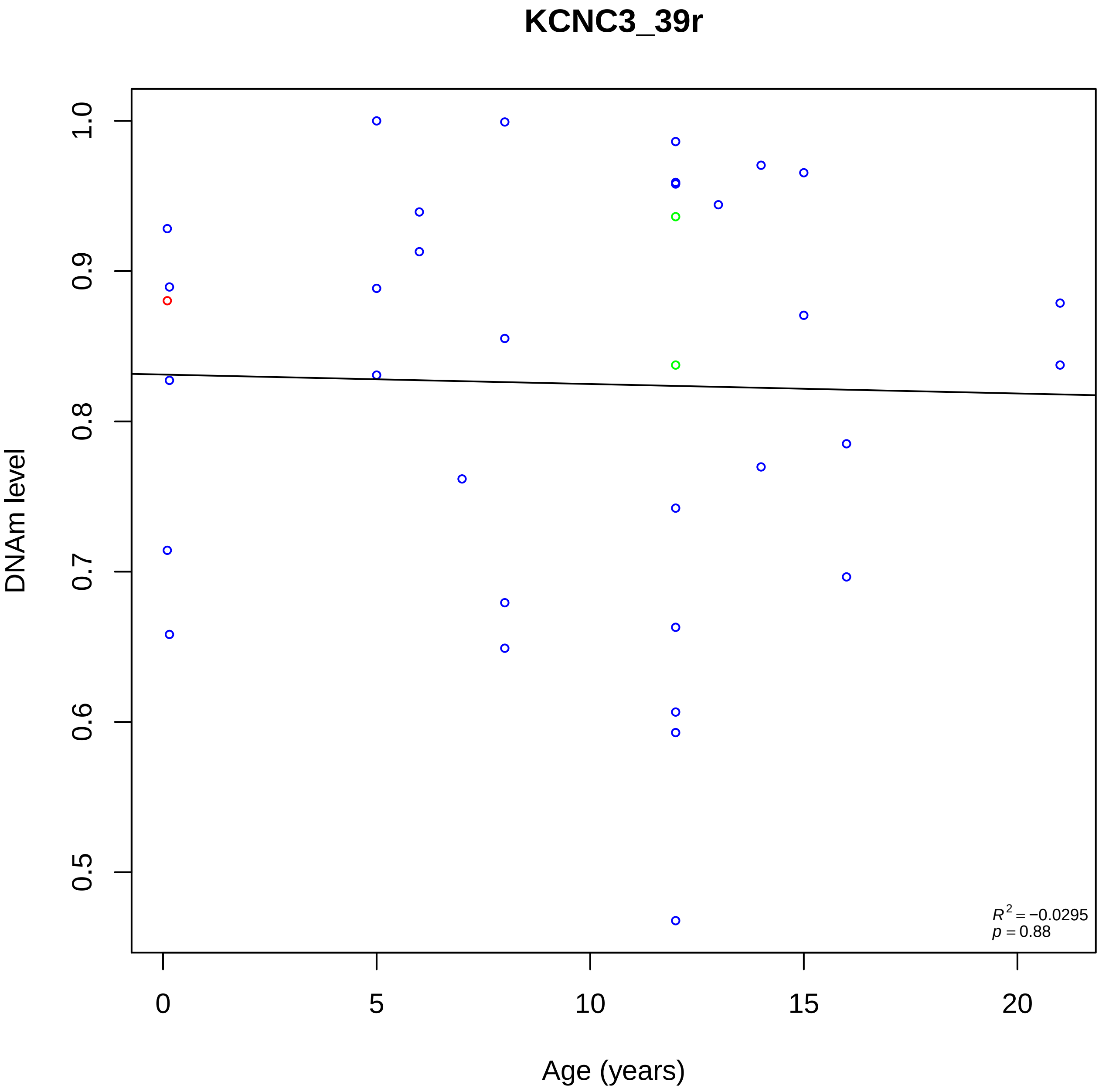

KCNC3_39r
DNAm level
Age (years)
0.5
0.6
0.7
0.8
0.9
1.0
0
5
10
15
20
$R^2 = -0.0295$
$p = 0.88$

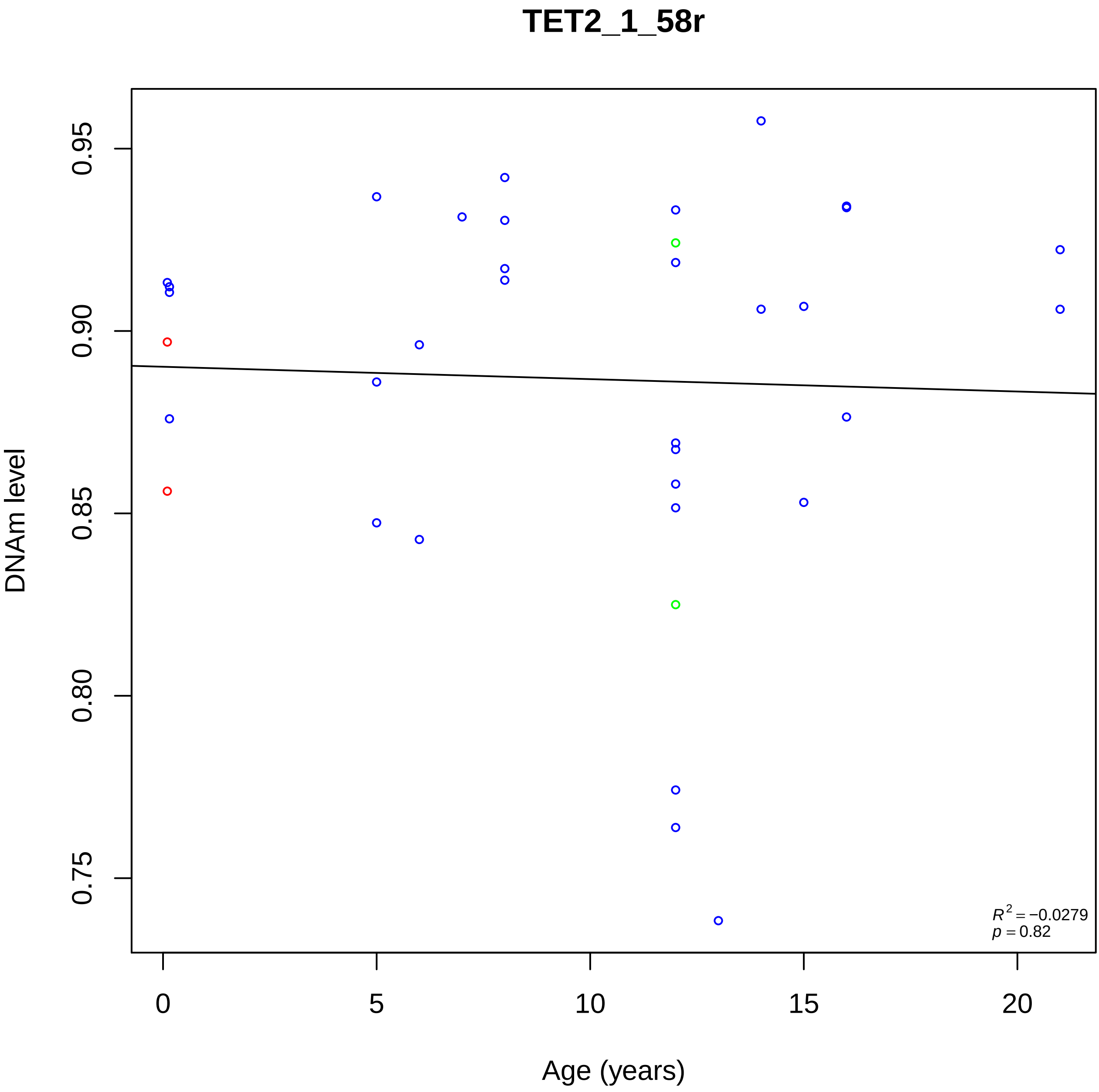
TET2_1_58r
DNAm level
0.75
0.80
0.85
0.90
0.95
Age (years)
0
5
10
15
20
$R^2 = -0.0279$
$p = 0.82$

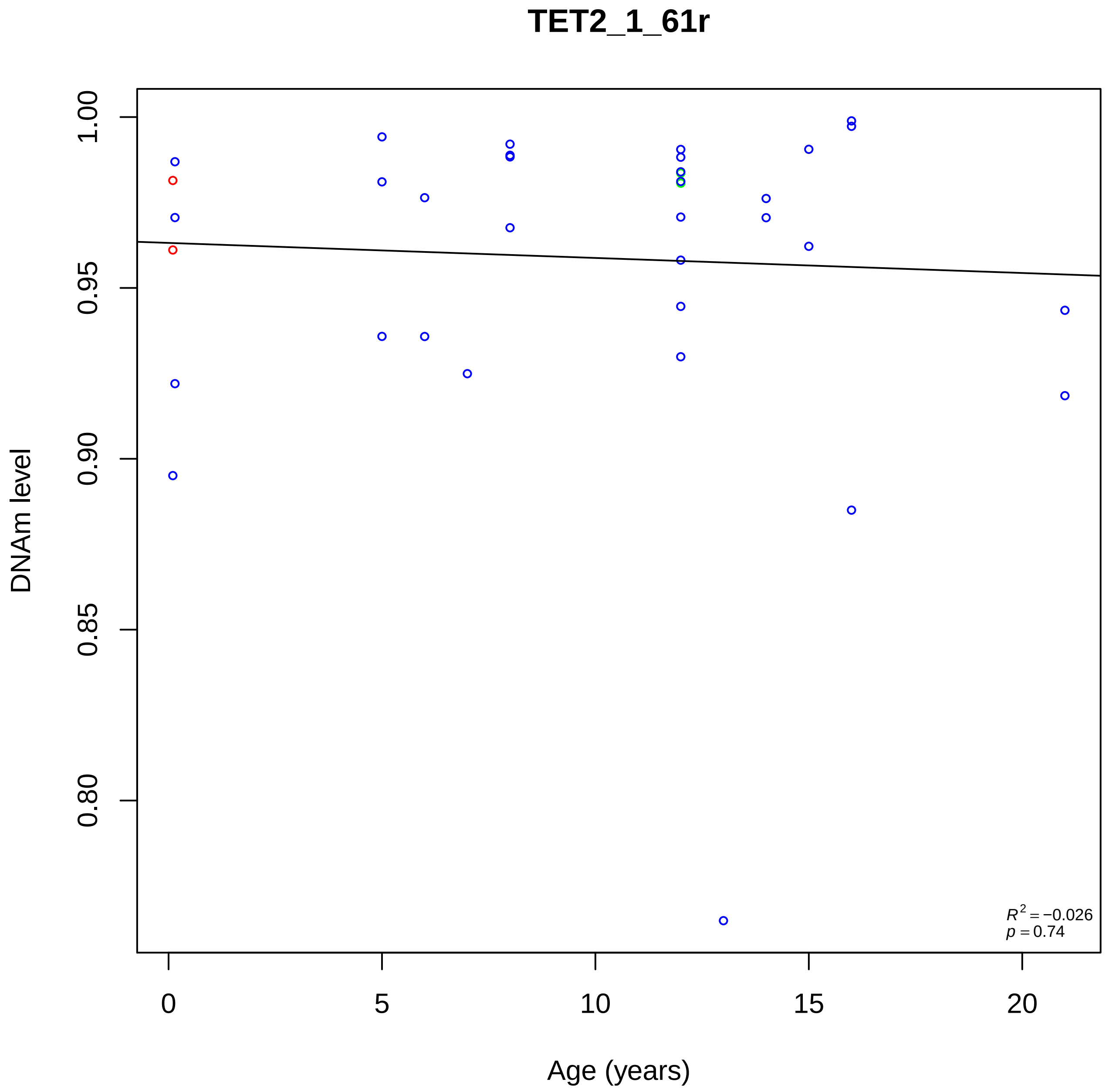

TET2_1_61r
DNAm level
Age (years)
0.80
0.85
0.90
0.95
1.00
0
5
10
15
20
$R^2 = -0.026$
$p = 0.74$

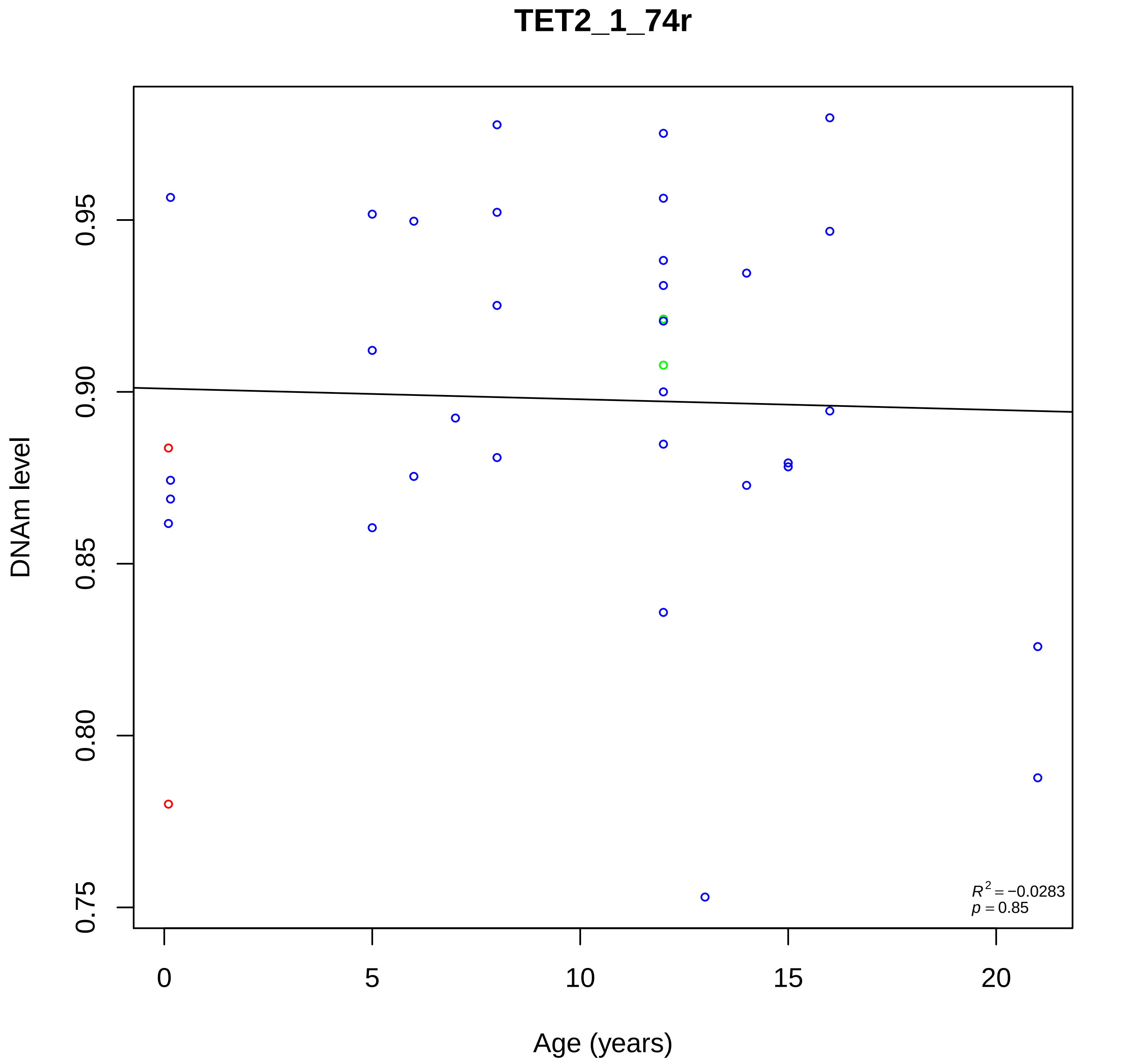
TET2_1_74r
DNAm level
0.75
0.80
0.85
0.90
0.95
0
5
10
15
20
Age (years)
$R^2 = -0.0283$
$p = 0.85$

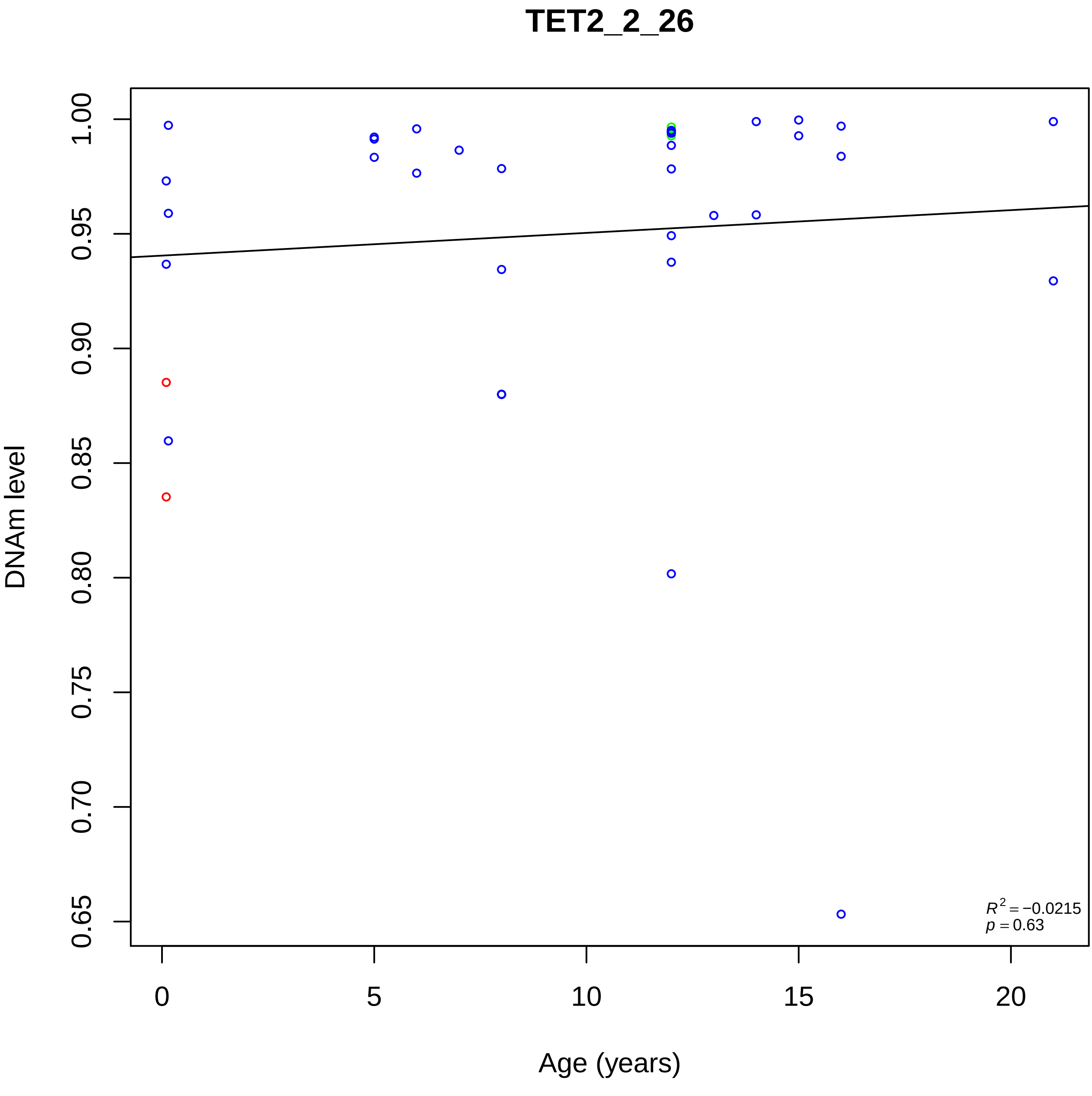

TET2_2_26
DNAm level
Age (years)
1.00
0.95
0.90
0.85
0.80
0.75
0.70
0.65
0
5
10
15
20
$R^2 = -0.0215$
$p = 0.63$

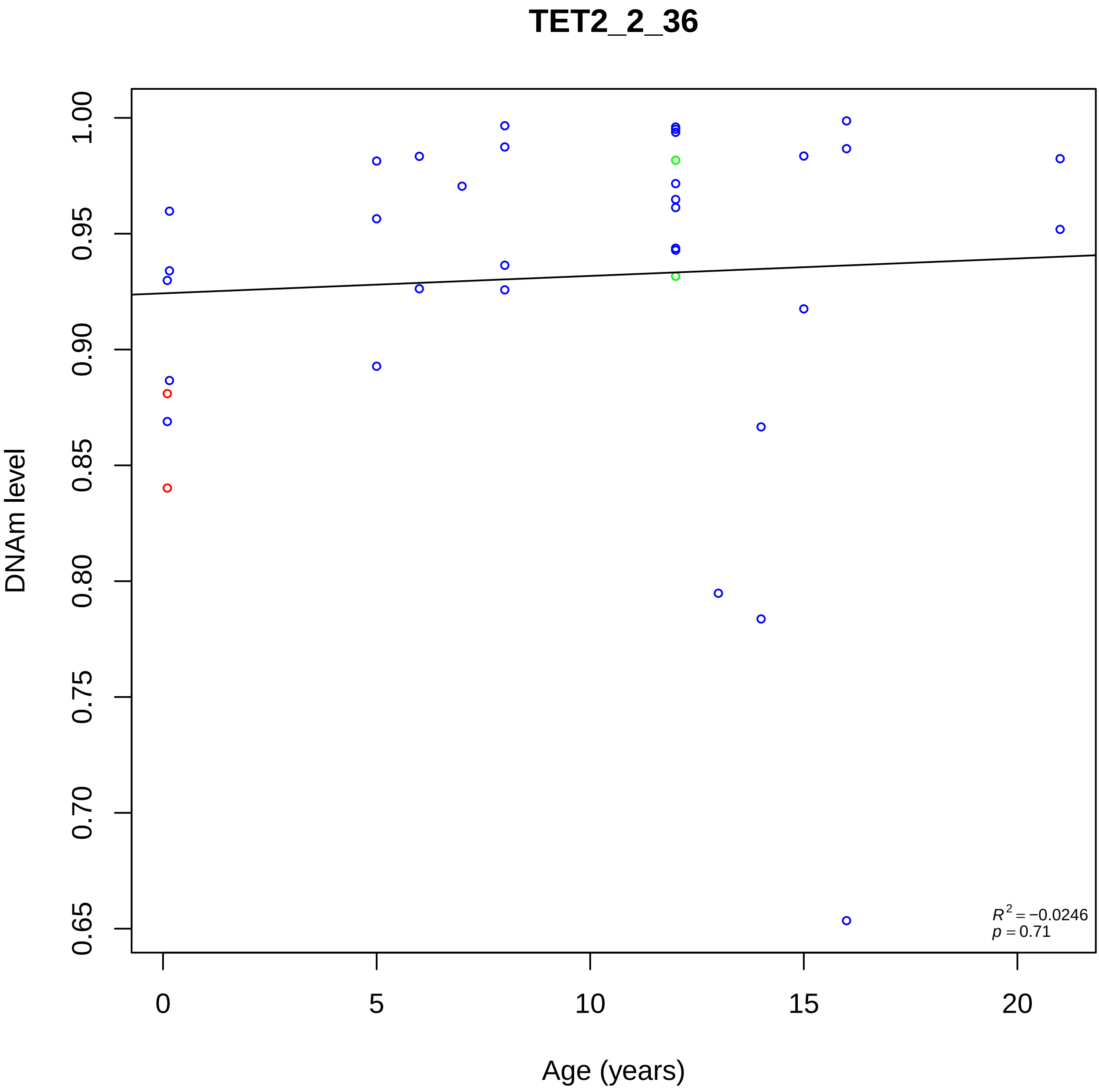

TET2_2_36
DNAm level
Age (years)
0.65
0.70
0.75
0.80
0.85
0.90
0.95
1.00
0
5
10
15
20
$R^2 = -0.0246$
$p = 0.71$

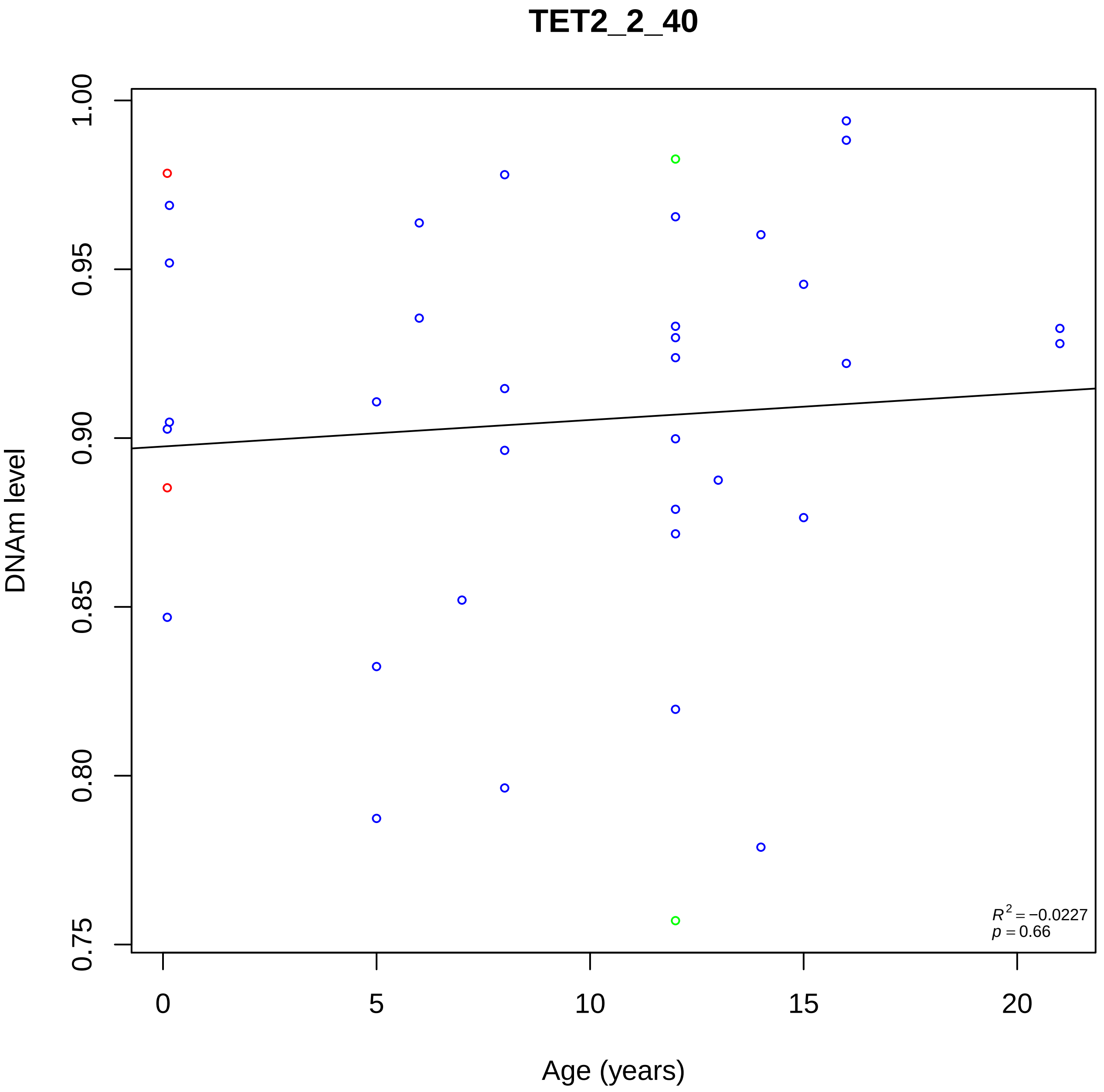
TET2_2_40
DNAm level
1.00
0.95
0.90
0.85
0.80
0.75
0
5
10
15
20
Age (years)
$R^2 = -0.0227$
$p = 0.66$

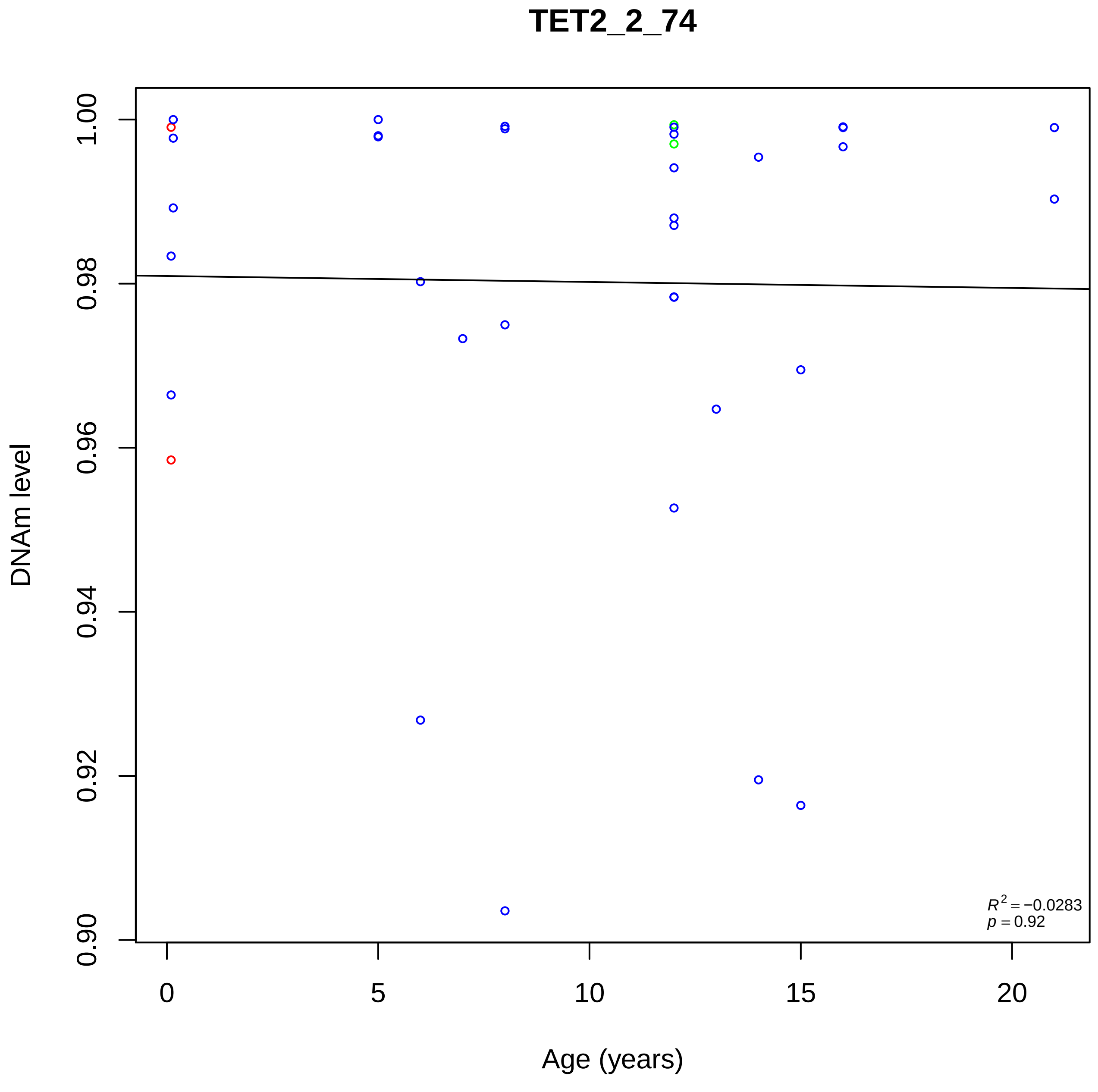

TET2_2_74
DNAm level
1.00
0.98
0.96
0.94
0.92
0.90
0
5
10
15
20
Age (years)
R^2 = −0.0283
p = 0.92

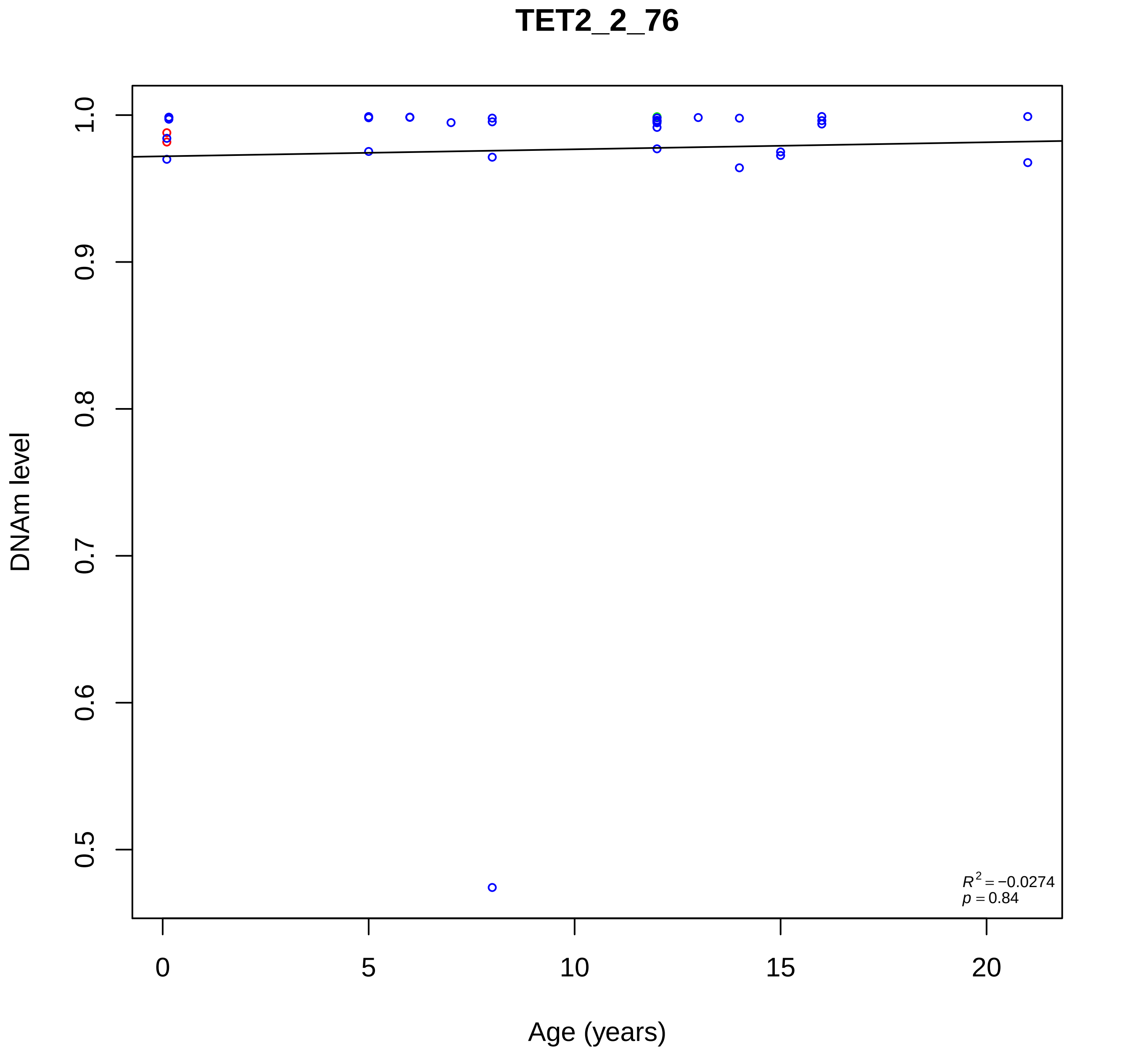

TET2_2_76
DNAm level
1.0
0.9
0.8
0.7
0.6
0.5
0
5
10
15
20
Age (years)
$R^2 = -0.0274$
$p = 0.84$

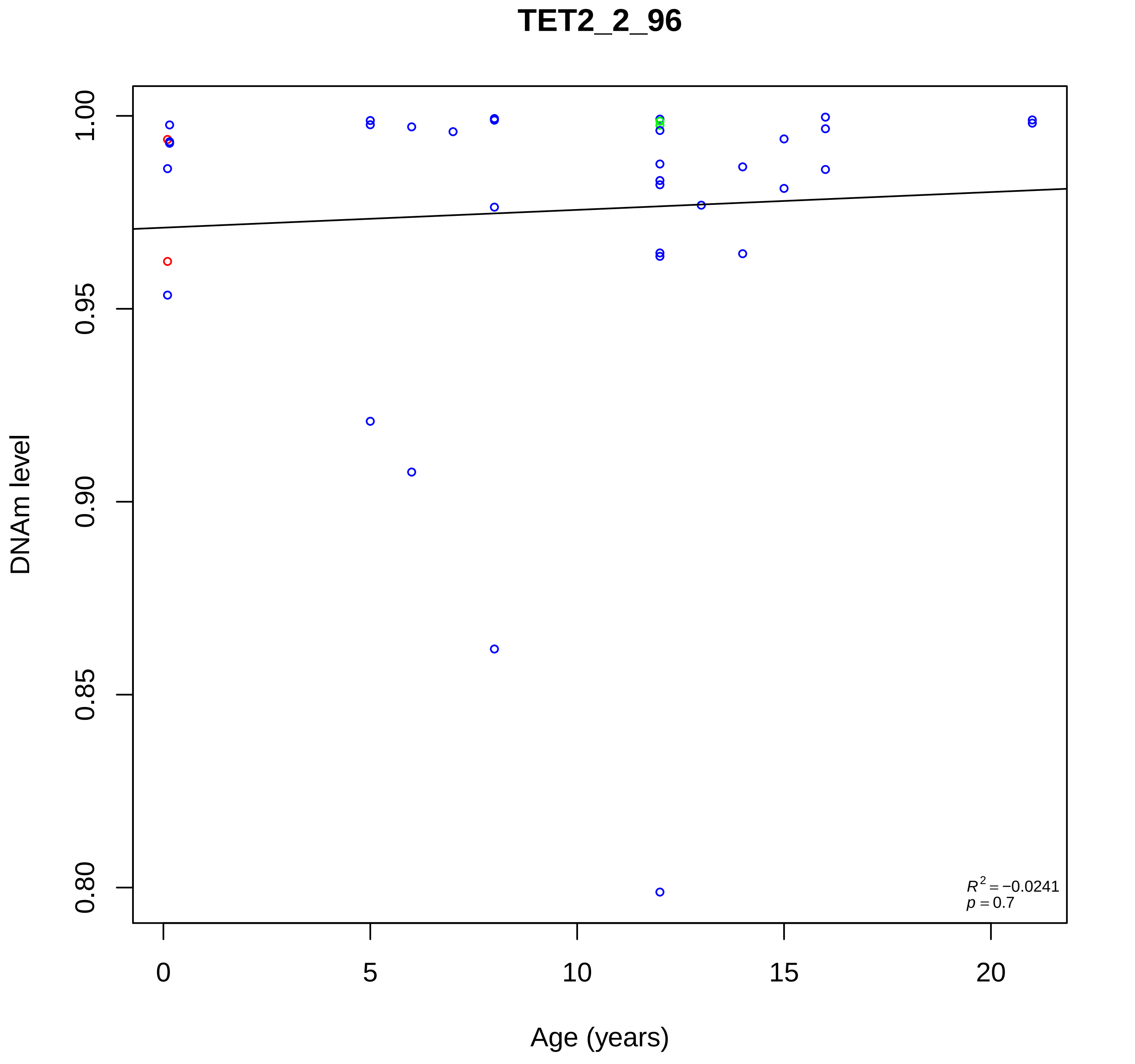

TET2_2_96
1.00
0.95
0.90
0.85
0.80
DNAm level
0
5
10
15
20
Age (years)
$R^2 = -0.0241$
$p = 0.7$
