# Supplementary material for: DNA methylation levels in candidate genes associated with chronological age in mammals are not conserved in a long-lived seabird
Source: PLoS One. 2017 Dec 7;12(12):e0189181. doi: 10.1371/journal.pone.0189181 (PMC5720723; doi:10.1371/journal.pone.0189181)
Supplement: S4 File — (PDF) [file pone.0189181.s009.pdf]

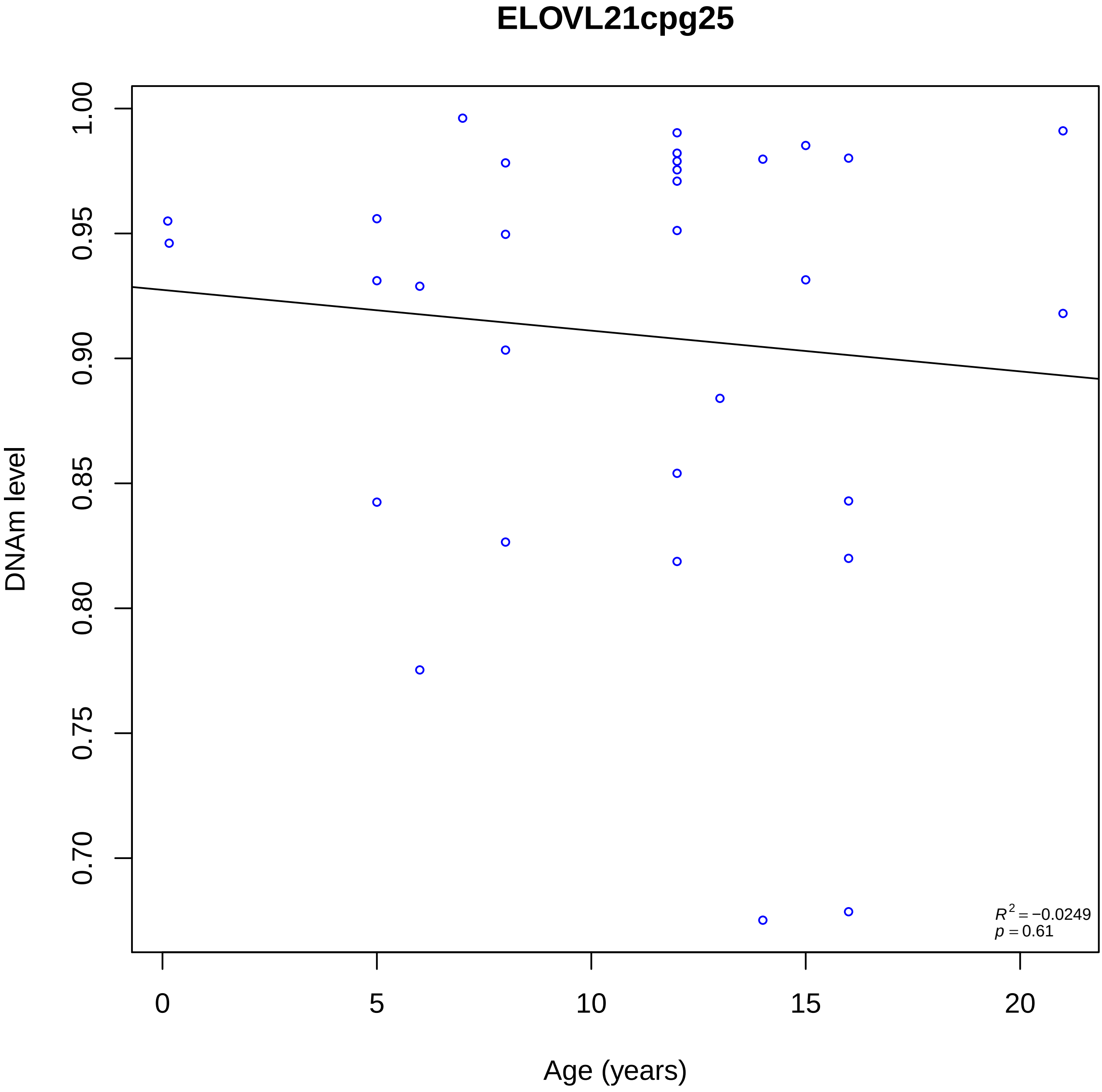

ELOVL21cpg25
DNAm level
Age (years)
1.00
0.95
0.90
0.85
0.80
0.75
0.70
0
5
10
15
20
$R^2 = -0.0249$
$p = 0.61$

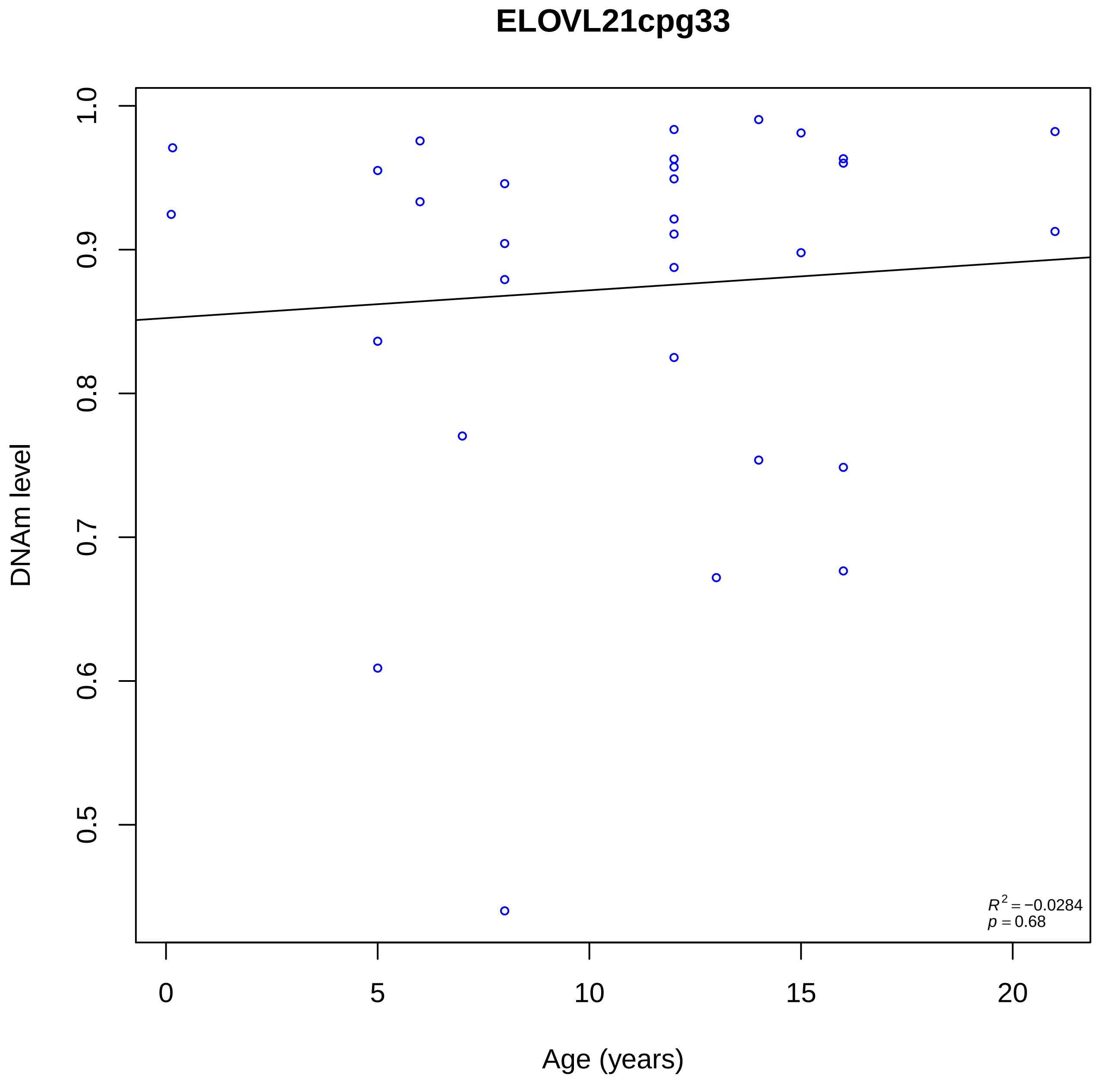
ELOVL21cpg33
DNAm level
Age (years)
0
5
10
15
20
0.5
0.6
0.7
0.8
0.9
1.0
$R^2 = -0.0284$
$p = 0.68$

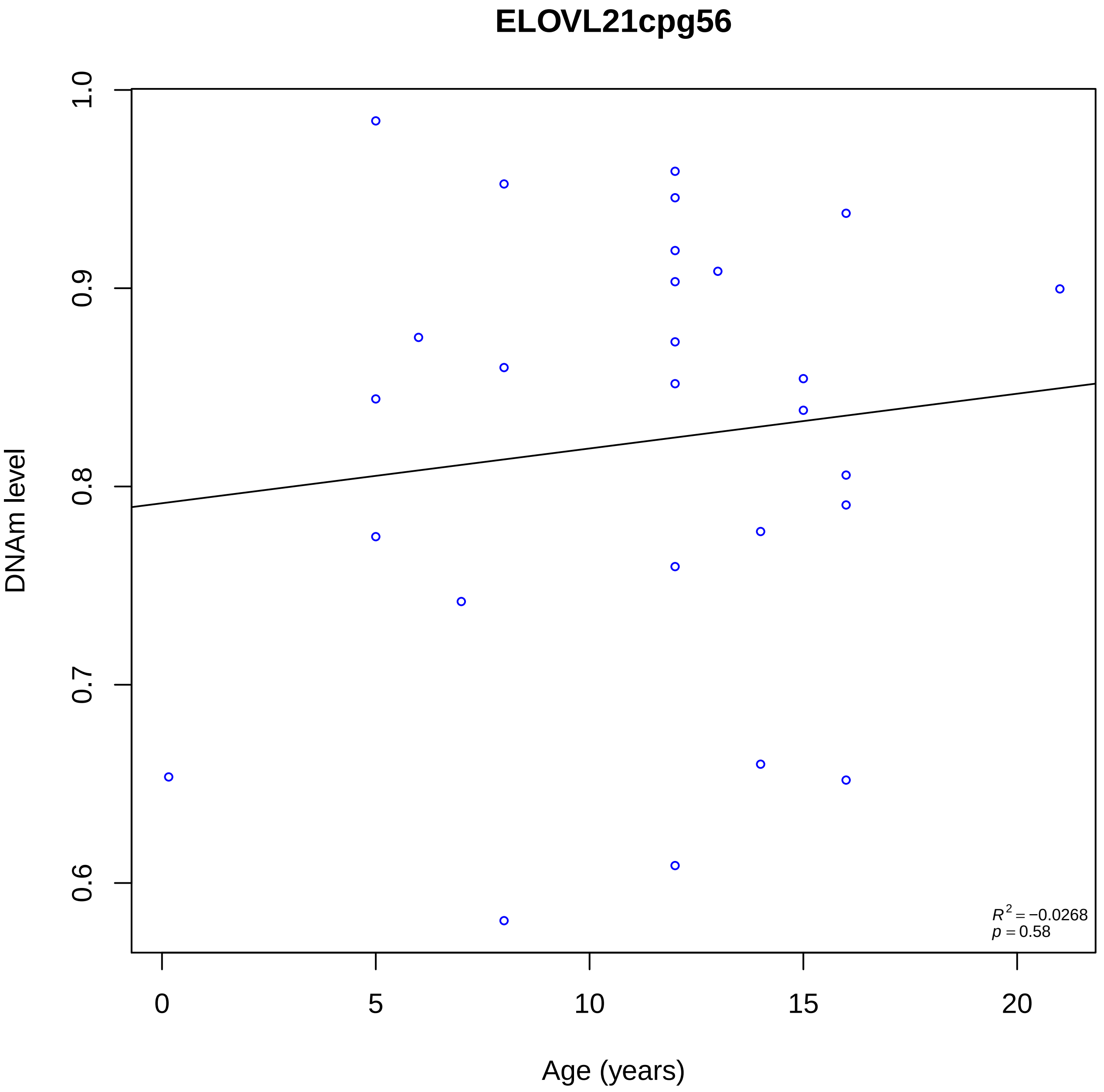
ELOVL21cpg56
DNAm level
1.0
0.9
0.8
0.7
0.6
0
5
10
15
20
Age (years)
$R^2 = -0.0268$
$p = 0.58$

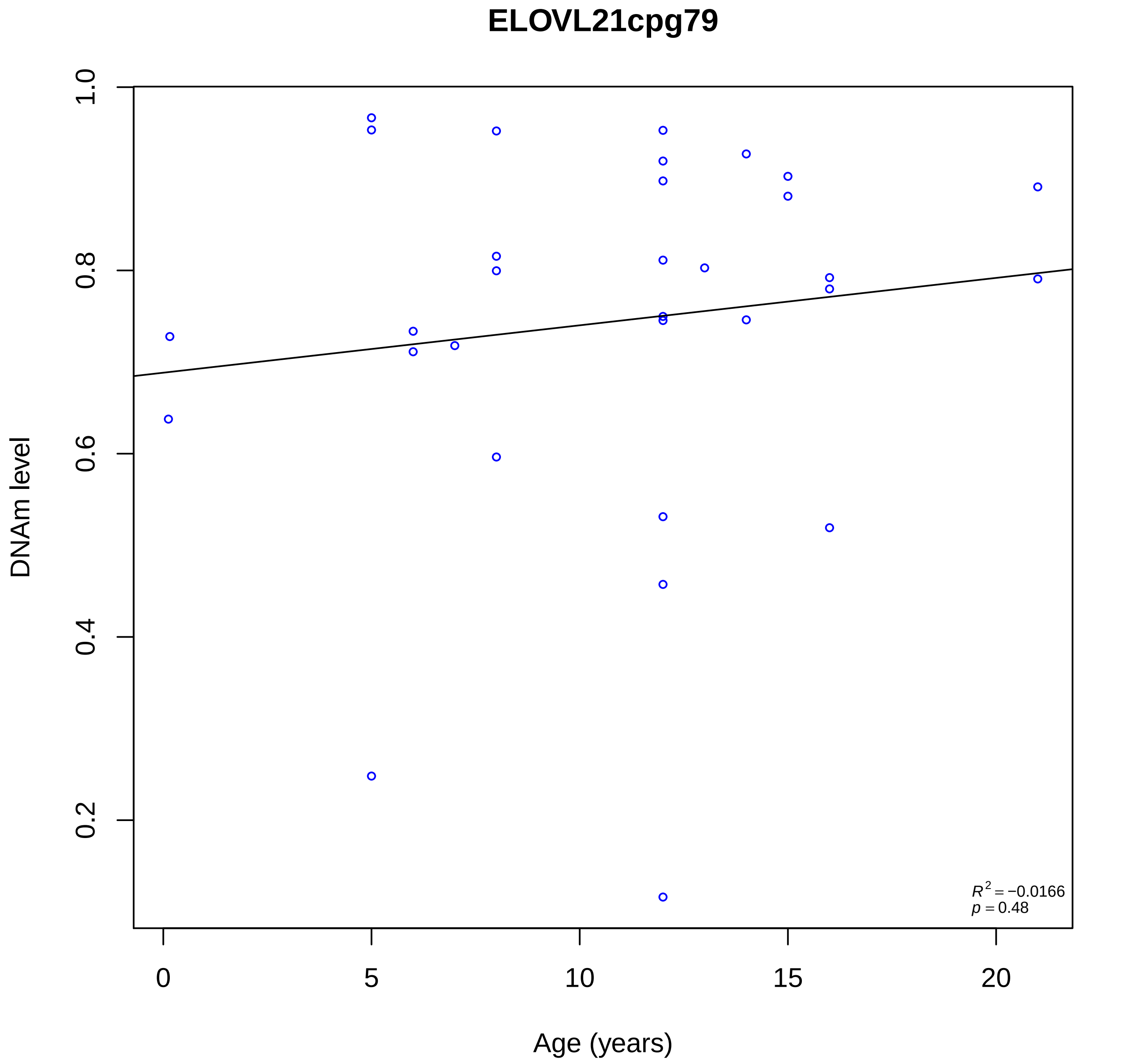
ELOVL21cpg79
DNAm level
1.0
0.8
0.6
0.4
0.2
0
5
10
15
20
Age (years)
$R^2 = -0.0166$
$p = 0.48$

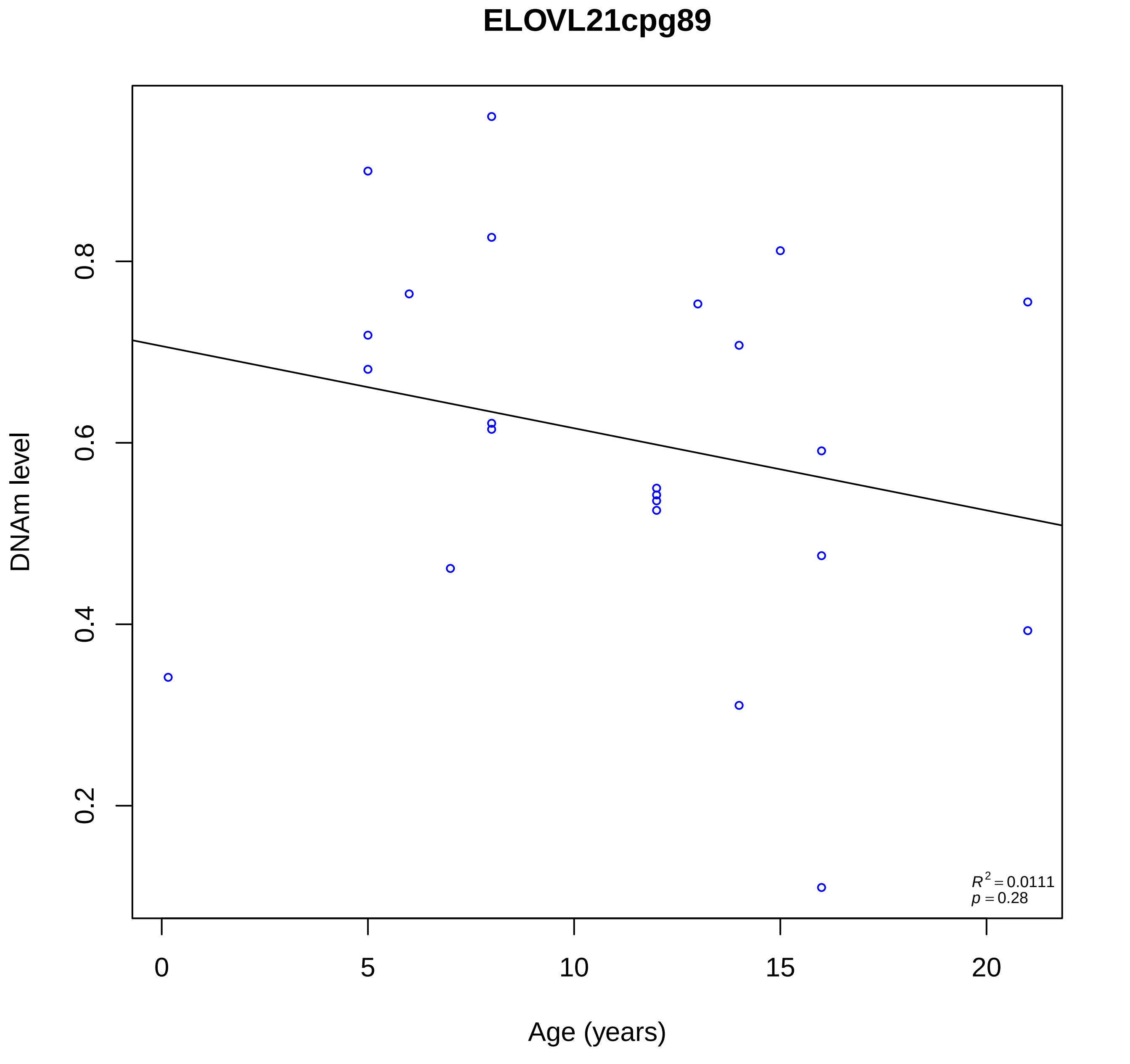

ELOVL21cpg89
DNAm level
0.2
0.4
0.6
0.8
Age (years)
0
5
10
15
20
$R^2 = 0.0111$
$p = 0.28$

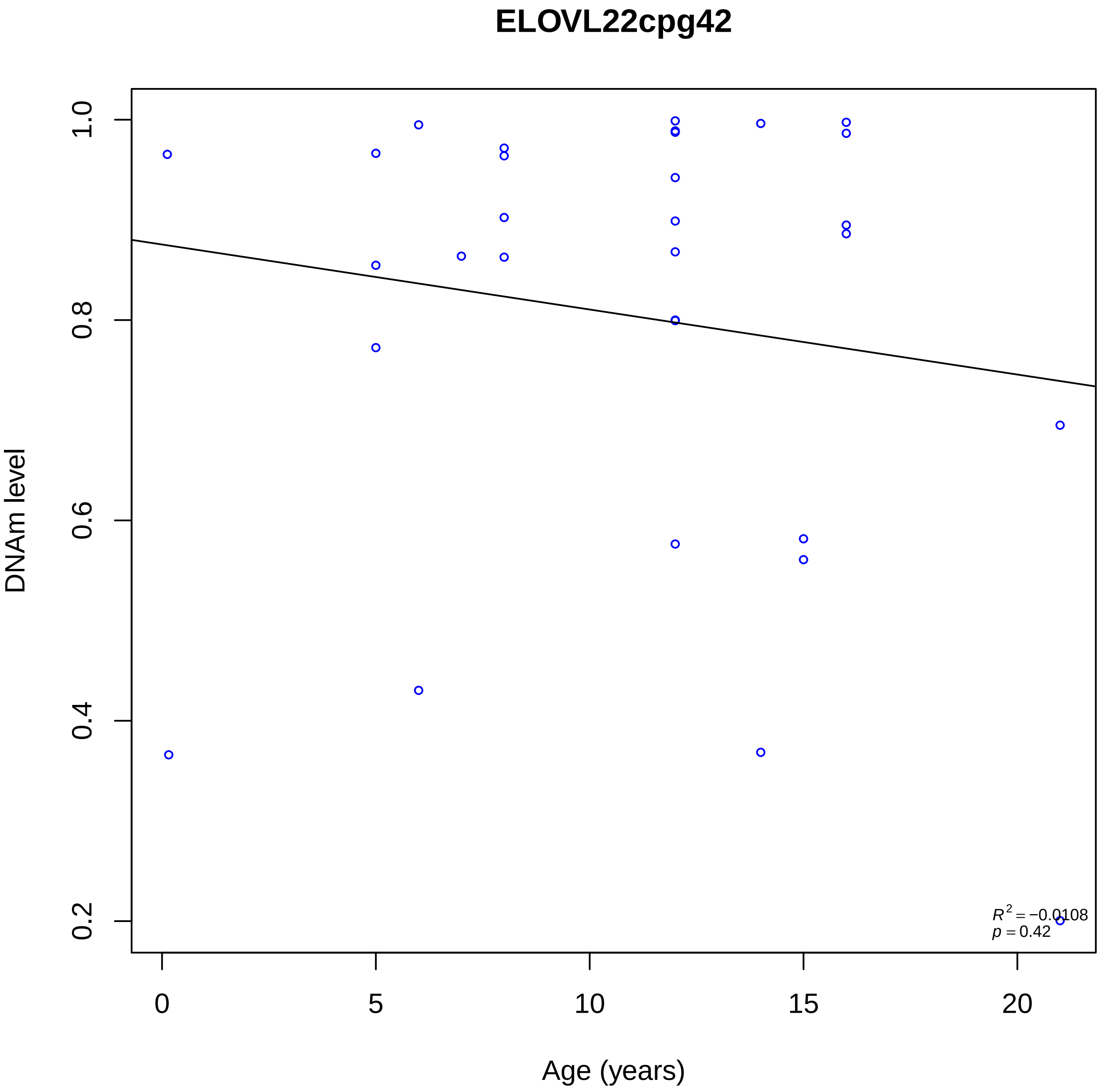
ELOVL22cpg42
DNAm level
1.0
0.8
0.6
0.4
0.2
0
5
10
15
20
Age (years)
$R^2 = -0.0108$
$p = 0.42$

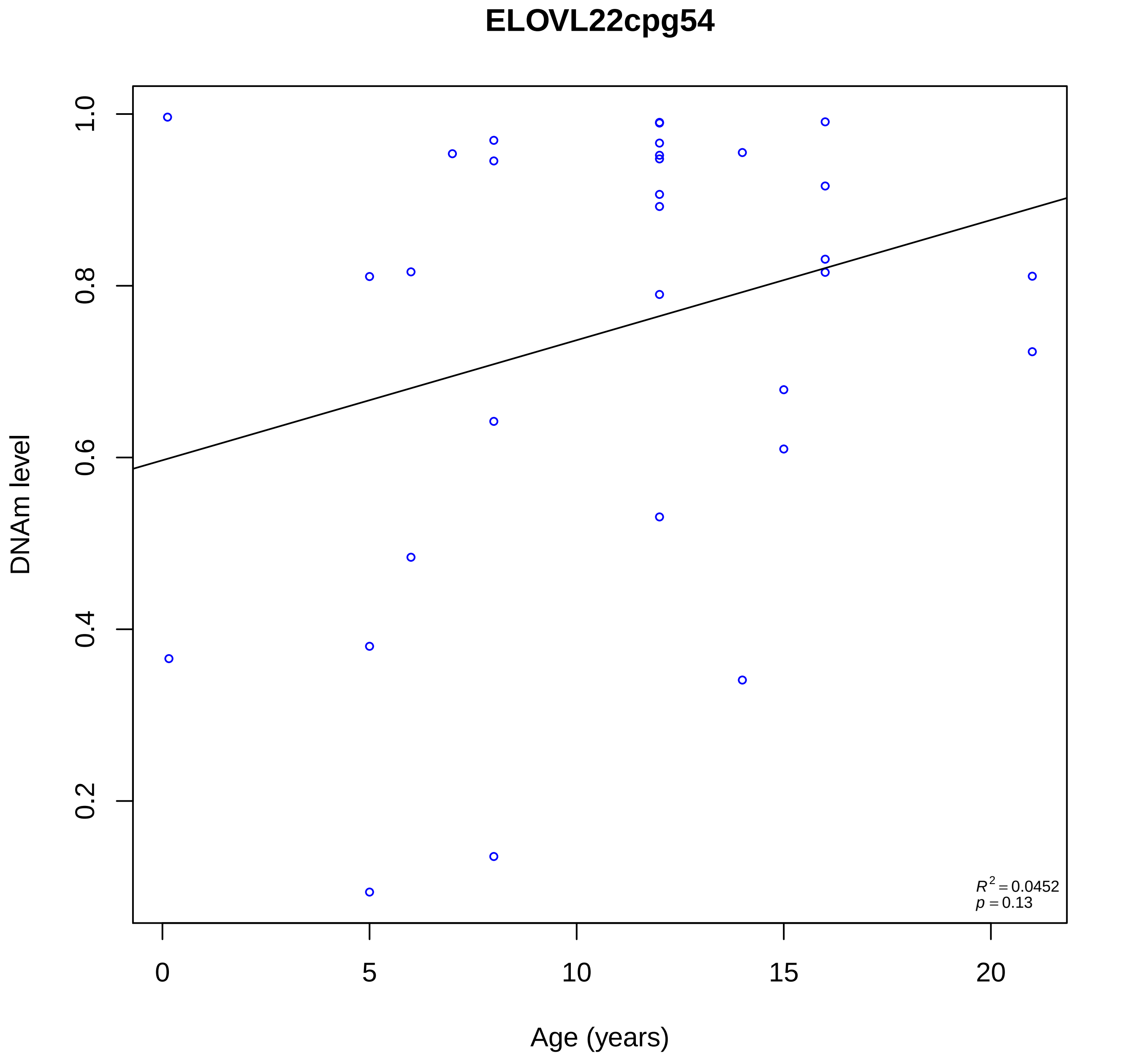

ELOVL22cpg54
DNAm level
0.2
0.4
0.6
0.8
1.0
0
5
10
15
20
Age (years)
$R^2 = 0.0452$
$p = 0.13$

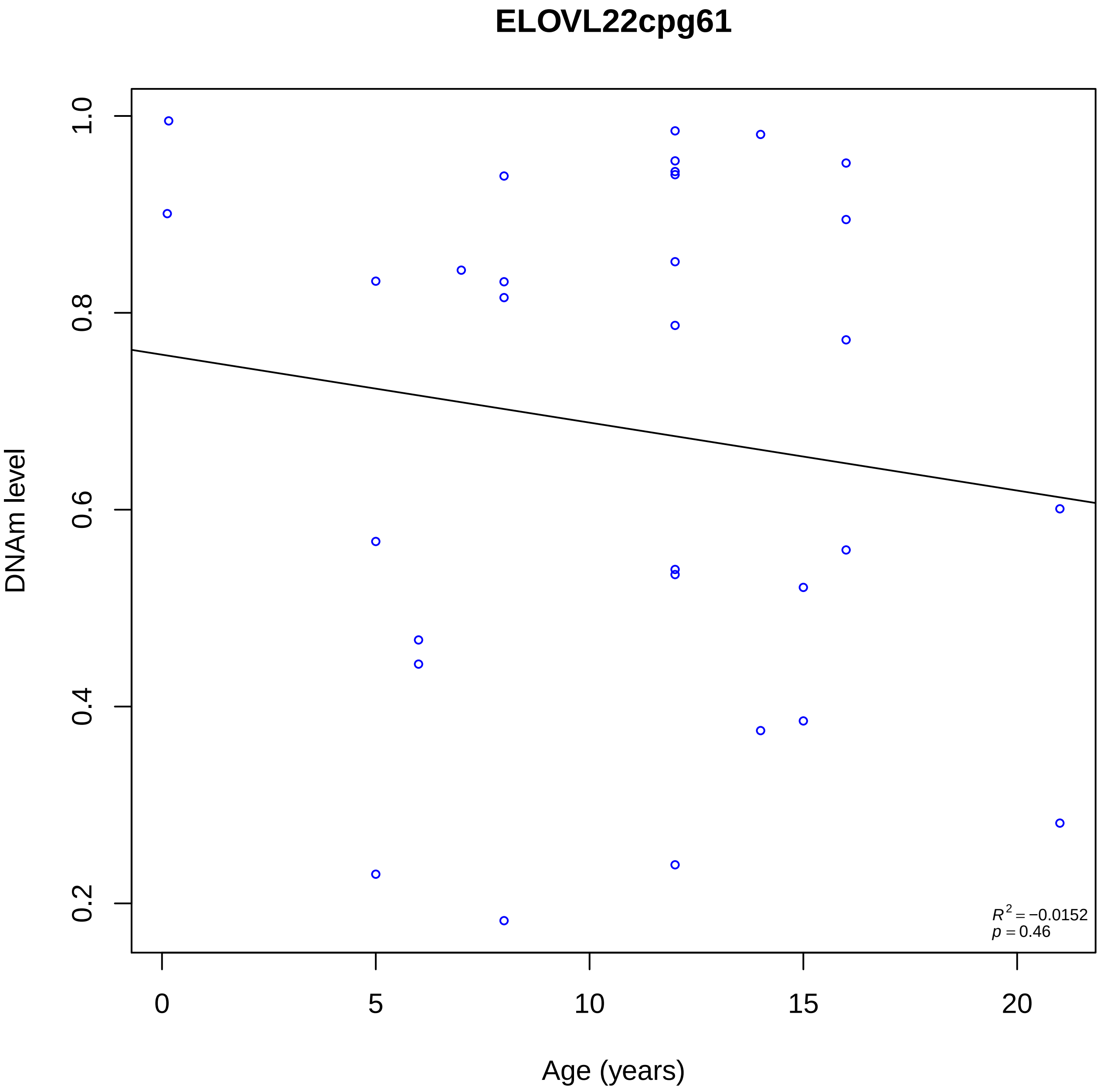
ELOVL22cpg61
DNAm level
Age (years)
1.0
0.8
0.6
0.4
0.2
0
5
10
15
20
$R^2 = -0.0152$
$p = 0.46$

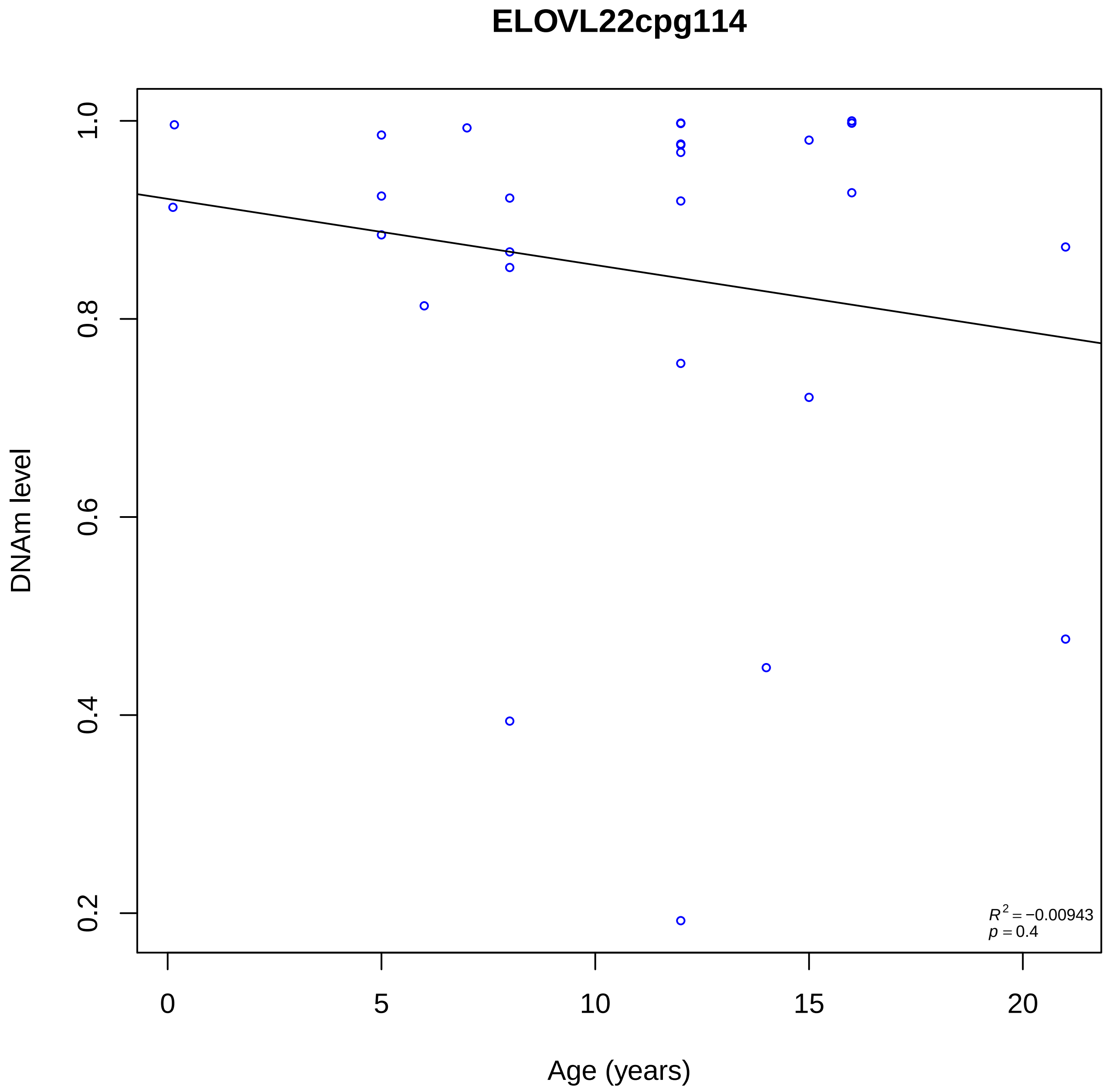
ELOVL22cpg114
DNAm level
Age (years)
0.2
0.4
0.6
0.8
1.0
0
5
10
15
20
$R^2 = -0.00943$
$p = 0.4$

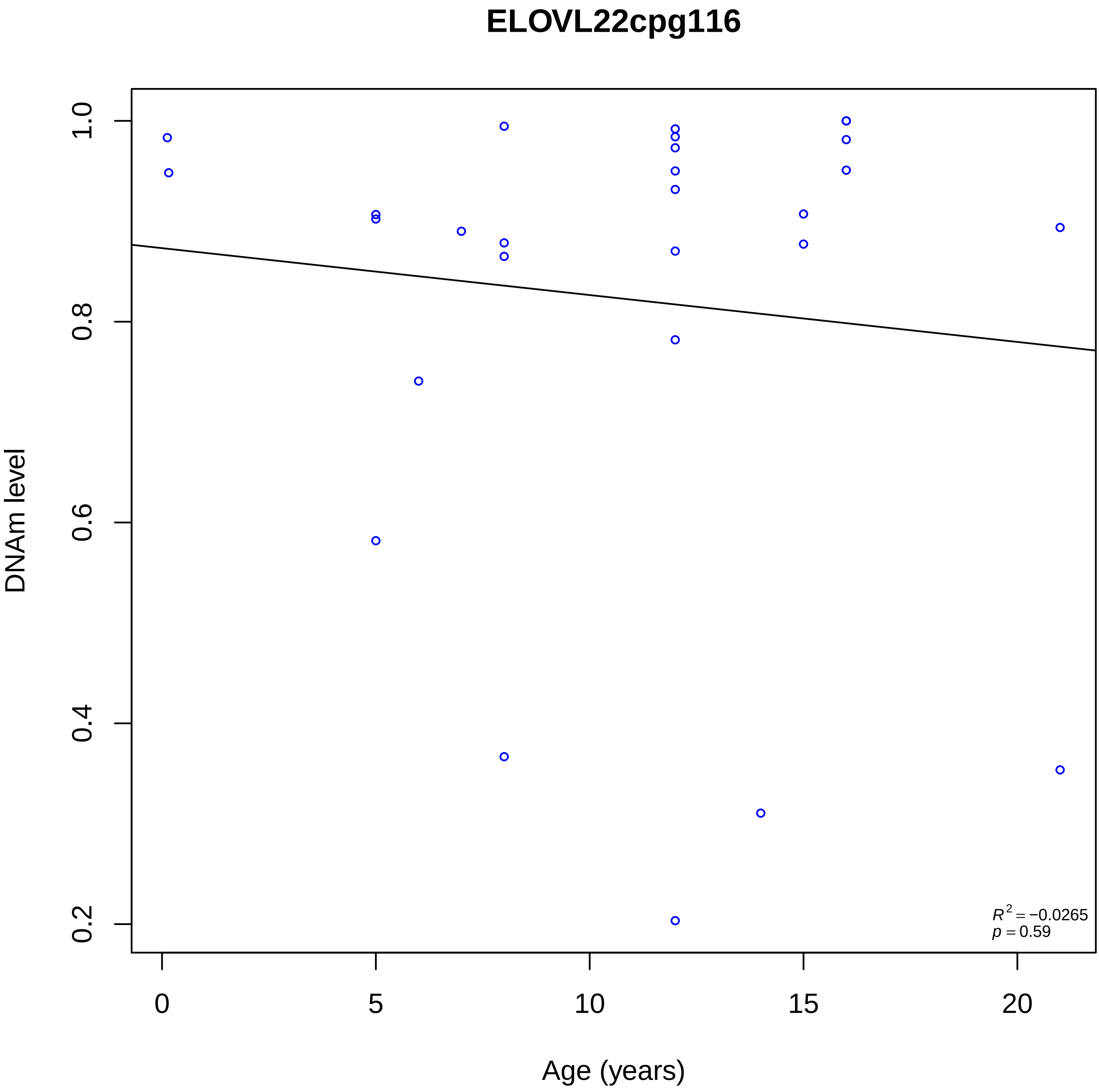
ELOVL22cpg116
DNAm level
1.0
0.8
0.6
0.4
0.2
0
5
10
15
20
Age (years)
$R^2 = -0.0265$
$p = 0.59$

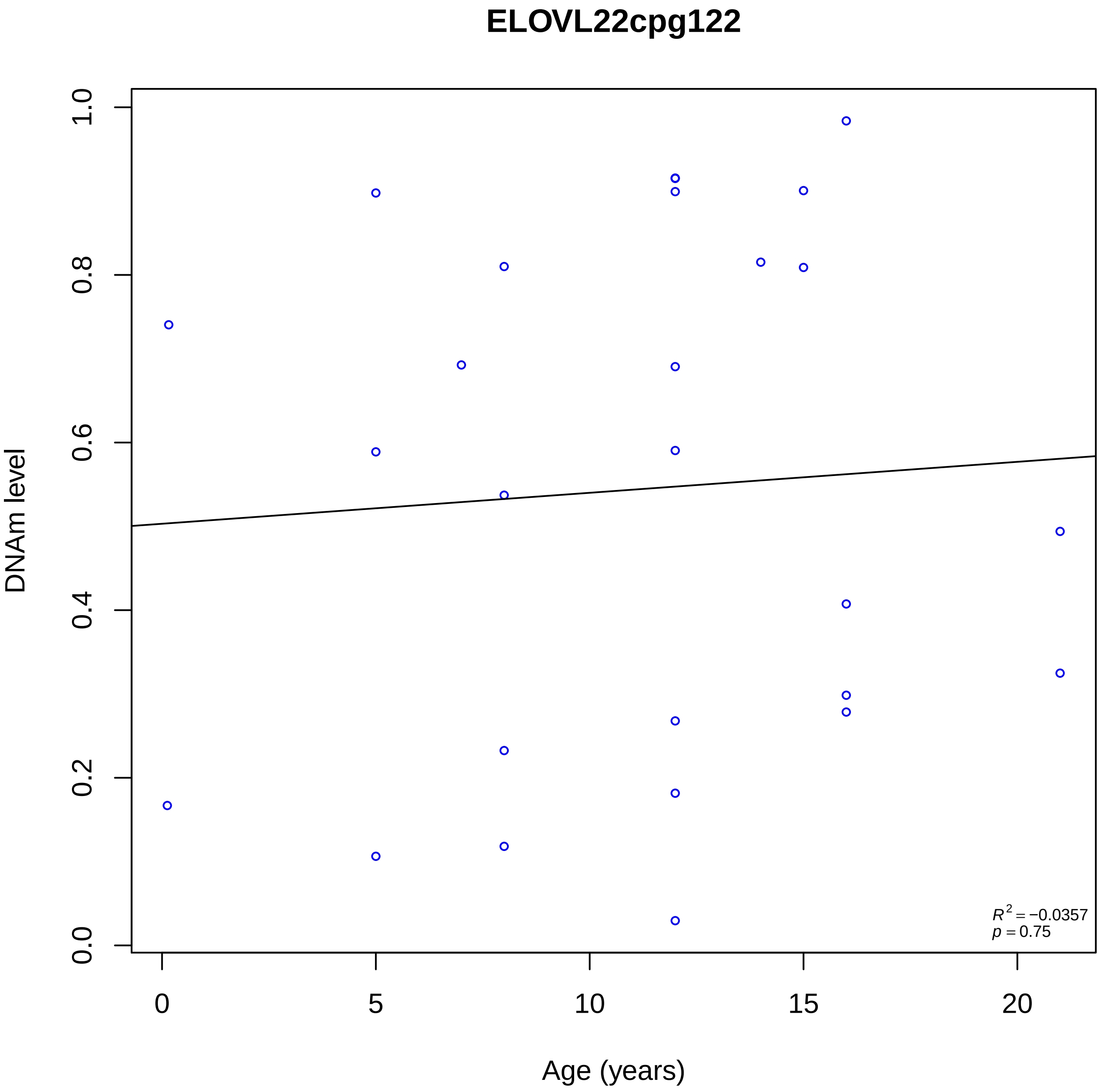

ELOVL22cpg122
DNAm level
Age (years)
0.0
0.2
0.4
0.6
0.8
1.0
0
5
10
15
20
$R^2 = -0.0357$
$p = 0.75$

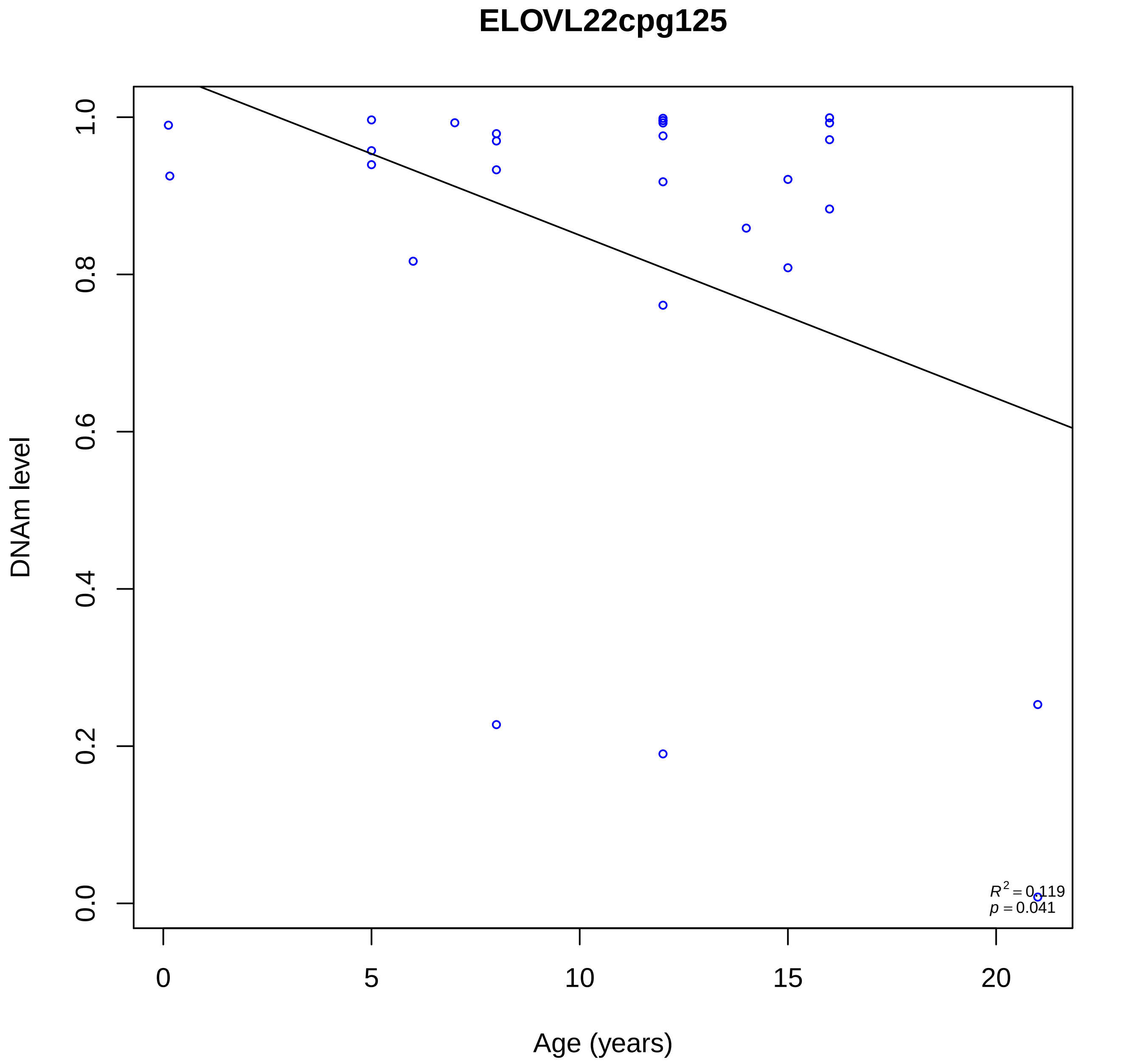
ELOVL22cpg125
DNAm level
Age (years)
0.0
0.2
0.4
0.6
0.8
1.0
0
5
10
15
20
$R^2 = 0.119$
$p = 0.041$

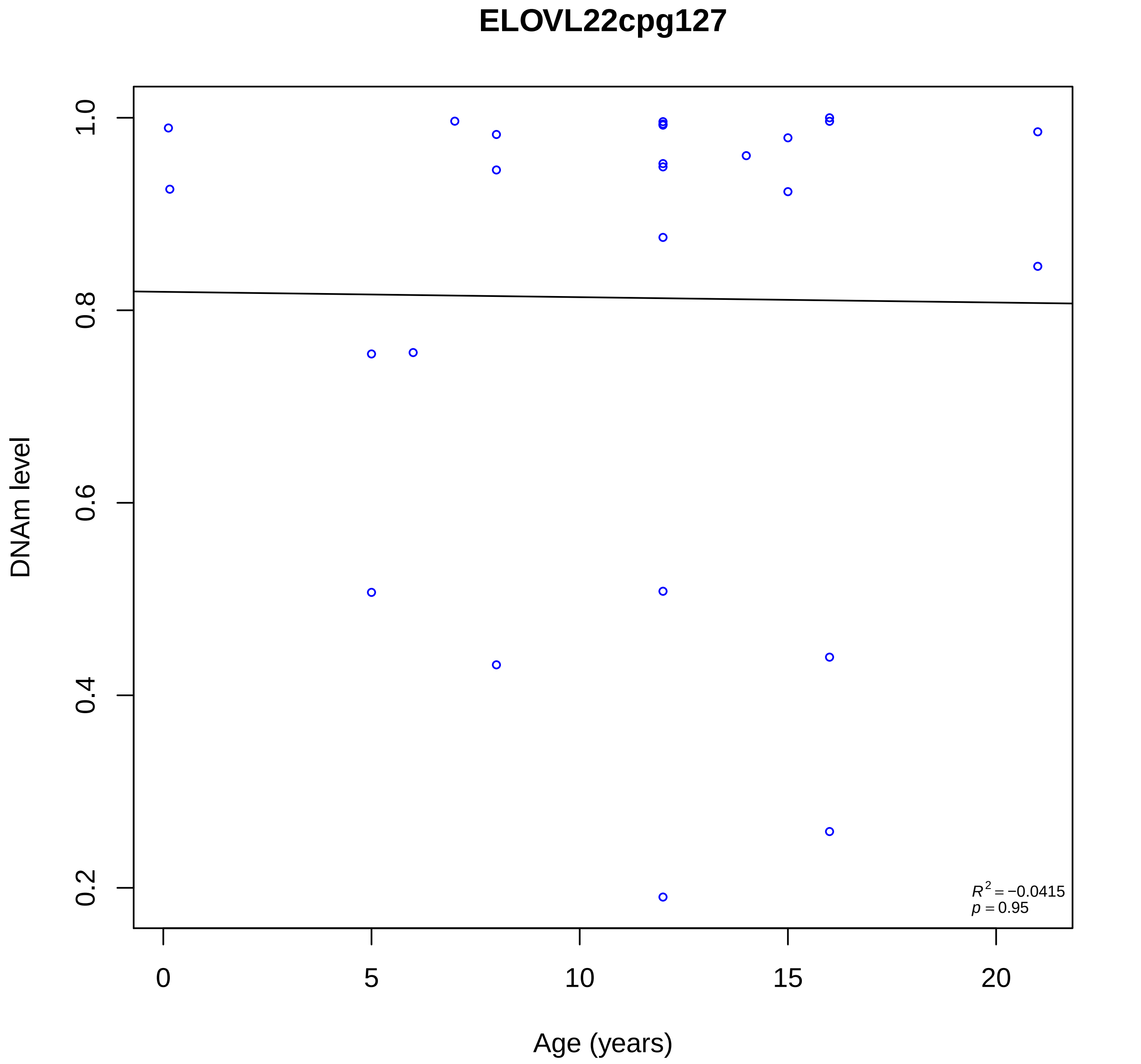
ELOVL22cpg127
DNAm level
1.0
0.8
0.6
0.4
0.2
0
5
10
15
20
Age (years)
$R^2 = -0.0415$
$p = 0.95$

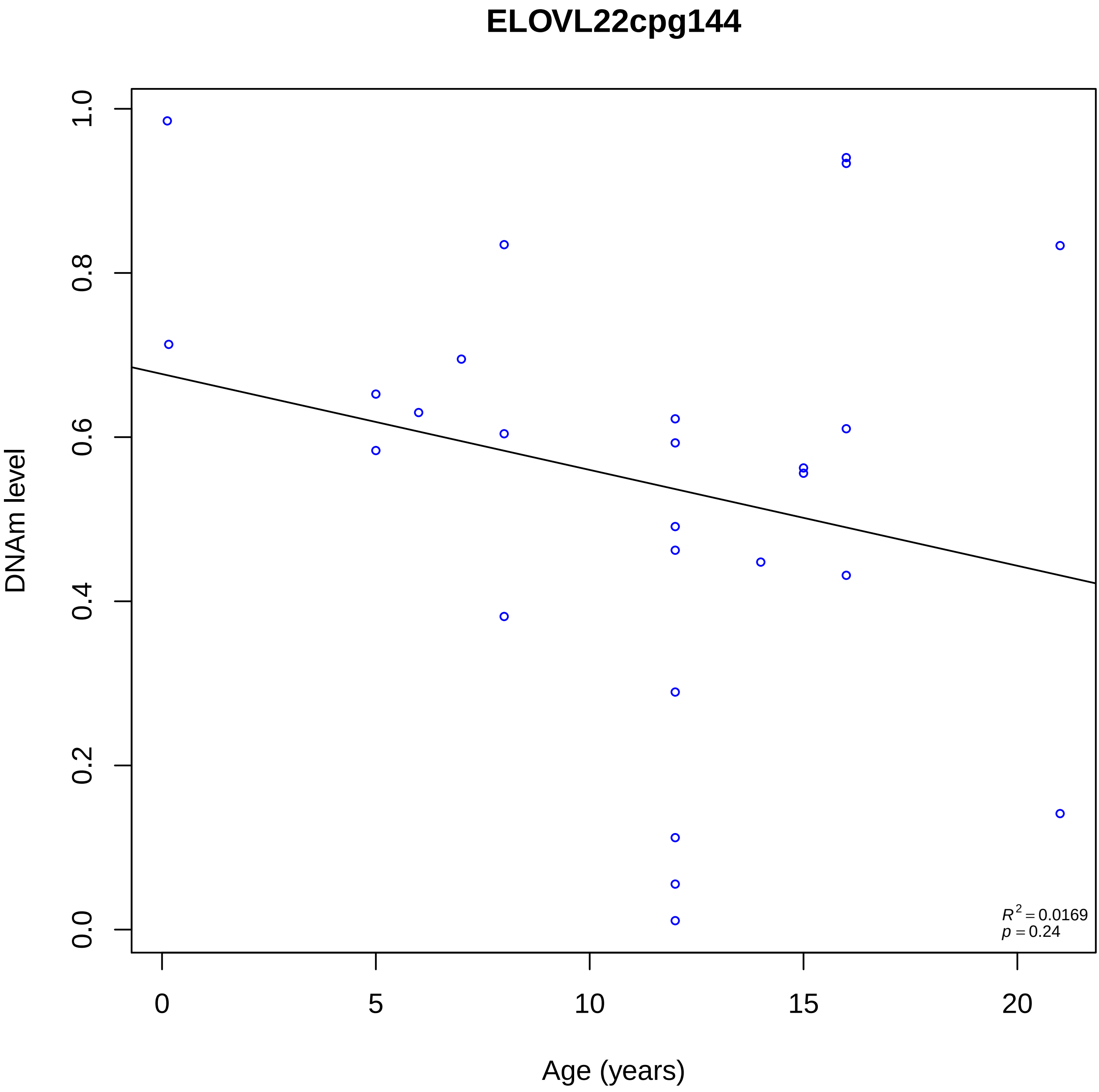
ELOVL22cpg144
DNAm level
Age (years)
0.0
0.2
0.4
0.6
0.8
1.0
0
5
10
15
20
$R^2 = 0.0169$
$p = 0.24$

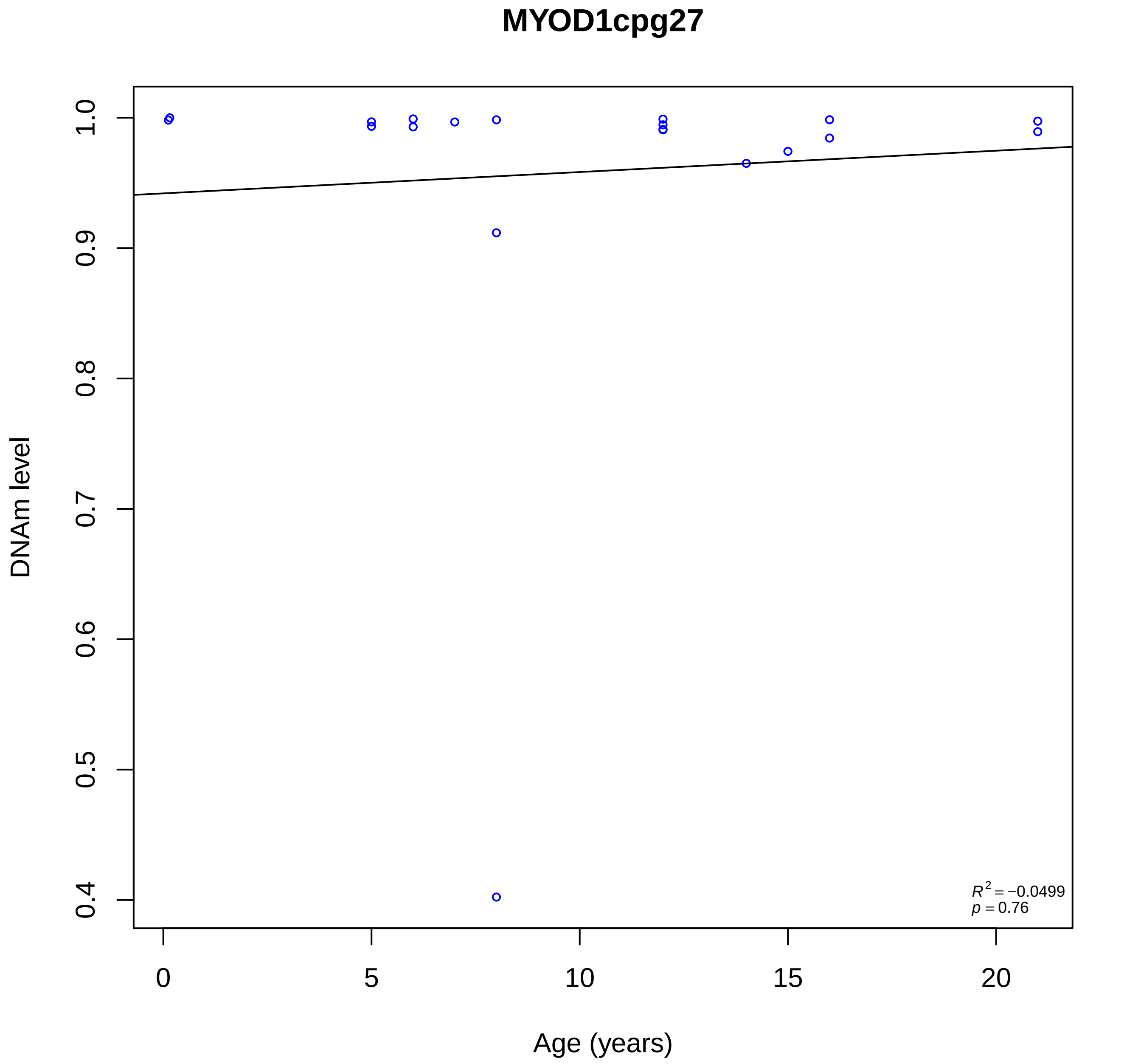
MYOD1cpg27
DNAm level
1.0
0.9
0.8
0.7
0.6
0.5
0.4
0
5
10
15
20
Age (years)
$R^2 = -0.0499$
$p = 0.76$

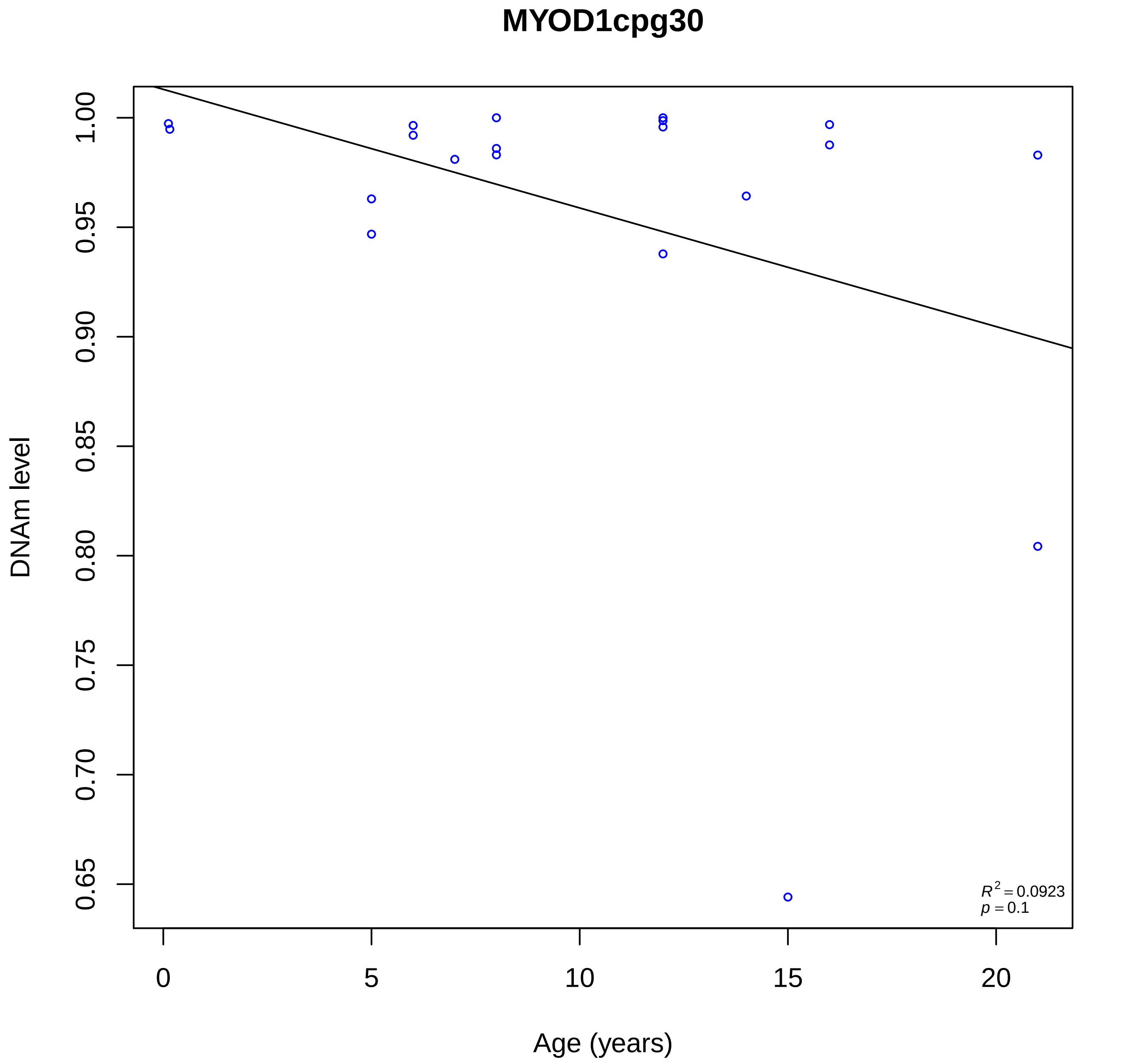

MYOD1cpg30
DNAm level
Age (years)
1.00
0.95
0.90
0.85
0.80
0.75
0.70
0.65
0
5
10
15
20
$R^2 = 0.0923$
$p = 0.1$

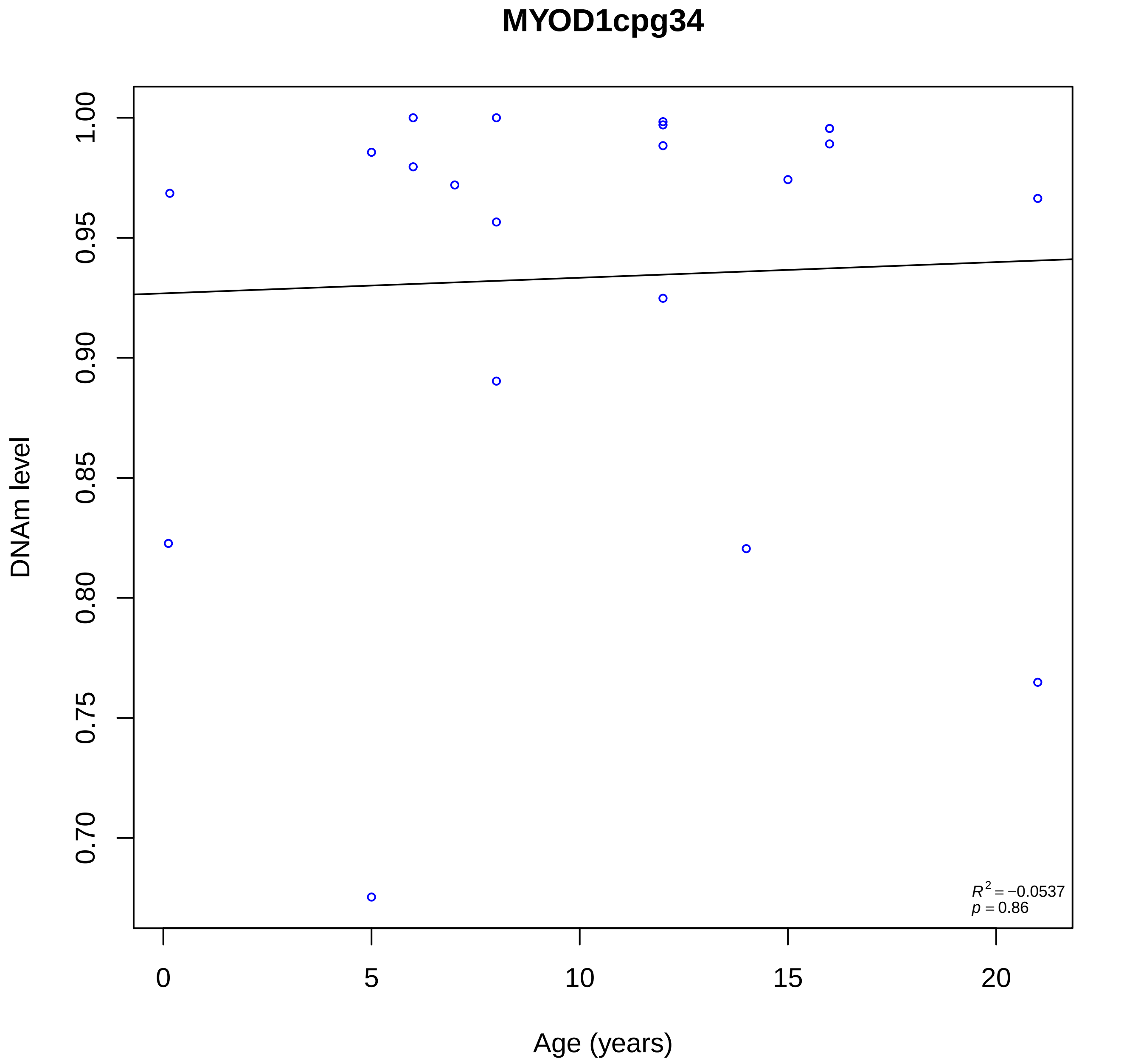

MYOD1cpg34
DNAm level
Age (years)
1.00
0.95
0.90
0.85
0.80
0.75
0.70
0
5
10
15
20
$R^2 = -0.0537$
$p = 0.86$

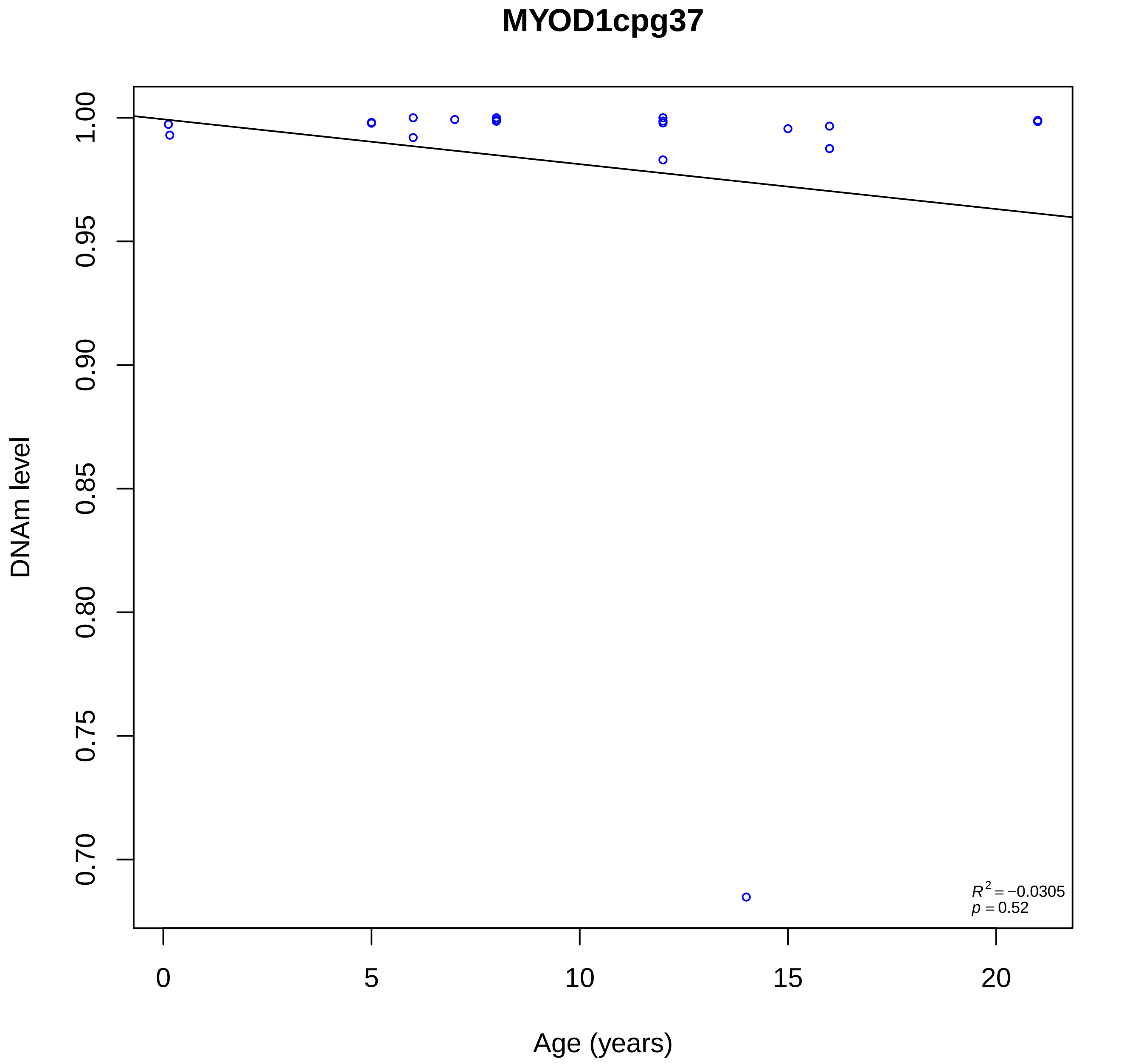
MYOD1cpg37
DNAm level
1.00
0.95
0.90
0.85
0.80
0.75
0.70
0
5
10
15
20
Age (years)
$R^2 = -0.0305$
$p = 0.52$

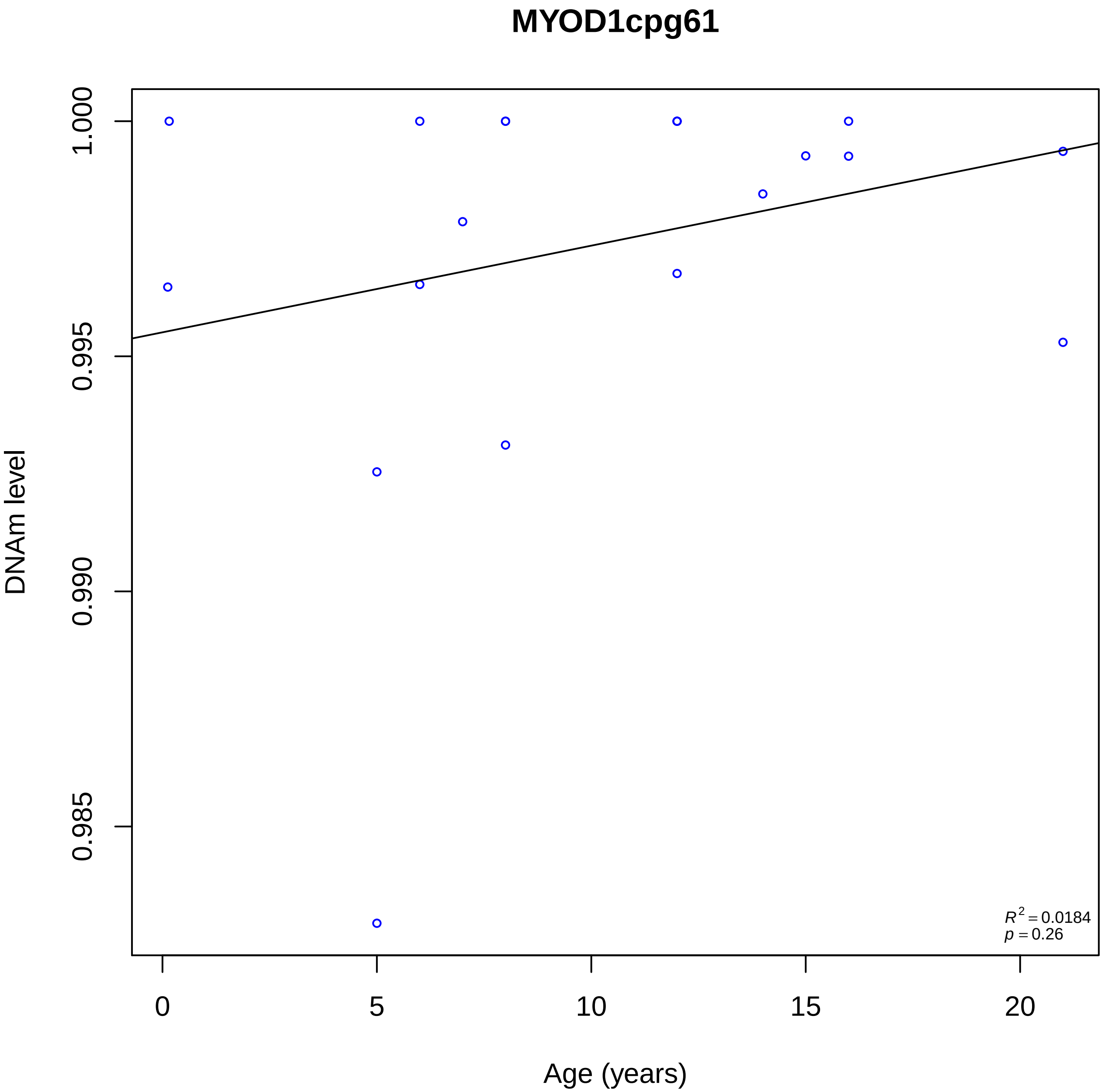
MYOD1cpg61
DNAm level
1.000
0.995
0.990
0.985
0
5
10
15
20
Age (years)
$R^2 = 0.0184$
$p = 0.26$

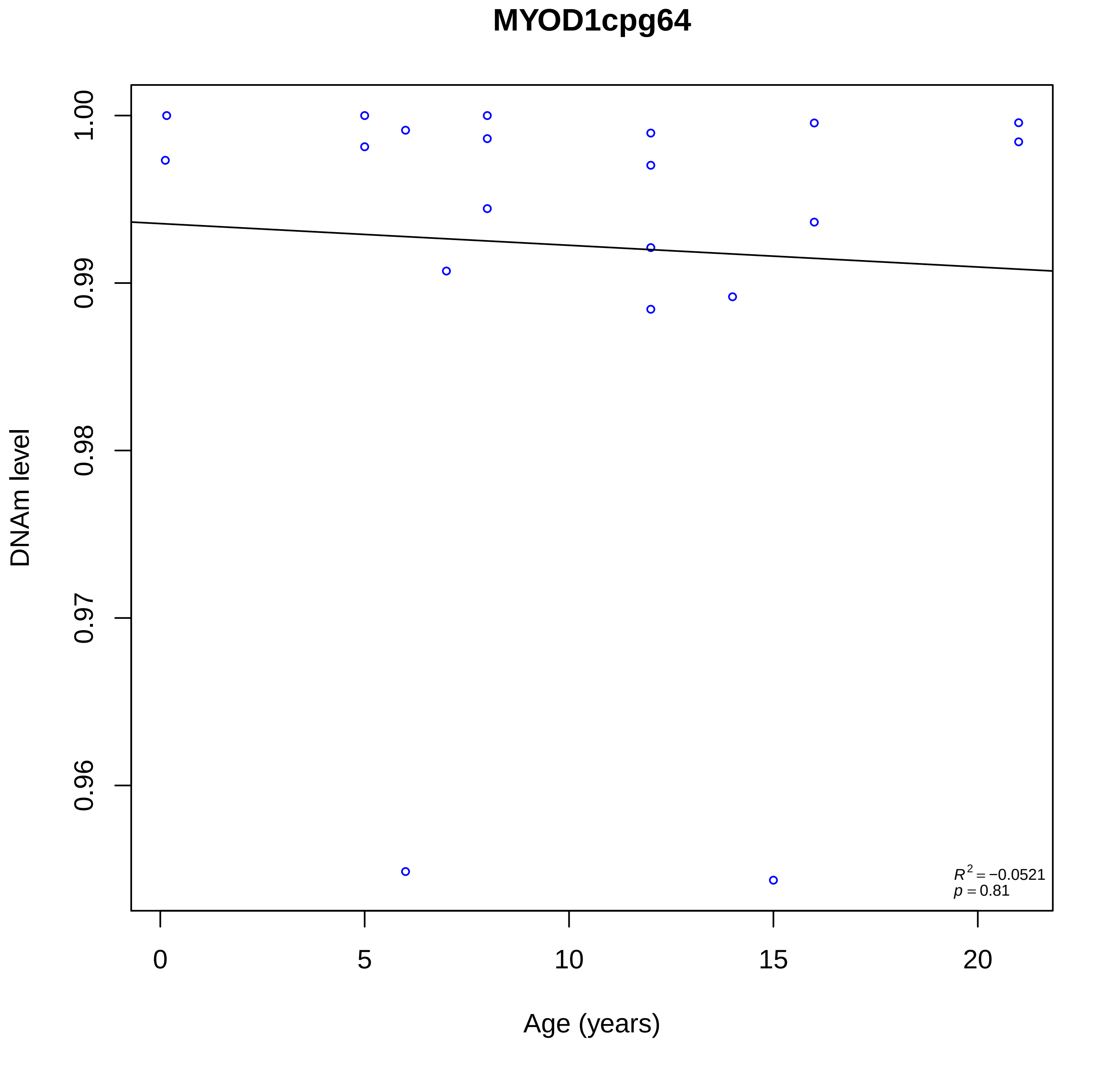
MYOD1cpg64
DNAm level
1.00
0.99
0.98
0.97
0.96
Age (years)
0
5
10
15
20
$R^2 = -0.0521$
$p = 0.81$

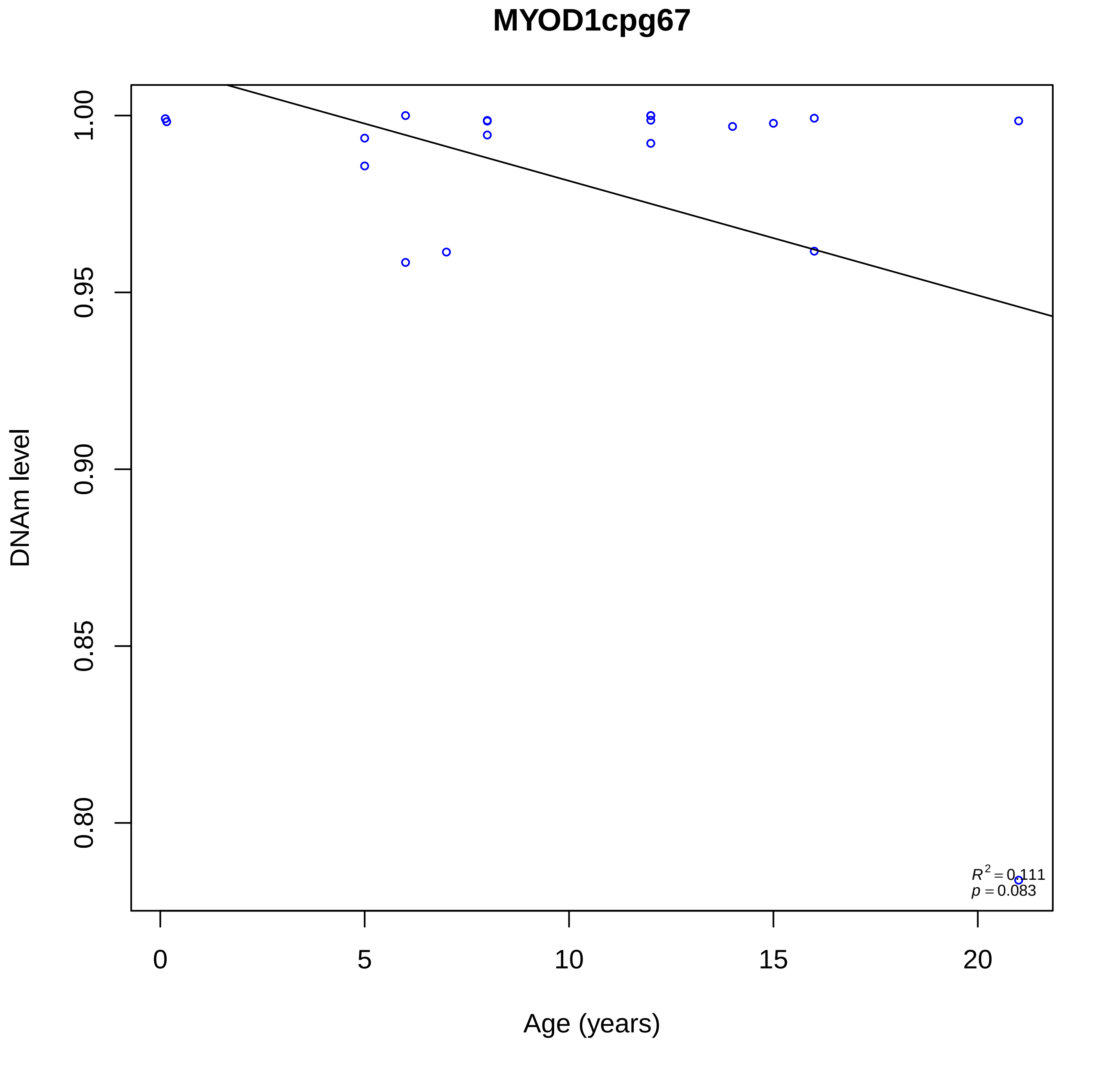

MYOD1cpg67
DNAm level
1.00
0.95
0.90
0.85
0.80
0
5
10
15
20
Age (years)
$R^2 = 0.111$
$p = 0.083$

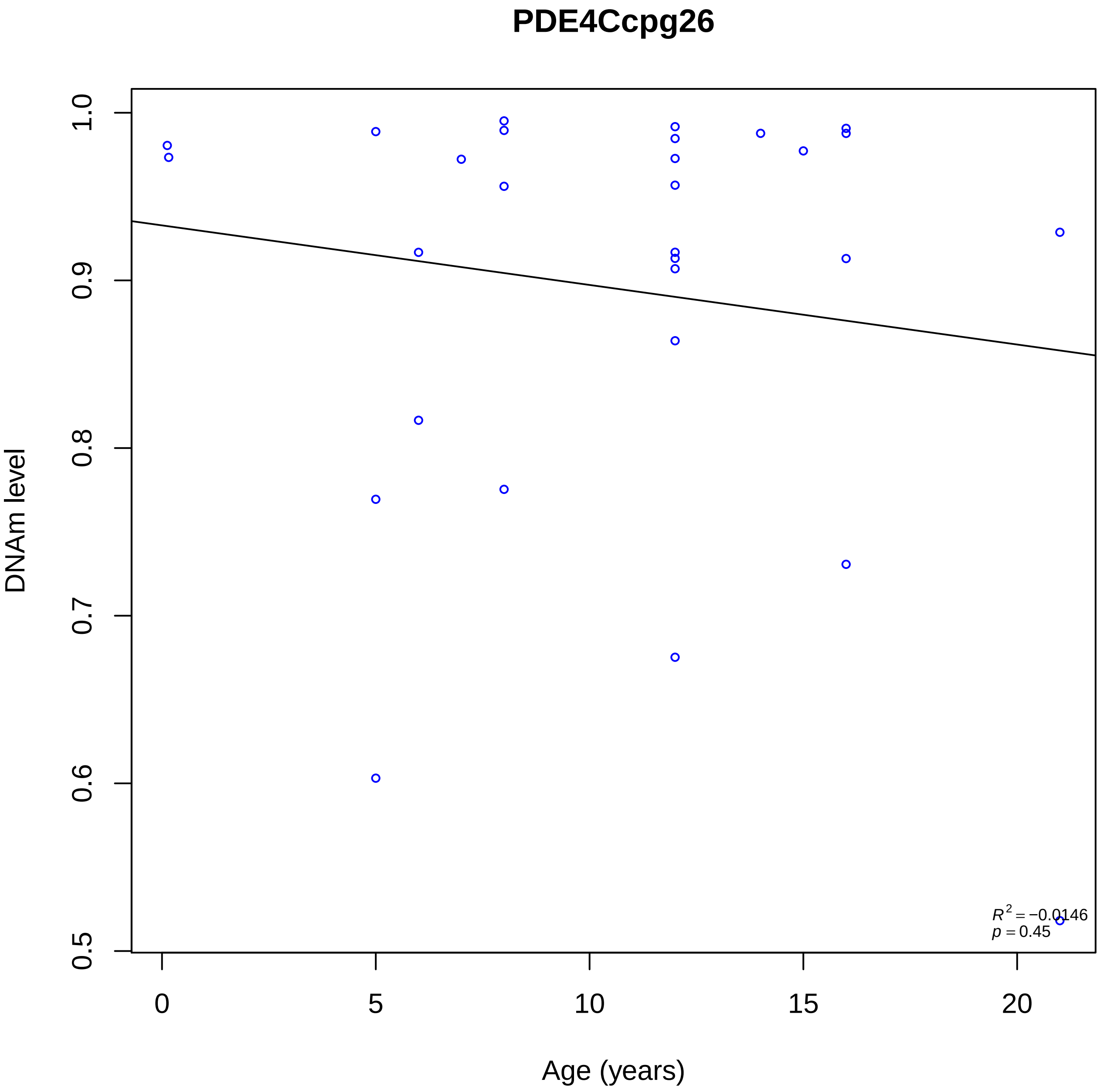

PDE4Ccpg26
DNAm level
Age (years)
0.5
0.6
0.7
0.8
0.9
1.0
0
5
10
15
20
$R^2 = -0.0146$
$p = 0.45$

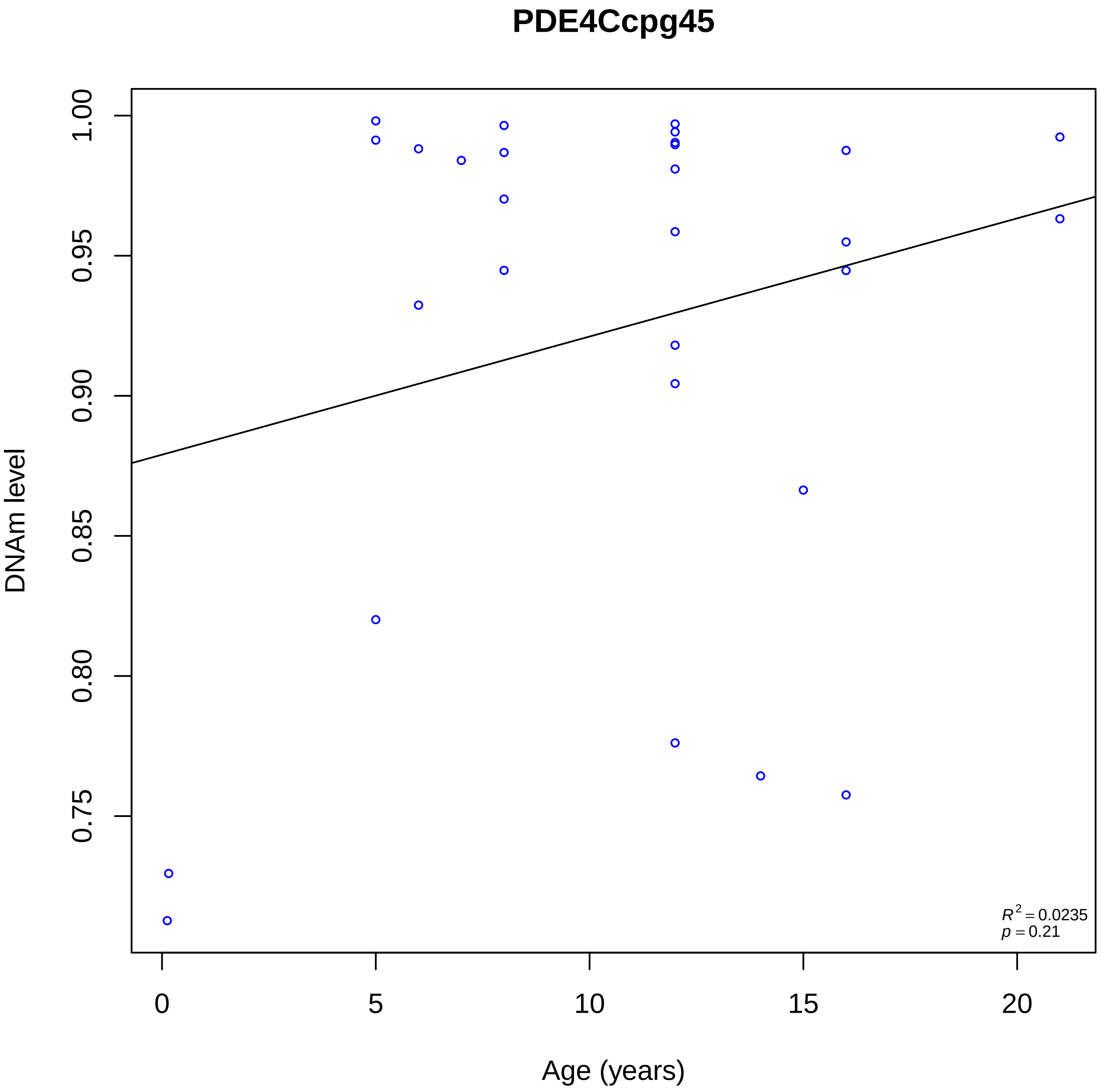

PDE4Ccpg45
DNAm level
Age (years)
1.00
0.95
0.90
0.85
0.80
0.75
0
5
10
15
20
$R^2 = 0.0235$
$p = 0.21$

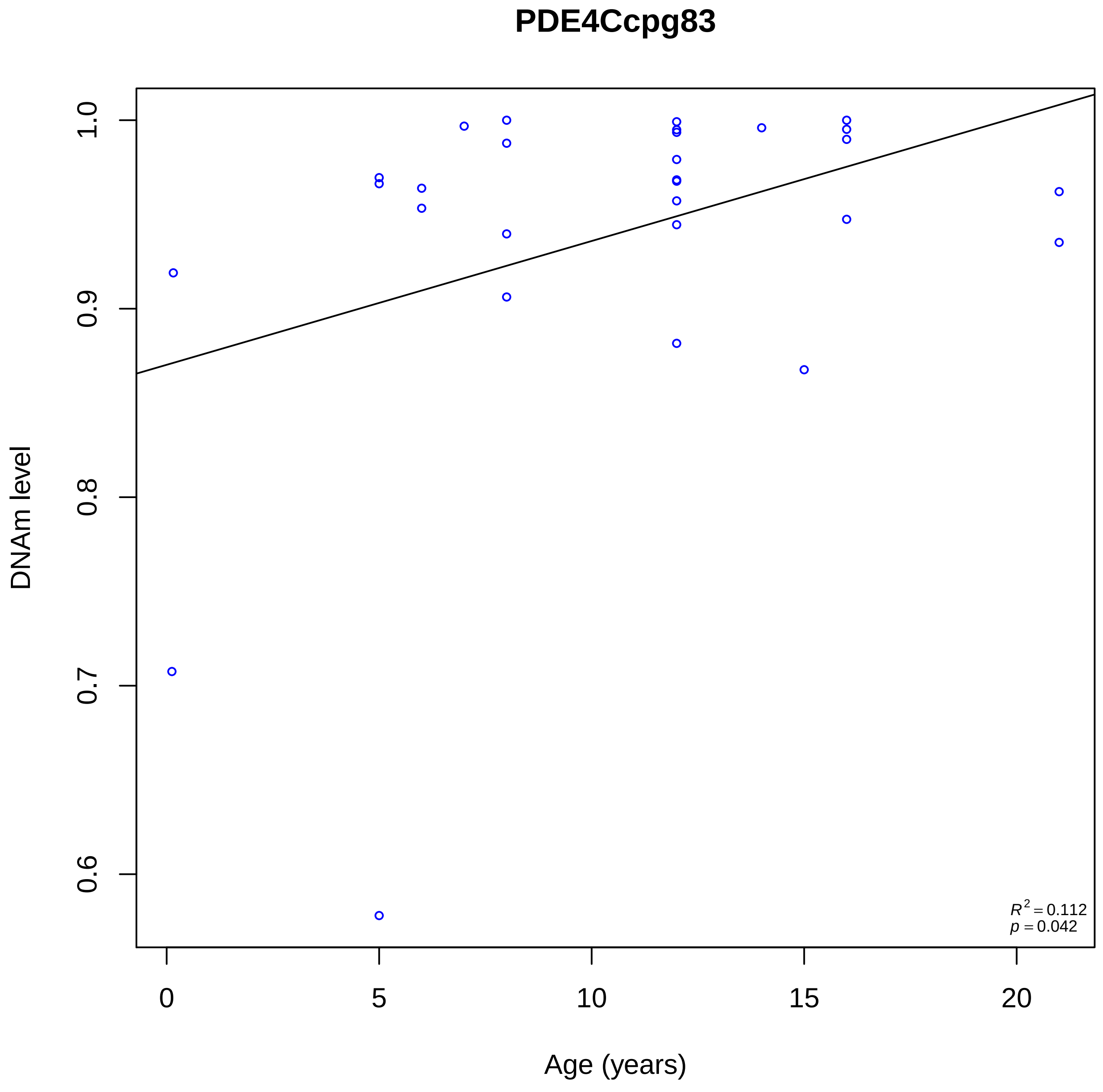
PDE4Ccpg83
DNAm level
Age (years)
0.6
0.7
0.8
0.9
1.0
0
5
10
15
20
$R^2 = 0.112$
$p = 0.042$

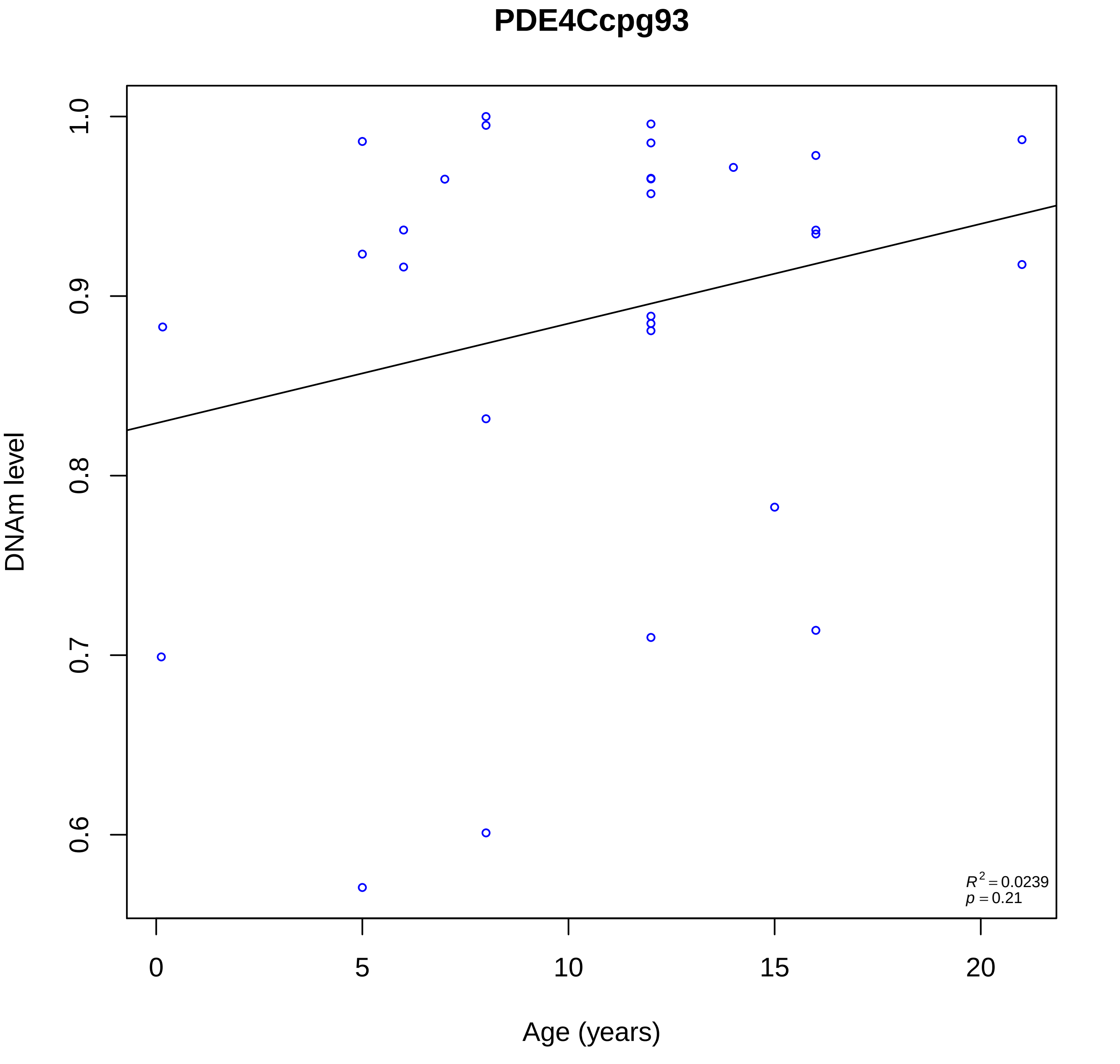
PDE4Ccpg93
DNAm level
1.0
0.9
0.8
0.7
0.6
0
5
10
15
20
Age (years)
R^2 = 0.0239
p = 0.21

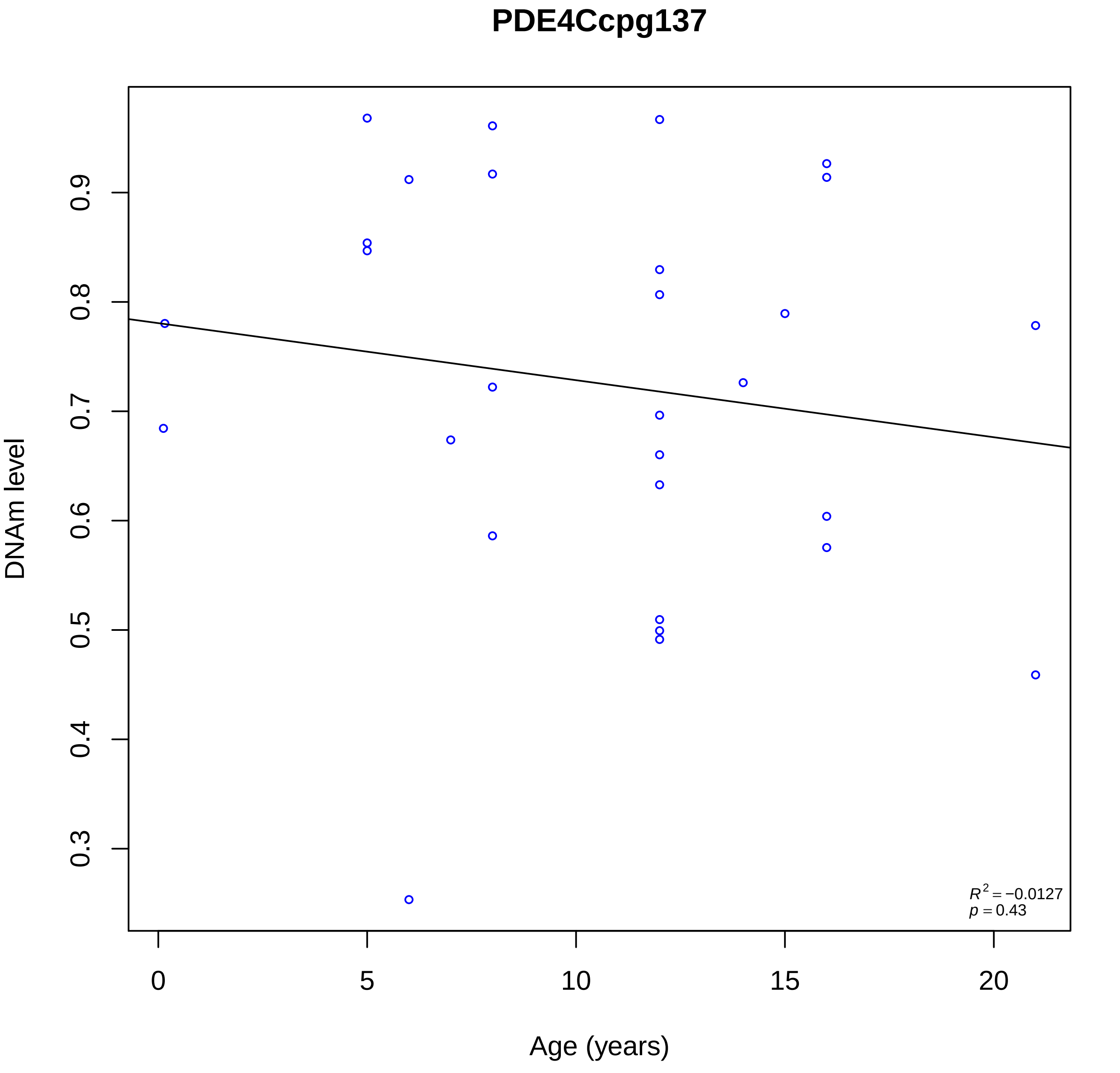

PDE4Ccpg137
DNAm level
0.3
0.4
0.5
0.6
0.7
0.8
0.9
Age (years)
0
5
10
15
20
$R^2 = -0.0127$
$p = 0.43$

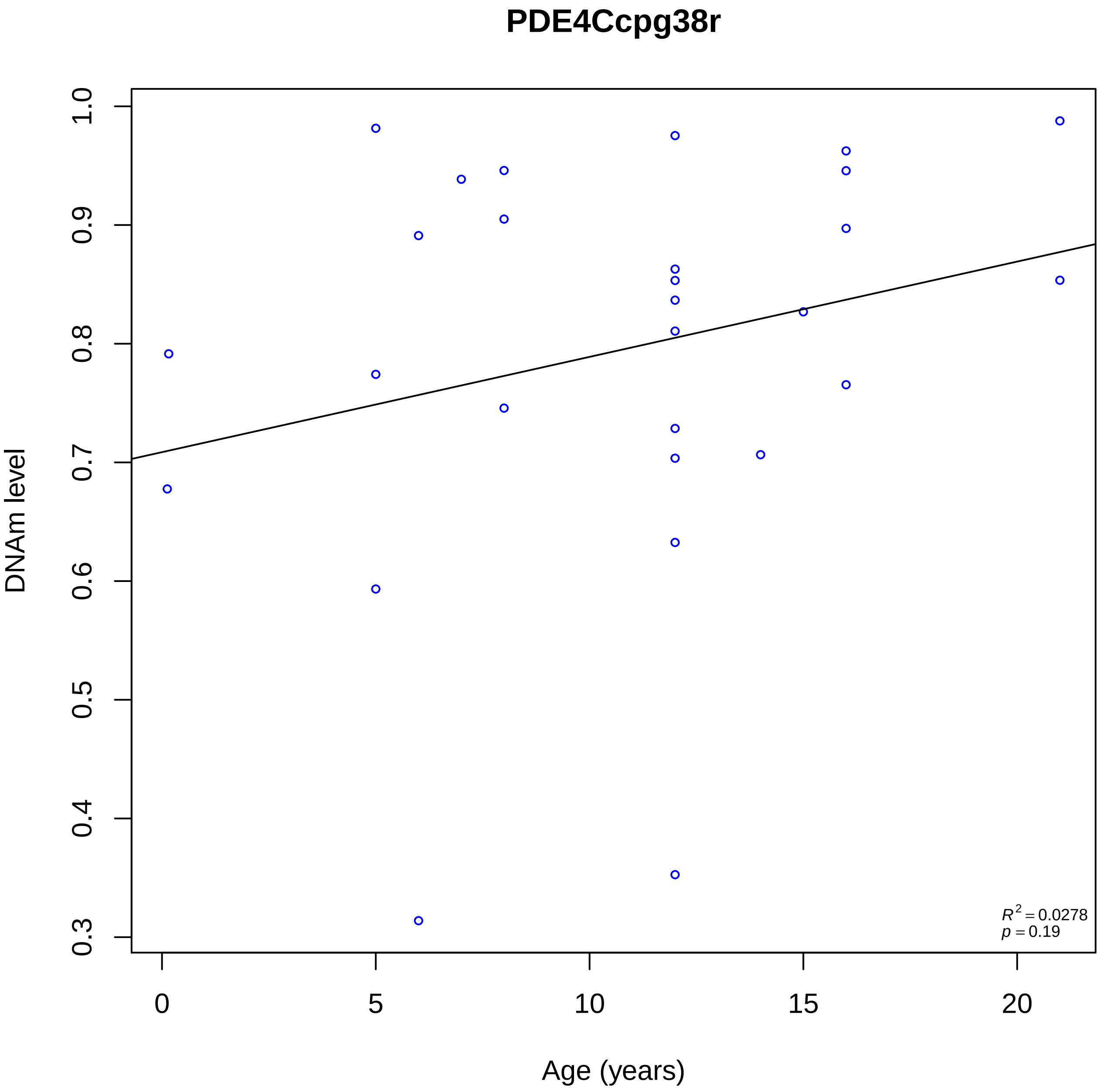
PDE4Ccpg38r
DNAm level
Age (years)
0.3
0.4
0.5
0.6
0.7
0.8
0.9
1.0
0
5
10
15
20
$R^2 = 0.0278$
$p = 0.19$

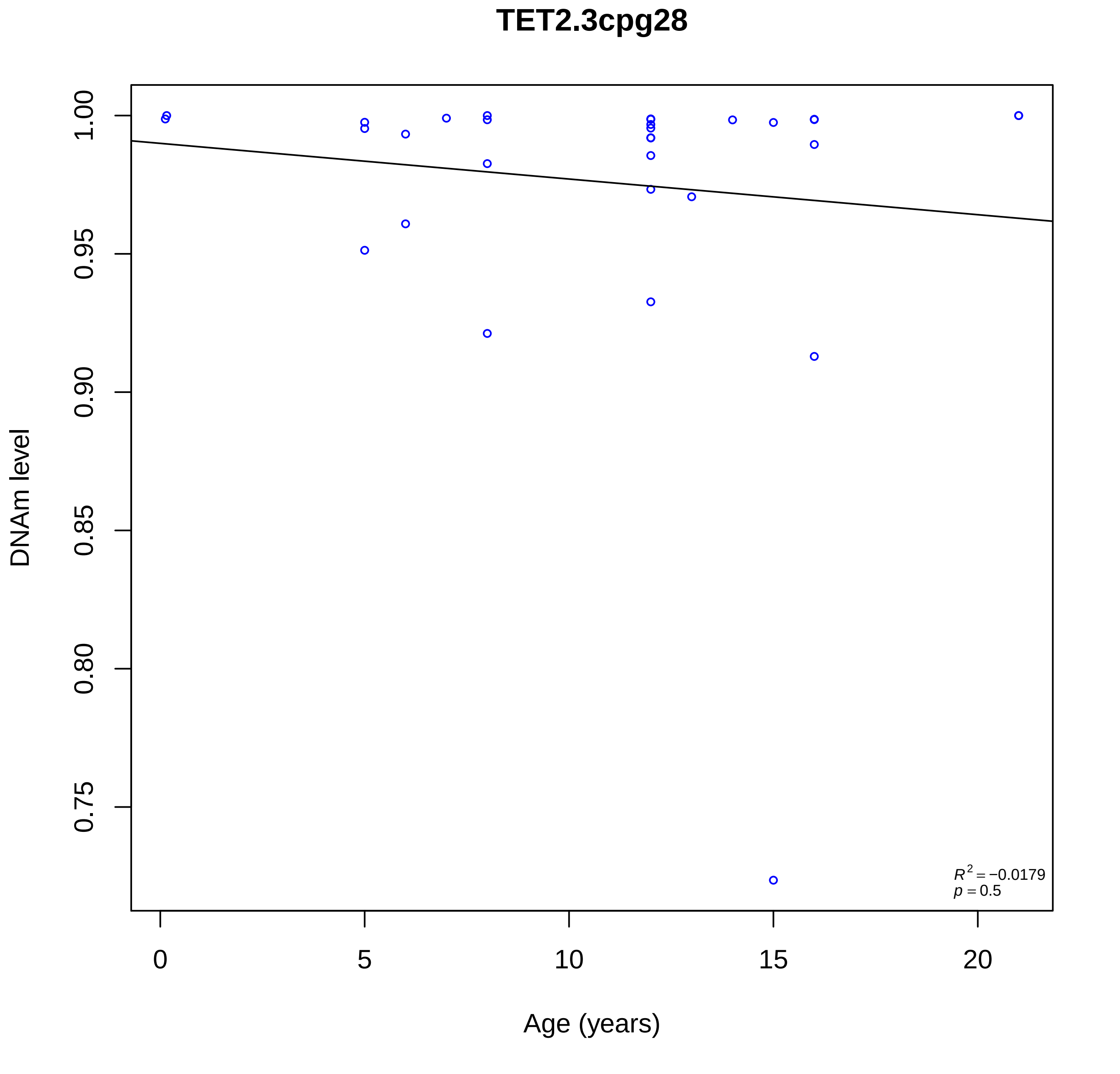
TET2.3cpg28
DNAm level
1.00
0.95
0.90
0.85
0.80
0.75
0
5
10
15
20
Age (years)
$R^2 = -0.0179$
$p = 0.5$

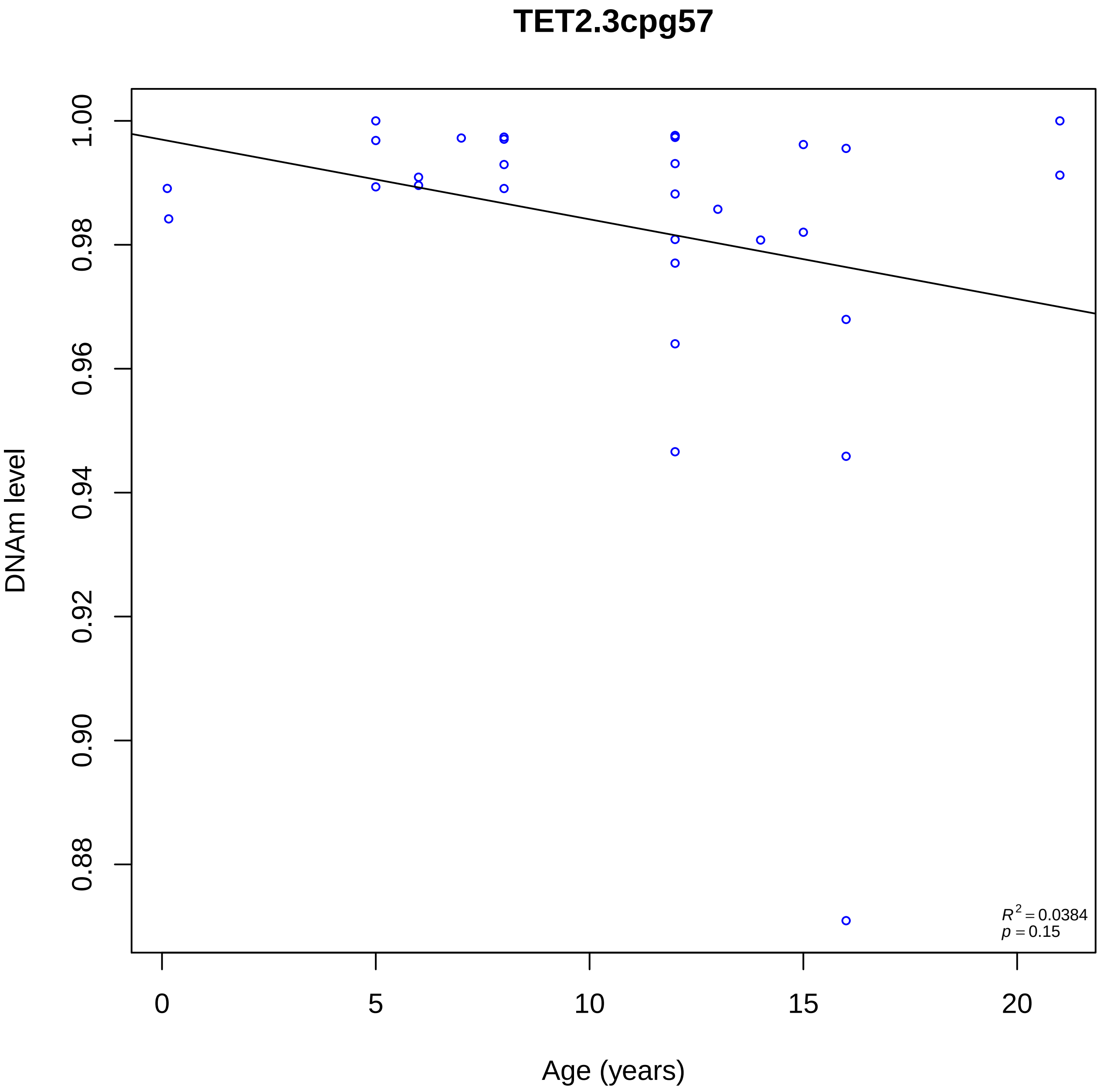

TET2.3cpg57
DNAm level
1.00
0.98
0.96
0.94
0.92
0.90
0.88
0
5
10
15
20
Age (years)
$R^2 = 0.0384$
$p = 0.15$

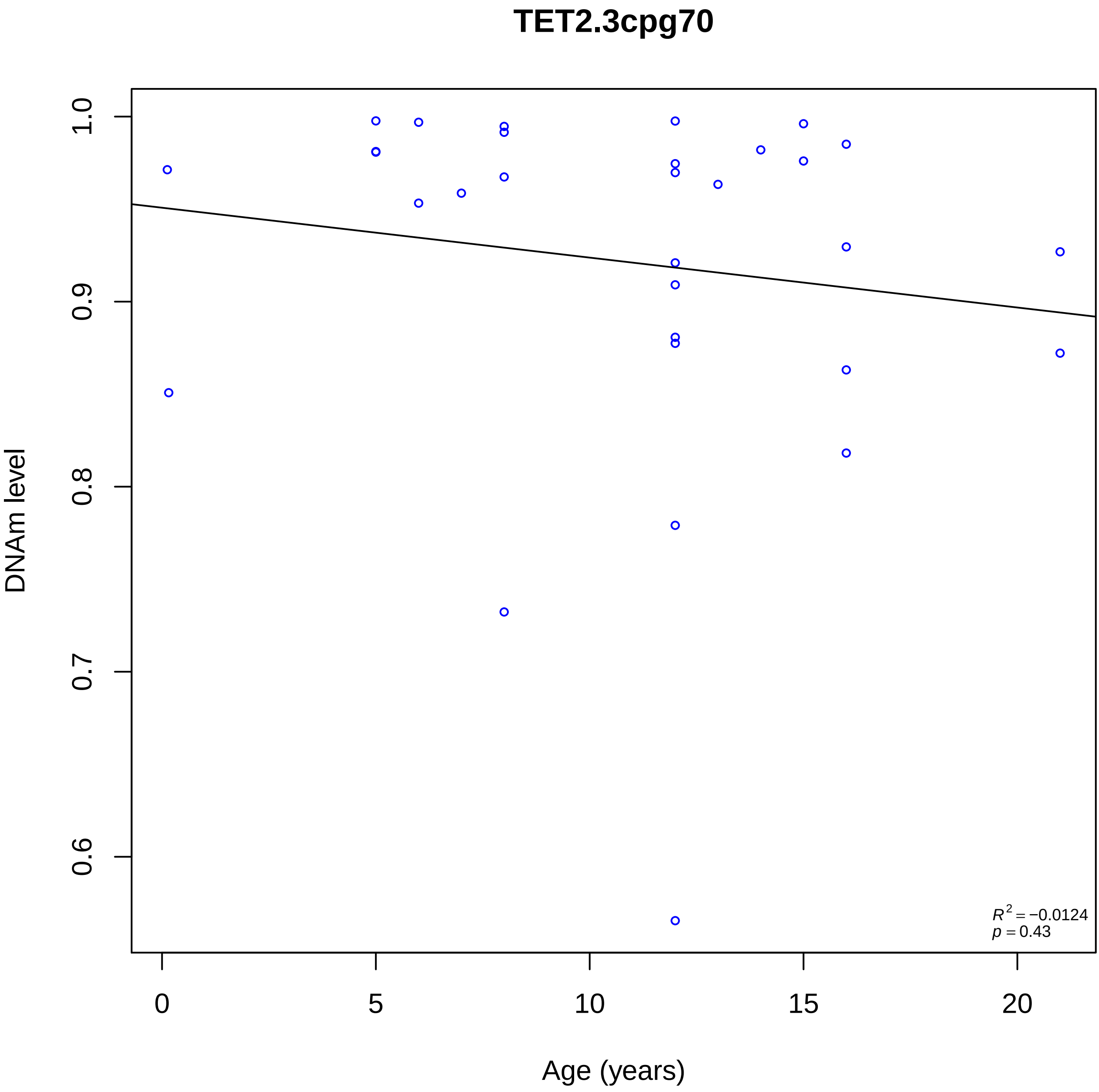
TET2.3cpg70
DNAm level
Age (years)
0
5
10
15
20
0.6
0.7
0.8
0.9
1.0
$R^2 = -0.0124$
$p = 0.43$

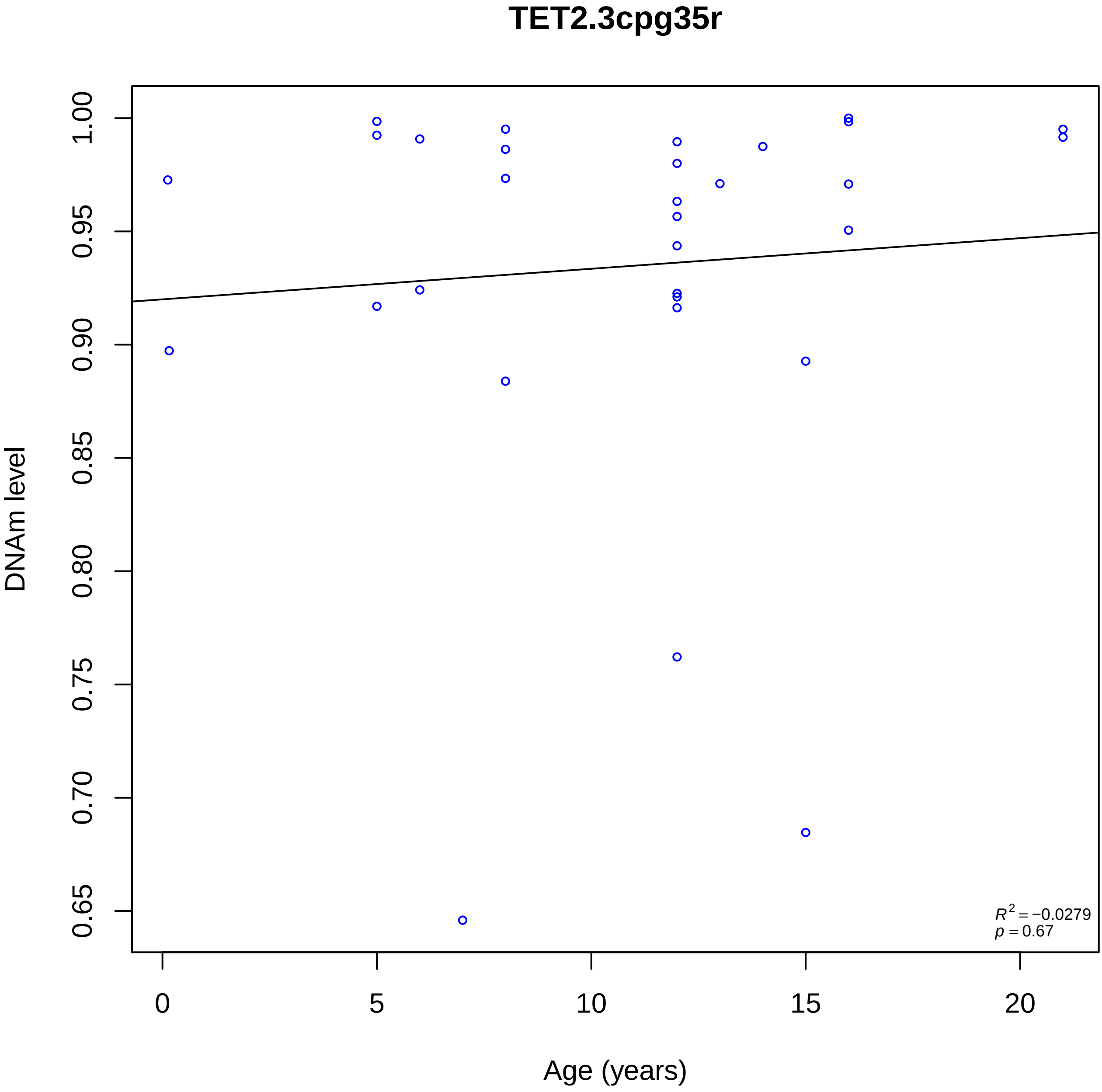

TET2.3cpg35r
DNAm level
1.00
0.95
0.90
0.85
0.80
0.75
0.70
0.65
0
5
10
15
20
Age (years)
$R^2 = -0.0279$
$p = 0.67$

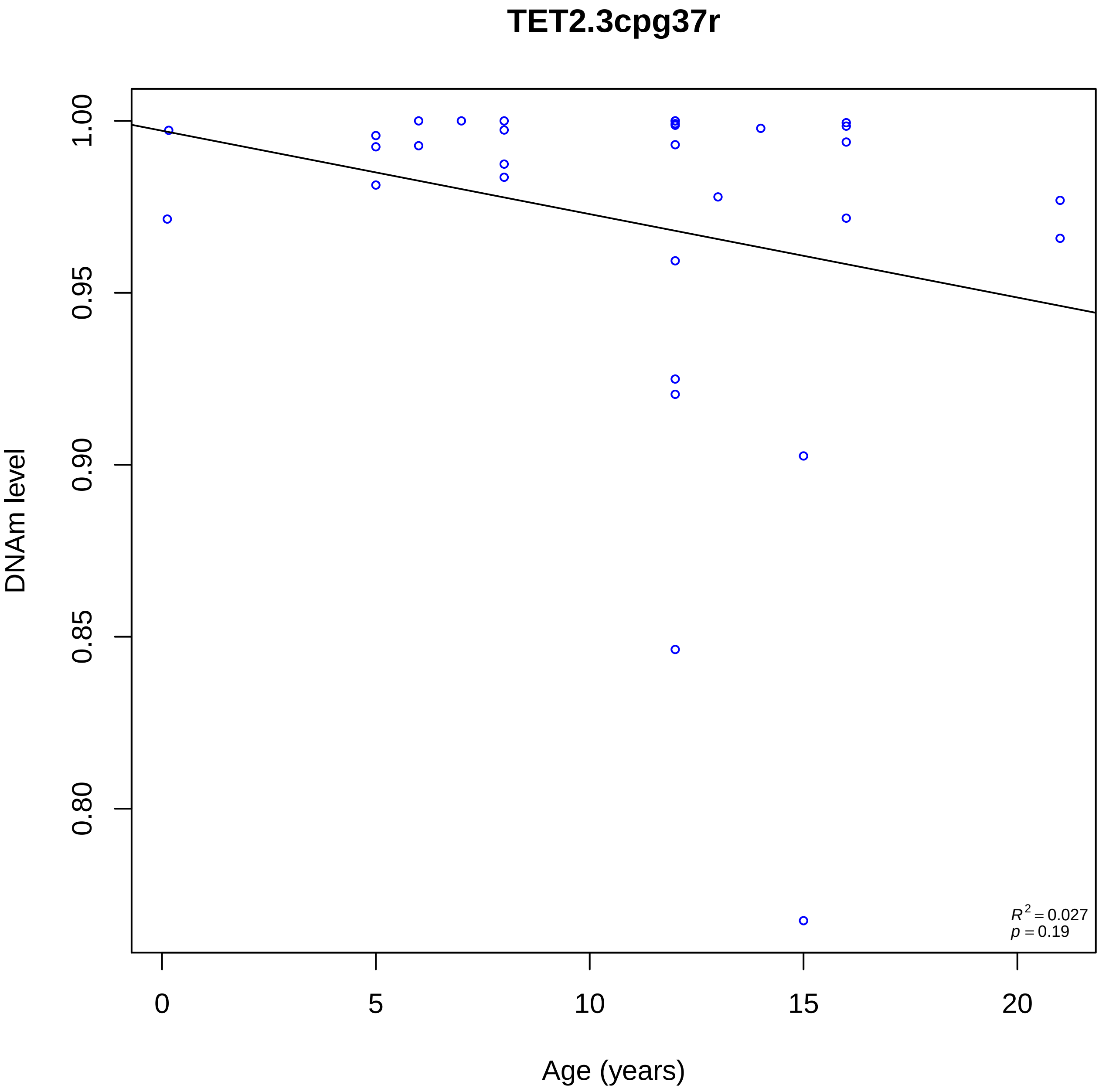

TET2.3cpg37r
DNAm level
1.00
0.95
0.90
0.85
0.80
0
5
10
15
20
Age (years)
$R^2 = 0.027$
$p = 0.19$

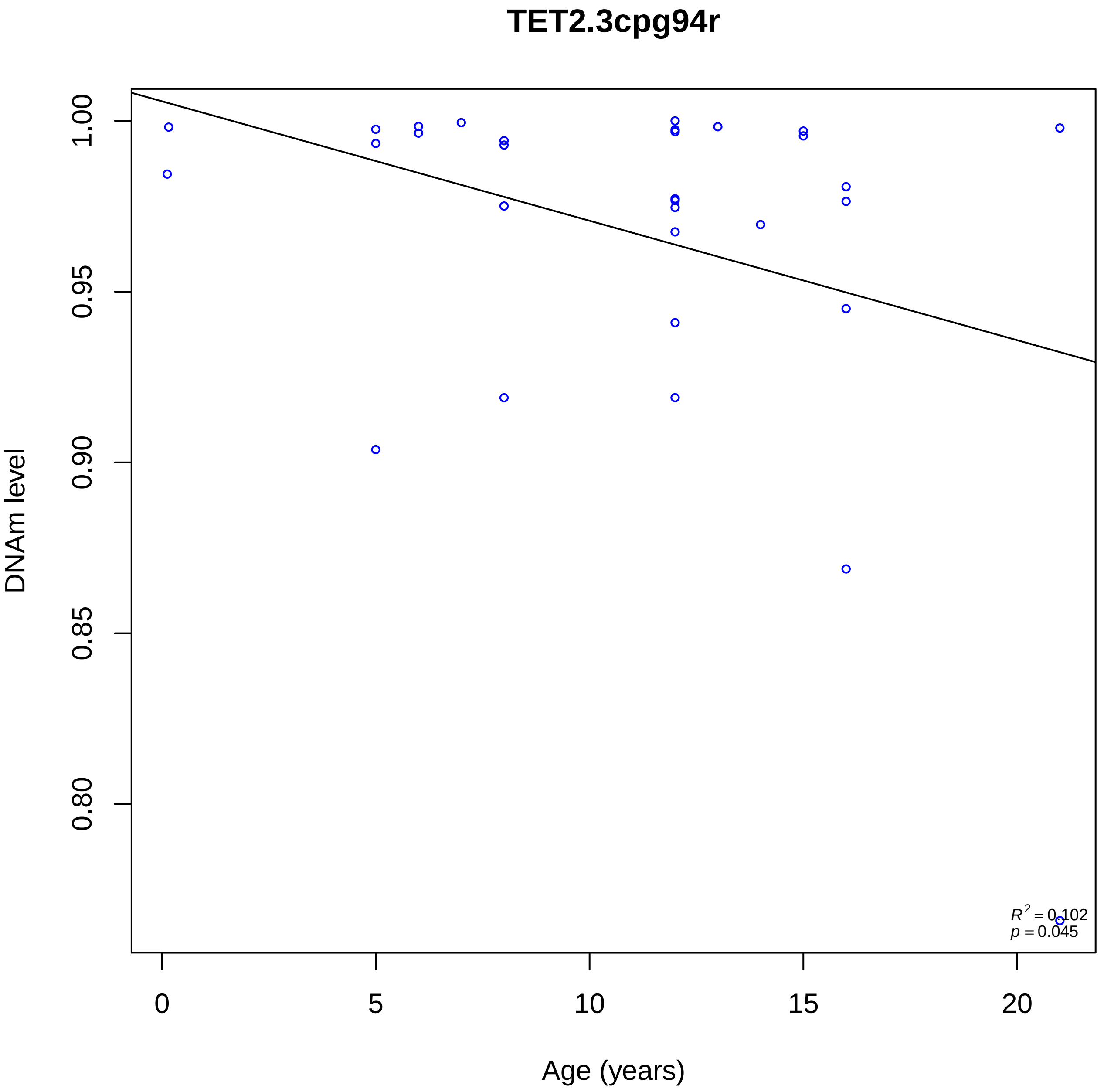
TET2.3cpg94r
DNAm level
1.00
0.95
0.90
0.85
0.80
0
5
10
15
20
Age (years)
$R^2 = 0.102$
$p = 0.045$
