# Supplementary material for: DNA methylation levels in candidate genes associated with chronological age in mammals are not conserved in a long-lived seabird
Source: PLoS One. 2017 Dec 7;12(12):e0189181. doi: 10.1371/journal.pone.0189181 (PMC5720723; doi:10.1371/journal.pone.0189181)
Supplement: S5 File — (PDF) [file pone.0189181.s010.pdf]

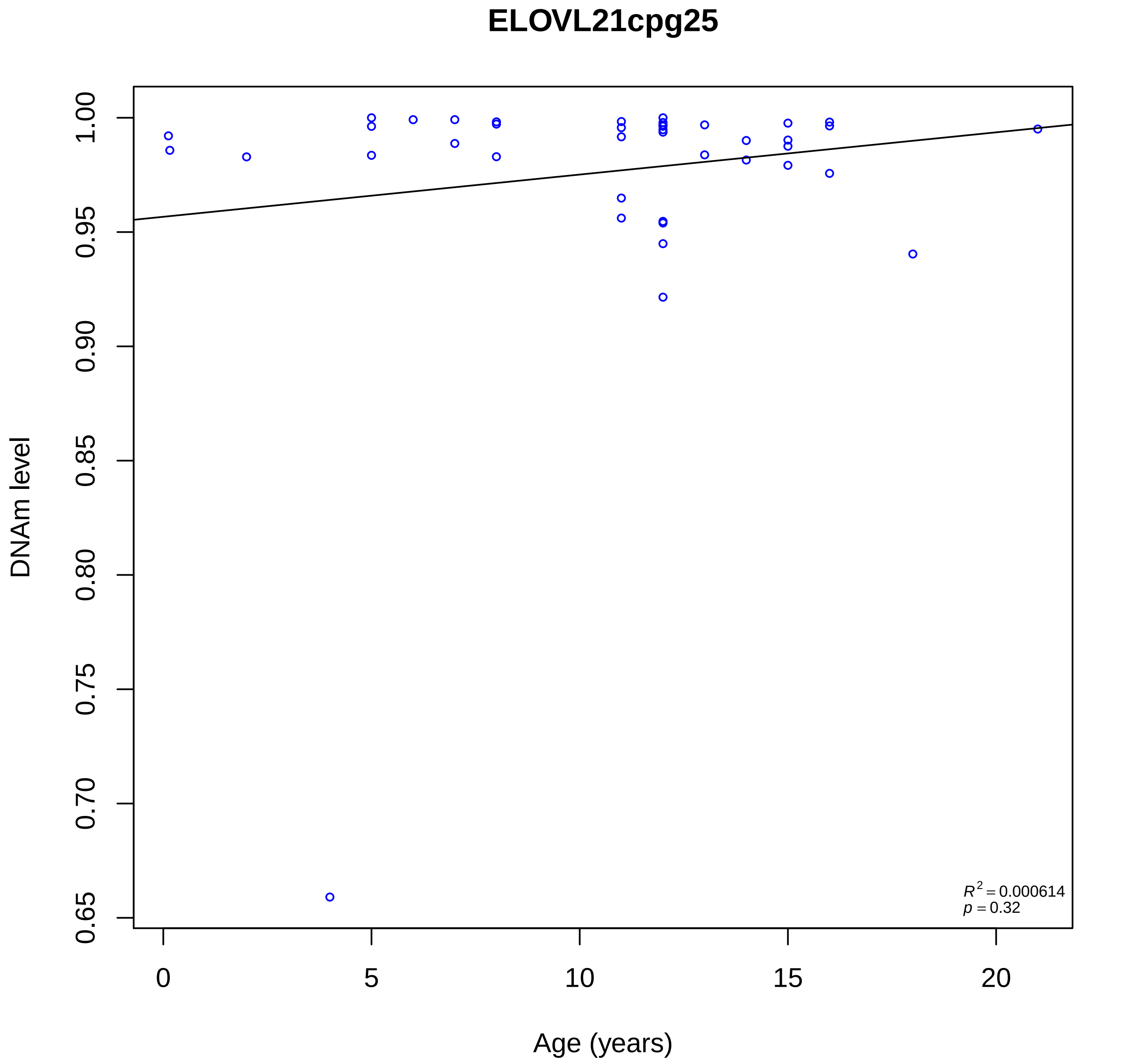
ELOVL21cpg25
DNAm level
Age (years)
0.65
0.70
0.75
0.80
0.85
0.90
0.95
1.00
0
5
10
15
20
$R^2 = 0.000614$
$p = 0.32$

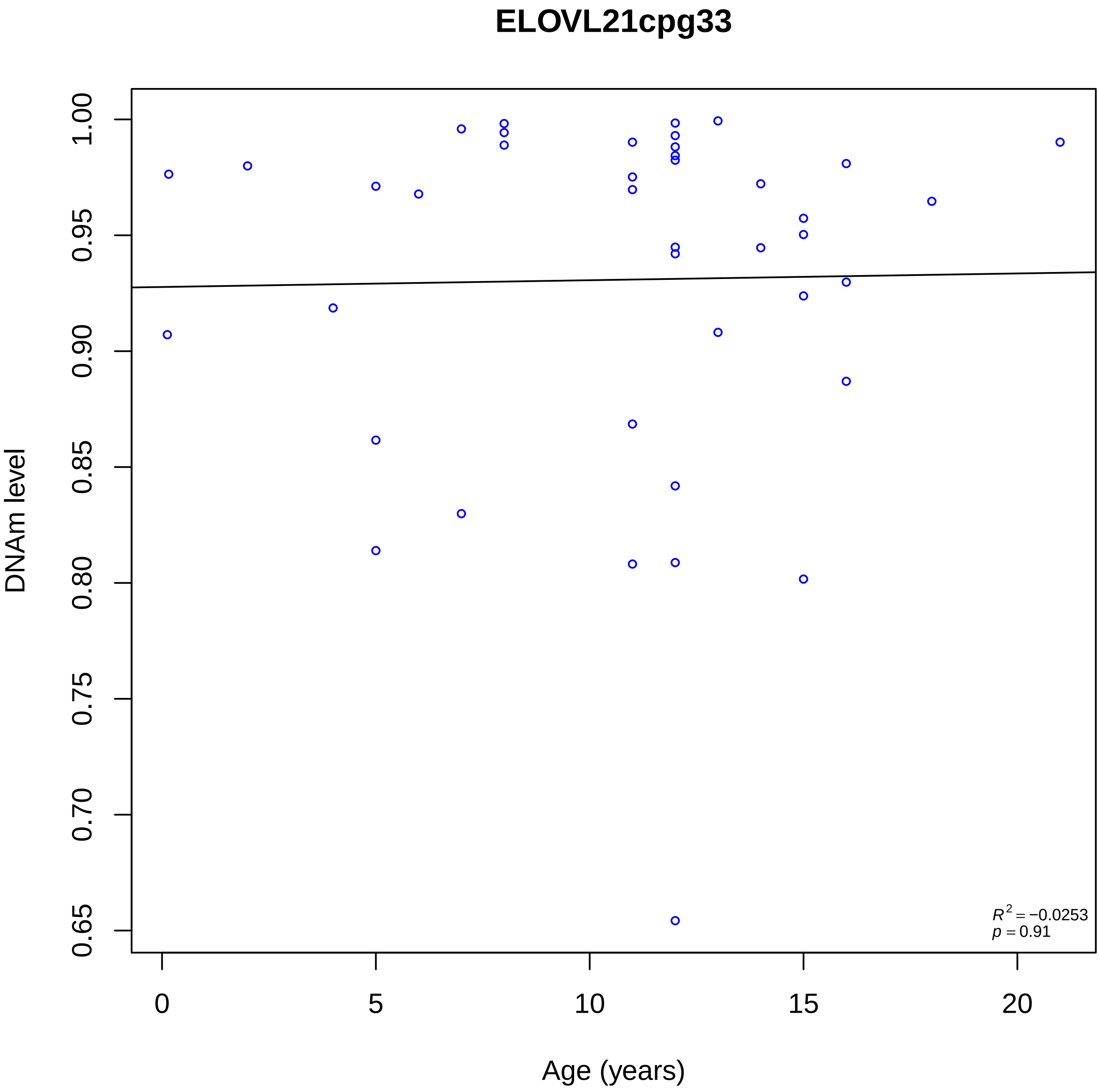
ELOVL21cpg33
DNAm level
Age (years)
0.65
0.70
0.75
0.80
0.85
0.90
0.95
1.00
0
5
10
15
20
$R^2 = -0.0253$
$p = 0.91$

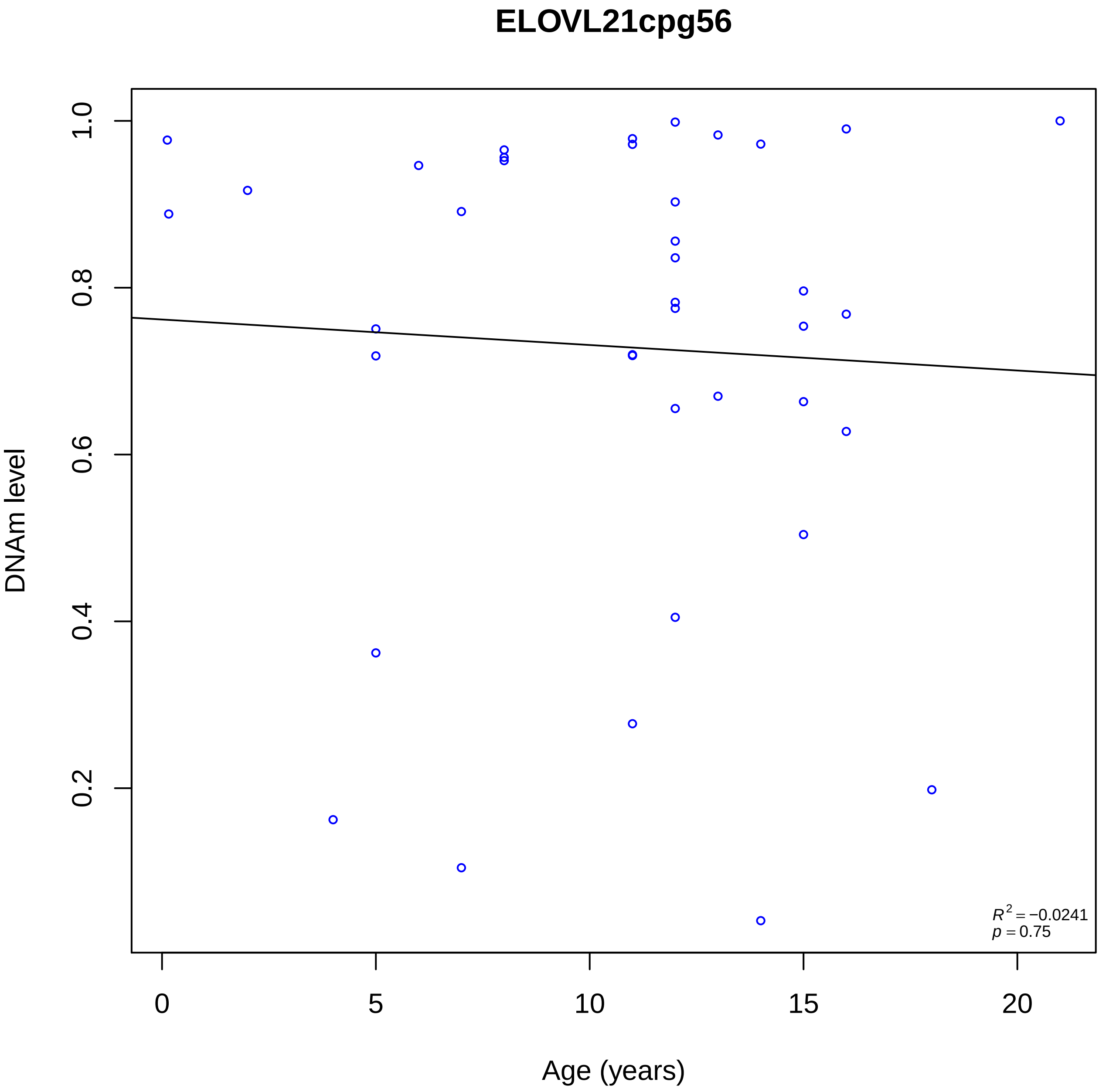

ELOVL21cpg56
1.0
0.8
0.6
0.4
0.2
DNAm level
0
5
10
15
20
Age (years)
$R^2 = -0.0241$
$p = 0.75$

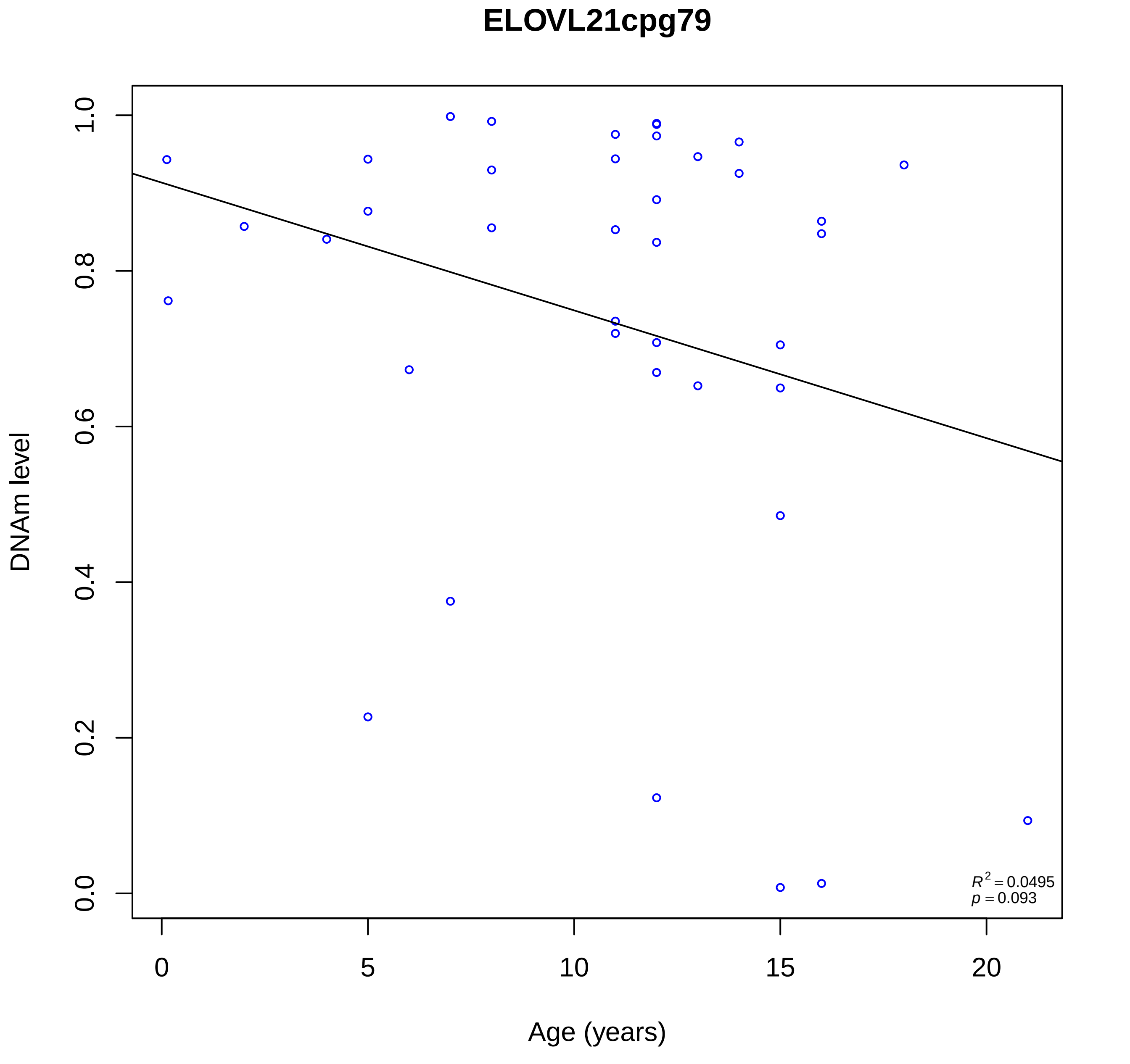
ELOVL21cpg79
DNAm level
Age (years)
0.0
0.2
0.4
0.6
0.8
1.0
0
5
10
15
20
$R^2 = 0.0495$
$p = 0.093$

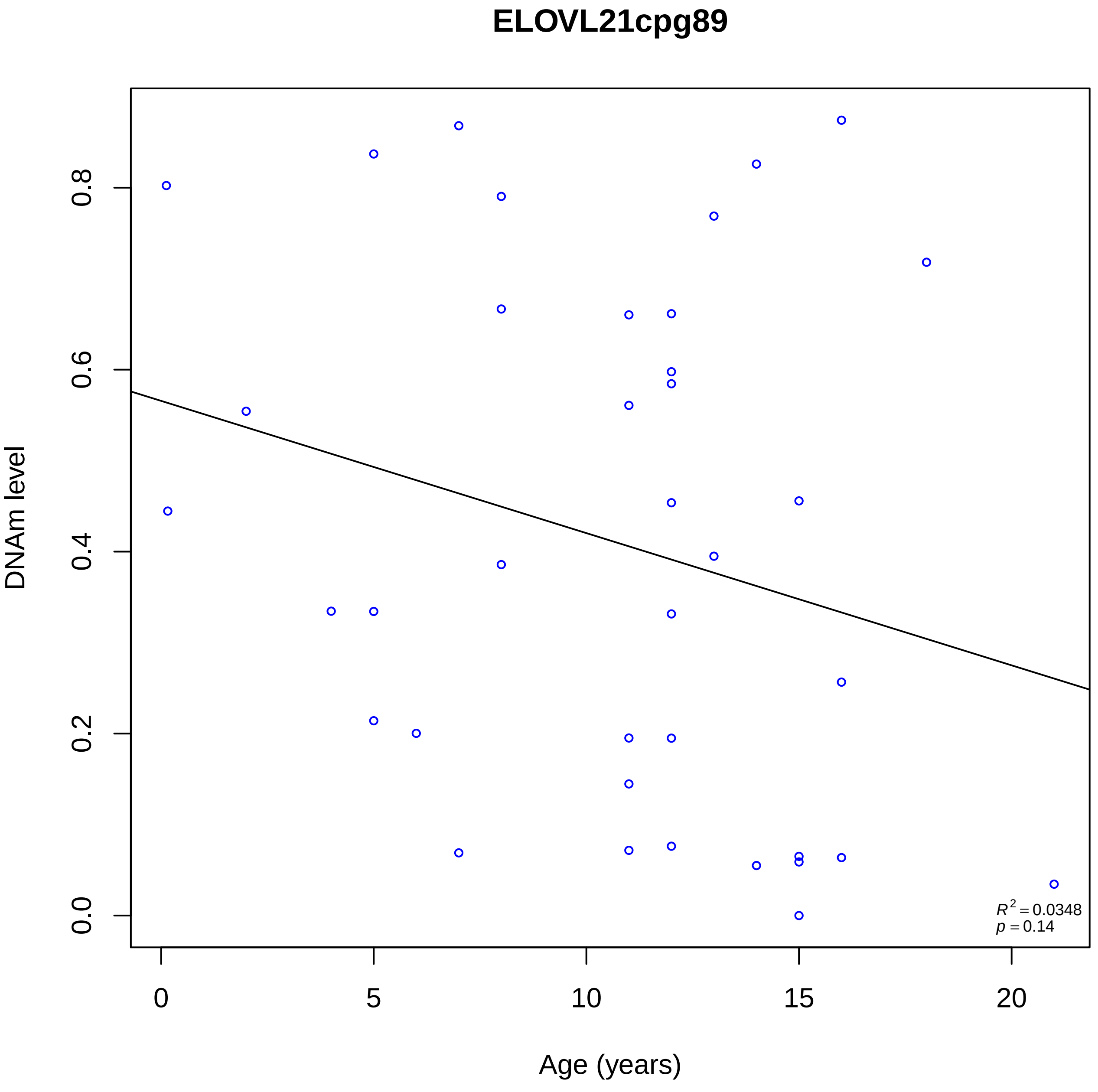
ELOVL21cpg89
DNAm level
Age (years)
0.0
0.2
0.4
0.6
0.8
0
5
10
15
20
$R^2 = 0.0348$
$p = 0.14$

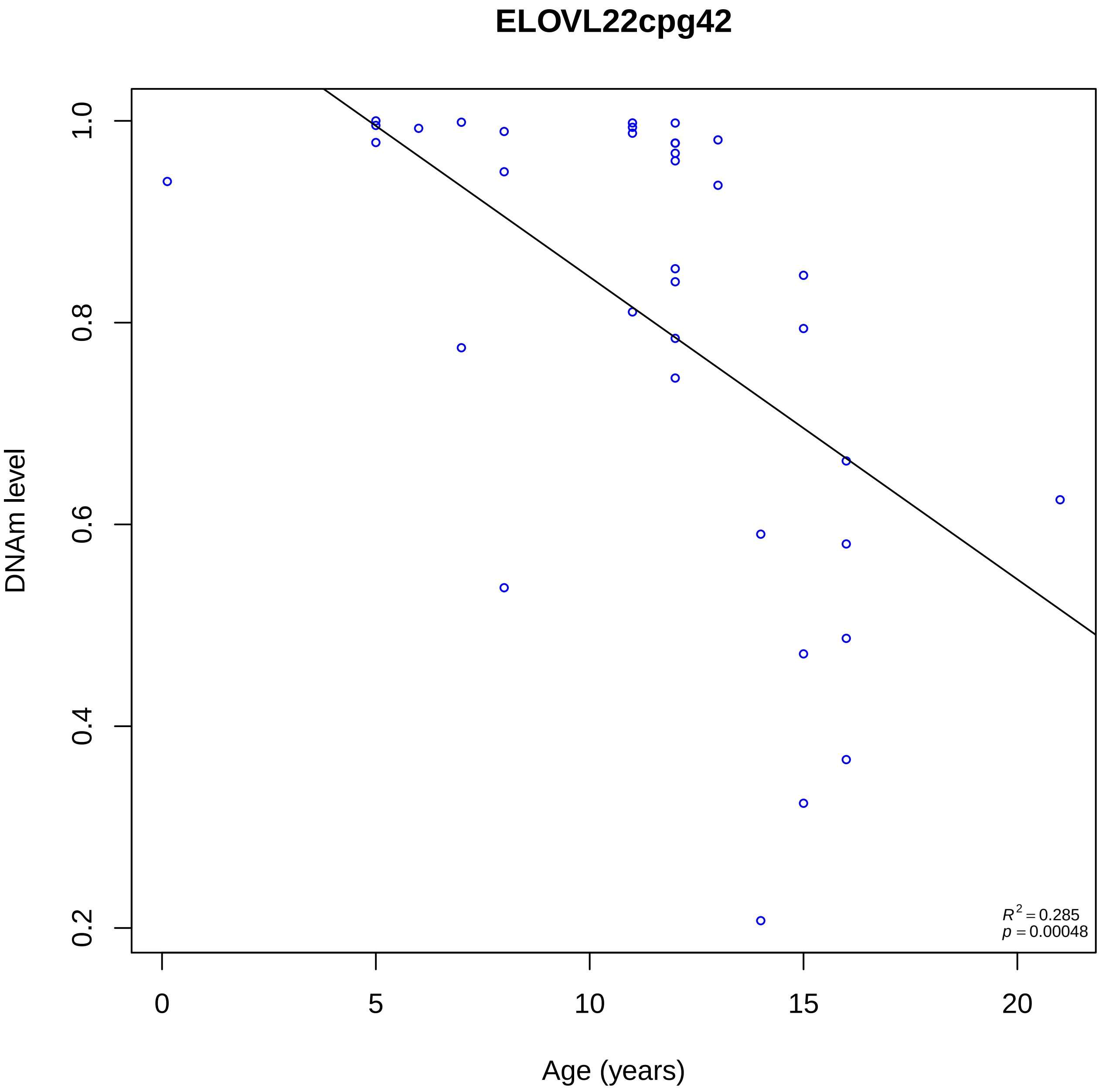
ELOVL22cpg42
DNAm level
Age (years)
0.2
0.4
0.6
0.8
1.0
0
5
10
15
20
$R^2 = 0.285$
$p = 0.00048$

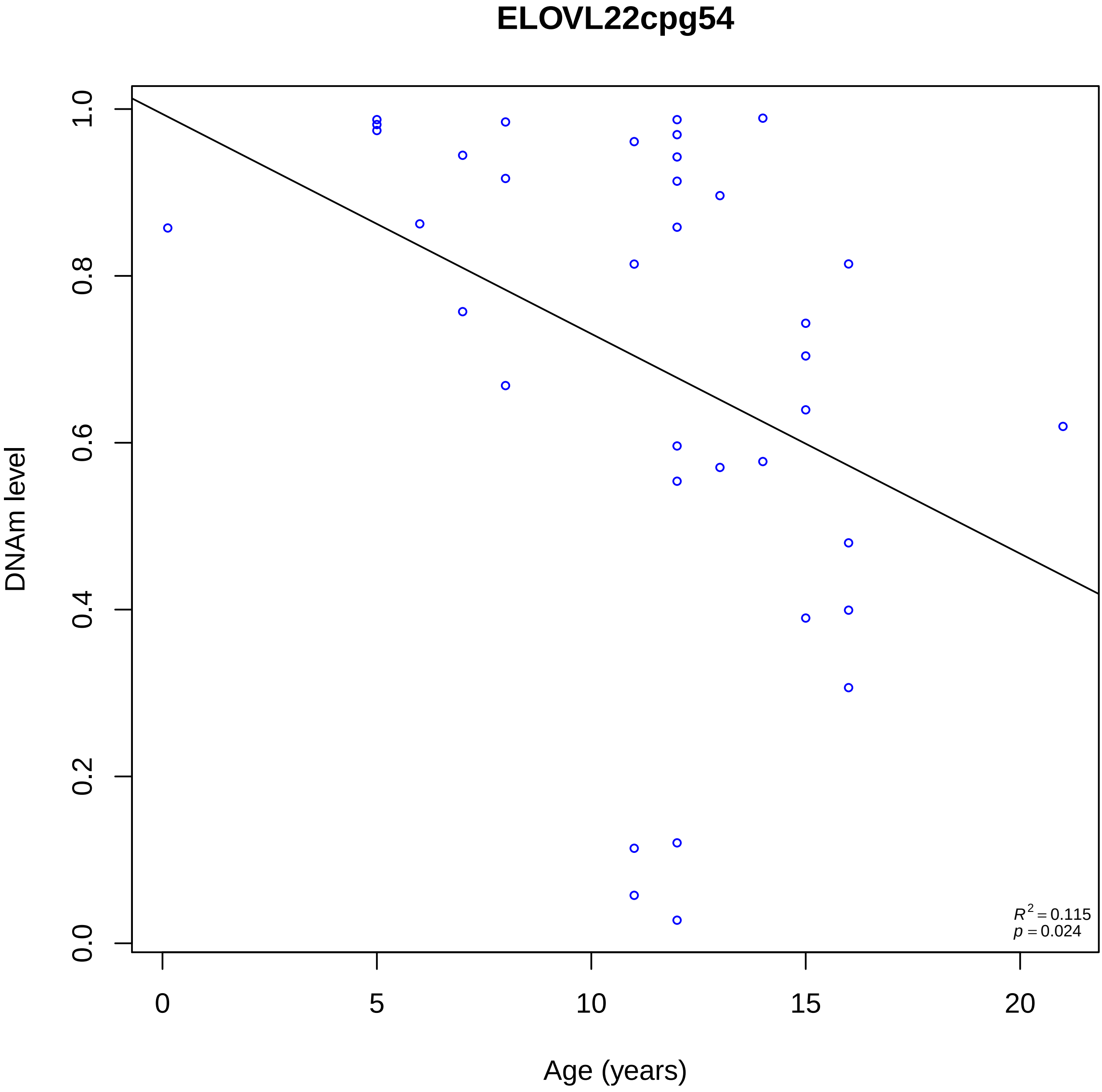
ELOVL22cpg54
DNAm level
0.0
0.2
0.4
0.6
0.8
1.0
0
5
10
15
20
Age (years)
$R^2 = 0.115$
$p = 0.024$

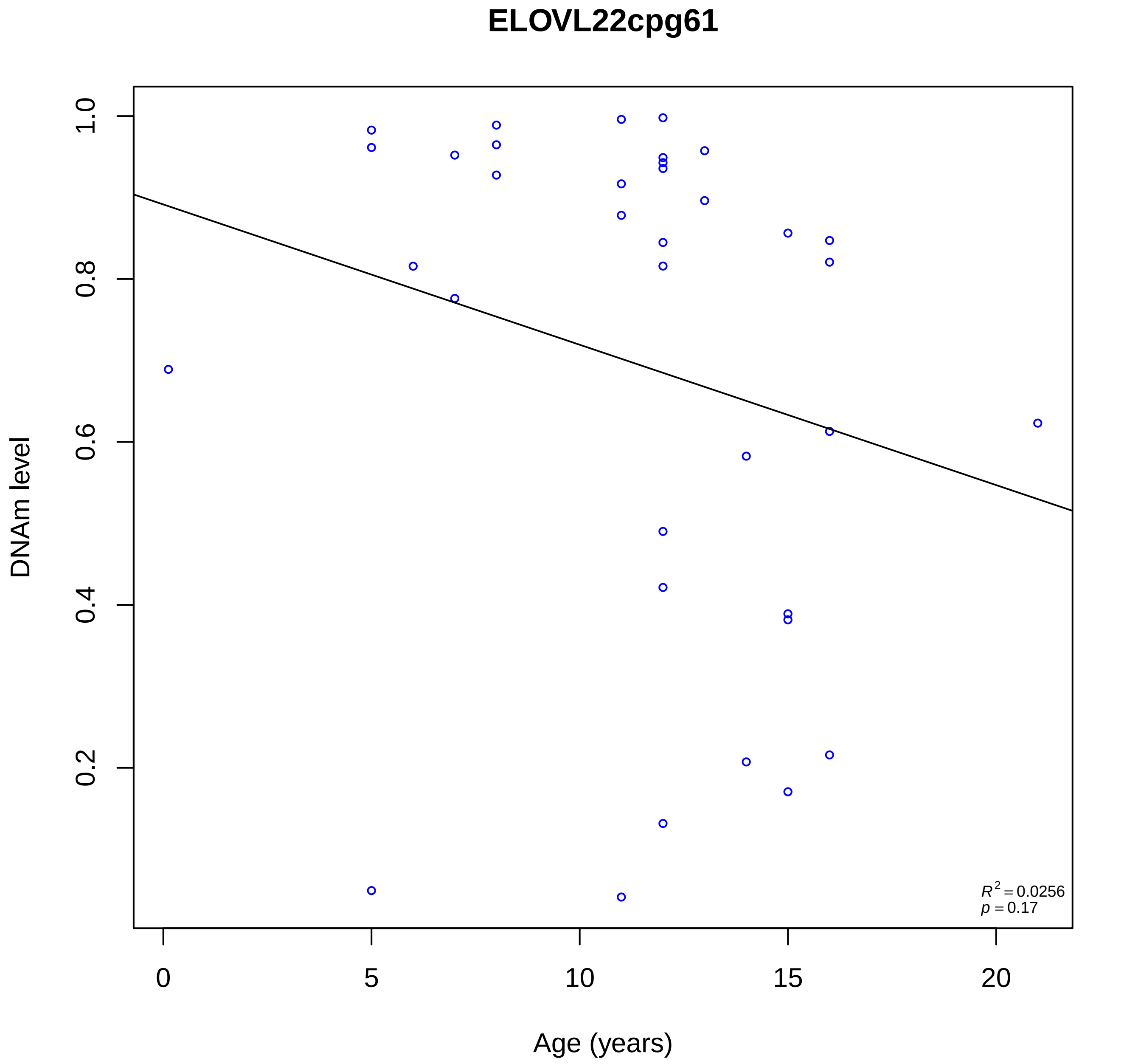

ELOVL22cpg61
DNAm level
1.0
0.8
0.6
0.4
0.2
0
5
10
15
20
Age (years)
$R^2 = 0.0256$
$p = 0.17$

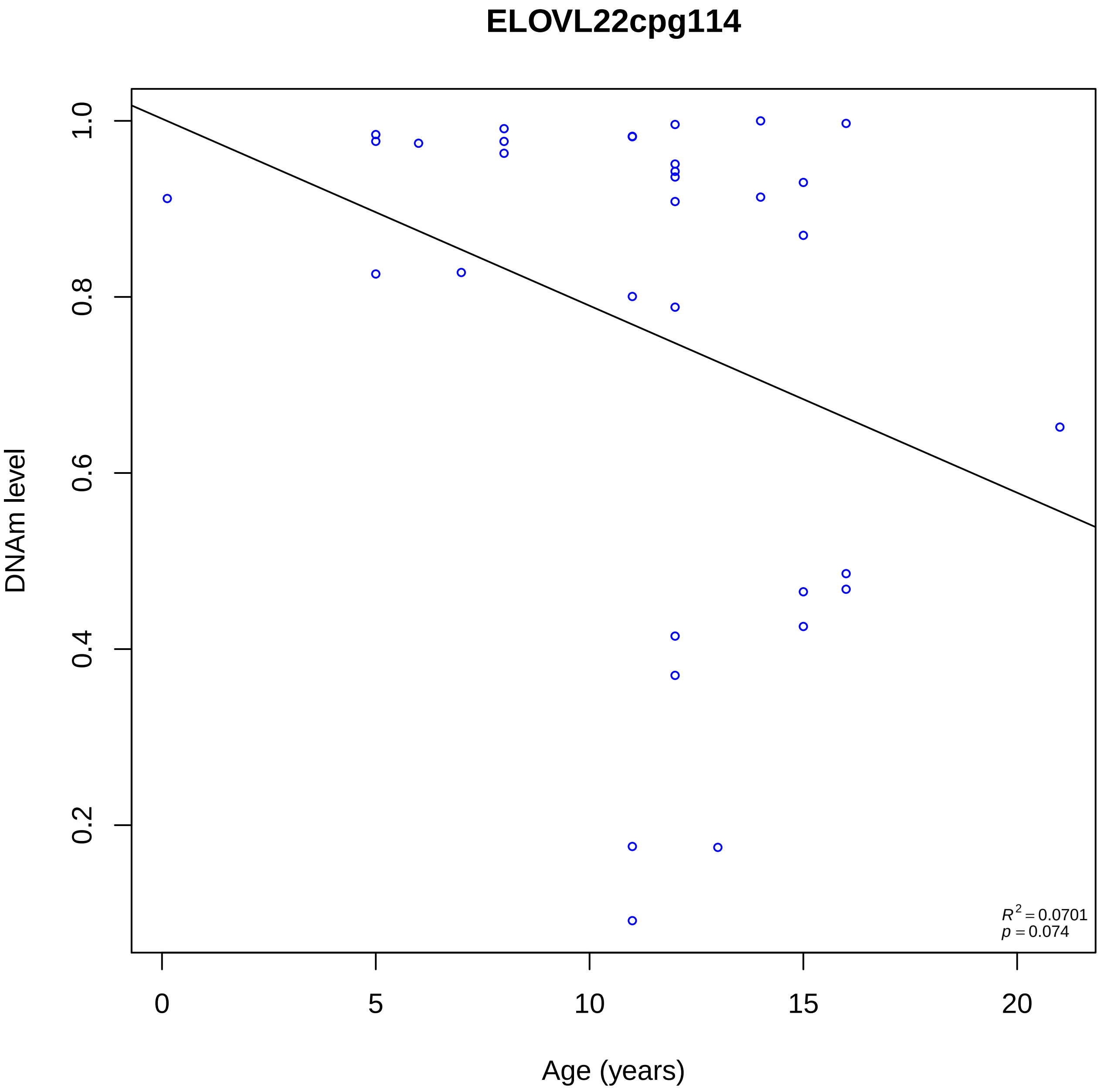
ELOVL22cpg114
DNAm level
Age (years)
0.2
0.4
0.6
0.8
1.0
0
5
10
15
20
$R^2 = 0.0701$
$p = 0.074$

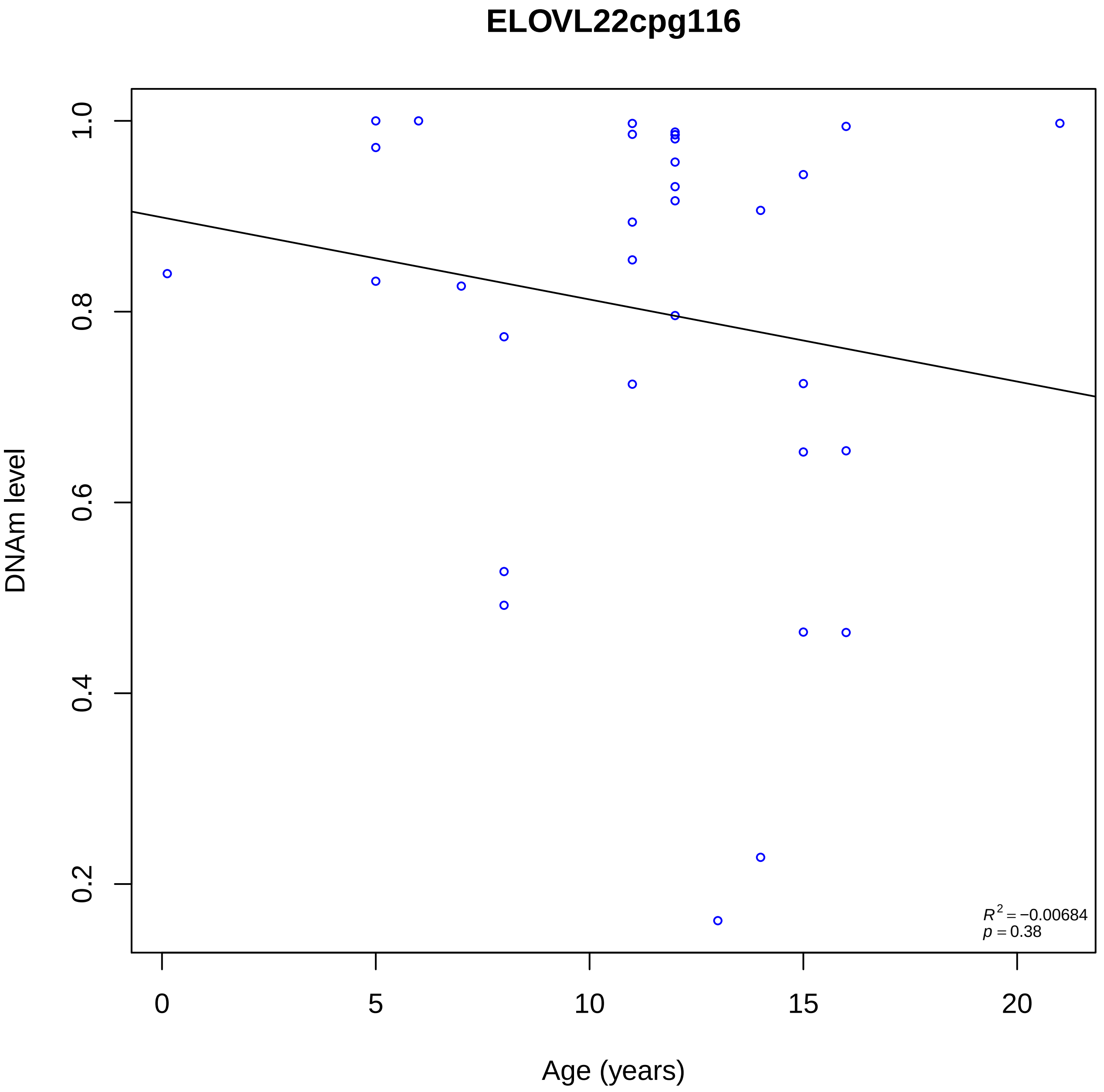

ELOVL22cpg116
1.0
0.8
0.6
0.4
0.2
DNAm level
0
5
10
15
20
Age (years)
R² = −0.00684
p = 0.38

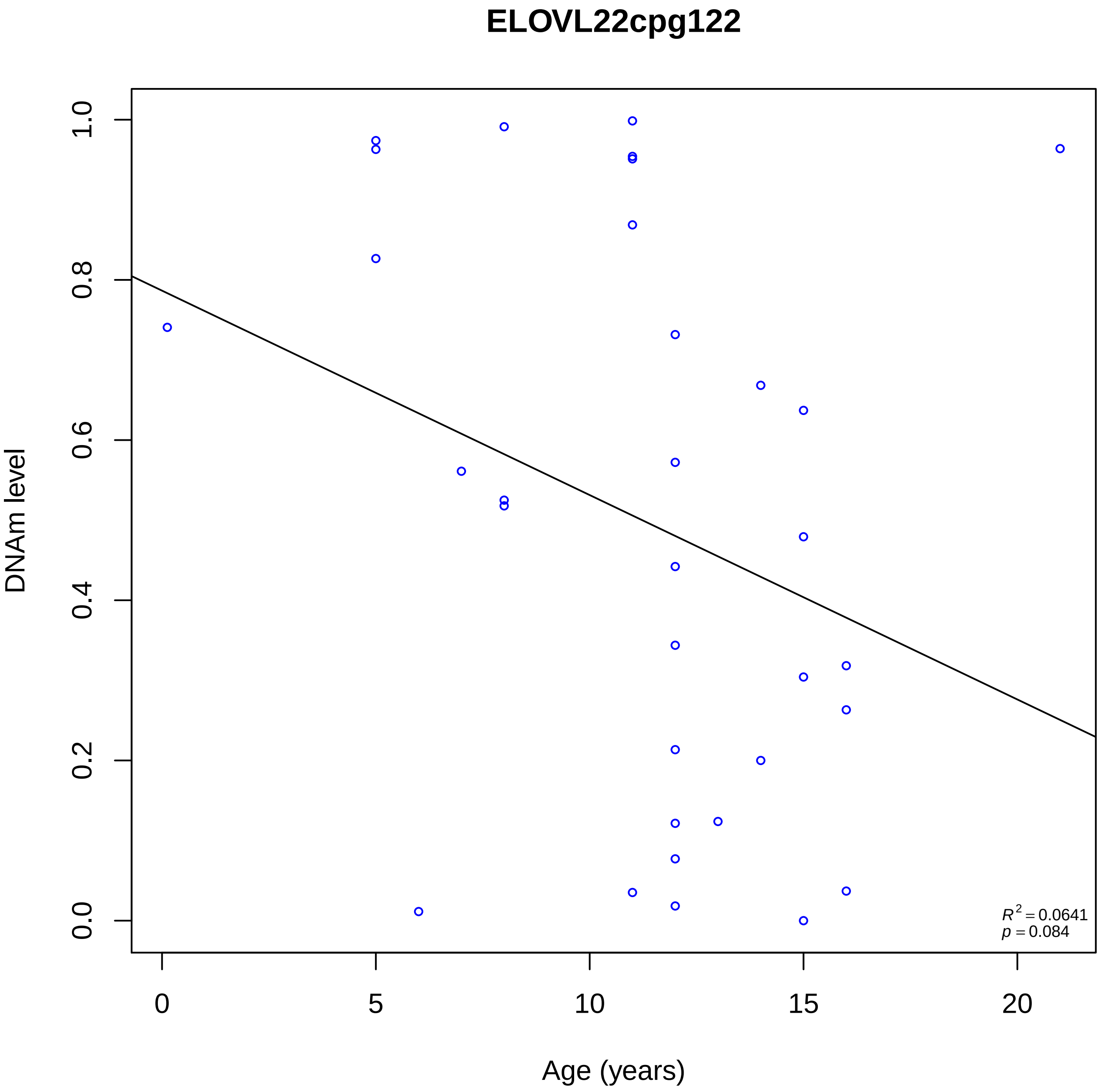

ELOVL22cpg122
DNAm level
0.0
0.2
0.4
0.6
0.8
1.0
0
5
10
15
20
Age (years)
$R^2 = 0.0641$
$p = 0.084$

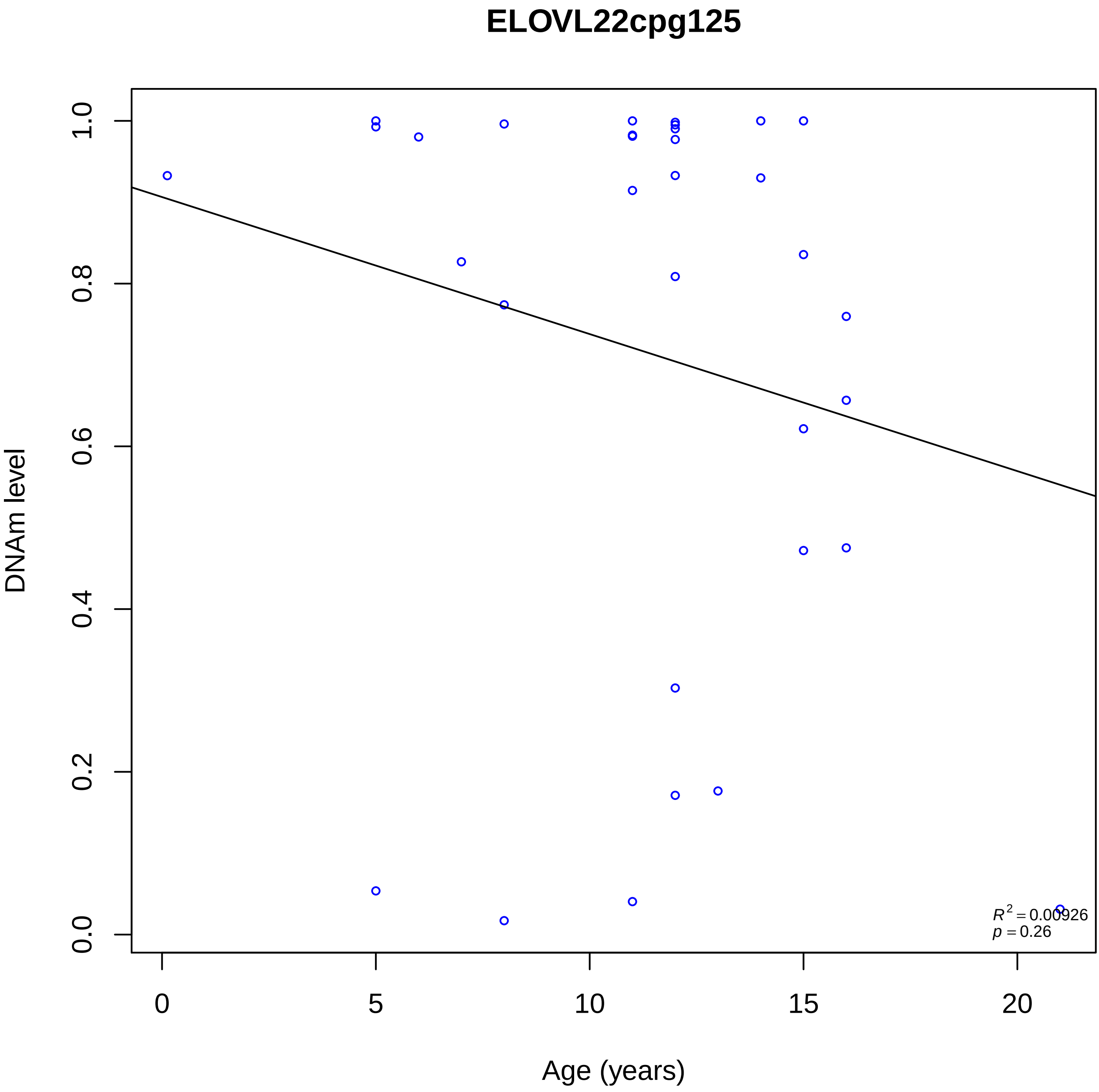
ELOVL22cpg125
DNAm level
0.0
0.2
0.4
0.6
0.8
1.0
0
5
10
15
20
Age (years)
$R^2 = 0.00926$
$p = 0.26$

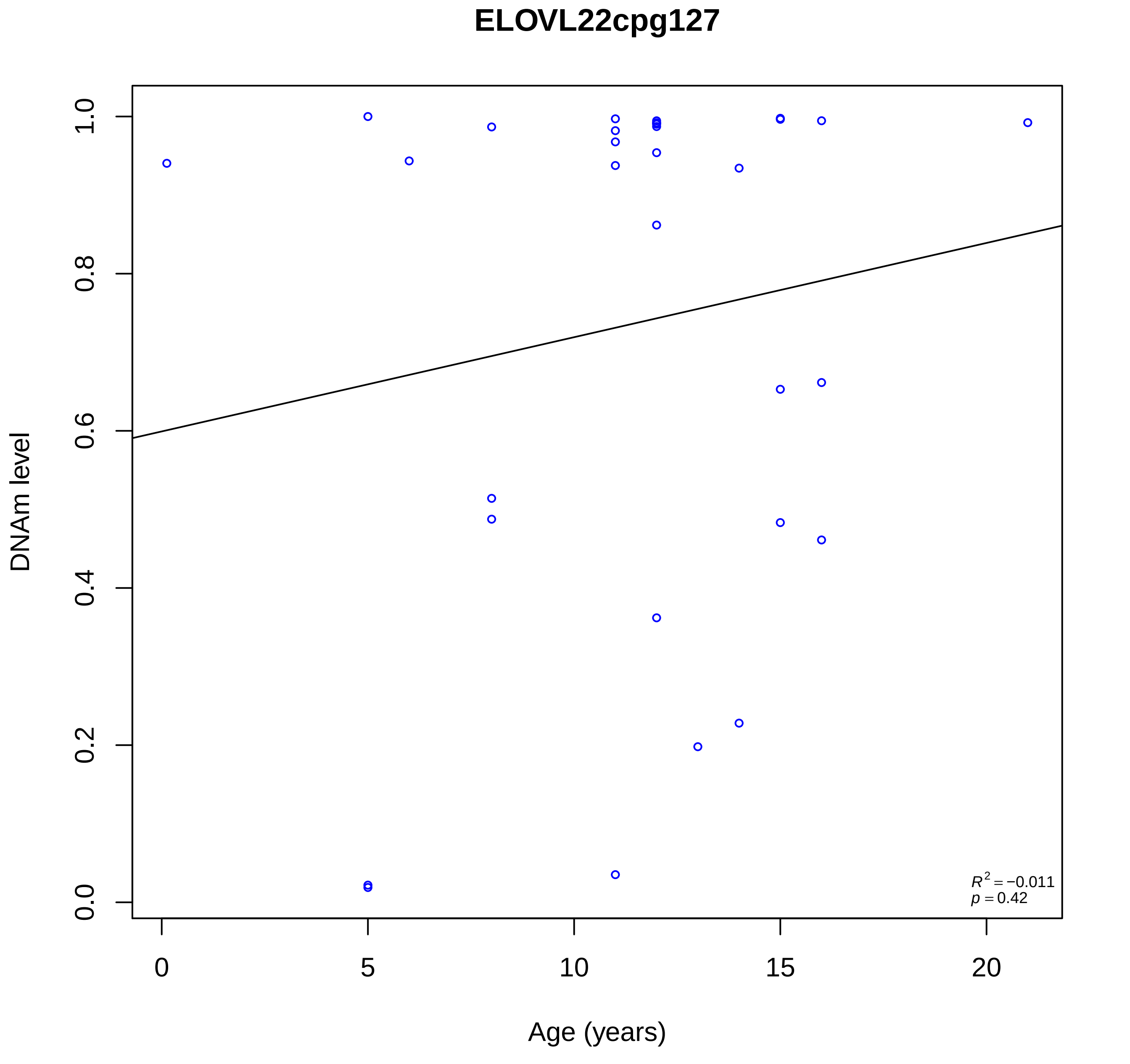
ELOVL22cpg127
DNAm level
Age (years)
0.0
0.2
0.4
0.6
0.8
1.0
0
5
10
15
20
$R^2 = -0.011$
$p = 0.42$

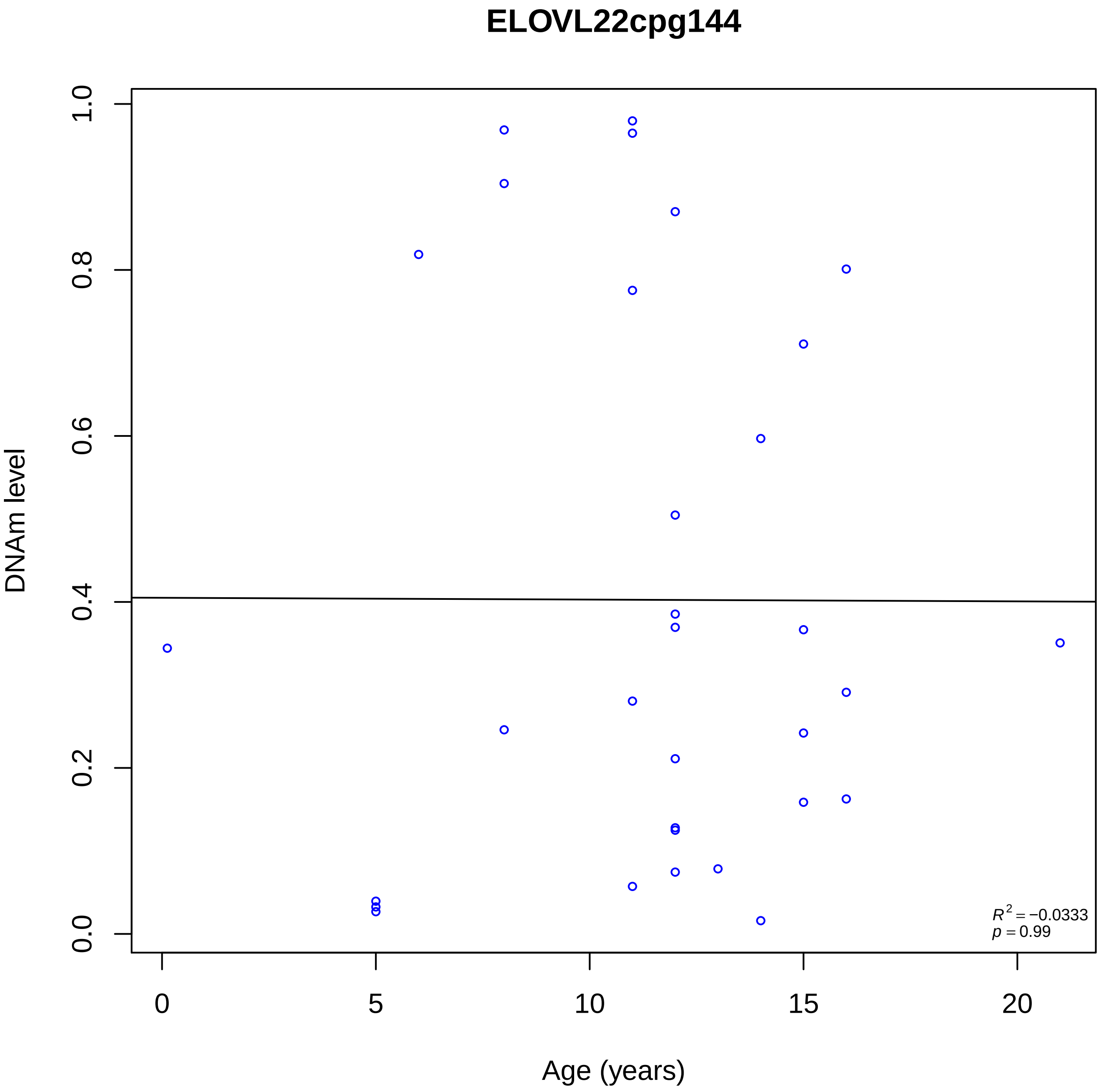
ELOVL22cpg144
DNAm level
Age (years)
0.0
0.2
0.4
0.6
0.8
1.0
0
5
10
15
20
$R^2 = -0.0333$
$p = 0.99$

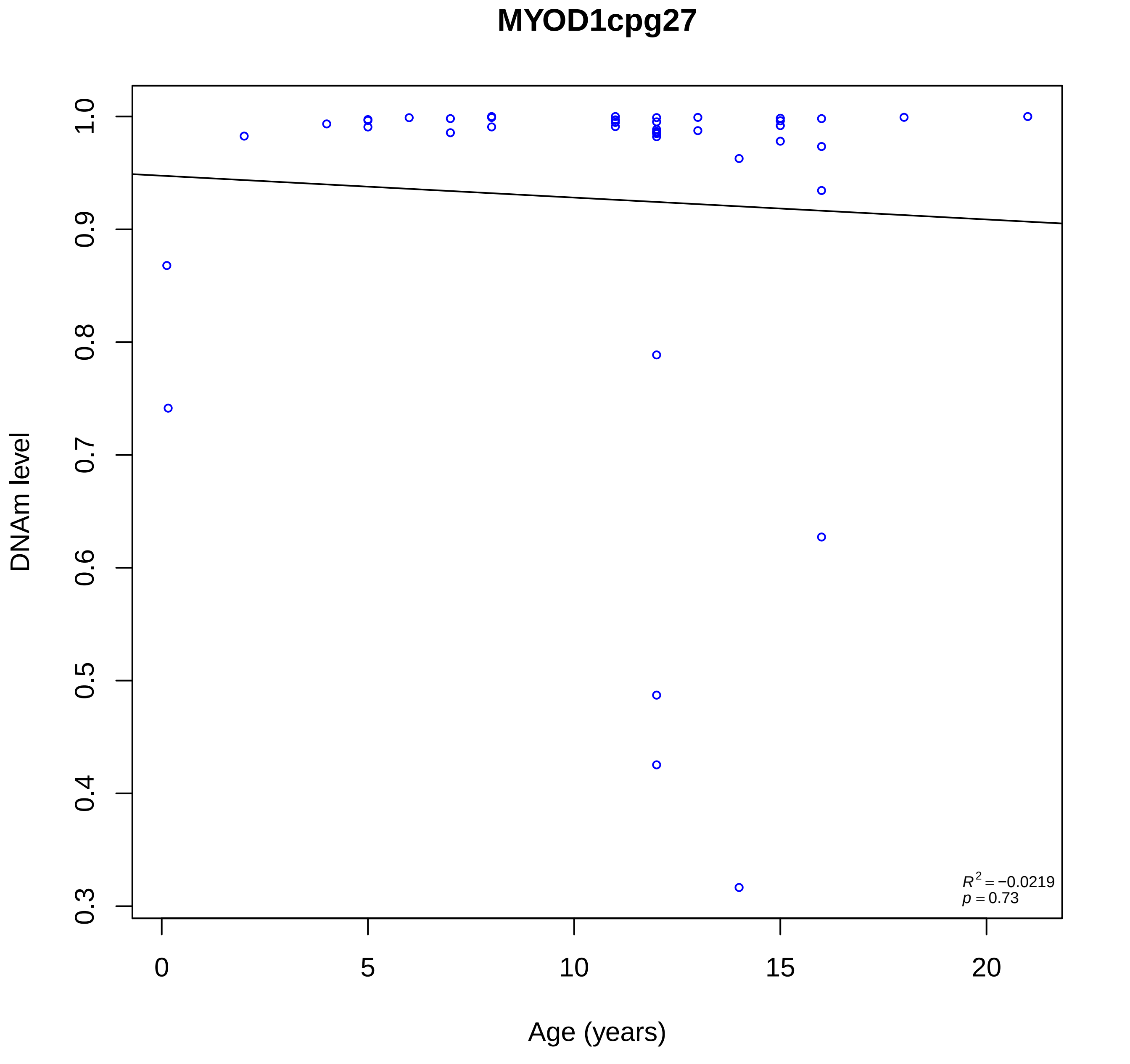
MYOD1cpg27
DNAm level
Age (years)
0.3
0.4
0.5
0.6
0.7
0.8
0.9
1.0
0
5
10
15
20
$R^2 = -0.0219$
$p = 0.73$

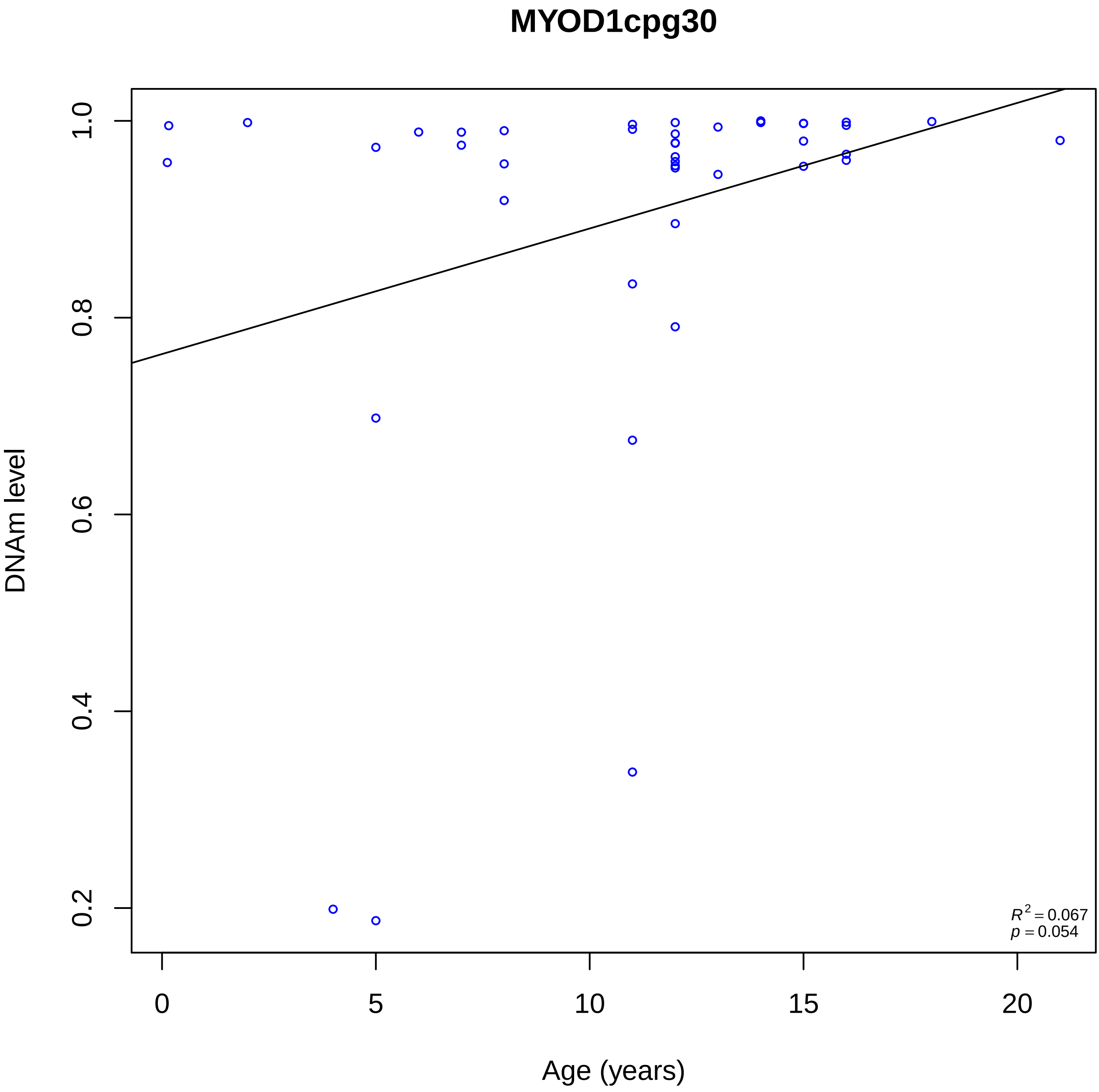
MYOD1cpg30
DNAm level
0.2
0.4
0.6
0.8
1.0
0
5
10
15
20
Age (years)
$R^2 = 0.067$
$p = 0.054$

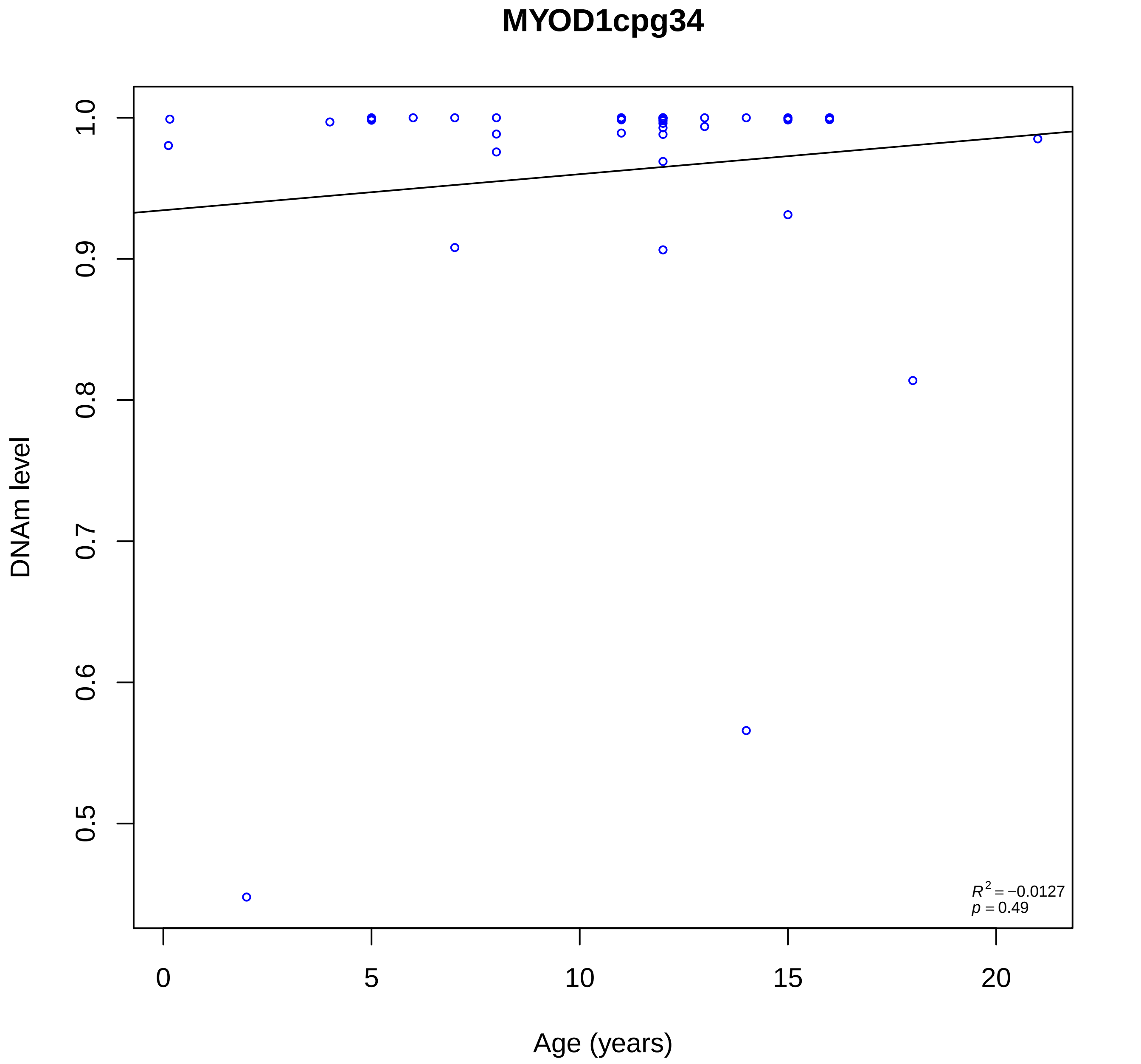

MYOD1cpg34
DNAm level
1.0
0.9
0.8
0.7
0.6
0.5
0
5
10
15
20
Age (years)
$R^2 = -0.0127$
$p = 0.49$

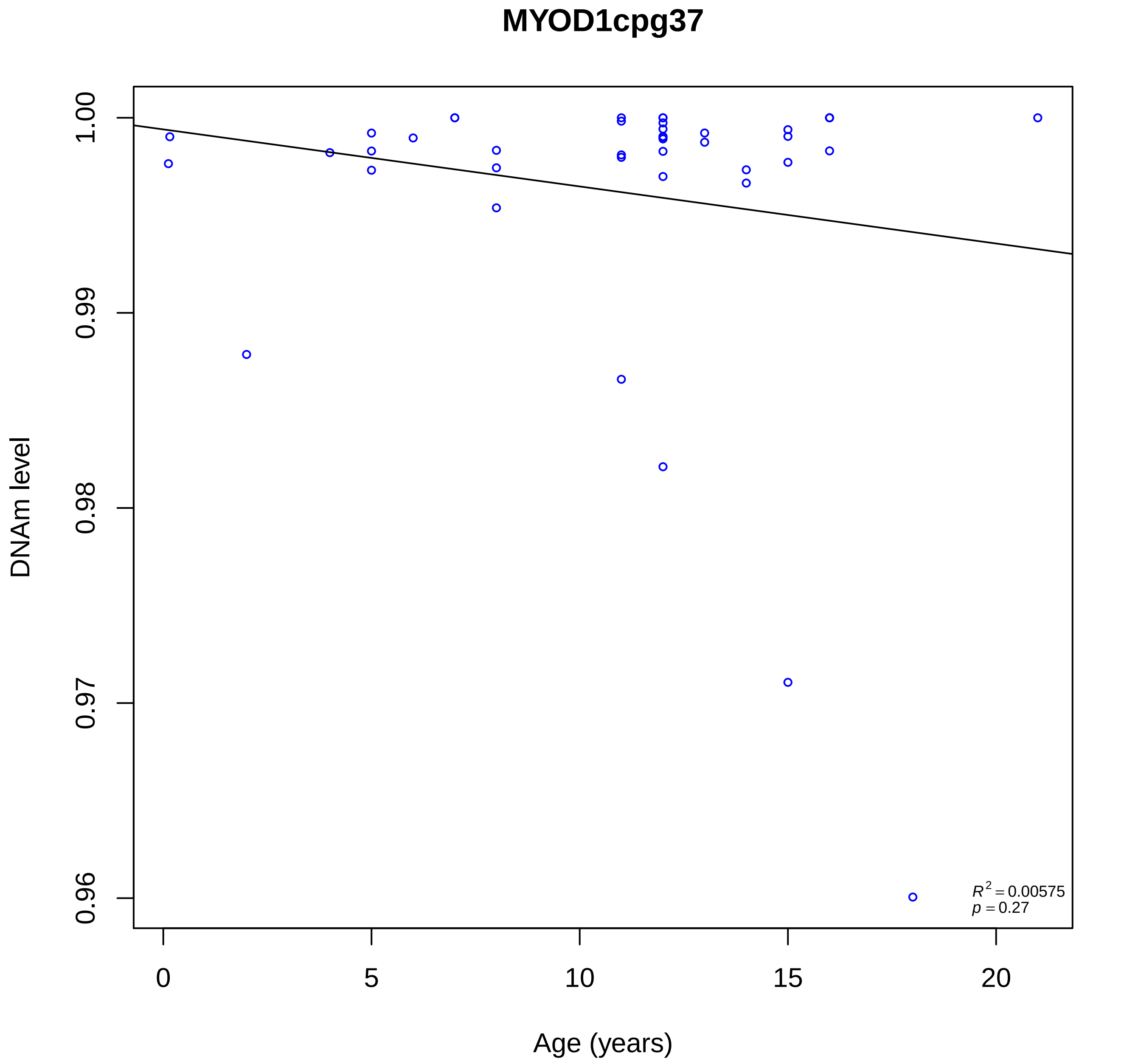
MYOD1cpg37
DNAm level
1.00
0.99
0.98
0.97
0.96
0
5
10
15
20
Age (years)
$R^2 = 0.00575$
$p = 0.27$

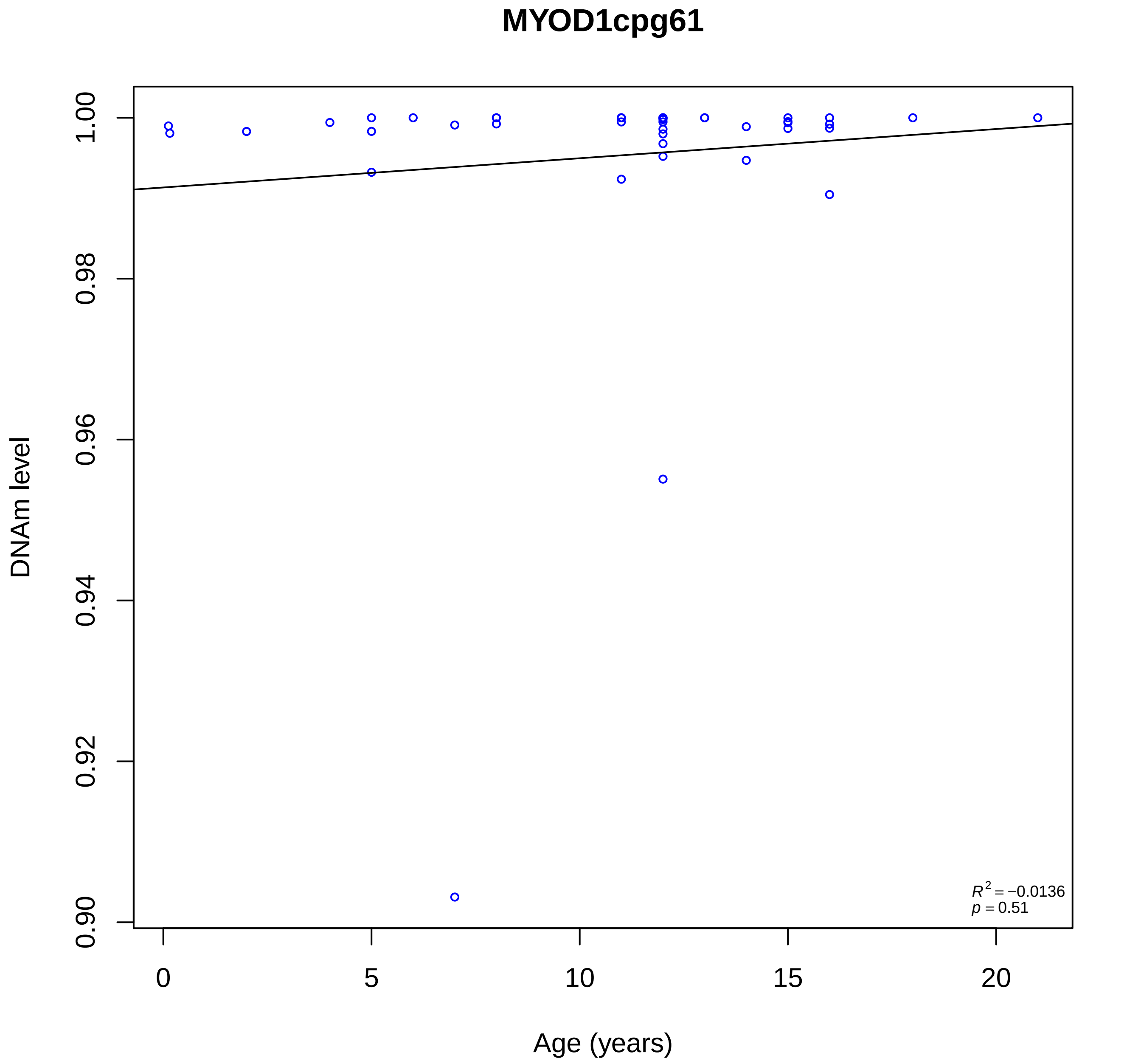
MYOD1cpg61
DNAm level
1.00
0.98
0.96
0.94
0.92
0.90
0
5
10
15
20
Age (years)
R2 = −0.0136
p = 0.51

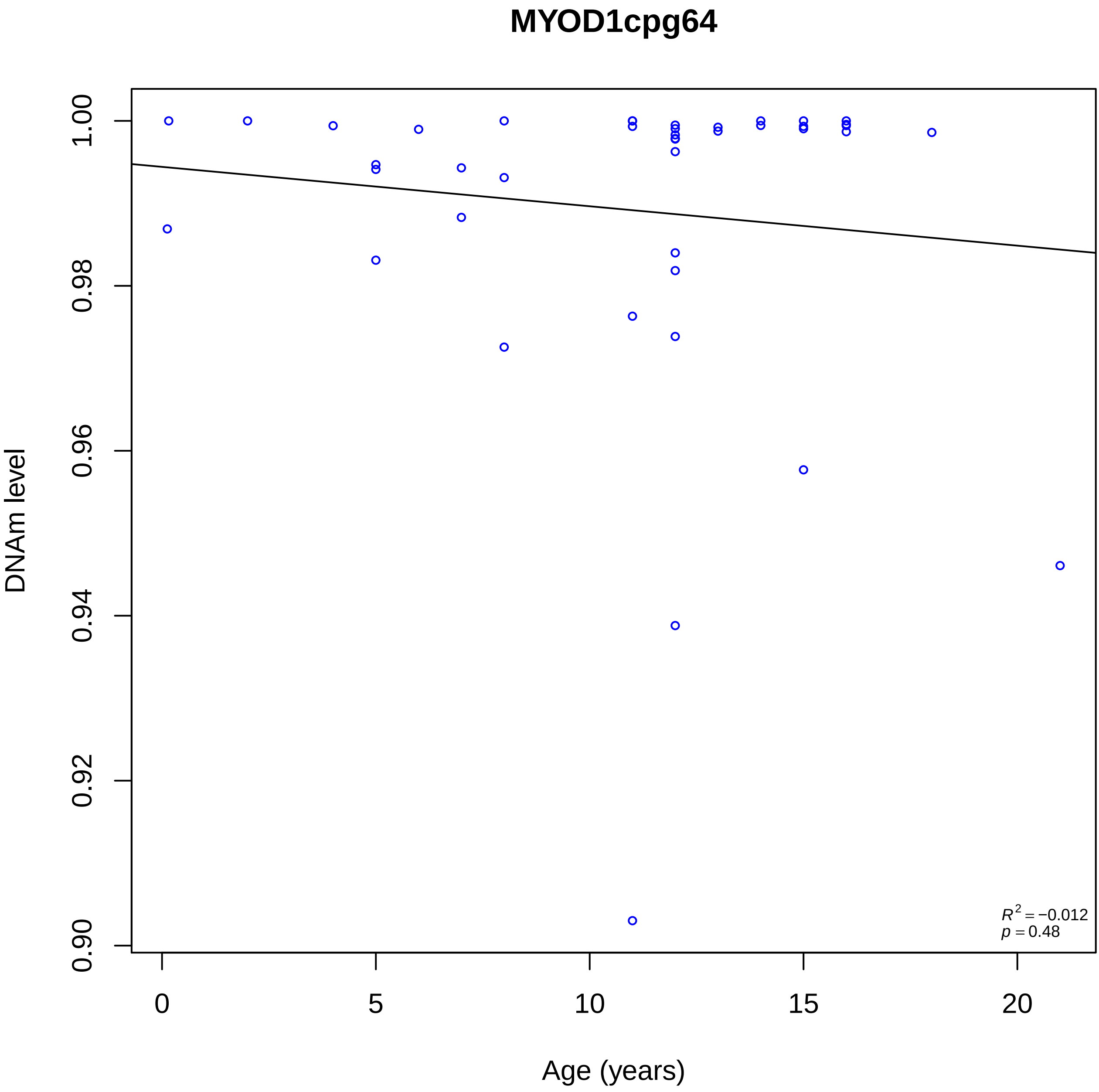

MYOD1cpg64
DNAm level
1.00
0.98
0.96
0.94
0.92
0.90
0
5
10
15
20
Age (years)
$R^2 = -0.012$
$p = 0.48$

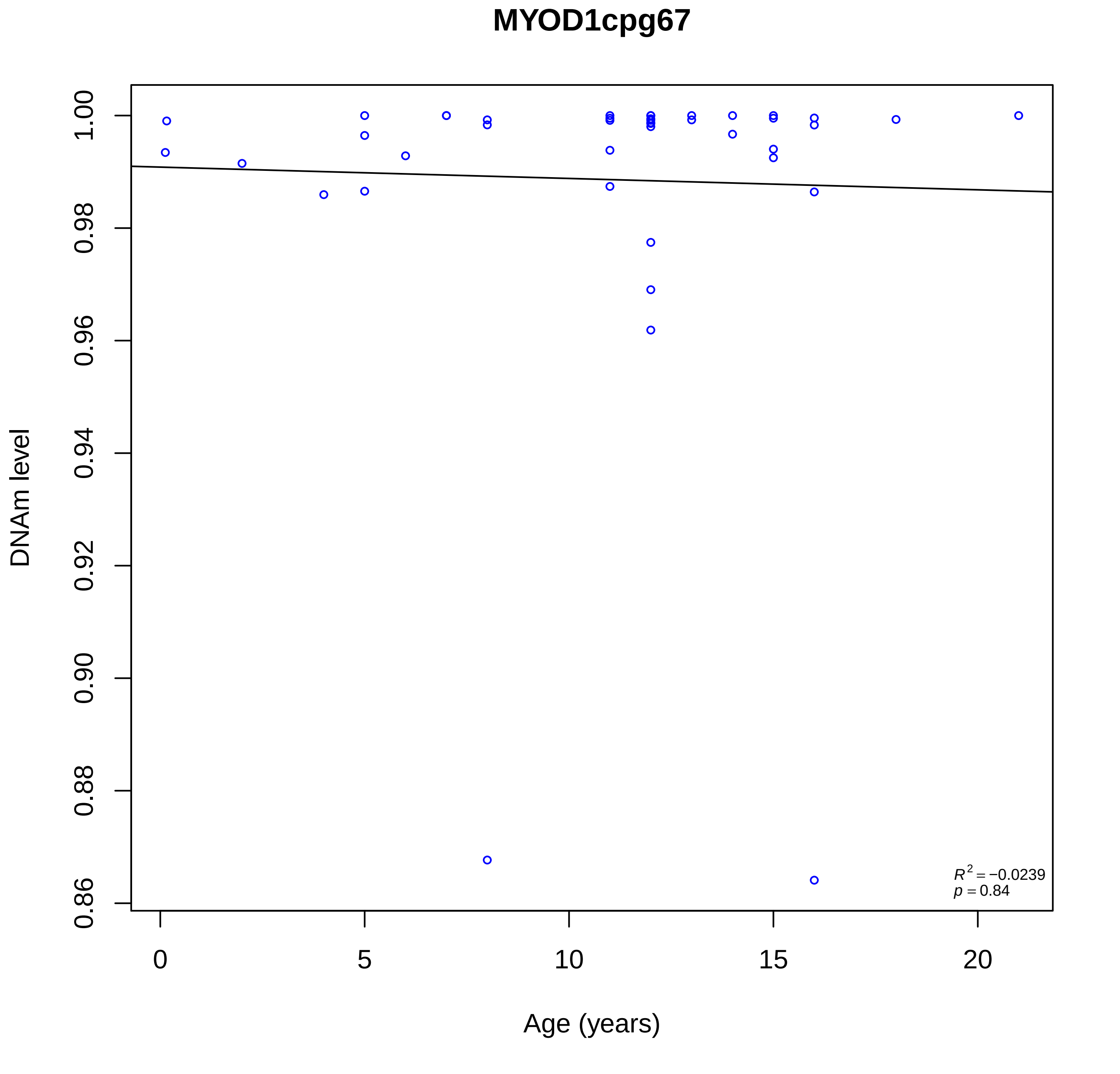

MYOD1cpg67
DNAm level
Age (years)
0.86
0.88
0.90
0.92
0.94
0.96
0.98
1.00
0
5
10
15
20
$R^2 = -0.0239$
$p = 0.84$

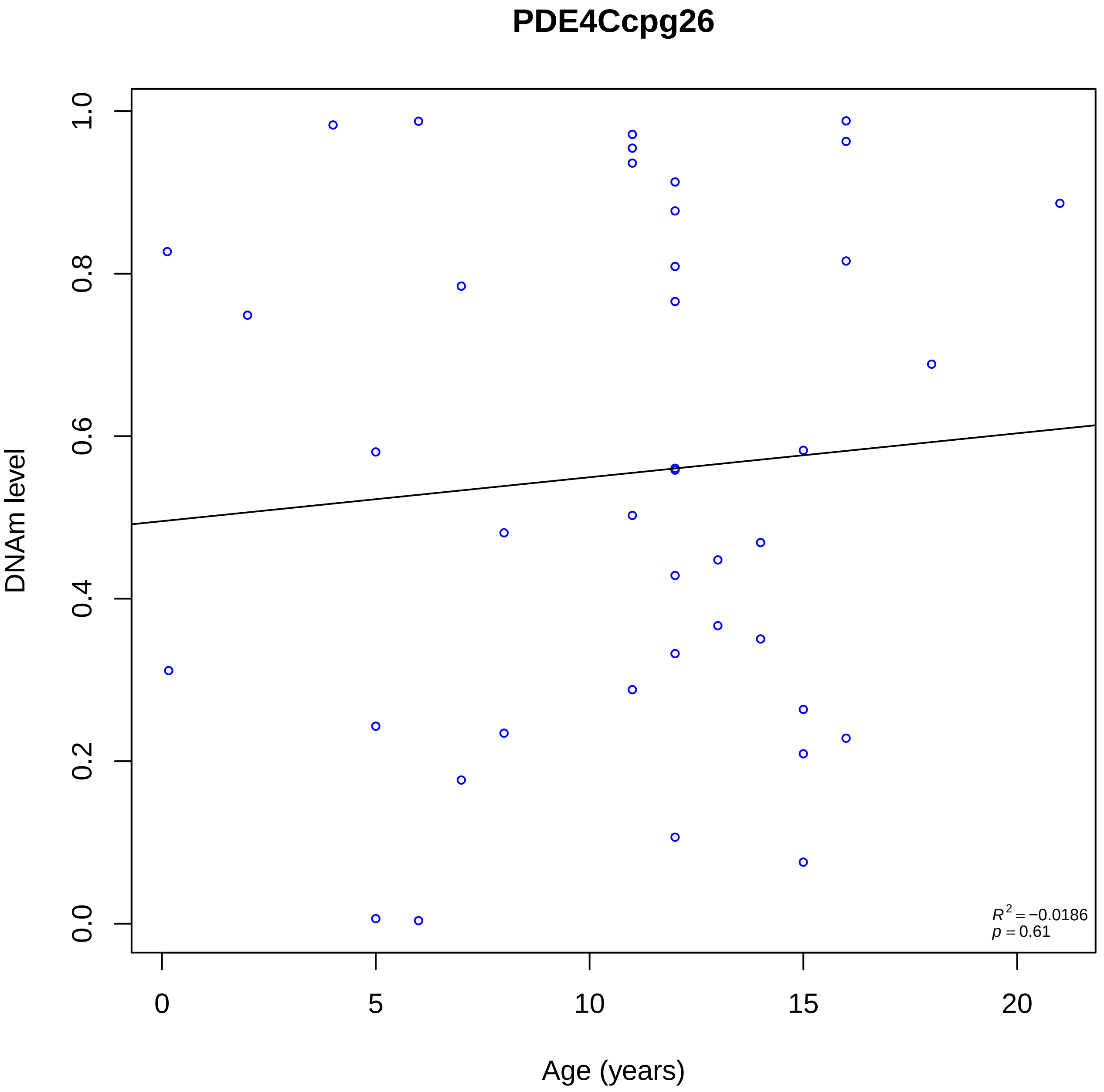

PDE4Ccpg26
DNAm level
Age (years)
0.0
0.2
0.4
0.6
0.8
1.0
0
5
10
15
20
$R^2 = -0.0186$
$p = 0.61$

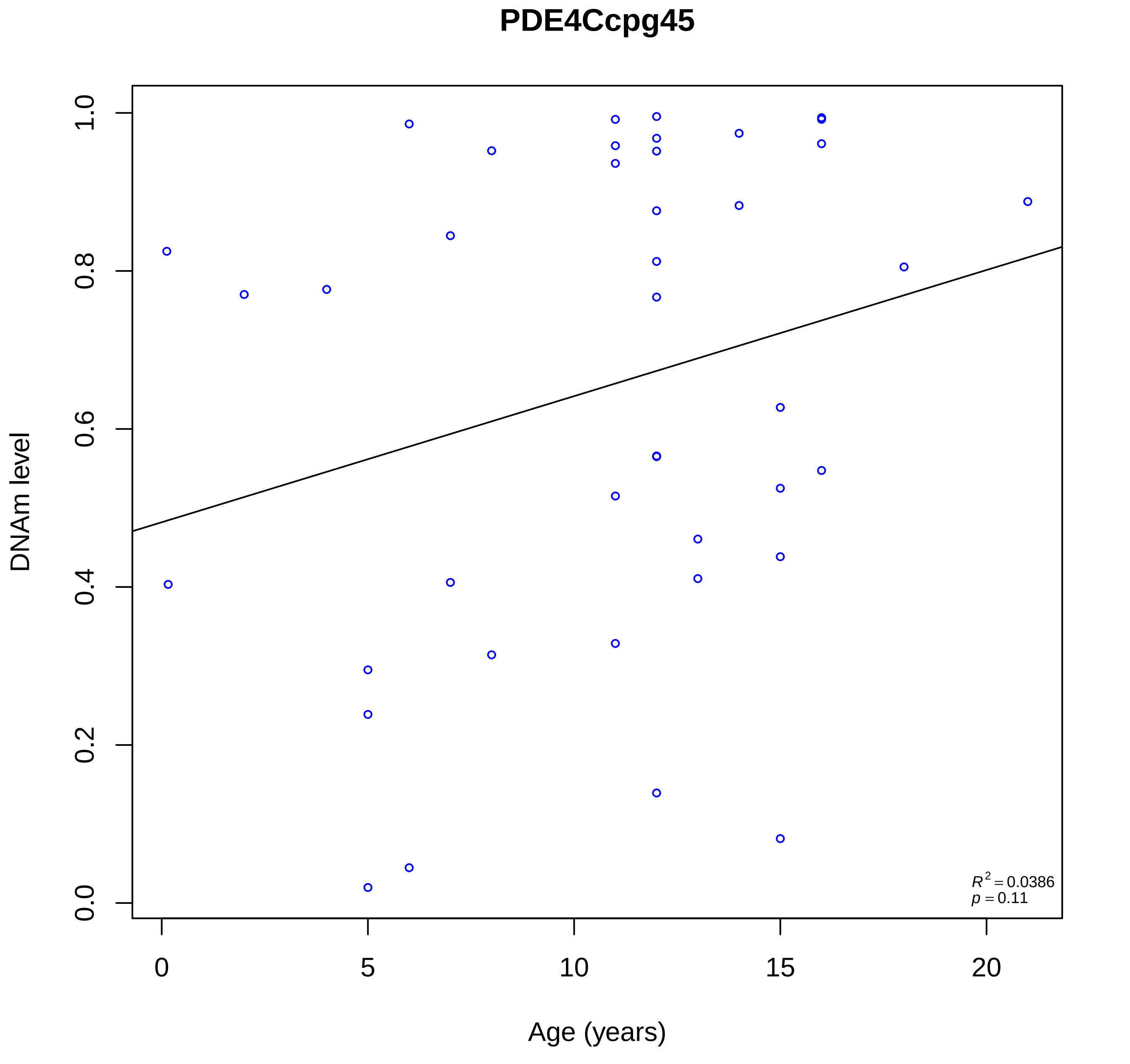

PDE4Ccpg45
DNAm level
0.0
0.2
0.4
0.6
0.8
1.0
0
5
10
15
20
Age (years)
$R^2 = 0.0386$
$p = 0.11$

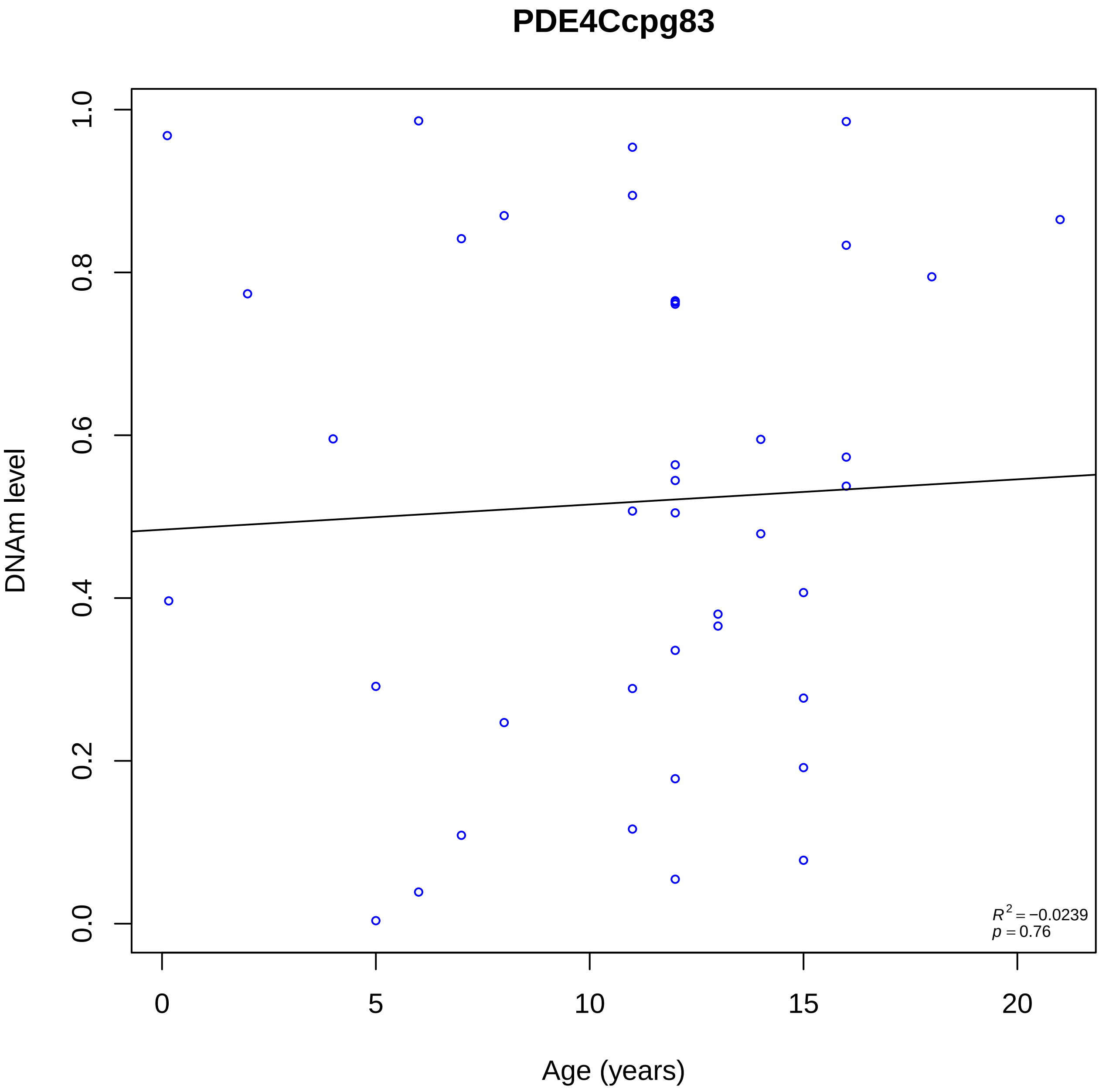
PDE4Ccpg83
DNAm level
0.0
0.2
0.4
0.6
0.8
1.0
0
5
10
15
20
Age (years)
$R^2 = -0.0239$
$p = 0.76$

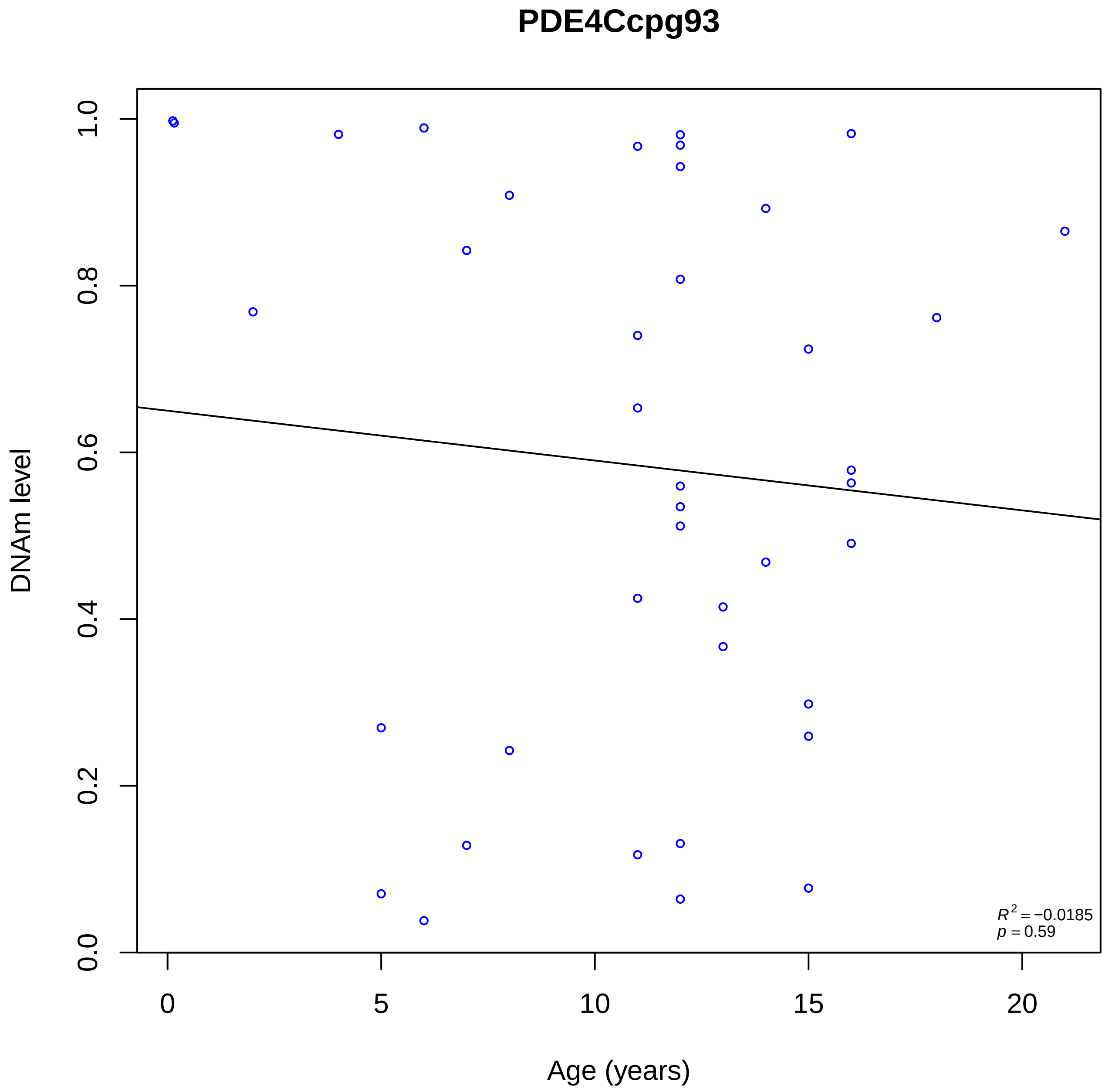

PDE4Ccpg93
DNAm level
Age (years)
0.0
0.2
0.4
0.6
0.8
1.0
0
5
10
15
20
$R^2 = -0.0185$
$p = 0.59$

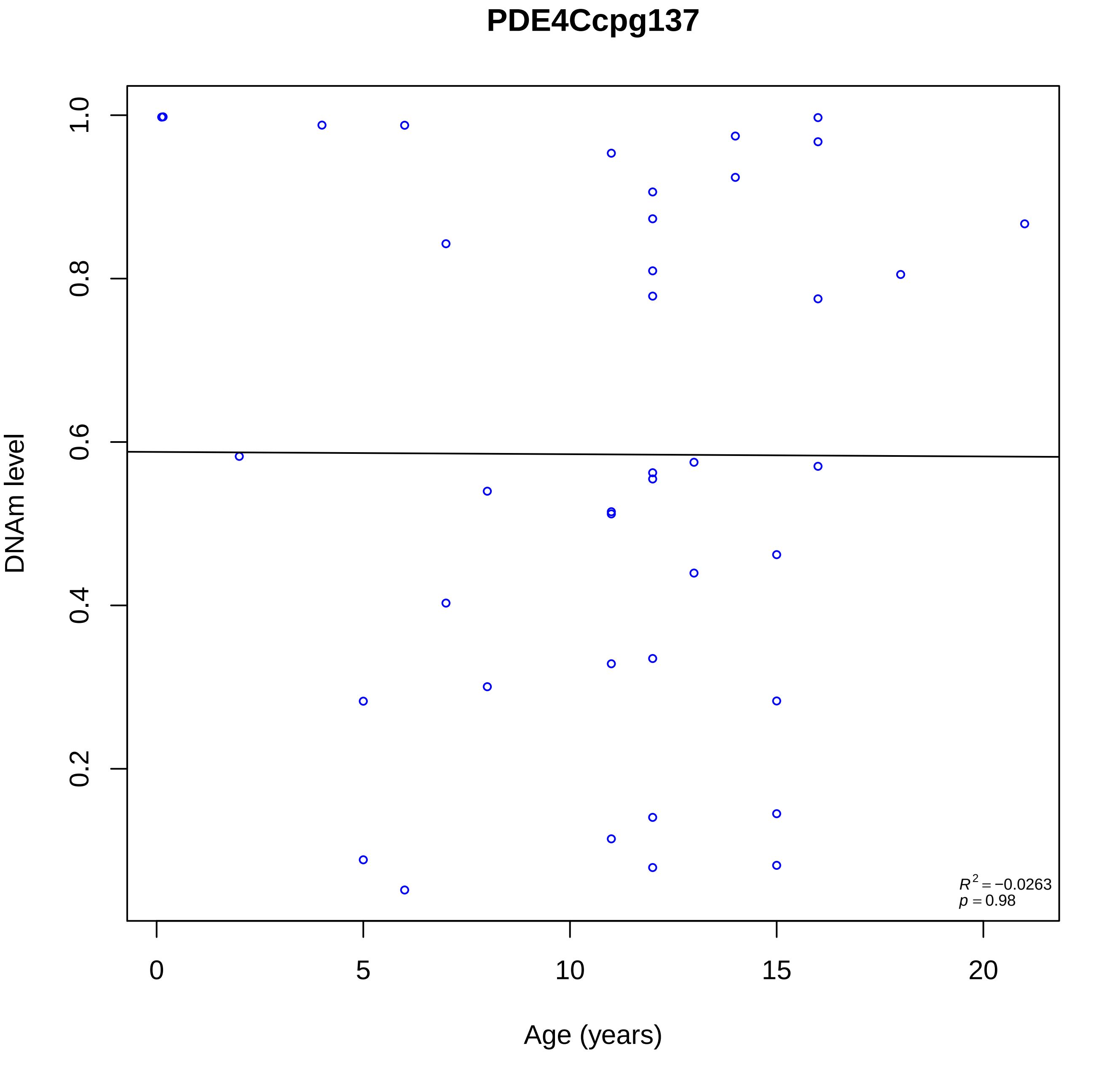

PDE4Ccpg137
DNAm level
Age (years)
0.2
0.4
0.6
0.8
1.0
0
5
10
15
20
$R^2 = -0.0263$
$p = 0.98$

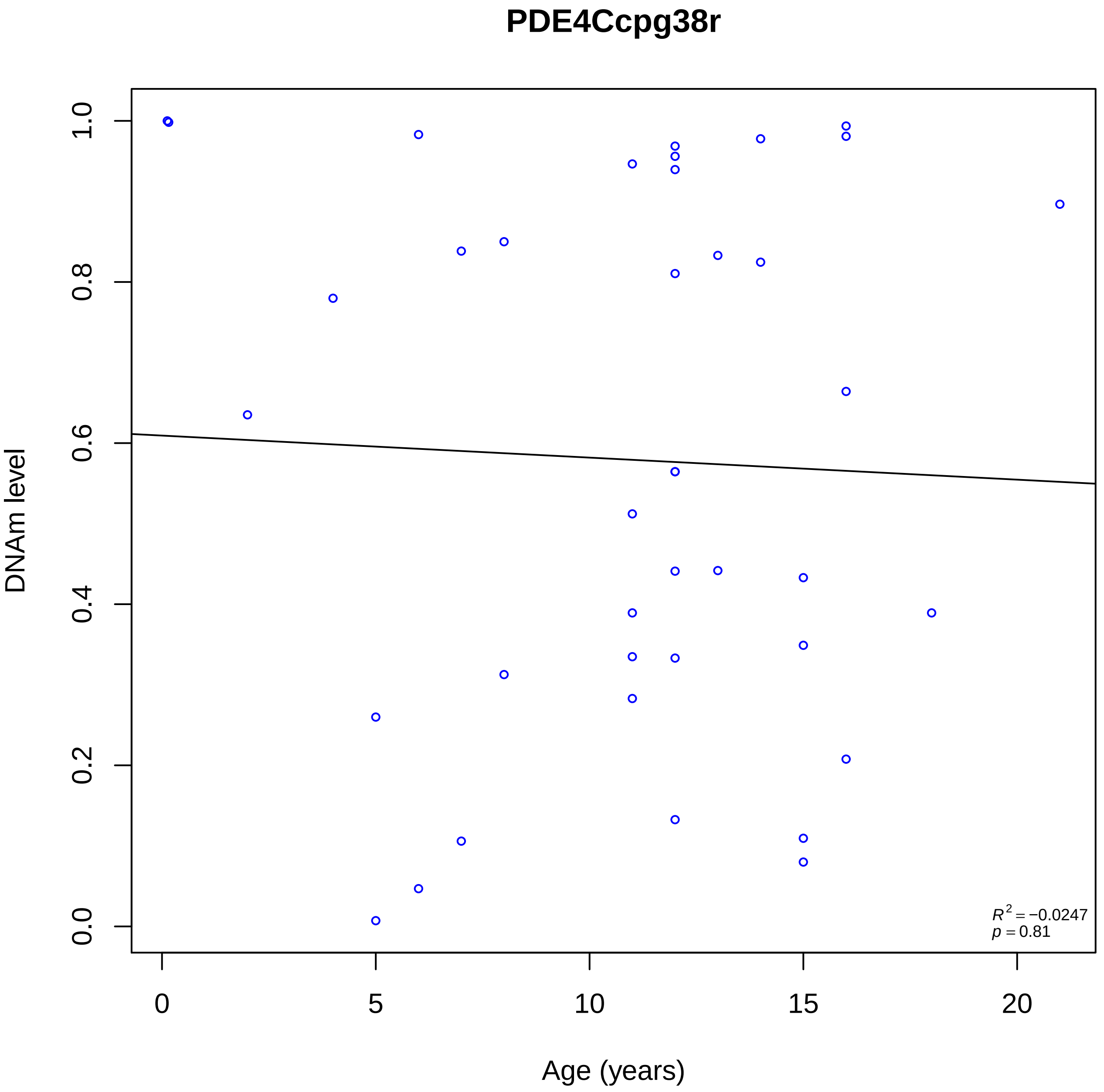

PDE4Ccpg38r
DNAm level
Age (years)
0.0
0.2
0.4
0.6
0.8
1.0
0
5
10
15
20
$R^2 = -0.0247$
$p = 0.81$

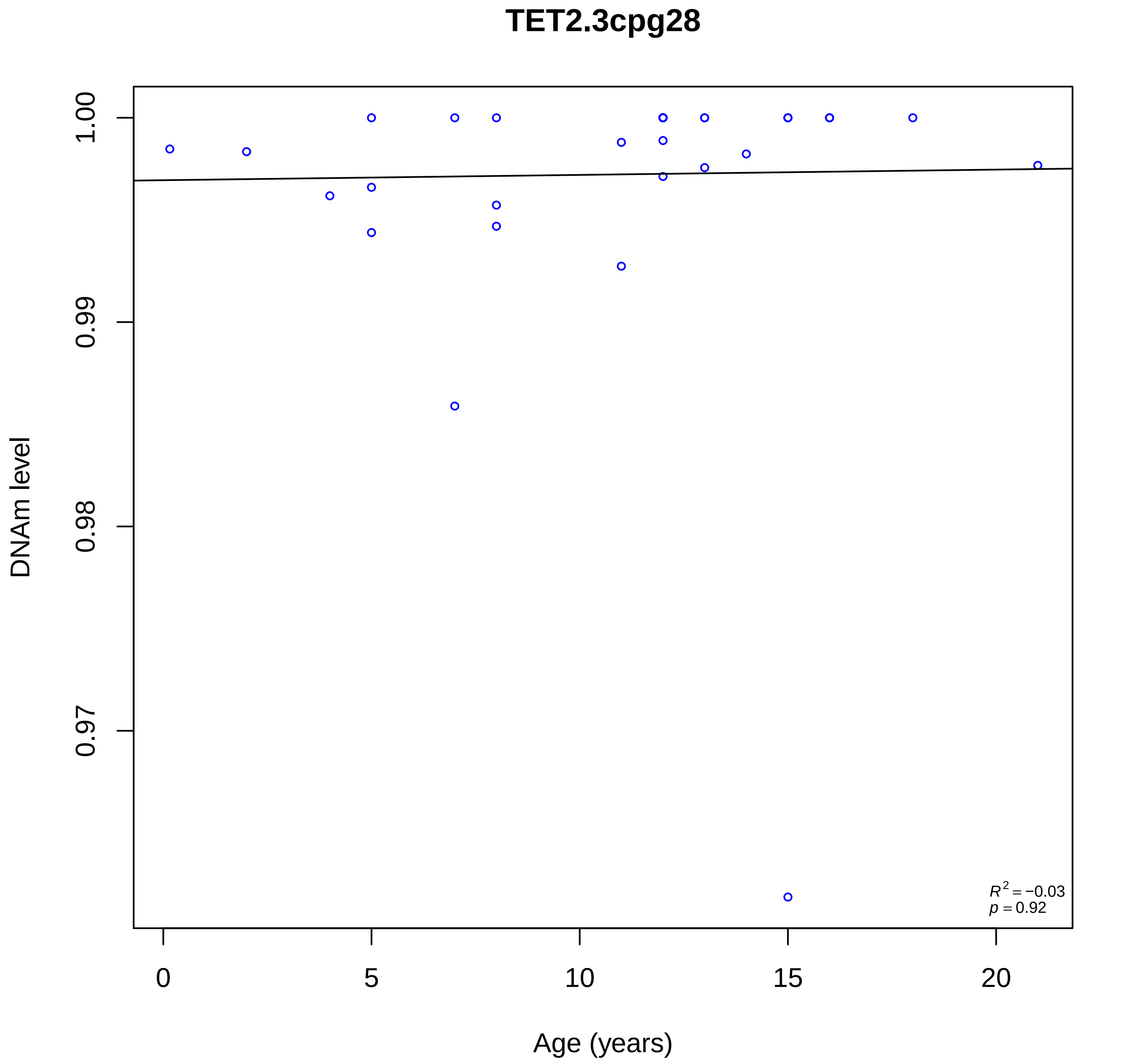
TET2.3cpg28
DNAm level
1.00
0.99
0.98
0.97
0
5
10
15
20
Age (years)
$R^2 = -0.03$
$p = 0.92$

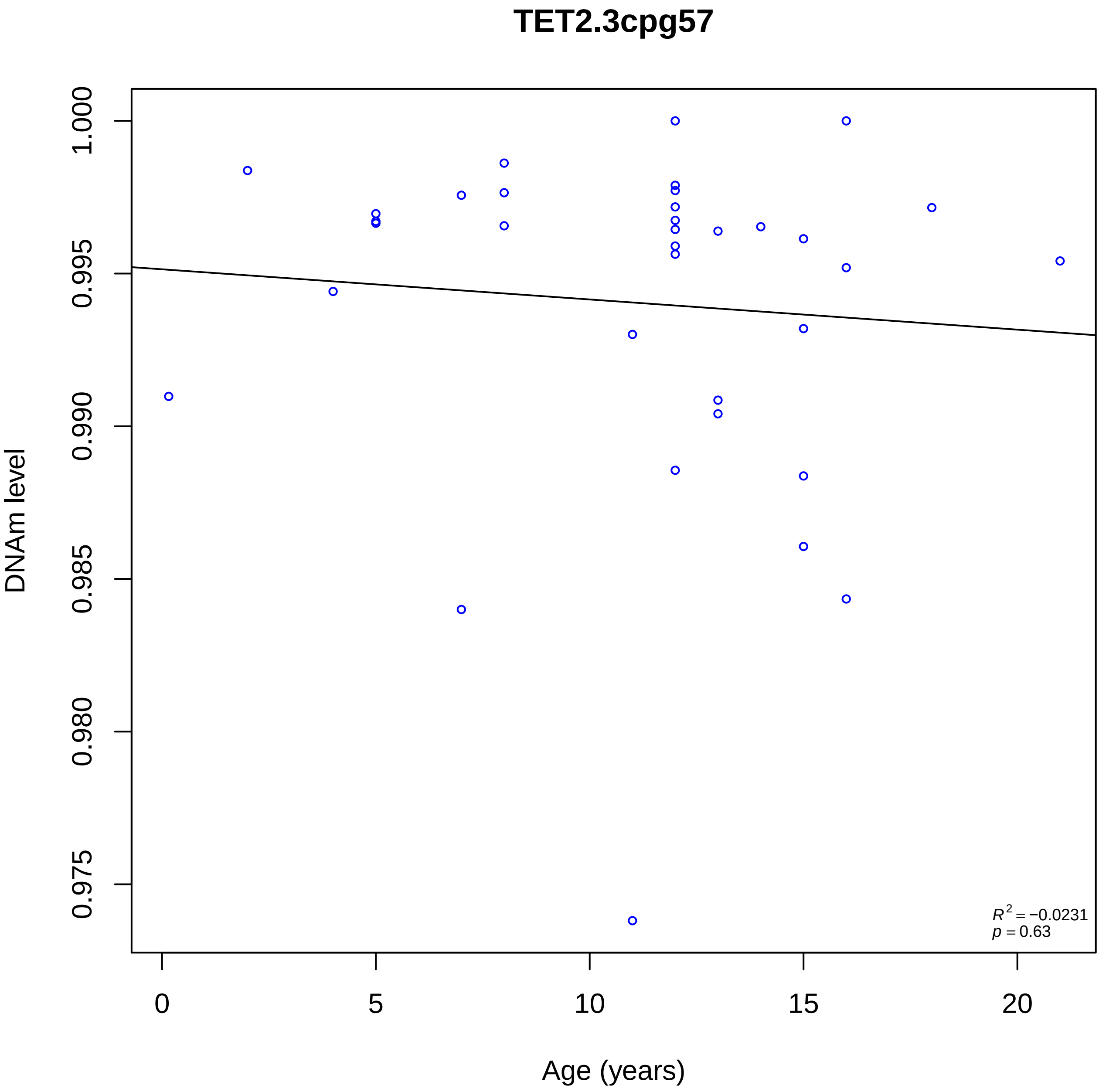

TET2.3cpg57
DNAm level
Age (years)
1.000
0.995
0.990
0.985
0.980
0.975
0
5
10
15
20
$R^2 = -0.0231$
$p = 0.63$

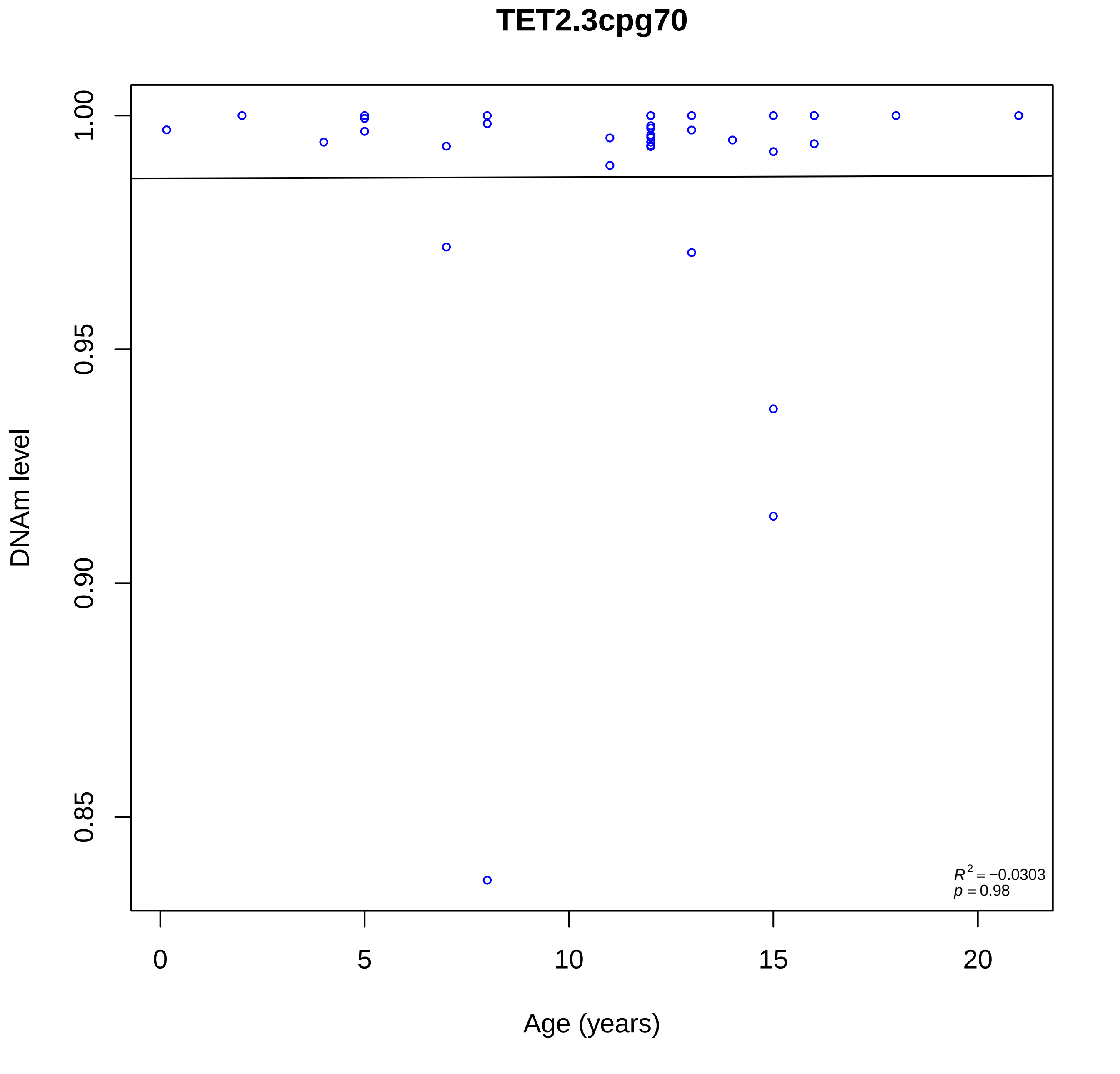

TET2.3cpg70
DNAm level
1.00
0.95
0.90
0.85
0
5
10
15
20
Age (years)
$R^2 = -0.0303$
$p = 0.98$

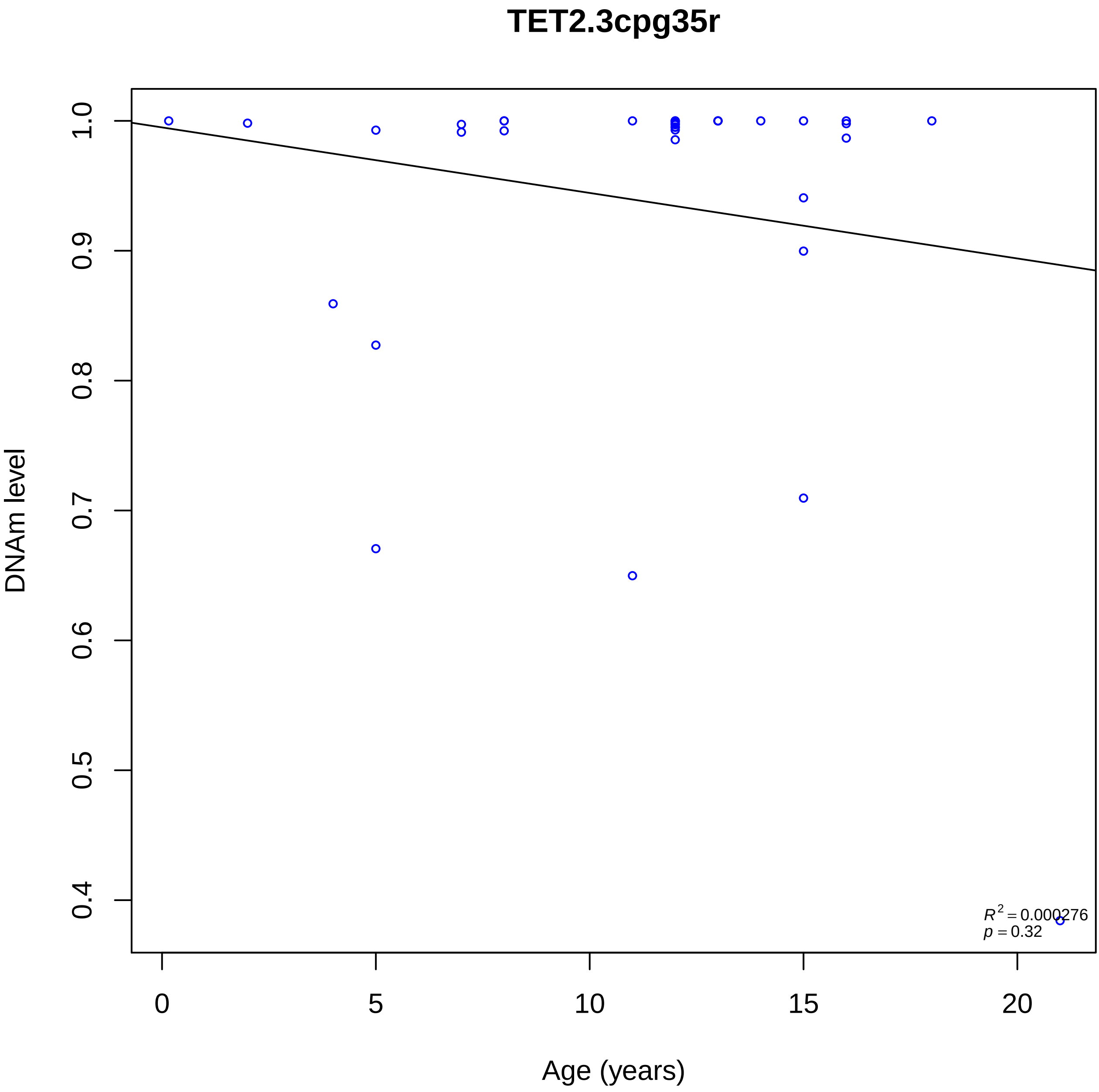

TET2.3cpg35r
DNAm level
Age (years)
1.0
0.9
0.8
0.7
0.6
0.5
0.4
0
5
10
15
20
$R^2 = 0.000276$
$p = 0.32$

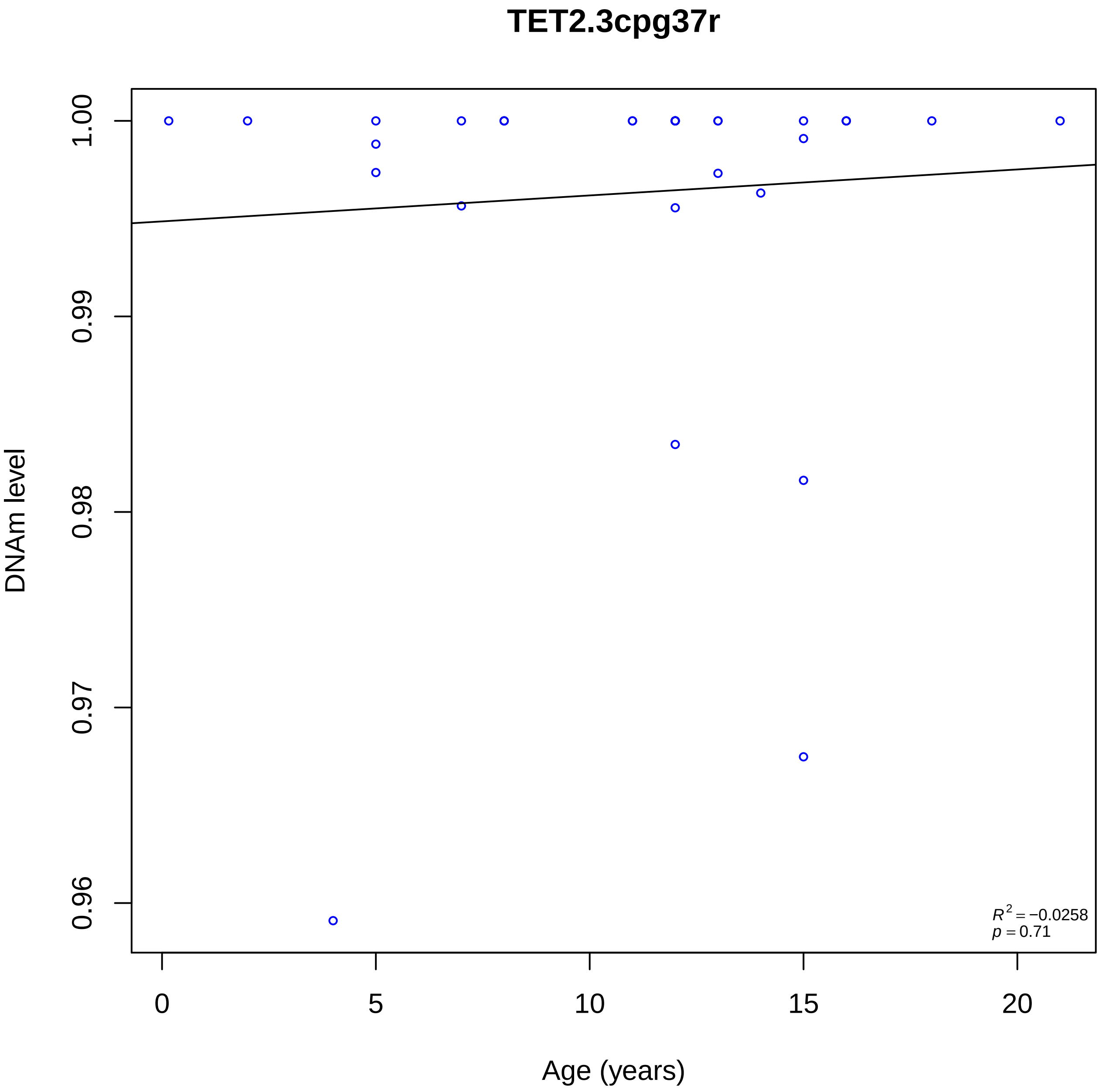

TET2.3cpg37r
DNAm level
1.00
0.99
0.98
0.97
0.96
Age (years)
0
5
10
15
20
R2 = −0.0258
p = 0.71

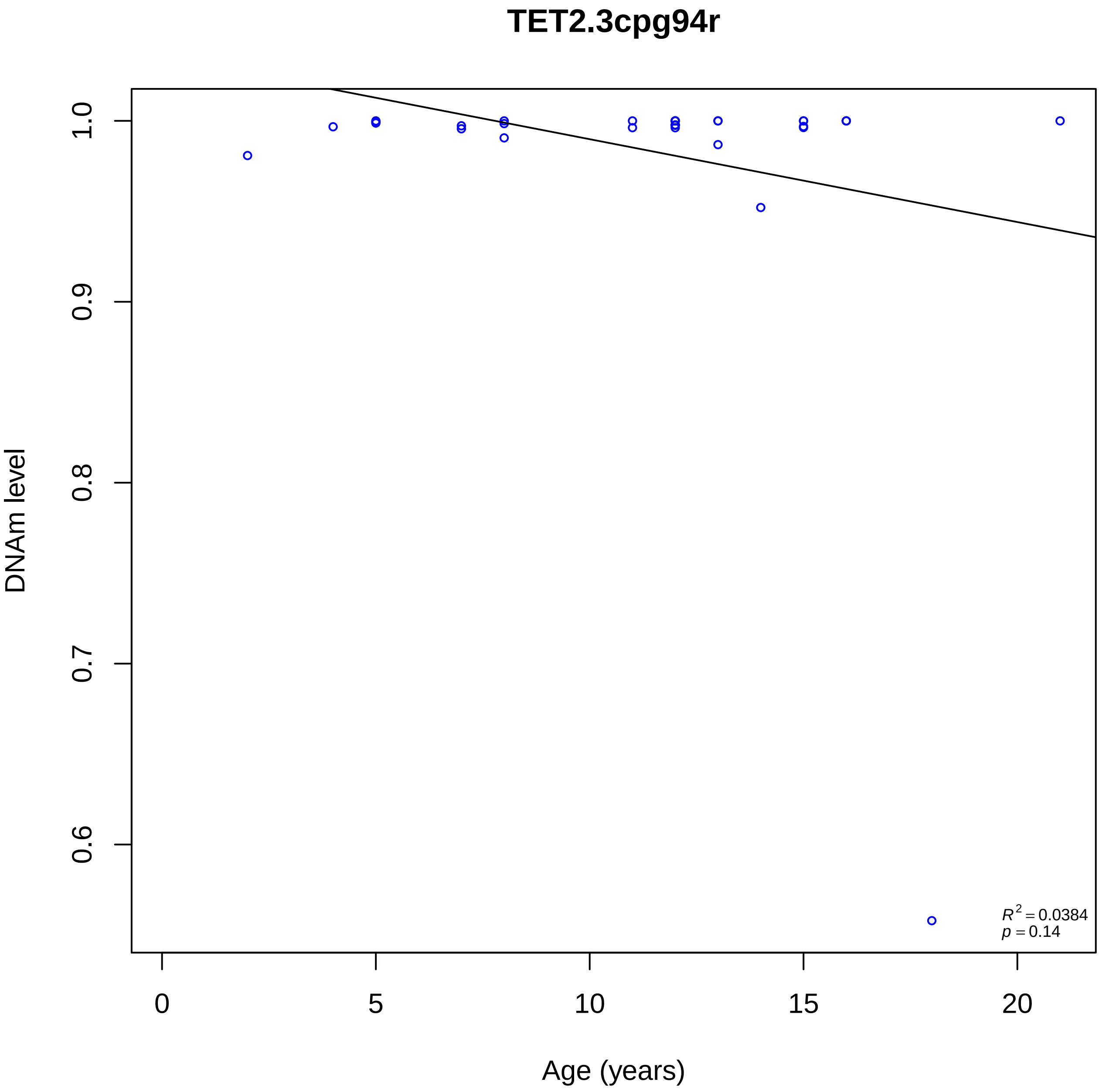
TET2.3cpg94r
DNAm level
Age (years)
0.6
0.7
0.8
0.9
1.0
0
5
10
15
20
$R^2 = 0.0384$
$p = 0.14$
